# Synthesis, Anticancer and Antitubercular Properties of New Chalcones and Their Nitrogen-Containing Five-Membered Heterocyclic Hybrids Bearing Sulfonamide Moiety

**DOI:** 10.3390/ijms232012589

**Published:** 2022-10-20

**Authors:** Lina Fernanda Castaño, Jairo Quiroga, Rodrigo Abonia, Daniel Insuasty, Oscar M. Vidal, Rosalia Seña, Vivian Rubio, Gloria Puerto, Manuel Nogueras, Justo Cobo, Juan Guzman, Alberto Insuasty, Braulio Insuasty

**Affiliations:** 1 Heterocyclic Compounds Research Group, Department of Chemistry, Universidad del Valle, A.A., Cali 25360, Colombia; 2 Centre for Bioinformatics and Photonics-CIBioFI, Universidad del Valle, A.A., Cali 25360, Colombia; 3 Department of Chemistry and Biology, Basic Sciences Division, Universidad del Norte, Barranquilla 081007, Colombia; 4 Department of Medicine, Health Division, Universidad del Norte, Barranquilla 081007, Colombia; 5 Grupo de Micobacterias, Red TB. Dirección de Investigación en Salud Pública, Instituto Nacional de Salud, Bogotá 111321, Colombia; 6 Department of Inorganic and Organic Chemistry, Universidad de Jaén, 23071 Jaén, Spain; 7 Department of Bioresources, Fraunhofer Institute for Molecular Biology and Applied Ecology, 35392 Giessen, Germany; 8 Nanostructured Functional Materials Research Group, Universidad CESMAG, Pasto 520003, Colombia

**Keywords:** anticancer, antituberculosis, chalcones, molecular hybrids, sulfonamides

## Abstract

A new series of sulfonamides, **8a-b, 10, 12**, and **14a-b**, were synthesized by *N*-sulfonation reaction with sulfonyl chlorides **6a-b**. Five new series of chalcone-sulfonamide hybrids **(16-20)a-f** were prepared via Claisen–Schmidt condensation of the newly obtained sulfonamides with aromatic aldehydes **15a-f** in basic medium. Chalcones substituted with chlorine at position 4 of each series were used as precursors for the generation of their five-membered heterocyclic pyrazoline (**22-23)a-d**, (**24-25**)**a-b** and carbothioamide **27a-f** derivatives. The synthesized compounds were evaluated for their anticancer and antituberculosis activities. To determine their anticancer activity, compounds were screened against sixty human cancer cell lines at a single dose (10 μM). Compounds **17a-c** were highly active against LOX IMVI (melanoma), with IC_50_ values of 0.34, 0.73 and 0.54 μM, respectively. Chalcone **18e** showed remarkable results against the entire panel of leukemia cell lines with IC_50_ values between 0.99–2.52 μM. Moreover, compounds **20e** and **20f** displayed growth inhibition of *Mycobacterium tuberculosis* H37Rv at concentrations below 10 μM. Although they showed low selectivity in cytotoxicity tests against the Vero cell line, further optimization could advance the potential biological activity of the selected compounds.

## 1. Introduction

Finding effective treatments for diseases with high mortality rates such as cancer and tuberculosis (TB) remains at the top of the biomedical research agenda worldwide. The number of fatalities associated with cancer have decreased over the last decades as a result of improvements in prevention, diagnosis and treatment [1]. However, the necessity to combine cytotoxic drugs in conventional chemotherapy demonstrates the difficulty in treating this complex and multifactorial disease [2]. TB is the second leading cause of mortality due to infectious agents after COVID [3], despite the availability of affordable and successful treatments [3]. The emergence of multidrug-resistant TB is one of the major threats to the control of the disease, and selecting a new combination of drugs to shorten the treatment period remains as a challenge [4,5,6].

Molecular hybridization, involving the combination of two or more pharmacophoric moieties into a single chemical entity, has become an attractive approach for overcoming limitations in the administration of active chemical entities for treating complex diseases. A successful hybrid compound should access multiple targets and lead to different mechanisms of action [2]. There are multiple known drug-like fragments, and we have selected the sulfonamide functionality in combination with a privileged small nitrogen heterocyclic such as pyrazole, or with its α,β-unsaturated ketone precursors such as chalcones.

On one hand, sulfonamide moiety prevails as the most promising candidate for the design of molecular hybrids [7]. The versatility of the chemical structure, being easily modified; the compatibility with most functional groups; a strong electron withdrawal nature; and the capacity to coordinate metal ions from metalloenzymes forming tetrahedral complexes stabilized by hydrogen bounds are remarkable features [8,9], without mentioning the variety of biological properties that sulfonamides have exhibited including anti-inflammatory [10], anticancer [11,12], antibacterial [13], antiviral [14], antidiabetic [15], and antimalarial [16] activities, among others.

**Figure 1:**
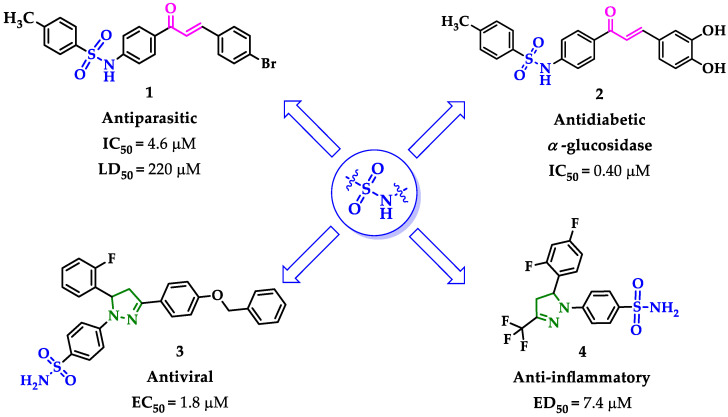
Sulfonamide moiety as promising candidate for molecular hybridization.

On the other hand, chalcones are compounds that have shown an extensive number of applications in the field of medicinal chemistry due to their wide range of pharmacological properties [17]. A wide variety of synthetic chalcones have been shown in applications such as analgesics [18], antioxidants [19], anti-inflammatories [20], antibacterial [21], antitumor [17], antiviral [22], antihypertensive [23], antidiabetic [24], and antituberculosis [25]. The biological activity of chalcones is attributed to their effect as cell blockers, influenced by the interactions of the α,β-unsaturated carbonyl system with various biomolecules [20,26]. The combination of sulfonamide moiety with chalcones to generate new hybrid compounds with potential therapeutic applications has been described [27,28] (Figure 1), and also used as key precursors for potentially bioactive heterocyclic derivatives [29,30,31,32].

Regarding pyrazoles, their structural and electronic properties allow them to interact with various biological entities involved in the development of several pathologies; moreover, they are responsible for the great variety of biological properties bearing this type of moiety [33]. In addition, it is known that compounds containing sulfur and nitrogen have received significant attention in the field of medicinal chemistry due to the ability of these atoms to generate donor ligands and coordinate complexes with metal cations of zinc, iron, nickel, and copper, which play important roles in different biological processes [34].

Synthetic pyrazoline derivatives have exhibited antitumor [35,36,37], antibacterial [38], immunosuppressive [39], anti-inflammatory [40], anti-diabetic [41], antidepressant [42], antimalarial [43], antiviral [44], and antihypertensive [45] activities. Particularly, the 4,5-dihydro-1*H*-pyrazole-1-carbothioamides have also shown a wide variety of pharmacological properties such as anticancer [46,47,48,49,50], anticonvulsant [51,52], antituberculosis [53], antimicrobial [54], and anti-inflammatory [55] agents (Figure 1).

In this paper we extend our ongoing research on chalcone derivatives by the synthesis of new nitrogen-containing five-membered heterocyclic hybrids bearing a sulfonamide moiety and performing the study of their anticancer and antituberculosis properties.

## 2. Results and Discussion

### 2.1. Chemistry

**Scheme 1:**
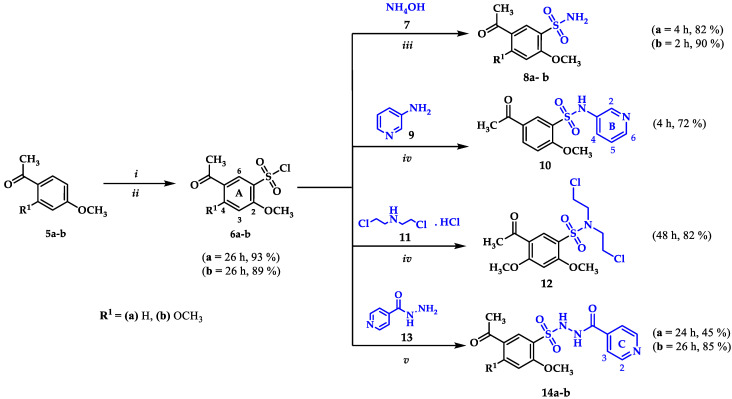
General procedure for the synthesis of sulfonamide precursors **8a-b**, **10**, **12,** and **14a-b** from benzenesulfonyl chlorides **6a-b**: (*i*) HSO_3_Cl, SOCl_2_, 0 °C, 0.5 h, (*ii*) r.t, 26 h, (*iii*) EtOH, r.t, (*iv*) EtOH, TEA, r.t, (*v*) Water, Na_2_CO_3_, r.t.

All hybrids were synthesized from the corresponding benzenesulfonyl chlorides **6a** and **6b**. In our previous work we described the procedure for the preparation of **6a** [56]. Compound **6b** was obtained by the chlorosulfonation reaction of acetophenone **5b** (Scheme 1), using chlorosulfonic acid and thionyl chloride as chlorinating agents in a 6:2 ratio regarding **5b**. The structure of **6b** was confirmed by spectroscopic techniques including FTIR, ^1^H NMR, ^13^C NMR and mass spectrometry. The IR spectrum shows absorption bands corresponding to =C-H, C=O, C=C and C-O-C bonds at 3110, 1657, 1590, and 1292 cm^−1^, respectively. An absorption band of the S=O bond was clearly observable at 1167 cm^−1^. The aliphatic region of the ^1^H NMR spectrum shows three singlets at 2.56, 4.11, and 4.05 ppm corresponding to COCH_3_, 4A-OCH_3_ and, 2A-OCH_3_ protons, respectively. In the aromatic region only two signals at 8.40 and 6.56 ppm were observed; the downfield signal corresponds to H_6A_ due the inductive effect generated by the sulfonyl group on the system. In the ^13^C NMR spectrum, ten signals were observed as expected for compound **6b**. The molecular ion peak in the mass spectrum revealed the characteristic isotopic profile for one chlorine atom *m/z* 278/280 (M^+^/(M+2)^+^).

**Figure 2:**
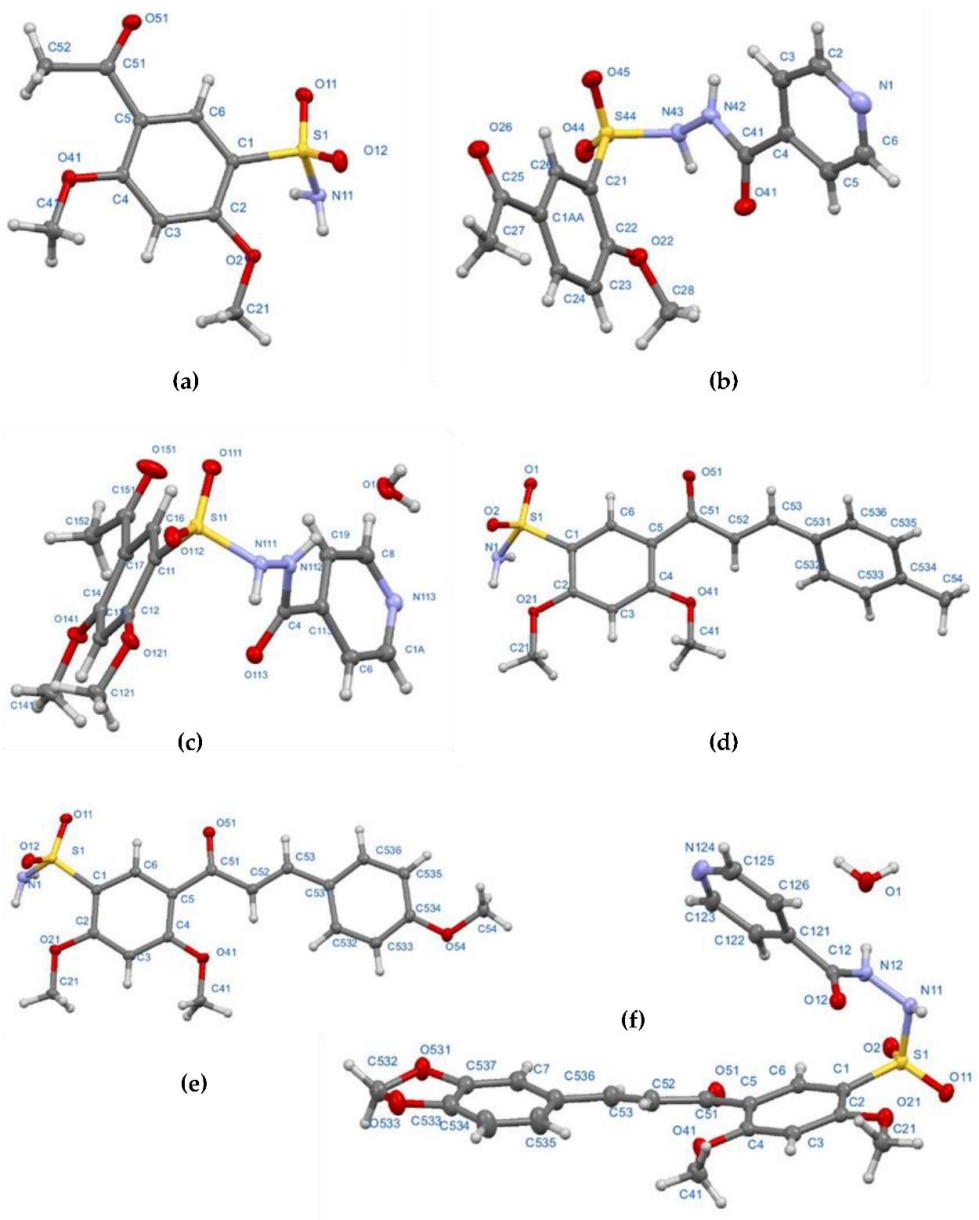
Molecular structure of compounds **8b** (**a**), **14a** (**b**), **14b** (**c**), **17c** (**d**), **17d** (**e**) and **20f** (**f**) determined from single-crystal X-ray diffraction, showing the atom-labelling scheme. Single crystal diffraction experimental details can be found in Table S1 (see Supplementary Materials).

Sulfonamides **8a** and **8b** were synthetized through *N*-sulfonation reactions of **6a-b** with ammonia at room temperature, using ethanol to promote product precipitation (Scheme 1). The IR spectra of both sulfonamides presents the two characteristic stretching vibration bands for a primary N-H bond in the region between 3342–3201 cm^−1^. The ^1^H NMR spectra confirms the structure of the compounds by the observation of broad singlets at 7.24 and 7.06 ppm corresponding to NH_2_ protons for **8a** and **8b**, respectively. The obtention of **8b** was also confirmed by single-crystal X-ray data (Figure 2a).

Compounds **10** and **12** were prepared using amines **9** and **11** and triethylamine to neutralize the reaction in ethanol as a solvent (Scheme 1), conditions reported in our previous work [55]. Compounds **10** and **12** were obtained in 72% and 82% yields. The substitution reaction for the synthesis of the secondary sulfonamide **10** was verified by observation in the ^1^H NMR spectrum of a singlet corresponding to the sulfonamidic proton at 10.45 ppm and the expected signals for the protons in the pyridinic ring H_2B_, H_6B,_ and H_4B_ at 8.30 (*J* = 1.7 Hz), 8.21 (d, *J* = 4.5 Hz), and 7.51 ppm (d, *J* = 8.3 Hz), in addition to the overlapped signal of proton H_5B_ with H_3A_ at 7.24 ppm. The sulfonamidic protons in compound **12** were absent and its structure was confirmed by the observed signals of protons N-CH_2_ and CH_2_ at 3.53 and 3.68 ppm.

In order to obtain the sulfonamides derived from isoniazid, the reaction between **6a** and **13** was carried out under the same reaction conditions used for the synthesis of **10** and **12**; however, a complex mixture of products was observed. A number of conditions were tested, including the use of KOH, NaOH and base-free; in all cases, either a complex mixture or traces of the desired product were obtained. To overcome this issue, an alternative route was sought, including a trial using water as solvent, Na_2_CO_3_ 1 N to keep a basic pH (8–10) level during the reaction and final work-up with HCl 1 N to reach pH 2–3 once the reaction was finished, but no product was isolated [57]. However, an adjustment of the final pH at 7–8 allowed compounds **14a** and **14b** in moderate yields (Scheme 1). The ^1^H NMR spectra showed all the expected signals for C-NH, S-NH (9.69–11.13 ppm), and the pyridinic protons of the target compounds. Both structures were elucidated by single-crystal X-ray diffraction (Figure 2b,c).

**Scheme 2:**
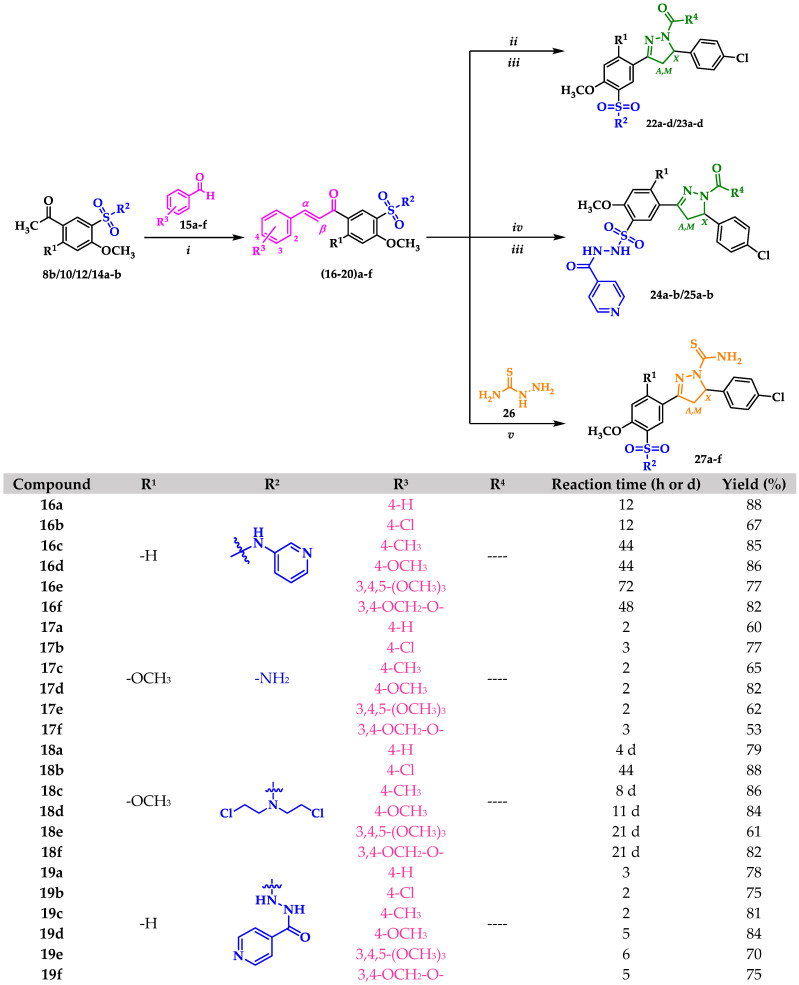

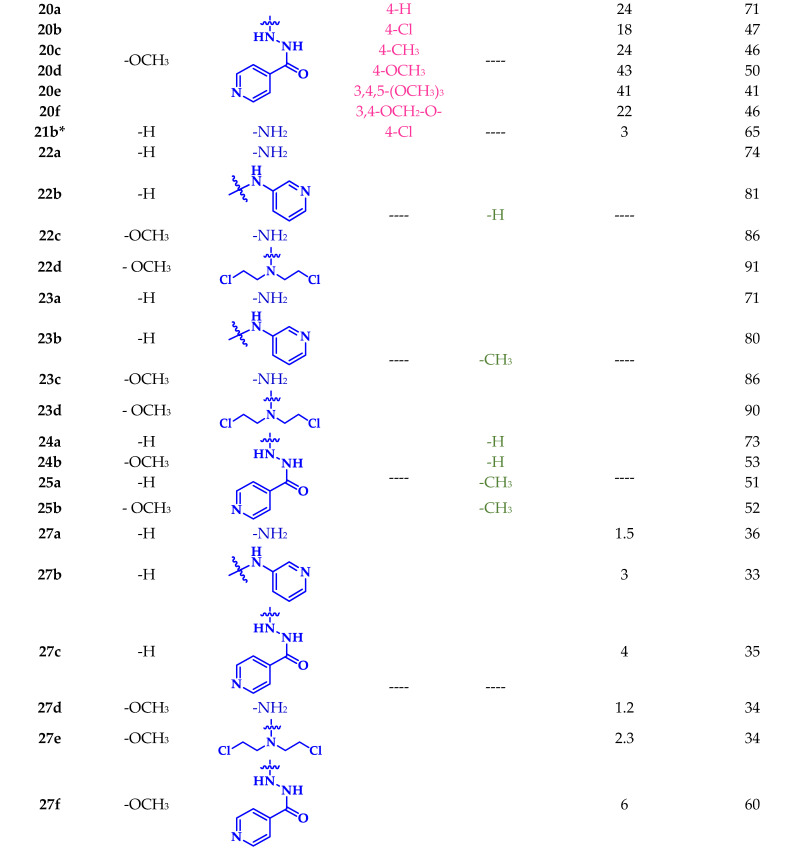
General procedure for the synthesis of new chalcone–sulfonamide **(16-20)a-f**, pyrazoline–sulfonamide **(22-23)a-d** and **(24-25)a-b**, and carbothioamide–sulfonamide **27a-f**: (*i*) EtOH, NaOH 50% (*w/v*), r.t; (*ii*) NH_2_-NH_2_∙H_2_O, EtOH, reflux; (*iii*) Ac_2_O or HCOOH, r.t; (*iv*) NH_2_-NH_2_∙H_2_O, EtOH, r.t; (*v*) EtOH, NaOH, 60 °C. * Reported in our previous work [56].

**Figure 3:**
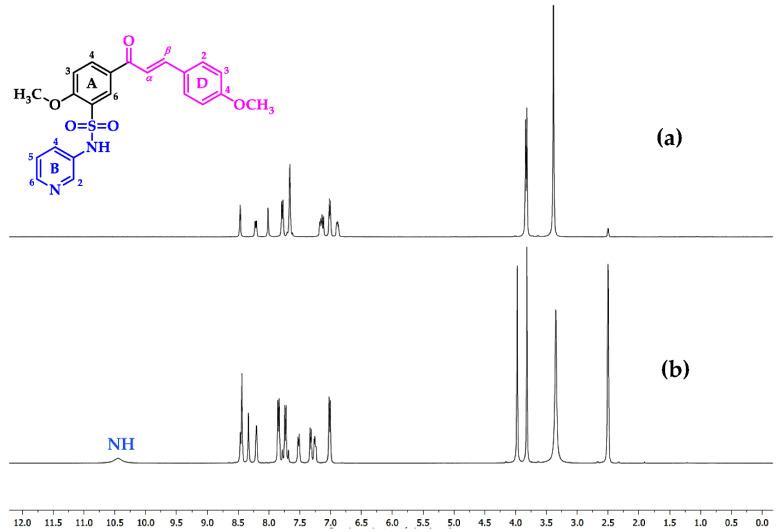
^1^H NMR spectrum (400 MHz, DMSO-*d_6_*) of compound **16d**: (**a**) salt form (basic media) and (**b**) neutral.

**Figure 4:**
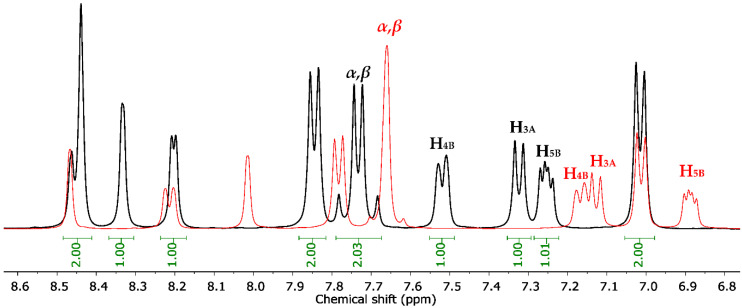
Chemical shift between ^1^H NMR signals of compound **16d** in salt form (red) and neutral (black).

Sulfonamides **8a-b**, **10**, **12,** and **14a-b** reacted with aromatic aldehydes (**15a-f**) by Claisen–Schmidt condensation in the presence of ethanol and aqueous NaOH (Scheme 2). This procedure resulted in good yields of the corresponding chalcone–sulfonamide hybrids **(16-20)a-f** (41–88%). Compounds **16a-f** and **17a-f** were initially obtained in salt form as evidenced in the ^1^H NMR spectrum of **16d** (Figure 3a and Figure 4 red). This behavior is observed due to the acidic character of the sulfonamidic protons by the strong electron withdrawing effect of the sulfonyl group and the resonance stabilization of the conjugate base formed in the basic reaction medium. Further neutralization of each compound with HCl 1% (*v/v*) allowed the isolation of **16d** in its neutral form (Figure 3b and Figure 4 black), and it was verified by the observation of a broad signal at 10.45 ppm corresponding to the sulfonamidic protons. Chalcones **18a-f** were directly obtained in their neutral form due to the absence of labile protons in their structures. The ^1^H NMR spectra for the five new series of chalcones showed the expected signals for H*_α_* and H*_β_*, confirming the condensation reaction. The coupling constants in the range 15.4–15.8 Hz determined the *E* configuration for the C=C double bond formed. In addition, the structures of compounds **17c-d** and **20f** were elucidated by single-crystal X-ray diffraction (Figure 2d–f).

For the synthesis of the nitrogen-containing five-membered heterocyclic hybrids the *p*-chloro-substituted chalcones of each series were selected. Compound **21b** was initially subjected to a cyclocondensation reaction with hydrazine monohydrate in ethanol under reflux; however, no significant progress in the reaction was observed. Thus, the same procedure was carried out, and after 40 min of reaction, the mixture was brought to room temperature, 1 mL of acetic anhydride was slowly added, and the progress of the reaction was checked. After 3 h a new product had formed, and the total consumption of the precursor was observed. The acid dependence of the reaction suggests that the carbonyl group must first be activated for the cyclization to proceed through a hydrazone-type intermediate [58]. In the ^1^H NMR spectrum, the absence of signals corresponding to *α,β*-unsaturation and the presence of one stereogenic and two diastereotopic protons revealed the obtention of the AMX spin system characteristic of pyrazoline derivatives. The spectrum of compound **23a** showed three doublet of doublets at 5.53, 3.85, and 3.09 ppm corresponding to H_X_, H_M_, and H_A_, with coupling constants of *^2^J_MA_* = 18.0 Hz, *^3^J_XM_* = 11.6 Hz, and *^3^J_XA_* = 4.3 Hz. Also, a singlet integrating for three protons at 2.28 ppm was assigned to the acetyl group and confirmed the functionalization of the product. The methodology was extended to the preparation of *N*-formylated (**22a-d**) and *N*-acetylated (**23a-d**) pyrazolines in good to excellent yields (Scheme 2).

Under the same reaction conditions, chalcones **19b** and **20b** decomposed according to the ^1^H NMR spectrum of the major product. In a second approach, the reaction was carried out at room temperature, and after 3 h the precursor was completely consumed, giving rise to pyrazolines **24a-b** and **25a-b** (Scheme 2).

Sulfonamide–carbothioamide hybrids **27a-f** were synthesized by condensation between chalcones **(16-21)b** and thiosemicarbazide in basic medium (Scheme 2). The reaction did not proceed at room temperature, but an increase to 60 °C was enough to observe the consumption of the precursor. The medium was neutralized with HCl 1% (*v/v*), the solid form was filtered, and the filtrate was purified by column chromatography on silica gel. This methodology afforded the expected compounds in moderate yields (33–60%). The structure of the new hybrids was consistent with the spectral data. Both ^1^H and ^13^C NMR revealed formation of the AMX system. Interestingly, the new carbothioamide protons denoted as C-NH_2_ were diastereotopic.

### 2.2. Anticancer Activity

**Figure 5:**
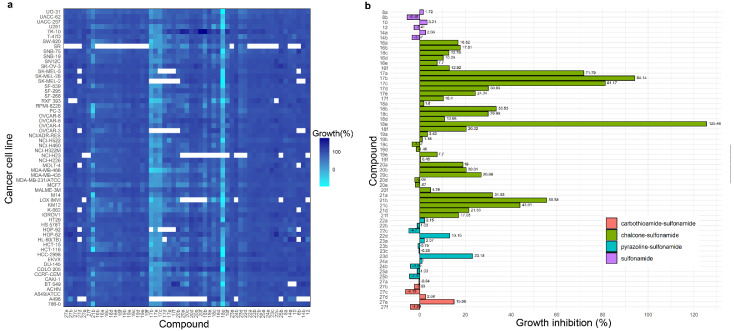
Anticancer activity of the synthesized hybrids at 10 µM. (**a**) Heatmap showing the growth of the cancer cells in presence of the compounds. Reduced growth of the cells is represented in cyan and dark blue represents the high growth of the cells. Blank cells indicate lack of data. (**b**) Mean growth inhibition (GI) percentage for each compound. A GI % value over 100% indicates there is no net growth of tumor cells over the course of the experiment; instead, compound causes the death of the respective cancer cell.

The new molecular hybrids bearing a sulfonamide moiety were submitted to the Developmental Therapeutics Program (DTP) at the National Cancer Institute (NCI) for a preliminary analysis of their structures based on the COMPARE algorithm. All synthesized compounds were selected for single-dose trial at a concentration of 10 μM against 60 human cancer cell lines corresponding to nine human cancer panels: leukemia, non-small-cell lung, melanoma, colon, central nervous system (CNS), ovary, renal, breast, and prostate cancer. The anticancer activity of the compounds was measured as a reduction in growth percentage for each of the cancer cell lines. Compounds **17a-c** and **18e** consistently reduced the growth of most cancer cells at 10 µM concentration (Figure 5).

Initial studies carried out at a single dose revealed that in general, sulfonamides (**8b**, **10**, **12**, **14a*,*** and **14b**), pyrazolines (**22a-d**, **23a-d**, **24a-b** and **25a-b**), and carbothioamides (**27a-f**) displayed low antiproliferative activity against the cancer cell lines evaluated, regardless of their substituent in the sulfonamide group and the *N*-1 of the five-membered rings. However, the chalcone–sulfonamide hybrids showed an increase in antitumoral activity, indicating the α,β-unsaturated carbonyl system could be responsible for imparting antiproliferative properties to the evaluated compounds.

A comparative analysis of the results obtained for the new series of chalcones and compounds **21a-f** (reported in our previous work) suggest that the antitumoral properties of these hybrids are influenced by the chlorine substituent in para position of the D ring. It is well known that the inclusion of halogenated atoms can modify the interaction strength and hydrophobicity of the molecules, increasing the specificity and selectivity in the processes of recognition, and binding with biomolecules such as proteins and DNA [59]. We found that the antitumoral activity is highly affected by steric factors in the sulfonamide substituent, being the chalcones containing the -NH_2_ group the most active and the isoniazid derivatives the least active [60].

Chalcones **17a-c** (4-H, 4-Cl, and 4-CH_3_, respectively) exhibited significant activity against most cancer cell lines evaluated. Compound **17a** displayed inhibitory activity against leukemia (GI = 70.81–94.75%), colon cancer (GI = 72.48–96.15%), renal cancer (GI = 66.21–100.00%), and breast cancer (GI = 73.49–100.00%) panels. This compound showed remarkable results against RXF 393 (renal cancer) and MDA-MB-468 (breast cancer) cell lines, being able to inhibit net growth and cause a cytotoxic effect with lethality values of 29.73% and 43.37%, respectively. Chalcone **17b** was the most active compound of the series and similar results were observed, leukemia (GI = 92.85–100.00%), colon cancer (GI = 92.23–100.00%), renal cancer (GI = 78.47–100.00%), and breast cancer (GI = 73.94–100.00%) panels were the most sensitive to this compound. Hybrid **17b** also displayed important cytotoxic effects against SR (leukemia) with a lethality of 19.50%; NCI-H226 (non-small cell lung cancer) with 25.96%; COLO 205, HCT-15, SW-620 (colon cancer) with values of 30.00%, 41.55%, and 39.92%, respectively; LOX IMVI (melanoma) with 50.40%; and RXF 393 (renal cancer) and MDA-MB-468 (breast cancer) with a 40.37% and 57.82% of lethality, respectively. Chalcone **17c** exhibited similar behavior to its analogs; the most sensitive panels were leukemia (GI = 78.87–94.34%), colon cancer (GI = 83.01–100.00%), and breast cancer (GI = 78.66–100.00%); and the cancer cell lines NCI-H460 (non-small cell lung cancer), RXF 393 (renal cancer), and MDA-MB-468 (breast cancer) with lethality values of 3.51%, 28.21%, and 36.18%, correspondingly.

Among the synthesized hybrids, compound **18e** was the most active against the totality of cancer cell lines as evidenced by a value of 125.48% in the mean GI%, denoting excellent inhibitory activity and significant cytotoxic effect in most assays. The GI% found for the evaluated cell lines rates between 76.62% and 100.00% except for HS 578T (21.70%). In several cases, compound **18e** displayed remarkable lethality and the best results were found for COLO 205, HCC-2998, and HCT-116 cell lines in the colon cancer panel with values of 89.76%, 91.08%, and 87.74%, respectively, but also for LOX IMVI and SK-MEL-2 from the melanoma panel with 84.53% and 94.49%, and for UO-31 (renal cancer) with a 93.86% of lethality.

According to the obtained data for single-dose trial, chalcone–sulfonamide hybrids **17a-c** and **18e** fulfilled the pre-determined threshold inhibition criteria of NCI and were selected for five-dose screening against 60 cancer cell lines at five different concentrations (100, 10, 1.0, 0.1, and 0.01 μM) to determine IC_50_ (half inhibitory concentration) and LC_50_ (half lethal concentration) values. Table 1 summarizes the recorded results for the mentioned compounds.

Results obtained from the five-dose screen revealed that compounds **17a**, **17b** and **17c** are potent antiproliferative agents, displaying IC_50_ values against most cell lines between 0.34−17.6 μM, 0.73−18.6 μM, and 0.54−11.5 μM, respectively. Despite the wide IC_50_ range showed by chalcone **18e** (0.99 - > 100 μM), the compound demonstrated potent activity against several cell lines.

Hybrid **17a** showed the best results against K-562 (leukemia) with IC_50_ = 1.50 μM, HCT-116 (colon cancer) with IC_50_ = 1.49 μM, LOX IMVI (melanoma) with IC_50_ = 0.34 μM, and MCF7 and MDA-MB-468 (breast cancer) with IC_50_ values of 0.97 and 1.20 μM, respectively. Compound **17b** was highly active against CCRF-CEM (leukemia) with IC_50_ = 1.52 μM, HOP-92 (non-small cell lung cancer) with IC_50_ = 1.46 μM, HCT-116 and HCT-15 (colon cancer) with IC_50_ values of 0.75 μM and 1.46 μM, and LOX IMVI (melanoma) with IC_50_ = 0.73 μM. This chalcone showed lower IC_50_ values in comparison with its analogs, exhibiting the best-marked cytotoxic effects against several cancer cell lines including BT-549 (breast cancer) with 5.97 μM, and COLO 205 and HCT-116 (colon cancer) with LC_50_ values of 5.88 μM and 5.13 μM, respectively. The highest IC_50_ values were obtained for chalcone **17c** against SNB-75 (CNS Cancer) at 1.84 μM, and LOX IMVI (melanoma) and MDA-MB-468 (breast cancer) with values of 0.54 μM and 1.96 μM. The entire leukemia panel was highly sensitive to compound **18e*,*** displaying IC_50_ values between 0.99–2.52 μM, the SR cancer cell line being most susceptible. Significant IC_50_ values were found for NCI-H460 and NCI-H522 (non-small cell lung cancer) with 1.95 μM and 1.66 μM, correspondingly. In addition, chalcone **18e** showed important cytotoxic activity against several cell lines including LOX IMVI (melanoma) and UO-31 (renal cancer) with respective LC_50_ values of 5.79 μM and 5.51 μM.

The results shown above suggest that compounds with structures analogous to **17b** constitute important templates for the future development of potential antitumor agents.

### 2.3. Antituberculosis Activity, Cytotoxicity and Synergism

All new sulfonamide hybrids were subjected to in vitro growth inhibition screening against *Mycobacterium bovis* BCG (ATCC 35734) and *Mycobacterium tuberculosis* H_37_Rv (ATCC 27294) strains (ATCC-USA, Virginia, USA). Antituberculosis activity was carried out using the agar dilution spot culture growth inhibition assay [61]. Initially, the compounds were evaluated at a concentration of 10 mg/L, and those showing inhibition were further evaluated at lower concentrations (5, 1, 0.5, 0.1 and 0.01 mg/L). Isoniazid was used as positive control. The results for each compound were expressed as minimum inhibitory concentration values (MICs), as shown in Table 2. The synthesized sulfonamides, pyrazolines and carbothioamides were inactive and only chalcones showed inhibitory effects with MIC values between 9.0–29 μM, showing the importance of an α,β-unsaturated carbonyl system as a pharmacophore unit. Isoniazid derivatives exhibited the most potent antitubercular activity. The substituents 4-H, 4-OCH_3_, 3,4,5-(OCH_3_)_3_, and 3,4-OCH_2_O- enhanced the inhibitory activity. The most active chalcones were **20a**, **20e,** and **20f** obtained from chloride **6b** (R^1^ = OCH_3_), displaying MIC values of 11 μM, 9.0 μM, and 9.8 μM against *Mycobacterium tuberculosis* H_37_Rv and **20a** and **20e** against *Mycobacterium bovis* BCG with MIC values of 11 μM and 9.0 μM, respectively. 

**Figure 6:**
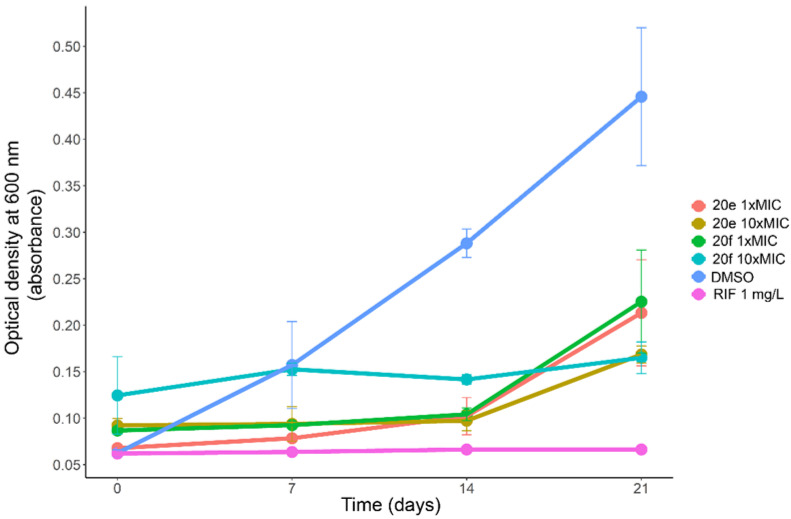
Growth curves of *M. tuberculosis* H_37_Rv in liquid media containing 1×MIC and 10×MIC concentrations of the compounds **20e** and **20f**.

The growth of *M. tuberculosis* H_37_Rv in liquid medium in presence of the compounds **20e** and **20f** at 1×MIC and 10×MIC concentrations was followed by optical density measurements (Figure 6). Both compounds showed similar potency in liquid medium with moderate growth observed after 21 days of incubation at 1×MIC, while at 10×MIC a modest growth was observed for **20e**, while for **20f** no significant growth was observed in comparison with day 0. The cultivation of solid media without compounds was undertaken for each sample for evaluating cidal or static effects of the compounds. Both **20e** and **20f** were found to be bacteriostatic even at 10×MIC concentrations, because growth resumed in all experiments.

Additionally, cytotoxicity studies were conducted on the Vero cell line for the mentioned hybrids as shown in Table 2. The results revealed low IC_50_ values (<10 μM) and therefore high cytotoxicity levels against Vero cells. Selectivity index (SI) was calculated for the screened compounds and none of the chalcones showed selectivity against *Mycobacterium* tuberculosis H_37_Rv strains compared to the Vero cell (SI < 1).

Additionally, the compounds with most potent activity were tested against a panel of *M. tuberculosis* strains from six different lineages that were resistant to isoniazid and rifampicin (Table 3), including an orphan strain with no match in spolDB4 [62]: A Beijing strain known to be an emerging pathogen in several areas [63,64]; a LAM 9 strain, which is the predominant genotypic family from Latin American and Mediterranean (LAM) lineage [65]; a Haarlem strain, which is ubiquitous worldwide [66]; the ATCC 35838 RR, RR (rifampicin-resistant strain); ATCC 35822 RI (isoniazid- resistant strain); and ATCC 27294 (Table 3).

All the evaluated compounds were inactive against *M. tuberculosis* Haarlem and *M. tuberculosis* ATCC 35838 RR (MIC >10 mg/L). On the other hand, compounds **20e** and **20f** were highly active against *M. tuberculosis* LAM 9 (SIT 42), showing MIC values of 8.97 µM and 9.77 µM, respectively. Against the *M. tuberculosis* Beijing strain, the compounds with lower MIC values were **20a** and **20e**. The compounds **19a**, **20a**, **20e*,*** and **20f** displayed MIC values <12 µM against the *M. tuberculosis* ATCC 35838 RR strain.

Moreover, the possibility of synergism between compounds **20a** and **20f** in combination with antibiotics rifampicin, isoniazid, levofloxacin, and amikacin was evaluated and the results are shown in Table 4. Compound **20f** in combination with rifampicin displayed a fractional inhibitory concentration index (FICI) of 0.37 (≤0.5), suggesting synergism. The other combinations of compounds and TB drugs did not show interaction.

## 3. Materials and Methods

### 3.1. General

All chemicals and solvents were purchased from Sigma-Aldrich and Merck unless stated otherwise. Melting points were measured using a Stuart SMP10 melting point device (Bibby Scientific Ltd., Staffordshire, UK) and are uncorrected. ATR-FTIR spectra were recorded on a Shimadzu IRAffinity–1 (Shimadzu Corp., Columbia, USA). The ^1^H and ^13^C NMR spectra were run on a BRUKER DPX 400 spectrometer (Bruker, Billerica, USA) operating at 400 and 100 MHz, respectively, using DMSO–*d_6_* and CDCl_3_ as solvents and TMS as internal standard. Elemental analyses were performed on a Thermo Finnigan Flash EA1112 CHN elemental analyzer (Thermo Fischer Scientific Inc., Madison, USA) and the values are within ±0.4% of the theoretical values. The mass spectra were recorded on a SHIMADZU–GCMS–QP 2010 spectrometer (Shimadzu Corp., Kyoto, Japan) with an electronic impact source operating at 70 eV. Thin layer chromatography (TLC) was performed on 0.2 mm pre-coated aluminum plates of silica gel 60 F_254_ (Merck_,_ Dramstand, Germany) and spots visualized with ultraviolet irradiation. The single-crystal X-ray data were collected in a Diffractometer Bruker D8 Venture (Bruker Daltonics GmbH & Co. KG, Bremen, Germany) at “Centro de Instrumentación Científico y Técnico”, (CICT) in “Universidad de Jaén” (UJA).

### 3.2. Chemistry

#### 3.2.1. General Procedure for the Synthesis of Acetophenone **5b**

Three hundred and forty-two milligrams of crushed KOH (6.10 mmol) and 9 mL of DMSO were stirred for 30 min at room temperature. To this mixture, a solution of 422 mg of 2’,4’-dihydroxyacetophenone (2.77 mmol) in 6 mL of DMSO was added and stirred for 15 min, then cooled in an ice-water bath. A quantity of 0.40 mL of methyl iodide (6.42 mmol) was slowly added and left under constant stirring at room temperature for 24 h.

The reaction mixture was poured into ice-water and extracted with chloroform; the organic phase was evaporated under reduced pressure and the product purification was carried out by CC, using silica gel as stationary phase and a mixture of CHCl_3_: petroleum ether (20:1) as mobile phase.

##### 1-(2,4-Dimethoxyphenyl)ethan-1-one (**5b**)

Beige solid; 79% yield; m.p. 37–38 °C. FTIR (ATR) υ(cm^−1^): 3011 (C-H Ar), 1650 (C=O), 1599 (C=C), 1255 (C-O-C (a)), 1018 (C-O-C (s)). ^1^H NMR (400 MHz, CDCl_3_) δ ppm 7.82 (d, *J* = 8.8 Hz, 1H, H_6A_), 6.51 (dd, *J* = 8.8, 2.3 Hz, 1H, H_1A_), 6.45 (d, *J* = 2.3 Hz, 1H, H_3A_), 3.88 (s, 3H, 4A-OCH_3_), 3.84 (s, 3H, 2A-OCH_3_), 2.56 (s, 3H, CH_3_). ^13^C NMR (101 MHz, CDCl_3_) δ ppm 197.9 (C), 164.7 (C), 161.2 (C), 132.8 (CH), 121.3 (C), 105.2 (CH), 98.4 (CH), 55.64 (CH_3_), 55.56 (CH_3_), 31.9 (CH_3_). MS (EI, *m*/*z* (%)): 180 (M^+^, 57), 165 (100), 122 (44), 107 (35), 77 (45), 43 (38). Anal. calcd. for C_10_H_12_O_3_: C, 66.65; H, 6.71. Found: C, 66.50; H, 6.89.

#### 3.2.2. General Procedure for the Synthesis of Benzenesulfonyl Chloride **6b**

A mixture of chlorosulfonic acid (60 mmol) and thionyl chloride (20 mmol) was stirred in an ice bath for 30 min. Subsequently, corresponding acetophenone **1a-b** (10 mmol) was added portion-wise and the reaction mixture was stirred at room temperature for 26 h. Next, it was quenched in ice-water and the precipitate obtained was filtered and washed with water. Compound **6b** was purified by CC using silica gel as stationary phase and a mixture of CHCl_3_: EtOAc (10:1) as mobile phase. Product **6a** did not require further purification. NMR spectra of all synthesized compounds from here in, can be found in File S1 (see Supplementary Materials).

##### 5-Acetyl-2,4-dimethoxybenzenesulfonyl Chloride (**6b**)

White solid; 89% yield; m.p. 152–155 °C. FTIR (ATR) υ(cm^−1^): 3110 (C-H Ar), 1657 (C=O), 1590 (C=C), 1292 (C-O-C), 1167 (S=O). ^1^H NMR (400 MHz, CDCl_3_) δ ppm 8.40 (s, 1H, H_6A_), 6.56 (s, 1H, H_3A_), 4.11 (s, 3H, 4A-OCH_3_), 4.05 (s, 3H, 2A-OCH_3_), 2.56 (s, 3H, CH_3_). ^13^C NMR (101 MHz, CDCl_3_) δ ppm 195.5 (C), 165.9 (C), 161.8 (C), 133.9 (CH), 124.7 (C), 120.4 (C), 96.2 (CH), 57.2 (CH_3_), 56.6 (CH_3_), 31.8 (CH_3_). MS (EI, *m*/*z* (%)): 278/280 (M^+^/M+2^+^, 31/12), 263 (100), 149 (35), 135 (49), 43 (54). Anal. calcd. for C_10_H_11_ClO_5_S: C, 43.09; H, 3.98; S, 11.50. Found: C, 43.12; H, 3.88; S, 11.54.

#### 3.2.3. General Procedure for the Synthesis of Sulfonamides **8a-b**

A mixture of the appropriate chloride **6a-b** (0.31 mmol) and ammonia **3** (1 mL) in ethanol 96% (1 mL) was stirred at room temperature and the progress was monitored by TLC. After completion, the solid formed was filtered and washed with ethanol and water. Compounds **8a-b** did not require further purification.

##### 5-Acetyl-2-methoxybenzenesulfonamide (**8a**)

Pale pink crystals; 82% yield; m.p. 195–198 °C. FTIR (ATR) ν(cm^−1^): 3329 and 3208 (N-H), 3098 (C-H Ar), 1657 (C=O), 1159 (S=O). ^1^H NMR (400 MHz, DMSO-*d_6_*) δ ppm 8.29 (d, *J* = 2.3 Hz, 1H, H_6A_), 8.19 (dd, *J* = 8.7, 2.3 Hz, 1H, H_4A_), 7.32 (d, *J* = 8.7 Hz, 1H, H_3A_), 7.24 (s, 2H, NH_2_), 4.00 (s, 3H, OCH_3_), 2.56 (s, 3H, CH_3_). ^13^C NMR (101 MHz, DMSO-*d_6_*) δ ppm 195.7 (C), 159.6 (C), 134.4 (CH), 131.4 (C), 128.8 (C), 127.7 (CH), 112.6 (CH), 56.7 (CH_3_), 26.4 (CH_3_). MS (EI, *m/z* (%)): 229 (M^+^, 22), 214 (100), 119 (25), 76 (57), 43 (87). Anal. calcd. for C_9_H_11_NO_4_S: C, 47.15; H, 4.84; N, 6.11; S, 13.98. Found: C, 47.09; H, 4.93; N, 6.15; S, 14.01.

##### 5-Acetyl-2,4-dimethoxybenzenesulfonamide (**8b**)

Colorless crystals; 90% yield; m.p. 226–228 °C. FTIR (ATR) υ(cm^−1^): 3342 and 3201 (N-H), 3121 (C-H Ar), 1650 (C=O), 1155 (S=O). ^1^H NMR (400 MHz, DMSO-*d*_6_) δ ppm 8.09 (s, 1H, H_6A_), 7.06 (s, 2H, NH_2_), 6.83 (s, 1H, H_3A_), 4.02 (s, 6H, 2A-OCH_3_ and 4A-OCH_3_), 2.50 (s, 3H, CH_3_). ^13^C NMR (101 MHz, DMSO-*d*_6_) δ ppm 195.6 (C), 163.5 (C), 160.8 (C), 130.4 (CH), 123.9 (C), 118.5 (C), 97.1 (CH), 56.7 (CH_3_), 56.6 (CH_3_), 31.6 (CH_3_). MS (EI, *m*/*z* (%)): 259 (M^+^, 95), 244 (100), 134 (91), 76 (79), 43 (100). Anal. calcd. for C_10_H_13_NO_5_S: C, 46.32; H, 5.05; N, 5.40; S, 12.37. Found: C, 46.43; H, 5.05; N, 5.42; S, 12.30. Crystals suitable for single-crystal X-ray diffraction were obtained from ethanolic solution, and the crystal data for **8b** were deposited at CCDC with reference CCDC2191609.

#### 3.2.4. General Procedure for the Synthesis of Sulfonamide **10**

To a mixture of chloride **6a** (0.33 mmol) and amine **9** (0.36 mmol) in ethanol 96% (1.5 mL), TEA (0.15 mL) was added and left under stirring at room temperature. Once the reaction was complete, the medium was neutralized with TEA and the solid obtained was filtered and washed with ethanol to afford compound **10**. No further purification was required.

##### 5-Acetyl-2-methoxy-*N*-(pyridin-3-yl)benzenesulfonamide (**10**)

Pink solid; 72% yield; m.p. 217–218 °C. FTIR (ATR) υ(cm^−1^): 3073 (C-H Ar), 1682 (C=O), 1595 (C=C), 1267 (C-N), 1152 (S=O). ^1^H NMR (400 MHz, DMSO-*d*_6_) δ ppm 10.45 (s, 1H, NH), 8.30 (d, *J* = 1.7 Hz, 1H, H_2B_), 8.27 (d, *J* = 2.0 Hz, 1H, H_6A_), 8.21 (d, *J* = 4.5 Hz, 1H, H_6B_), 8.18 (dd, *J* = 8.8, 2.0 Hz, 1H, H_4A_), 7.51 (d, *J* = 8.3 Hz, 1H, H_4B_), 7.30–7.24 (m, 2H, H_3A_ and H_5B_), 3.94 (s, 3H, OCH_3_), 2.52 (s, 3H, CH_3_). ^13^C NMR (101 MHz, DMSO-*d*_6_) δ ppm 195.9 (C), 159.8 (C), 145.3 (CH), 141.5 (CH), 136.2 (CH), 134.5 (C), 130.2 (CH), 129.2 (C), 127.4 (CH), 126.4 (C), 124.3 (CH), 113.2 (CH), 57.0 (CH_3_), 26.6 (CH_3_). MS (EI, *m*/*z* (%)): 306 (M^+^, 40), 213 (46), 119 (97), 91 (63), 43 (100), 39 (51). Anal. calcd. for C_14_H_14_N_2_O_4_S: C, 54.89; H, 4.61; N, 9.14; S, 10.47. Found: C, 54.90; H, 4.68; N, 9.13; S, 10.42.

#### 3.2.5. General Procedure for the Synthesis of Sulfonamide **12**

To a mixture of chloride **6b** (0.33 mmol) and amine **11** (0.36 mmol) in ethanol (1.5 mL), TEA (0.15 mL) was added and left under stirring at room temperature. Once the reaction was complete, the medium was neutralized with TEA and the solid obtained was filtered and washed with ethanol. Compound **12** did not require further purification.

##### 5-Acetyl-*N*,*N*-bis(2-chloroethyl)-2,4-dimethoxybenzenesulfonamide (**12**)

White solid; 82% yield; m.p. 190–192 °C. FTIR (ATR) υ(cm^−1^): 3111 (C-H Ar), 2982 (C-H), 1661 (C=O), 1591 (C=C), 1143 (S=O). ^1^H NMR (400 MHz, DMSO-*d*_6_) δ ppm 8.12 (s, 1H, H_6A_), 6.87 (s, 1H, H_3A_), 4.06–4.01 (m, 6H, 2A-OCH_3_ and 4A-OCH_3_), 3.68 (t, *J* = 6.8 Hz, 4H, Cl-CH_2_), 3.53 (t, *J* = 6.8 Hz, 4H, N-CH_2_), 2.51 (s, 3H, CH_3_). ^13^C NMR (101 MHz, DMSO-*d*_6_) δ ppm 195.4 (C), 164.4 (C), 161.1 (C), 133.3 (CH), 119.1 (C), 119.0 (C), 97.6 (CH), 57.0 (CH_3_), 56.8 (CH_3_), 49.6 (CH_2_), 42.1 (CH_2_), 31.7 (CH_3_). MS (EI, *m*/*z* (%)): 384 (M^+^, 1), 334 (83), 243 (100), 195 (50), 149 (76). Anal. calcd. for C_14_H_19_Cl_2_NO_5_S: C, 43.76; H, 4.98; N, 3.65; S, 8.34. Found: C, 43.92; H, 5.06; N, 3.60; S, 8.17.

#### 3.2.6. General Procedure for the Synthesis of Sulfonamides **14a-b**

To a mixture of isoniazid **13** (0.29 mmol) and chloride **6a** for **14a** or **6b** for **14b** (0.31 mmol) in water (2 mL), 1 N sodium carbonate was added to reach pH 8–10 and was stirred at room temperature maintaining this pH. Once the reaction was finished, the pH was adjusted to 7–8 by adding HCl 10% *v/v* if necessary, the precipitate was filtered and washed with water. No further purification was required.

##### 5-Acetyl-*N*’-isonicotinoyl-2-methoxybenzenesulfonohydrazide (**14a**)

Beige crystals; 45% yield; m.p. 191–194 °C; FTIR (ATR) υ(cm^−1^): 3303 (N-H), 3234 (N-H), 3091 (C-H Ar), 1666 (C=O), 1595 (C=C), 1162 (S=O). ^1^H NMR (400 MHz, DMSO-*d*_6_) δ ppm 11.13 (s, 1H, S-NH), 10.08 (s, 1H, C-NH), 8.87 (s, 2H, H_2C_), 8.46–8.33 (m, 2H, H_6A_ and H_4A_), 7.76 (s, 2H, H_3C_), 7.49 (d, *J* = 8.7 Hz, 1H, H_3A_), 4.16 (s, 3H, OCH_3_), 2.68 (s, 3H, CH_3_). ^13^C NMR (101 MHz, DMSO-*d*_6_) δ ppm 195.6 (C), 164.1 (C), 161.0 (C), 150.0 (CH), 139.3 (C), 135.7 (CH), 129.7 (CH), 128.5 (C), 127.4 (C), 121.4 (CH), 113.0 (CH), 57.0 (CH_3_), 26.4 (CH_3_). MS (EI, *m*/*z* (%)): 349 (M^+^, 1), 106 (100), 78 (66), 51 (50), 43 (52). Anal. calcd. for C_15_H_15_N_3_O_5_S: C, 51.57; H, 4.33; N, 12.03; S, 9.18. Found: C, 51.60; H, 4.41; N, 12.03; S, 9.16. Crystals suitable for single-crystal X-ray diffraction were obtained from ethanolic solution, and the crystal data for **14a** were deposited at CCDC with reference CCDC 2191607.

##### 5-Acetyl-*N*’-isonicotinoyl-2,4-dimethoxybenzenesulfonohydrazide (**14b**)

Pale yellow crystals; 85% yield; m.p. 188–189 °C. FTIR (ATR) υ(cm^−1^): 3163 (C-H Ar), 1676 (C=O), 1659 (C=O), 1592 (C=C), 1166 (S=O), 1288 (C-N). ^1^H NMR (400 MHz, DMSO-*d*_6_) δ ppm 10.91 (d, *J* = 3.6 Hz, 1H, S-NH), 9.69 (d, *J* = 3.6 Hz, 1H, C-NH), 8.69 (d, *J* = 5.0 Hz, 2H, H_2C_), 8.06 (s, 1H, H_6A_), 7.57 (d, *J* = 5.0 Hz, 2H, H_3C_), 6.82 (s, 1H, H_3A_), 4.03–4.00 (m, 6H, 2A-OCH_3_ and 4A-OCH_3_), 2.46 (s, 3H, CH_3_). ^13^C NMR (101 MHz, DMSO-*d*_6_) δ ppm 195.3 (C), 164.5 (C), 164.1 (C), 162.4 (C), 150.3 (CH), 139.1 (C), 132.8 (CH), 121.2 (CH), 119.2 (C), 118.5 (C), 97.3 (CH), 57.1 (CH_3_), 56.7 (CH_3_), 31.6 (CH_3_). MS (EI, *m*/*z* (%)): 379 (M^+^, 6), 243 (40), 149 (32), 106 (88), 78 (82), 43 (100). Anal. calcd. for C_16_H_17_N_3_O_6_S: C, 50.65; H, 4.52; N, 11.08; S, 8.45. Found: C, 50.50; H, 4.52; N, 11.13; S, 8.40. Crystals suitable for single-crystal X-ray diffraction were obtained from ethanolic solution, and the crystal data for **14b** were deposited at CCDC with reference CCDC 2191610.

#### 3.2.7. General Procedure for the Synthesis of Chalcone–Sulfonamide Hybrids **16a-f** and **17a-f**

To a mixture of sulfonamide **8b** or **10** (0.20 mmol) and the corresponding aldehyde **15a-f** (0.24 mmol) in ethanol (1.5 mL), 2 drops of 50% (*w/v*) NaOH solution were added and the reaction mixture was stirred at room temperature. The sodium salt formed was filtered and washed with small portions of ethanol. Next, it was poured into water and neutralized by adding 10% (*w/v*) HCl aqueous solution. The precipitate was filtered and washed with water to afford the corresponding compounds **16a-f** and **17a-f**. No further purification was required.

##### 5-Cinnamoyl-2-methoxy-*N*-(pyridin-3-yl)benzenesulfonamide (**16a**)

White solid; 88% yield; m.p. 229–230 °C. FTIR (ATR) υ(cm^−1^): 3079 (C-H Ar), 1659 (C=O), 1601 (C=C), 1340 (C-N), 1158 (S=O). ^1^H NMR (400 MHz, DMSO-*d*_6_) δ ppm 8.48 (d, *J* = 1.4 Hz, 1H, H_6A_), 8.23 (dd, *J* = 8.4, 1.4 Hz, 1H, H_4A_), 8.01 (d, *J* = 1.8 Hz, 1H, H_2B_), 7.86–7.75 (m, 3H, H*_α_* and H_2D_), 7.72–7.61 (m, 2H, H*_β_* and H_6B_), 7.50–7.41 (m, 3H, H_3D_ and H_4D_), 7.20–7.08 (m, 2H, H_3A_ and H_4B_), 6.88 (dd, *J* = 8.1, 4.6 Hz, 1H, H_5B_), 3.84 (s, 3H, OCH_3_). ^13^C NMR (101 MHz, DMSO-*d*_6_) δ ppm 187.4 (C), 160.4 (C), 146.8 (C), 143.6 (CH), 143.4 (CH), 137.0 (CH), 134.8 (C), 134.7 (C), 132.5 (CH), 130.6 (CH), 129.9 (CH), 129.0 (CH), 128.9 (C), 128.8 (CH), 125.5 (CH), 122.9 (CH), 121.9 (CH), 112.1 (CH), 56.2 (CH_3_). MS (EI, *m*/*z* (%)): 394 (M^+^, 8), 368 (36), 236 (26), 97 (56), 57 (100), 43 (77). Anal. calcd. for C_21_H_18_N_2_O_4_S: C, 63.94; H, 4.60; N, 7.10; S, 8.13. Found: C, 63.90; H, 4.69; N, 7.24; S, 8.10.

##### (*E*)-5-(3-(4-Chlorophenyl)acryloyl)-2-methoxy-*N*-(pyridin-3-yl)benzenesulfonamide (**16b**)

White solid; 67% yield; m.p. 256–258 °C. FTIR (ATR) υ(cm^−1^): 3068 (C-H Ar), 1661 (C=O), 1604 (C=C), 1312 (C-N), 1152 (S=O). ^1^H NMR (400 MHz, DMSO-*d*_6_) δ ppm 8.47 (d, *J* = 2.3 Hz, 1H, H_6A_), 8.26 (dd, *J* = 8.7, 2.3 Hz, 1H, H_4A_), 8.04 (d, *J* = 2.6 Hz, 1H, H_2B_), 7.86 (d, *J* = 8.6 Hz, 2H, H_2D_), 7.82 (d, *J* = 15.7 Hz, 1H, H*_α_*), 7.71 (dd, *J* = 4.5, 1.5 Hz, 1H, H_6B_), 7.67 (d, *J* = 15.7 Hz, 1H, H*_β_*), 7.51 (d, *J* = 8.6 Hz, 2H, H_3D_), 7.23–7.18 (m, 1H, H_4B_), 7.15 (d, *J* = 8.7 Hz, 1H, H_3A_), 6.92 (dd, *J* = 8.3, 4.5 Hz, 1H, H_5B_), 3.84 (s, 3H, OCH_3_). ^13^C RMN (101 MHz, DMSO-*d*_6_) δ ppm 187.3 (C), 160.5 (C), 146.4 (C), 143.5 (CH), 142.0 (CH), 137.3 (CH), 135.0 (C), 134.6 (C), 133.7 (C), 132.7 (CH), 130.5 (CH), 129.9 (CH), 129.0 (CH), 128.9 (C), 125.6 (CH), 122.9 (CH), 122.7 (CH), 112.1 (CH), 56.2 (CH_3_). MS (EI, *m*/*z* (%)): 428/430 (M^+^/M+2^+^, 9/3), 313 (29), 236 (37), 97 (60), 57 (100). Anal. calcd. for C_21_H_17_ClN_2_O_4_S: C, 58.81; H, 4.00; N, 6.53; S, 7.48. Found: C, 58.79; H, 3.93; N, 6.50; S, 7.48.

##### (*E*)-2-Methoxy-*N*-(pyridin-3-yl)-5-(3-(*p*-tolyl)acryloyl)benzenesulfonamide (**16c**)

Beige solid; 85% yield; m.p. 231–234 °C. FTIR (ATR) υ(cm^−1^): 2947 (C-H), 1659 (C=O), 1598 (C=C), 1339 (C-N), 1156 (S=O). ^1^H NMR (400 MHz, DMSO-*d*_6_) δ ppm 8.47 (d, *J* = 2.4 Hz, 1H, H_6A_), 8.22 (dd, *J* = 8.6, 2.4 Hz, 1H, H_4A_), 8.01 (d, *J* = 2.6 Hz, 1H, H_2B_), 7.75–7.64 (m, 5H, H*_α_*, H*_β_*, H_2D_ and H_6B_), 7.27 (d, *J* = 7.7 Hz, 2H, H_3D_), 7.19–7.11 (m, 2H, H_3A_ and H_4B_), 6.89 (dd, *J* = 8.3, 4.6 Hz, 1H, H_5B_), 3.84 (s, 3H, OCH_3_), 2.35 (s, 3H, CH_3_). ^13^C NMR (101 MHz, DMSO-*d*_6_) δ ppm 187.3 (C), 160.3 (C), 146.8 (C), 143.6 (CH), 143.5 (CH), 140.6 (C), 137.0 (CH), 134.7 (C), 132.4 (CH), 132.0 (C), 129.8 (CH), 129.6 (CH), 129.0 (C), 128.8 (CH), 125.4 (CH), 122.8 (CH), 120.8 (CH), 112.0 (CH), 56.2 (CH_3_), 21.1 (CH_3_). MS (EI, *m*/*z* (%)): 408 (M^+^, 38), 315 (24), 178 (36), 145 (68), 115 (78), 39 (100). Anal. calcd. for C_22_H_20_N_2_O_4_S: C, 64.69; H, 4.94; N, 6.86; S, 7.85. Found: C, 64.70; H, 5.01; N, 6.87; S, 7.79.

##### (*E*)-2-Methoxy-5-(3-(4-methoxyphenyl)acryloyl)-*N*-(pyridin-3-yl)benzenesulfonamide (**16d**)

Pale yellow solid; 86% yield; m.p. 228–230 °C. FTIR (ATR) υ(cm^−1^): 3072 (C-H Ar), 1653 (C=O), 1593 (C=C), 1220 (C-O-C), 1159 (S=O). ^1^H NMR (400 MHz, DMSO-*d*_6_) δ ppm 8.47 (d, *J* = 2.4 Hz, 1H, H_6A_), 8.21 (dd, *J* = 8.6, 2.4 Hz, 1H, H_4A_), 8.01 (d, *J* = 2.6 Hz, 1H, H_2B_), 7.78 (d, *J* = 8.3 Hz, 2H, H_2D_), 7.71–7.61 (m, 3H, H*_α_*, H*_β_* and H_6B_), 7.18–7.10 (m, 2H, H_4B_ and H_3A_), 7.01 (d, *J* = 8.3 Hz, 2H, H_3D_), 6.89 (dd, *J* = 8.3, 4.6 Hz, 1H, H_5B_), 3.84 (s, 3H, 2A-OCH_3_), 3.82 (s, 3H, 4D-OCH_3_). ^13^C NMR (101 MHz, DMSO-*d*_6_) δ ppm 187.2 (C), 161.3 (C), 160.2 (C), 146.8 (C), 143.6 (CH), 143.4 (CH), 137.0 (CH), 134.6 (C), 132.3 (CH), 130.6 (CH), 129.8 (CH), 129.2 (C), 127.3 (C), 125.4 (CH), 122.8 (CH), 119.3 (CH), 114.5 (CH), 112.0 (CH), 56.2 (CH_3_), 55.4 (CH_3_). MS (EI, *m*/*z* (%)): 424 (M^+^, 98), 237 (58), 161 (85), 133 (81), 95 (100), 39 (77). Anal. calcd. for C_22_H_20_N_2_O_5_S: C, 62.25; H, 4.75; N, 6.60; S, 7.55. Found: C, 62.30; H, 4.82; N, 6.61; S, 7.47.

##### (*E*)-2-Methoxy-*N*-(pyridin-3-yl)-5-(3-(3,4,5-trimethoxyphenyl)acryloyl)benzenesulfonamide (**16e**)

Yellow solid; 77% yield; m.p. 239–241 °C. FTIR (ATR) υ(cm^−1^): 3077 (C-H Ar), 1660 (C=O), 1595 (C=C), 1272 (C-O-C), 1126 (S=O). ^1^H NMR (400 MHz, DMSO-*d*_6_) δ ppm 8.49 (d, *J* = 2.3 Hz, 1H, H_6A_), 8.27 (dd, *J* = 8.7, 2.3 Hz, 1H, H_4A_), 8.02 (d, *J* = 2.6 Hz, 1H, H_2B_), 7.78 (d, *J* = 15.5 Hz, 1H, H*_α_*), 7.69–7.64 (m, 2H, H*_β_* and H_6B_), 7.20 (s, 2H, H_2D_), 7.19–7.13 (m, 2H, H_3A_ and H_4B_), 6.89 (dd, *J* = 8.3, 4.6 Hz, 1H, H_5B_), 3.88 (s, 6H, 3D-OCH_3_), 3.85 (s, 3H, OCH_3_), 3.73 (s, 3H, 4D-OCH_3_). ^13^C NMR (101 MHz, DMSO-*d*_6_) δ ppm 187.5 (C), 160.3 (C), 153.1 (C), 146.8 (C), 143.9 (CH), 143.5 (CH), 139.7 (C), 137.0 (CH), 134.9 (C), 132.5 (CH), 130.3 (C), 129.8 (CH), 129.1 (C), 125.5 (CH), 122.8 (CH), 121.2 (CH), 111.9 (CH), 106.4 (CH), 60.2 (CH_3_), 56.2 (CH_3_), 56.2 (CH_3_). MS (EI, *m*/*z* (%)): 484 (M^+^, 13), 264 (7), 109 (35), 95 (50), 57 (47), 43 (100). Anal. calcd. for C_24_H_24_N_2_O_7_S: C, 59.49; H, 4.99; N, 5.78; S, 6.62. Found: C, 59.51; H, 4.80; N, 5.79; S, 6.58.

##### (*E*)-5-(3-(Benzo[d][1,3]dioxol-5-yl)acryloyl)-2-methoxy-*N*-(pyridin-3-yl)benzenesulfonamide (**16f**)

Yellow solid; 82% yield; m.p. 220–223 °C. FTIR (ATR) υ(cm^−1^): 3076 (C-H Ar), 1659 (C=O), 1596 (C=C), 1236 (C-O-C), 1159 (S=O). ^1^H NMR (400 MHz, DMSO-*d*_6_) δ ppm 8.46 (d, *J* = 2.4 Hz, 1H, H_6A_), 8.24 (dd, *J* = 8.6, 2.4 Hz, 1H, H_4A_), 8.00 (d, *J* = 2.7 Hz, 1H, H_2B_), 7.72–7.60 (m, 3H, H*_α_*, H*_β_* and H_6B_), 7.57 (s, 1H, H_2D_), 7.29 (d, *J* = 8.1 Hz, 1H, H_6D_), 7.18–7.09 (m, 2H, H_3A_ and H_4B_), 6.98 (d, *J* = 8.1 Hz, 1H, H_5D_), 6.88 (dd, *J* = 8.3, 4.6 Hz, 1H, H_5B_), 6.10 (s, 2H, CH_2_), 3.83 (s, 3H, OCH_3_). ^13^C NMR (101 MHz, DMSO-*d*_6_) δ ppm 187.2 (C), 160.2 (C), 149.5 (C), 148.1 (C), 146.8 (C), 143.6 (CH), 143.4 (CH), 136.9 (CH), 134.8 (C), 132.4 (CH), 129.7 (CH), 129.2 (C), 129.1 (C), 125.6 (CH), 125.4 (CH), 122.8 (CH), 119.9 (CH), 111.9 (CH), 108.5 (CH), 107.0 (CH), 101.6 (CH_2_), 56.2 (CH_3_). MS (EI, *m*/*z* (%)): 438 (M^+^, 55), 251 (42), 165 (56), 89 (86), 66 (57), 39 (100). Anal. calcd. for C_22_H_18_N_2_O_6_S: C, 60.27; H, 4.14; N, 6.39; S, 7.31. Found: C, 60.34; H, 4.10; N, 6.39; S, 7.50.

##### 5-Cinnamoyl-2,4-dimethoxybenzenesulfonamide (**17a**)

Beige solid; 60% yield; m.p. 230–231 °C. FTIR (ATR) υ(cm^−1^): 3403 and 3375 (N-H), 3097 (C-H Ar), 1638 (C=O), 1596 (C=C), 1153 (S=O). ^1^H NMR (400 MHz, DMSO-*d*_6_) δ ppm 8.04 (s, 1H, H_6A_), 7.77–7.71 (m, 2H, H_2D_), 7.63–7.52 (m, 2H, H*_α_* and H*_β_*), 7.48–7.42 (m, 3H, H_3D_ and H_4D_), 7.10 (s, 2H, NH_2_), 6.89 (s, 1H, H_3A_), 4.04 (s, 6H, 2A-OCH_3_ and 4A-OCH_3_). ^13^C NMR (101 MHz, DMSO-*d*_6_) δ ppm 188.6 (C), 162.9 (C), 160.5 (C), 142.1 (CH), 134.7 (C), 130.6 (CH), 130.4 (CH), 129.0 (CH), 128.5 (CH), 126.6 (CH), 124.1 (C), 119.5 (C), 97.2 (CH), 56.8 (CH_3_). MS (EI, *m*/*z* (%)): 347 (M^+^, 36), 319 (34), 256 (55), 131 (62), 103 (100), 77 (69). Anal. calcd. for C_17_H_17_NO_5_S: C, 58.78; H, 4.93; N, 4.03; S, 9.23. Found: C, 58.59; H, 4.90; N, 4.15; S, 9.22.

##### (*E*)-5-(3-(4-Chlorophenyl)acryloyl)-2,4-dimethoxybenzenesulfonamide (**17b**)

Pale yellow solid; 77% yield; m.p. 260–262 °C. FTIR (ATR) υ(cm^−1^): 3397 and 3238 (N-H), 3106 (C-H Ar), 1642 (C=O), 1597 (C=C), 1154 (S=O). ^1^H NMR (400 MHz, DMSO-*d*_6_) δ ppm 8.04 (s, 1H, H_6A_), 7.77 (d, *J* = 8.6 Hz, 2H, H_2D_), 7.54–7.59 (m, 2H, H*_α_* and H*_β_*), 7.50 (d, *J* = 8.6 Hz, 2H, H_3D_), 7.10 (s, 2H, NH_2_), 6.88 (s, 1H, H_3A_), 4.06–4.02 (m, 6H, 2A-OCH_3_ and 4A-OCH_3_). ^13^C NMR (101 MHz, DMSO-*d*_6_) δ ppm 188.5 (C), 163.0 (C), 160.7 (C), 140.6 (CH), 134.9 (C), 133.7 (C), 130.7 (CH), 130.2 (CH), 129.1 (CH), 127.3 (CH), 124.1 (C), 119.4 (C), 97.2 (CH), 56.85 (CH_3_), 56.83 (CH_3_). MS (EI, *m*/*z* (%)): 381/383 (M^+^/M+2^+^, 22/8), 353 (36), 256 (100), 137 (55), 102 (70), 75 (43). Anal. calcd. for C_17_H_16_ClNO_5_S: C, 53.47; H, 4.22; N, 3.67; S, 8.40. Found: C, 53.50; H, 4.30; N, 3.63; S, 8.32.

##### (*E*)-2,4-Dimethoxy-5-(3-(*p*-tolyl)acryloyl)benzenesulfonamide (**17c**)

Pale yellow crystals; 65% yield; m.p. 206–208 °C. FTIR (ATR) υ(cm^−1^): 3369 and 3256 (N-H), 2987 (C-H), 1654 (C=O), 1600 (C=C), 1152 (S=O). ^1^H NMR (400 MHz, DMSO-*d*_6_) δ ppm 8.04 (s, 1H, H_6A_), 7.65 (d, *J* = 7.7 Hz, 2H, H_2D_), 7.59 (d, *J* = 15.8 Hz, 1H, H*_β_*), 7.50 (d, *J* = 15.8 Hz, 1H, H*_α_*), 7.28 (d, *J* = 7.7 Hz, 2H, H_3D_), 7.11 (s, 2H, NH_2_), 6.90 (s, 1H, H_3A_), 4.08–4.03 (m, 6H, 2A-OCH_3_ and 4A-OCH_3_), 2.36 (s, 3H, CH_3_). ^13^C NMR (101 MHz, DMSO-*d*_6_) δ ppm 188.7 (C), 162.9 (C), 160.5 (C), 142.3 (CH), 140.6 (C), 132.0 (C), 130.6 (CH), 129.7 (CH), 128.6 (CH), 125.6 (CH), 124.0 (C), 119.6 (C), 97.2 (CH), 56.8 (CH_3_), 21.1 (CH_3_). MS (EI, *m*/*z* (%)): 361 (M^+^, 21), 346 (53), 281 (25), 115 (70), 105 (100), 91 (47). Anal. calcd. for C_18_H_19_NO_5_S: C, 59.82; H, 5.30; N, 3.88; S, 8.87. Found: C, 59.77; H, 5.30; N, 3.95; S, 8.79. Crystals suitable for single-crystal X-ray diffraction were obtained from ethanolic solution, and the crystal data for **17c** were deposited at CCDC with reference CCDC 2191611.

##### (*E*)-2,4-Dimethoxy-5-(3-(4-methoxyphenyl)acryloyl)benzenesulfonamide (**17d**)

Yellow crystals; 82% yield; m.p. 215–218 °C. FTIR (ATR) υ(cm^−1^): 3398 and 3199 (N-H), 1638 (C=O), 1592 (C=C), 1251 (C-O-C), 1152 (S=O). ^1^H NMR (400 MHz, DMSO-*d*_6_) δ ppm 8.01 (s, 1H, H_6A_), 7.70 (d, *J* = 8.4 Hz, 2H, H_2D_), 7.56 (d, *J* = 15.8 Hz, 1H, H*_β_*), 7.40 (d, *J* = 15.8 Hz, 1H, H*_α_*), 7.09 (s, 2H, NH_2_), 7.00 (d, *J* = 8.4 Hz, 2H, H_3D_), 6.88 (s, 1H, H_3A_), 4.05–4.01 (m, 6H, 2A-OCH_3_ and 4A-OCH_3_), 3.81 (s, 3H, 4D-OCH_3_). ^13^C NMR (101 MHz, DMSO-*d*_6_) δ ppm 188.6 (C), 162.7 (C), 161.2 (C), 160.3 (C), 142.3 (CH), 130.4 (CH), 130.3 (CH), 127.3 (C), 124.2 (CH), 124.0 (C), 119.8 (C), 114.5 (CH), 97.2 (CH), 56.71 (CH_3_), 56.70 (CH_3_), 55.3 (CH_3_). MS (EI, *m*/*z* (%)): 377 (M^+^, 48), 297 (44), 244 (27), 161 (52), 133 (47), 121 (100). Anal. calcd. for C_18_H_19_NO_6_S: C, 57.28; H, 5.07; N, 3.71; S, 8.50. Found: C, 57.09; H, 5.14; N, 3.65; S, 8.52. Crystals suitable for single-crystal X-ray diffraction were obtained from ethanolic solution, and the crystal data for **17d** were deposited at CCDC with reference CCDC 2191612.

##### (*E*)-2,4-Dimethoxy-5-(3-(3,4,5-trimethoxyphenyl)acryloyl)benzenesulfonamide (**17e**)

Beige solid; 62% yield; m.p. 253–256 °C. FTIR (ATR) υ(cm^−1^): 3381 and 3253 (N-H), 2972 (C-H Ar), 1653 (C=O), 1607 (C=C), 1270 (C-O-C), 1149 (S=O). ^1^H NMR (400 MHz, DMSO-*d*_6_) δ ppm 7.98 (s, 1H, H_6A_), 7.53–7.43 (m, 2H, H*_β_* and H*_α_*), 7.09 (s, 2H, NH_2_), 7.07 (s, 2H, H_2D_), 6.89 (s, 1H, H_3A_), 4.05–4.01 (m, 6H, 2A-OCH_3_ and 4A-OCH_3_), 3.84 (s, 6H, 3D-OCH_3_), 3.71 (s, 3H, 4D-OCH_3_). ^13^C NMR (101 MHz, DMSO-*d*_6_) δ ppm 189.2 (C), 162.7 (C), 160.3 (C), 153.1 (C), 142.8 (CH), 139.6 (C), 130.2(CH, C), 126.2 (CH), 124.0 (C), 119.8 (C), 106.0 (CH), 97.2 (CH), 60.1 (CH_3_), 56.74 (CH_3_), 56.69 (CH_3_), 56.0 (CH_3_). MS (EI, *m*/*z* (%)): 437 (M^+^, 100), 406 (40), 355 (24), 181 (43), 127 (35), 43 (29). Anal. calcd. for C_20_H_23_NO_8_S: C, 54.91; H, 5.30; N, 3.20; S, 7.33. Found: C, 55.00; H, 5.28; N, 3.35; S, 7.38.

##### (*E*)-5-(3-(Benzo[d][1,3]dioxol-5-yl)acryloyl)-2,4-dimethoxybenzenesulfonamide (**17f**)

Pale yellow solid; 53% yield; m.p. 257–258 °C. FTIR (ATR) υ(cm^−1^): 3389 and 3300 (N-H), 3109 (C-H Ar), 1646 (C=O), 1595 (C=C), 1244 (C-O-C), 1154 (S=O). ^1^H NMR (400 MHz, DMSO-*d*_6_) δ ppm 7.99 (s, 1H, H_6A_), 7.52 (d, *J* = 15.7 Hz, 1H, H*_β_*), 7.42–7.35 (m, 2H, H*_α_* and H_2D_), 7.24 (d, *J* = 8.0 Hz, 1H, H_6D_), 7.08 (s, 2H, NH_2_), 6.98 (d, *J* = 8.0 Hz, 1H, H_5D_), 6.87 (s, 1H, H_3A_), 6.09 (s, 2H, CH_2_), 4.06–4.00 (m, 6H, 2A-OCH_3_ and 4A-OCH_3_). ^13^C NMR (101 MHz, DMSO-*d*_6_) δ ppm 188.7 (C), 162.7 (C), 160.3 (C), 149.4 (C), 148.1 (C), 142.3 (CH), 130.4 (CH), 129.1 (C), 125.2 (CH), 124.7 (CH), 124.0 (C), 119.8 (C), 108.6 (CH), 106.8 (CH), 101.6 (CH_2_), 97.2 (CH), 56.7 (CH_3_). MS (EI, *m*/*z* (%)): 391 (M^+^, 100), 377 (22), 69 (27), 55 (31), 43 (67). Anal. calcd. for C_18_H_17_NO_7_S: C, 55.24; H, 4.38; N, 3.58; S, 8.19. Found: C, 55.09; H, 4.40; N, 3.63; S, 8.13.

#### 3.2.8. General Procedure for the Synthesis of Chalcone–Sulfonamide Hybrids **18a-f**

To a mixture of sulfonamide **12** (0.20 mmol) and the corresponding aldehyde **15a-f** (0.23 mmol) in ethanol (1.5 mL), 2 drops of 50% (*w/v*) NaOH solution were added and the reaction mixture was stirred at room temperature. The resulting precipitate was collected by filtration under vacuum, washed with ethanol and purified by column chromatography on silica gel, using a mixture of CHCl_3_:EtOAc (10:1) as eluent.

##### *N*,*N*-bis(2-chloroethyl)-5-cinnamoyl-2,4-dimethoxybenzenesulfonamide (**18a**)

Beige solid; 79% yield; m.p. 184–186 °C. FTIR (ATR) υ(cm^−1^): 3065 (C-H Ar), 2949 (C-H), 1645 (C=O), 1599 (C=C), 1145 (S=O). ^1^H NMR (400 MHz, CDCl_3_) δ ppm 8.29 (s, 1H, H_6A_), 7.66 (d, *J* = 15.8 Hz, 1H, H*_β_*), 7.59 (dd, *J* = 6.6, 3.0 Hz, 2H, H_2D_), 7.42–7.34 (m, 4H, H_3D_, H_4D_ and H*_α_*), 6.54 (s, 1H, H_3A_), 4.04 (s, 3H, 4A-OCH_3_), 4.00 (s, 3H, 2A-OCH_3_), 3.64 (s, 8H, N-CH_2_ and Cl-CH_2_). ^13^C NMR (101 MHz, CDCl_3_) δ ppm 189.5 (C), 163.8 (C), 160.8 (C), 143.9 (CH), 135.1 (C), 134.9 (CH), 130.6 (CH), 129.1 (CH), 128.6 (CH), 126.4 (CH), 121.6 (C), 120.1 (C), 95.9 (CH), 56.6 (CH_3_), 56.5 (CH_3_), 51.3 (CH_2_), 42.4 (CH_2_). MS (EI, *m*/*z* (%)): 472 (M^+^, 4), 331 (100), 283 (38), 131 (52), 103 (41). Anal. calcd. for C_21_H_23_Cl_2_NO_5_S: C, 53.40; H, 4.91; N, 2.97; S, 6.79. Found: C, 53.25; H, 4.90; N, 3.06; S, 6.68.

##### (*E*)-*N*,*N*-bis(2-Chloroethyl)-5-(3-(4-chlorophenyl)acryloyl)-2,4-dimethoxybenzenesulfonamide (**18b**)

White solid; 88% yield; m.p. 186–187 °C. FTIR (ATR) υ(cm^−1^): 3084 (C-H Ar), 2914 (C-H), 1645 (C=O), 1601 (C=C), 1149 (S=O). ^1^H NMR (400 MHz, CDCl_3_) δ ppm 8.30 (s, 1H, H_6A_), 7.60 (d, *J* = 15.8 Hz, 1H, H*_β_*), 7.51 (d, *J* = 8.4 Hz, 2H, H_2D_), 7.39–7.31 (m, 3H, H_3D_ and H*_α_*), 6.53 (s, 1H, H_3A_), 4.04 (s, 3H, 4A-OCH_3_), 4.00 (s, 3H, 2A-OCH_3_), 3.63 (s, 8H, N-CH_2_ and Cl-CH_2_). ^13^C NMR (101 MHz, CDCl_3_) δ ppm 189.0 (C), 163.9 (C), 161.0 (C), 142.2 (CH), 136.4 (C), 135.0 (CH), 133.6 (C), 129.7 (CH), 129.3 (CH), 126.8 (CH), 121.4 (C), 120.2 (C), 95.9 (CH), 56.6 (CH_3_), 56.5 (CH_3_), 51.3 (CH_2_), 42.4 (CH_2_). MS (EI, *m*/*z* (%)): 507 (M^+^, 4), 365 (100), 317 (26), 165 (30), 135 (24). Anal. calcd. for C_21_H_22_Cl_3_NO_5_S: C, 49.77; H, 4.38; N, 2.76; S, 6.33. Found: C, 49.68; H, 4.45; N, 2.79; S, 6.09. 

##### (*E*)-*N*,*N*-bis(2-Chloroethyl)-2,4-dimethoxy-5-(3-(*p*-tolyl)acryloyl)benzenesulfonamide (**18c**)

Pale yellow solid; 86% yield; m.p. 186–189 °C. FTIR (ATR) υ(cm^−1^): 2923 (C-H), 1644 (C=O), 1597 (C=C), 1276 (C-O-C), 1150 (S=O). ^1^H NMR (400 MHz, CDCl_3_) δ ppm 8.27 (s, 1H, H_6A_), 7.62 (d, *J* = 15.8 Hz, 1H, H*_β_*), 7.48 (d, *J* = 7.9 Hz, 2H, H_2D_), 7.30 (d, *J* = 15.8 Hz, 1H, H*_α_*), 7.20 (d, *J* = 7.9 Hz, 2H, H_3D_), 6.53 (s, 1H, H_3A_), 4.03 (s, 3H, 4A-OCH_3_), 3.99 (s, 3H, 2A-OCH_3_), 3.63 (s, 8H, N-CH_2_ and Cl-CH_2_), 2.38 (s, 3H, CH_3_). ^13^C NMR (101 MHz, CDCl_3_) δ ppm 189.6 (C), 163.7 (C), 160.7 (C), 144.1 (CH), 141.1 (C), 134.8 (CH), 132.3 (C), 129.8 (CH), 128.6 (CH), 125.4 (CH), 121.7 (C), 119.9 (C), 95.9 (CH), 56.6 (CH_3_), 56.5 (CH_3_), 51.3 (CH_2_), 42.4 (CH_2_), 21.6 (CH_3_). MS (EI, *m*/*z* (%)): 486 (M^+^, 4), 345 (100), 297 (28), 145 (39), 105 (56). Anal. calcd. for C_22_H_25_Cl_2_NO_5_S: C, 54.33; H, 5.18; N, 2.88; S, 6.59. Found: C, 54.52; H, 5.22; N, 2.57; S, 6.68.

##### (*E*)-*N*,*N*-bis(2-Chloroethyl)-2,4-dimethoxy-5-(3-(4-methoxyphenyl)acryloyl)benzenesulfonamide (**18d**)

Yellow solid; 84% yield; m.p. 168–171 °C. FTIR (ATR) υ(cm^−1^): 3016 (C-H Ar), 2847 (C-H), 1642 (C=O), 1593 (C=C), 1143 (S=O). ^1^H NMR (400 MHz, CDCl_3_) δ ppm 8.26 (s, 1H, H_6A_), 7.61 (d, *J* = 15.8 Hz, 1H, H*_β_*), 7.54 (d, *J* = 8.8 Hz, 2H, H_2D_), 7.22 (d, *J* = 15.8 Hz, 1H, H*_α_*), 6.91 (d, *J* = 8.7 Hz, 2H, H_3D_), 6.53 (s, 1H, H_3A_), 4.03 (s, 3H, 4A-OCH_3_), 3.99 (s, 3H, 2A-OCH_3_), 3.84 (s, 3H, 4D-OCH_3_), 3.63 (s, 8H, N-CH_2_ and Cl-CH_2_). ^13^C NMR (101 MHz, CDCl_3_) δ ppm 189.6 (C), 163.7 (C), 161.8 (C), 160.6 (C), 144.0 (CH), 134.7 (CH), 130.4 (CH), 127.7 (C), 124.2 (CH), 121.9 (C), 119.9 (C), 114.5 (CH), 95.9 (CH), 56.6 (CH_3_), 56.5 (CH_3_), 55.5 (CH_3_), 51.3 (CH_2_), 42.4 (CH_2_). MS (EI, *m*/*z* (%)): 502 (M^+^, 4), 361 (46), 267 (28), 161 (39), 121 (100). Anal. calcd. for C_22_H_25_Cl_2_NO_6_S: C, 52.60; H, 5.02; N, 2.79; S, 6.38. Found: C, 52.43; H, 5.03; N, 2.54; S, 6.35.

##### (*E*)-*N*,*N*-bis(2-Chloroethyl)-2,4-dimethoxy-5-(3-(3,4,5-trimethoxyphenyl)acryloyl)benzenesulfonamide (**18e**)

Yellow solid; 61% yield; m.p. 186–188 °C. FTIR (ATR) υ(cm^−1^): 2957 (C-H), 1647 (C=O), 1600 (C=C), 1273 (C-O-C), 1145 (S=O). ^1^H NMR (400 MHz, CDCl_3_) δ ppm 8.24 (s, 1H, H_6A_), 7.52 (d, *J* = 15.8 Hz, 1H, H*_β_*), 7.19 (d, *J* = 15.8 Hz, 1H, H*_α_*), 6.80 (s, 2H, H_2D_), 6.53 (s, 1H, H_3A_), 4.03 (s, 3H, 4A-OCH_3_), 3.98 (s, 3H, 2A-OCH_3_), 3.91–3.87 (m, 9H, 3D-OCH_3_ and 4D-OCH_3_), 3.63 (s, 8H, N-CH_2_ and Cl-CH_2_). ^13^C NMR (101 MHz, CDCl_3_) δ 189.7 (C), 163.7 (C), 160.7 (C), 153.6 (C), 144.3 (CH), 140.6 (C), 134.5 (CH), 130.5 (C), 125.8 (CH), 121.7 (C), 119.9 (C), 105.9 (CH), 96.0 (CH), 61.1 (CH_3_), 56.6 (CH_3_), 56.5 (CH_3_), 56.4 (CH_3_), 51.3 (CH_2_), 42.4 (CH_2_). MS (EI, *m*/*z* (%)): 562 (M^+^, 2), 334 (45), 243 (100), 83 (79), 43 (51). Anal. calcd. for C_24_H_29_Cl_2_NO_8_S: C, 51.25; H, 5.20; N, 2.49; S, 5.70. Found: C, 51.05; H, 5.43; N, 2.52; S, 5.91.

##### (*E*)-5-(3-(Benzo[d][1,3]dioxol-5-yl)acryloyl)-*N*,*N*-bis(2-chloroethyl)-2,4-dimethoxybenzenesulfonamide (**18f**)

Yellow solid; 82% yield; m.p. 193–196 °C. FTIR (ATR) υ(cm^−1^): 2885 (C-H), 1643 (C=O), 1595 (C=C), 1250 (C-O-C), 1150 (S=O). ^1^H NMR (400 MHz, CDCl_3_) δ 8.27 (s, 1H, H_6A_), 7.57 (d, *J* = 15.7 Hz, 1H, H*_β_*), 7.18 (d, *J* = 15.7 Hz, 1H, H*_α_*), 7.13–7.02 (m, 2H H_2D_ and H_6D_), 6.82 (d, *J* = 7.9 Hz, 1H, H_5D_), 6.53 (s, 1H, H_3A_), 6.02 (s, 2H, O-CH_2_), 4.06–3.97 (m, 6H, 2A-OCH_3_ and 4A-OCH_3_), 3.63 (s, 8H, N-CH_2_ and Cl-CH_2_). ^13^C NMR (101 MHz, CDCl_3_) δ ppm 189.3 (C), 163.7 (C), 160.7 (C), 150.0 (C), 148.5 (C), 143.9 (CH), 134.8 (CH), 129.5 (C), 125.4 (CH), 124.5 (CH), 121.8 (C), 120.0 (C), 108.8 (CH), 106.8 (CH), 101.7 (CH_2_), 95.9 (CH), 56.6 (CH_3_), 56.5 (CH_3_), 51.3 (CH_2_), 42.4 (CH_2_). MS (EI, *m*/*z* (%)): 516 (M^+^, 7), 375 (49), 311 (47), 281 (36), 135 (100). Anal. calcd. for C_22_H_23_Cl_2_NO_7_S: C, 51.17; H, 4.49; N, 2.71; S, 6.21. Found: C, 51.10; H, 4.58; N, 2.82; S, 6.22.

#### 3.2.9. General Procedure for the Synthesis of Chalcone–Sulfonamide Hybrids **19a-f**

To a mixture of sulfonamide **14a** (0.20 mmol) and the corresponding aldehyde **15a-f** (0.24 mmol) in ethanol (1.5 mL), 2 drops of a 50% *w/v* NaOH solution were added and stirred at room temperature. After completion of the reaction, the medium was neutralized using an aqueous 10% *v/v* HCl solution, the precipitate was filtered and washed with water. The products **19a-f** were purified by CC on silica gel, using a CHCl_3_:EtOH (15:1) mixture as eluent.

##### 5-Cinnamoyl-*N*’-isonicotinoyl-2-methoxybenzenesulfonohydrazide (**19a**)

Beige solid; 78% yield; m.p. 227–229 °C. FTIR (ATR) υ(cm^−1^): 3212 (N-H), 3013 (C-H Ar), 1682 (C=O), 1654 (C=O), 1597 (C=C), 1155 (S=O). ^1^H NMR (400 MHz, DMSO-*d*_6_) δ 10.97 (s, 1H, S-NH), 9.95 (s, 1H, C-NH), 8.67 (d, *J* = 6.0 Hz, 2H, H_2C_), 8.50 (dd, *J* = 8.8, 2.3 Hz, 1H, H_4A_), 8.43 (d, *J* = 2.3 Hz, 1H, H_6A_), 7.90 (d, *J* = 15.6 Hz, 1H, H*_α_*), 7.88–7.83 (m, 2H, H_2D_), 7.72 (d, *J* = 15.6 Hz, 1H, H*_β_*), 7.58 (d, *J* = 6.0 Hz, 2H, H_3C_), 7.48–7.43 (m, 3H, H_3D_ and H_4D_), 7.38 (d, *J* = 8.8 Hz, 1H, H_3A_), 4.04 (s, 3H, OCH_3_). ^13^C NMR (101 MHz, DMSO-*d*_6_) δ ppm 186.8 (C), 164.2 (C), 161.1 (C), 150.4 (CH), 144.1 (CH), 138.9 (C), 135.9 (CH), 134.6 (C), 130.7 (CH), 130.2 (CH), 129.1 (C), 128.9 (CH), 128.9 (CH), 127.7 (C), 121.5 (CH), 121.2 (CH), 113.0 (CH), 57.1 (CH_3_). MS (EI, *m*/*z* (%)): 437 (M^+^, 1), 238 (61), 135 (88), 106 (100), 78 (77), 51 (63). Anal. calcd. for C_22_H_19_N_3_O_5_S: C, 60.40; H, 4.38; N, 9.61; S, 7.33. Found: C, 60.36; H, 4.30; N, 9.55; S, 7.45.

##### (*E*)-5-(3-(4-Chlorophenyl)acryloyl)-*N*’-isonicotinoyl-2-methoxybenzenesulfonohydrazide (**19b**)

White solid; 75%; m.p. 242–244 °C. FTIR (ATR) υ(cm^−1^): 3235 (N-H), 3030 (C-H Ar), 1681 (C=O), 1655 (C=O), 1600 (C=C), 1155 (S=O). ^1^H NMR (400 MHz, DMSO-*d*_6_) δ ppm 10.99 (s, 1H, S-NH), 9.99 (s, 1H, C-NH), 8.67 (d, *J* = 5.3 Hz, 2H, H_2C_), 8.52 (d, *J* = 8.2 Hz, 1H, H_4A_), 8.42 (s, 1H, H_6A_), 7.99–7.89 (m, 3H, H_2D_ and H*_α_*), 7.71 (d, *J* = 15.5 Hz, 1H, H*_β_*), 7.57 (d, *J* = 5.3 Hz, 2H, H_3C_), 7.52 (d, *J* = 8.1 Hz, 2H, H_3D_), 7.38 (d, *J* = 8.2 Hz, 1H, H_3A_), 4.03 (s, 3H, OCH_3_). ^13^C NMR (101 MHz, DMSO-*d*_6_) δ ppm 186.6 (C), 164.2 (C), 161.2 (C), 150.4 (CH), 142.7 (CH), 138.9 (C), 136.0 (CH), 135.2 (C), 133.6 (C), 130.7 (CH), 130.3 (CH), 129.02 (C), 128.98 (CH), 127.8 (C), 122.2 (CH), 121.2 (CH), 113.1 (CH), 57.1 (CH_3_). MS (EI, *m*/*z* (%)): 471/473 (M^+^/M+2^+^, 5/2), 272 (42), 106 (100), 78 (98), 51 (88). Anal. calcd. for C_22_H_18_ClN_3_O_5_S: C, 55.99; H, 3.84; N, 8.90; S, 6.79. Found: C, 56.08; H, 3.70; N, 8.91; S, 6.75.

##### (*E*)-*N*’-Isonicotinoyl-2-methoxy-5-(3-(*p*-tolyl)acryloyl)benzenesulfonohydrazide (**19c**)

White solid; 81% yield; m.p. 250-252 °C. FTIR (ATR) υ(cm^-1^): 3508 (N-H), 3033 (C-H Ar), 1691 (C=O), 1658 (C=O), 1599 (C=C), 1162 (S=O). ^1^H NMR (400 MHz, DMSO-*d*_6_) δ ppm 10.97 (s, 1H, S-NH), 9.94 (s, 1H, C-NH), 8.67 (d, *J* = 5.0 Hz, 2H, H_2C_), 8.49 (d, *J* = 8.7 Hz, 1H, H_4A_), 8.42 (s, 1H, H_6A_), 7.84 (d, *J* = 15.5 Hz, 1H, H*_α_*), 7.75 (d, *J* = 7.8 Hz, 2H, H_2D_), 7.69 (d, *J* = 15.5 Hz, 1H, H*_β_*), 7.58 (d, *J* = 5.0 Hz, 2H, H_3C_), 7.37 (d, *J* = 8.7 Hz, 1H, H_3A_), 7.26 (d, *J* = 7.8 Hz, 2H, H_3D_), 4.03 (s, 3H, OCH_3_), 2.34 (s, 3H, CH_3_). ^13^C NMR (101 MHz, DMSO-*d*_6_) δ ppm 186.7 (C), 164.2 (C), 161.0 (C), 150.4 (CH), 144.2 (CH), 140.8 (C), 138.9 (C), 135.8 (CH), 131.9 (C), 130.2 (CH), 129.5 (CH), 129.2 (C), 129.0 (CH), 127.7 (C), 121.2 (CH), 120.4 (CH), 113.0 (CH), 57.1 (CH_3_), 21.1(CH_3_). MS (EI, *m*/*z* (%)): 451 (M^+^, 3), 252 (61), 135 (69), 106 (100), 78 (77), 51 (58). Anal. calcd. for C_23_H_21_N_3_O_5_S: C, 61.18; H, 4.69; N, 9.31; S, 7.10. Found: C, 61.01; H, 4.76; N, 9.47; S, 7.11.

##### (*E*)-*N*’-Isonicotinoyl-2-methoxy-5-(3-(4-methoxyphenyl)acryloyl)benzenesulfonohydrazide (**19d**)

Pale yellow solid; 84% yield; m.p. 216–217 °C. FTIR (ATR) υ(cm^−1^): 3233 (N-H), 3042 (C-H Ar), 1676 (C=O), 1655 (C=O), 1597 (C=C), 1256 (C-O-C), 1154 (S=O). ^1^H NMR (400 MHz, DMSO-*d*_6_) δ ppm 10.97 (s, 1H, S-NH), 9.93 (s, 1H, C-NH), 8.67 (d, *J* = 5.9 Hz, 2H, H_2C_), 8.48 (dd, *J* = 8.8, 2.3 Hz, 1H, H_4A_), 8.41 (d, *J* = 2.3 Hz, 1H, H_6A_), 7.83 (d, *J* = 8.6 Hz, 2H, H_2D_), 7.76 (d, *J* = 15.5 Hz, 1H, H*_α_*), 7.69 (d, *J* = 15.5 Hz, 1H, H*_β_*), 7.58 (d, *J* = 5.9 Hz, 2H, H_3C_), 7.36 (d, *J* = 8.8 Hz, 1H, H_3A_), 7.01 (d, *J* = 8.6 Hz, 2H, H_3D_), 4.03 (s, 3H, OCH_3_), 3.82 (s, 3H, 4D-OCH_3_). ^13^C NMR (101 MHz, DMSO-*d*_6_) δ ppm 186.6 (C), 164.2 (C), 161.4 (C), 160.9 (C), 150.3 (CH), 144.2 (CH), 138.9 (C), 135.7 (CH), 130.8 (CH), 130.1 (CH), 129.4 (C), 127.7 (C), 127.2 (C), 121.2 (CH), 118.9 (CH), 114.4 (CH), 113.0 (CH), 57.0 (CH_3_), 55.4 (CH_3_). MS (EI, *m*/*z* (%)): 467 (M^+^, 3), 300 (32), 268 (82), 78 (74), 51 (100). Anal. calcd. for C_23_H_21_N_3_O_6_S: C, 59.09; H, 4.53; N, 8.99; S, 6.86. Found: C, 59.20; H, 4.53; N, 9.06; S, 6.72.

##### (*E*)-*N*’-Isonicotinoyl-2-methoxy-5-(3-(3,4,5-trimethoxyphenyl)acryloyl)benzenesulfonohydrazide (**19e**)

Yellow solid; 70% yield; m.p. 202–204 °C. FTIR (ATR) υ(cm^−1^): 3260 (N-H), 3049 (C-H Ar), 1678 (C=O), 1636 (C=O), 1595 (C=C), 1279 (C-O-C), 1123 (S=O). ^1^H NMR (400 MHz, DMSO-*d*_6_) δ ppm 10.98 (d, *J* = 2.8 Hz, 1H, S-NH), 9.95 (d, *J* = 2.8 Hz, 1H, C-NH), 8.68 (d, *J* = 6.1 Hz, 2H, H_2C_), 8.51 (dd, *J* = 8.8, 2.3 Hz, 1H, H_4A_), 8.42 (d, *J* = 2.3 Hz, 1H, H_6A_), 7.86 (d, *J* = 15.5 Hz, 1H, H*_α_*), 7.68 (d, *J* = 15.5 Hz, 1H, H*_β_*), 7.58 (d, *J* = 6.1 Hz, 2H, H_3C_), 7.38 (d, *J* = 8.8 Hz, 1H. H_3A_), 7.22 (s, 2H, H_2D_), 4.04 (s, 3H, OCH_3_), 3.85 (s, 6H, 3D-OCH_3_), 3.72 (s, 3H, 4D-OCH_3_). ^13^C NMR (101 MHz, DMSO-*d*_6_) δ ppm 186.7 (C), 164.2 (C), 161.0 (C), 153.1 (C), 150.4 (CH), 144.6 (CH), 139.9 (C), 138.9 (C), 135.9 (CH), 130.19 (C), 130.16 (CH), 129.3 (C), 127.8 (C), 121.2 (CH), 120.8 (CH), 112.9 (CH), 106.7 (CH), 60.2 (CH_3_), 57.1 (CH_3_), 56.2 (CH_3_). MS (EI, *m*/*z* (%)): 527 (M^+^, 0.07), 328 (25), 297 (14), 135 (100), 106 (50), 78 (72), 51 (96). Anal. calcd. for C_25_H_25_N_3_O_8_S: C, 56.92; H, 4.78; N, 7.97; S, 6.08. Found: C, 57.00; H, 4.71; N, 7.80; S, 6.09.

##### (*E*)-5-(3-(Benzo[d][1,3]dioxol-5-yl)acryloyl)-*N*’-isonicotinoyl-2-methoxybenzenesulfonohydrazide (**19f**)

Yellow solid; 75% yielf; m.p. 245–248 °C. FTIR (ATR) υ(cm^−1^): 3230 (N-H), 3041 (C-H Ar), 1689 (C=O), 1648 (C=O), 1596 (C=C), 1220 (C-O-C), 1153 (S=O). ^1^H NMR (400 MHz, DMSO-*d*_6_) δ ppm 10.97 (s, 1H, S-NH), 9.93 (s, 1H, C-NH), 8.67 (d, *J* = 6.2 Hz, 2H,H_2C_), 8.49 (dd, *J* = 8.8, 2.3 Hz, 1H, H_4A_), 8.42 (d, *J* = 2.3 Hz, 1H, H_6A_), 7.79 (d, *J* = 15.4 Hz, 1H, H*_α_*), 7.69–7.61 (m, 2H, H*_β_* and H_2D_), 7.58 (d, *J* = 6.2 Hz, 2H, H_3C_), 7.36 (d, *J* = 8.8 Hz, 1H, H_3A_), 7.30 (dd, *J* = 8.2, 1.7 Hz, 1H, H_6D_), 6.98 (d, *J* = 8.2 Hz, 1H, H_5D_), 6.10 (s, 2H, CH_2_), 4.03 (s, 3H, OCH_3_). ^13^C NMR (101 MHz, DMSO-*d*_6_) δ ppm 186.5 (C), 164.2 (C), 161.0 (C), 150.4 (CH), 149.6 (C), 148.1 (C), 144.2 (CH), 138.9, 135.8 (CH), 130.1 (CH), 129.3 (C), 129.2 (C), 127.7 (C), 126.1 (CH), 121.2 (CH), 119.4 (CH), 113.0 (CH), 108.5 (CH), 107.0 (CH), 101.7 (CH_2_), 57.1 (CH_3_). MS (EI, *m*/*z* (%)): 481 (0.09), 282 (99.8), 135 (99.9), 106 (99.9), 78 (99.9), 51 (100). Anal. calcd. for C_23_H_19_N_3_O_7_S: C, 57.37; H, 3.98; N, 8.73; S, 6.66. Found: C, 57.32; H, 4.01; N, 8.59; S, 6.79.

#### 3.2.10. General Procedure for the Synthesis of Chalcone–Sulfonamide Hybrids **20a-f**

To a mixture of sulfonamide **14b** (0.20 mmol) and the respective aldehyde **15a-f** (0.24 mmol) in ethanol (1.5 mL), 2 drops of a 50% *w/v* NaOH solution were added and stirred at room temperature. The reaction media was neutralized with an aqueous 10% *v/v* HCl, the precipitate was filtered and washed with water. The products **20a-f** were purified by CC on silica gel and a DCM:MeOH (20:1) mixture as mobile phase.

##### 5-Cinnamoyl-*N*’-isonicotinoyl-2,4-dimethoxybenzenesulfonohydrazide (**20a**)

Beige crystals; 71% yield; m.p. 143–145 °C. FTIR (ATR) υ(cm^−1^): 3135 (N-H), 2988 (C-H Ar), 1673 (C=O), 1650 (C=O), 1597 (C=C), 1157 (S=O). ^1^H NMR (400 MHz, DMSO-*d*_6_) δ ppm 10.93 (d, *J* = 3.0 Hz, 1H, S-NH), 9.73 (d, *J* = 3.0 Hz, 1H, C-NH), 8.67 (d, *J* = 5.5 Hz, 2H, H_2C_), 7.99 (s, 1H, H_6A_), 7.70–7.65 (m, 2H, H_3D_), 7.58 (d, *J* = 5.5 Hz, 2H, H_3C_), 7.53 (d, *J* = 15.9 Hz, 1H, H*_β_*), 7.48 (d, *J* = 15.9 Hz, 1H, H*_α_*), 7.45–7.40 (m, 3H, H_3D_ and H_4D_), 6.88 (s, 1H, H_3A_), 4.06–4.02 (m, 6H, 2A-OCH_3_ and 4A-OCH_3_). ^13^C NMR (101 MHz, DMSO-*d*_6_) δ ppm 188.5 (C), 164.1 (C), 163.9 (C), 162.1 (C), 150.3 (CH), 142.3 (CH), 139.0 (C), 134.6 (C), 132.8 (CH), 130.5 (CH), 129.0 (CH), 128.4 (CH), 126.5 (CH), 121.2 (CH), 119.5 (C), 119.2 (C), 97.4 (CH), 57.1 (CH_3_), 56.8 (CH_3_). MS (EI, *m*/*z* (%)): 467 (M^+^, 0.3), 300 (18), 165 (52), 103 (79), 77 (94), 51 (100). Anal. calcd. for C_23_H_21_N_3_O_6_S: C, 59.09; H, 4.53; N, 8.99; S, 6.86. Found: C, 59.01; H, 4.55; N, 9.07; S, 6.70.

##### (*E*)-5-(3-(4-Chlorophenyl)acryloyl)-*N*’-isonicotinoyl-2,4-dimethoxybenzenesulfonohydrazide (**20b**)

Pale yellow solid; 47% yield; m.p. 196–198 °C. FTIR (ATR) υ(cm^−1^): 3183 (N-H), 2987 (C-H Ar), 1678 (C=O), 1650 (C=O), 1601 (C=C), 1157 (S=O). ^1^H NMR (400 MHz, DMSO-*d*_6_) δ ppm 10.93 (s, 1H, S-NH), 9.73 (s, 1H, C-NH), 8.67 (d, *J* = 5.8 Hz, 2H, H_2C_), 7.99 (s, 1H, H_6A_), 7.72 (d, *J* = 8.4 Hz, 2H, H_2D_), 7.58 (d, *J* = 5.8 Hz, 2H, H_3C_), 7.52–7.46 (m, 4H, H*_α_*, H*_β_* and H_3D_), 6.87 (s, 1H, H_3A_), 4.06–4.01 (m, 6H, 2A-OCH_3_ and 4A-OCH_3_). ^13^C NMR (101 MHz, DMSO-*d*_6_) δ ppm 188.3 (C), 164.1 (C), 163.9 (C), 162.2 (C), 150.3 (CH), 140.7 (CH), 139.0 (C), 134.9 (C), 133.6 (C), 132.9 (CH), 130.1 (CH), 129.0 (CH), 127.2 (CH), 121.2 (CH), 119.4 (C), 119.3 (C), 97.3 (CH), 57.1 (CH_3_), 56.8 (CH_3_). MS (EI, *m*/*z* (%)): 501/504 (M^+^/M+2^+^, 0.16/0.10), 274 (29), 165 (95), 106 (100), 78 (99.7), 51 (99.1). Anal. calcd. for

C_23_H_20_ClN_3_O_6_S: C, 55.04; H, 4.02; N, 8.37; S, 6.39. Found: C, 54.99; H, 4.07; N, 8.39; S, 6.52.

##### (*E*)-*N*’-Isonicotinoyl-2,4-dimethoxy-5-(3-(*p*-tolyl)acryloyl)benzenesulfonohydrazide (**20c**)

White solid; 46% yield; m.p. 195–197 °C. FTIR (ATR) υ(cm^−1^): 3161 (N-H), 2987 (C-H Ar), 1677 (C=O), 1650 (C=O), 1598 (C=C), 1157 (S=O). ^1^H NMR (400 MHz, DMSO-*d*_6_) δ ppm 10.93 (s, 1H, S-NH), 9.72 (s, 1H, C-NH), 8.67 (d, *J* = 6.1 Hz, 2H, H_2C_), 7.97 (s, 1H, H_6A_), 7.60–7.54 (m, 4H, H_3C_ and H_2D_), 7.49 (d, *J* = 15.8 Hz, 1H, H*_β_*), 7.41 (d, *J* = 15.8 Hz, 1H, H*_α_*), 7.23 (d, *J* = 7.7 Hz, 2H, H_3D_), 6.87 (s, 1H, H_3A_), 4.05–4.01 (m, 6H, 2A-OCH_3_ and 4A-OCH_3_), 2.33 (s, 3H, CH_3_). ^13^C NMR (101 MHz, DMSO-*d*_6_) δ ppm 188.6 (C), 164.1 (C), 163.8 (C), 162.0 (C), 150.3 (CH), 142.5 (CH), 140.5 (C), 139.0 (C), 132.8 (CH), 131.9 (C), 129.6 (CH), 128.5 (CH), 125.6 (CH), 121.2 (CH), 119.6 (C), 119.2 (C), 97.3 (CH), 57.1 (CH_3_), 56.8 (CH_3_), 21.0 (CH_3_). MS (EI, *m*/*z* (%)): 481 (M^+^, 0.6), 282 (25), 123 (66), 106 (100), 78 (97), 51 (86). Anal. calcd. for C_24_H_23_N_3_O_6_S: C, 59.86; H, 4.81; N, 8.73; S, 6.66. Found: C, 59.81; H, 4.72; N, 8.79; S, 6.74.

##### (*E*)-*N*’-Isonicotinoyl-2,4-dimethoxy-5-(3-(4-methoxyphenyl)acryloyl)benzenesulfonohydrazide (**20d**)

Pale yellow solid; 50% yield; m.p. 214–217 °C. FTIR (ATR) υ(cm^−1^): 3327 (N-H), 3231 (N-H), 2986 (C-H Ar), 1699 (C=O), 1651 (C=O), 1593 (C=C), 1223 (C-O-C), 1150 (S=O). ^1^H NMR (400 MHz, DMSO-*d*_6_) δ ppm 10.93 (s, 1H, S-NH), 9.71 (s, 1H, C-NH), 8.68 (d, *J* = 5.5 Hz, 2H, H_2C_), 7.96 (s, 1H, H_6A_), 7.63 (d, *J* = 8.3 Hz, 2H, H_2D_), 7.58 (d, *J* = 5.5 Hz, 2H, H_3C_), 7.48 (d, *J* = 15.8 Hz, 1H, H*_β_*), 7.33 (d, *J* = 15.8 Hz, 1H, H*_α_*), 6.98 (d, *J* = 8.3 Hz, 2H, H_3D_), 6.87 (s, 1H, H_3A_), 4.05–4.00 (m, 6H, 2A-OCH_3_ and 4A-OCH_3_), 3.80 (s, 3H, 4D-OCH_3_). ^13^C NMR (101 MHz, DMSO-*d*_6_) δ ppm 188.5 (C), 164.1 (C), 163.7 (C), 161.9 (C), 161.2 (C), 150.3 (CH), 142.5 (CH), 139.0 (C), 132.7 (CH), 130.3 (CH), 127.2 (C), 124.2 (CH), 121.2 (CH), 119.8 (C), 119.1 (C), 114.5 (CH), 97.3 (CH), 57.0 (CH_3_), 56.7 (CH_3_), 55.3 (CH_3_). MS (EI, *m*/*z* (%)): 497 (M^+^, 3), 282 (96), 139 (100), 111 (50), 75 (13). Anal. calcd. for C_24_H_23_N_3_O_7_S: C, 57.94; H, 4.66; N, 8.45; S, 6.45. Found: C, 57.85; H, 4.60; N, 8.55; S, 6.32.

##### (*E*)-*N*’-Isonicotinoyl-2,4-dimethoxy-5-(3-(3,4,5-trimethoxyphenyl)acryloyl)benzenesulfonohydrazide (**20e**)

Yellow solid; 42% yield; m.p. 162–165 °C. FTIR (ATR) υ(cm^−1^): 3222 (N-H), 1700 (C=O), 3002 (C-H Ar) 1678 (C=O), 1597 (C=C), 1276 (C-O-C), 1150 (S=O). ^1^H NMR (400 MHz, DMSO-*d*_6_) δ ppm 10.94 (s, 1H, S-NH), 9.72 (s, 1H, C-NH), 8.67 (d, *J* = 4.2 Hz, 2H, H_2C_), 7.96 (s, 1H, H_6A_), 7.58 (d, *J* = 5.0 Hz, 2H, H_3C_), 7.52–7.38 (m, 2H, H*_α_* and H*_β_*), 7.04 (s, 2H, H_2D_), 6.88 (s, 1H, H_3A_), 4.02 (s, 6H, 2A-OCH_3_ and 4A-OCH_3_), 3.82 (s, 6H, 3D-OCH_3_), 3.70 (s, 3H, 4D-OCH_3_). ^13^C NMR (101 MHz, DMSO-*d*_6_) δ ppm 189.2 (C), 164.6 (C), 164.3 (C), 162.5(C), 153.6 (C), 150.8 (CH), 143.2 (CH), 140.1 (C), 139.5 (C), 133.1 (CH), 130.7 (C), 126.6 (CH), 121.7 (CH), 120.3 (C), 119.7 (C), 106.5 (CH), 97.8 (CH), 60.6 (CH_3_), 57.6 (CH_3_), 57.2 (CH_3_), 56.5 (CH_3_). MS (EI, *m*/*z* (%)): 557 (M^+^, 0.4), 358 (78), 165 (62), 106 (100), 78 (82), 51 (75). Anal. calcd. for C_26_H_27_N_3_O_9_S: C, 56.01; H, 4.88; N, 7.54; S, 5.75. Found: C, 55.97; H, 4.82; N, 7.64; S, 5.73.

##### (*E*)-5-(3-(Benzo[d][1,3]dioxol-5-yl)acryloyl)-*N*’-isonicotinoyl-2,4-dimethoxybenzenesulfonohydrazide (**20f**)

Yellow crystals; 46% yield; m.p. 150–152 °C. FTIR (ATR) υ(cm^−1^): 3227 (N-H), 2988 (C-H Ar), 1649 (C=O), 1593 (C=C), 1242 (C-O-C), 1154 (S=O). ^1^H NMR (400 MHz, DMSO-*d*_6_) δ ppm 10.92 (d, *J* = 3.4 Hz, 1H, S-NH), 9.68 (d, *J* = 3.4 Hz, 1H, C-NH), 8.67 (d, *J* = 5.3 Hz, 2H, H_2C_), 7.95 (s, 1H, H_6A_), 7.58 (d, *J* = 5.3 Hz, 2H, H_3C_), 7.44 (d, *J* = 16.5 Hz, 1H, H*_β_*), 7.36–7.29 (m, 2H, H*_α_* and H_2D_), 7.15 (d, *J* = 8.0 Hz, 1H, H_6D_), 6.94 (d, *J* = 8.0 Hz, 1H, H_5D_), 6.86 (s, 1H, H_3A_), 6.09 (s, 2H, CH_2_), 4.06–3.99 (m, 6H, 2A-OCH_3_ and 4A-OCH_3_). ^13^C NMR (101 MHz, DMSO-*d*_6_) δ ppm 188.5 (C), 164.1 (C), 163.7 (C), 161.9 (C), 150.3 (CH),149.4 (C), 148.0 (C), 142.5 (CH), 139.0 (C), 132.6 (CH), 129.0 (C), 125.1 (CH), 124.7 (CH), 121.2 (CH), 119.8 (C), 119.1 (C), 108.5 (CH), 106.7 (CH), 101.6 (CH_2_), 97.3 (CH), 57.0 (CH_3_), 56.7 (CH_3_). MS (EI, *m*/*z* (%)): 511 (M^+^, 0.2), 312 (43), 135 (100), 106 (82), 78 (70), 51 (70). Anal. calcd. for C_24_H_21_N_3_O_8_S: C, 56.35; H, 4.14; N, 8.22; S, 6.27. Found: C, 56.43; H, 4.19; N, 8.20; S, 6.14. Crystals suitable for single-crystal X-ray diffraction were obtained from ethanolic solution, and the crystal data for **20f** were deposited at CCDC with reference CCDC 2191613.

#### 3.2.11. General Procedure for the Synthesis of Pyrazoline–Sulfonamide Hybrids **22a-d**

A mixture of the respective chalcone **(16-18)b** or **21b** (0.27 mmol) and hydrazine monohydrate (5.3 mmol) in ethanol (1.0 mL) was heated at reflux for 0.67 h. The reaction mixture was cooled, then 1.0 mL of formic acid was slowly added and stirred overnight. The solid formed was filtered and washed with small amounts of water and ethanol to afford compounds **22a-d** without further purification.

##### 5-(5-(4-Chlorophenyl)-1-formyl-4,5-dihydro-1*H*-pyrazol-3-yl)-2-methoxybenzenesulfonamide (**22a**)

White solid; 74% yield; m.p. 230–231 °C. FTIR (ATR) υ(cm^−1^): 3332 and 3230 (N-H), 3109 (C-H Ar), 2849 (-(C=O)-H), 1653 (C=O), 1600 (C=N), 1148 (S=O). ^1^H NMR (400 MHz, DMSO-*d*_6_) δ ppm 8.88 (s, 1H, CHO), 8.19 (d, *J* = 2.3 Hz, 1H, H_6A_), 7.87 (dd, *J* = 8.7, 2.3 Hz, 1H, H_4A_), 7.39 (d, *J* = 8.3 Hz, 2H, H_2D_), 7.32–7.17 (m, 5H, NH_2_, H_3A_ and H_3D_), 5.53 (dd, *J* = 11.6, 4.8 Hz, 1H, H_X_), 3.98–3.87 (m, 4H, H_M_ and OCH_3_), 3.17 (dd, *J* = 18.1, 4.8 Hz, 1H, H_A_). ^13^C NMR (101 MHz, DMSO-*d*_6_) δ ppm 160.0 (CHO), 157.8 (C), 155.4 (C), 140.3 (C), 132.8 (CH), 132.3 (C), 131.9 (C), 128.9 (CH), 128.0 (CH), 125.9 (CH), 122.7 (C), 113.3 (CH), 58.3 (CH), 56.7 (CH_3_), 42.3 (CH_2_). MS (EI, *m/z* (%)): 393/395 (M^+^/M+2^+^, 94/38), 254 (48), 228 (66), 213 (75), 57 (100), 43 (87). Anal. calcd. for C_17_H_16_ClN_3_O_4_S: C, 51.84; H, 4.09; N, 10.67; S, 8.14. Found: C, 51.99; H, 4.00; N, 10.75; S, 8.12.

##### 5-(5-(4-Chlorophenyl)-1-formyl-4,5-dihydro-1*H*-pyrazol-3-yl)-2-methoxy-*N*-(pyridin-3-yl)benzenesulfonamide (**22b**)

White solid; 81% yield; m.p. 241–243 °C. FTIR (ATR) υ(cm^−1^): 3192 (N-H), 3076 (C-H Ar), 1668 (C=O), 1604 (C=N), 1595 (C=C), 1159 (S=O). ^1^H NMR (400 MHz, DMSO-*d*_6_) δ ppm 10.39 (s, 1H, NH), 8.90 (s, 1H, CHO), 8.32 (d, *J* = 2.7 Hz, 1H, H_2B_), 8.25–8.18 (m, 2H, H_6A_ and H_6B_), 7.87 (dd, *J* = 8.7, 2.3 Hz, 1H, H_4A_), 7.51 (dt, *J* = 8.5, 2.7 Hz, 1H, H_4B_), 7.39 (d, *J* = 8.2 Hz, 2H, H_3D_), 7.30–7.24 (m, 4H, H_3A_, H_5B_ and H_2D_), 5.52 (dd, *J* = 11.9, 5.2 Hz, 1H, H_X_), 3.92 (s, 3H, OCH_3_), 3.87 (dd, *J* = 18.1, 11.9 Hz, 1H, H_M_), 3.21 (dd, *J* = 18.1, 5.2 Hz, 1H, H_A_). ^13^C NMR (101 MHz, DMSO-*d*_6_) δ ppm 159.8 (CHO), 157.6 (C), 154.7 (C), 145.2 (CH), 141.6 (CH), 140.1 (C), 134.3 (C), 133.9 (CH), 132.0 (C), 128.6 (CH), 127.84 (CH), 127.80 (CH), 127.2 (CH), 126.8 (C), 123.9 (CH), 122.9 (C), 113.3 (CH), 58.1 (CH), 56.6 (CH_3_), 41.9 (CH_2_). MS (EI, *m*/*z* (%)): 470/472 (M^+^/M+2^+^, 15/6), 359 (25), 331 (51), 153 (24), 94 (100), 39 (72). Anal. calcd. for C_22_H_19_ClN_4_O_4_S: C, 56.11; H, 4.07; N, 11.90; S, 6.81. Found: C, 56.01; H, 4.22; N, 11.98; S, 6.73.

##### 5-(5-(4-Chlorophenyl)-1-formyl-4,5-dihydro-1*H*-pyrazol-3-yl)-2,4-dimethoxybenzenesulfonamide (**22c**)

White solid; 86% yield; m.p. 269–271 °C. FTIR (ATR) υ(cm^−1^): 3319 and 3219 (N-H), 3122 (C-H Ar), 2847 (-(C=O)-H), 1651 (C=O), 1602 (C=N), 1590 (C=C), 1146 (S=O). ^1^H NMR (400 MHz, DMSO-*d*_6_) δ ppm 8.89 (s, 1H, CHO), 8.25 (s, 1H, H_6A_), 7.40 (d, *J* = 8.5 Hz, 2H, H_3D_), 7.24 (d, *J* = 8.5 Hz, 2H, H_2D_), 7.09 (s, 2H, NH_2_), 6.82 (s, 1H, H_3A_), 5.48 (dd, *J* = 11.7, 4.8 Hz, 1H, H_X_), 3.99 (s, 3H, 4A-CH_3_), 3.97–3.88 (m, 4H, 2A-CH_3_ and H_M_), 3.15 (dd, *J* = 18.7, 4.8 Hz, 1H, H_A_). ^13^C NMR (101 MHz, DMSO-*d*_6_) δ ppm 162.2 (C), 159.7 (CHO), 159.2 (C), 154.3 (C), 140.4 (C), 132.0 (C), 128.8 (CH), 128.2 (CH), 127.6 (CH), 124.2 (C), 110.8 (C), 97.3 (CH), 57.7 (CH), 56.6 (CH_3_), 56.5 (CH_3_), 45.3 (CH_2_). MS (EI, *m*/*z* (%)): 423/425 (M^+^/M+2^+^, 100/50), 284 (69), 243 (83), 162 (56), 89 (50), 77 (43). Anal. calcd. for C_18_H_18_ClN_3_O_5_S: C, 51.01; H, 4.28; N, 9.91; S, 7.56. Found: C, 50.99; H, 4.35; N, 9.97; S, 7.39.

##### *N*,*N*-bis(2-Chloroethyl)-5-(5-(4-chlorophenyl)-1-formyl-4,5-dihydro-1*H*-pyrazol-3-yl)-2,4-dimethoxybenzenesulfonamide (**22d**)

White solid; 91% yield; m.p. 222–224 °C. FTIR (ATR) υ(cm^−1^): 2971 (C-H Ar), 2844 (-(C=O)-H), 1665 (C=O), 1605 (C=N), 1558 (C=C), 1152 (S=O). ^1^H NMR (400 MHz, CDCl_3_) δ ppm 8.93 (s, 1H, CHO), 8.47 (s, 1H, H_6A_), 7.31 (d, *J* = 8.4 Hz, 2H, H_3D_), 7.19 (d, *J* = 8.4 Hz, 2H, H_2D_), 6.49 (s, 1H, H_3A_), 5.43 (dd, *J* = 11.8, 4.9 Hz, 1H, H_X_), 4.00 (s, 3H, 4A-CH_3_), 3.93–3.83 (m, 4H, 2A-CH_3_ and H_M_), 3.64 (s, 8H, N-CH_2_ and Cl-CH_2_), 3.26 (dd, *J* = 18.6, 4.9 Hz, 1H, H_A_). ^13^C NMR (101 MHz, CDCl_3_) δ ppm 163.2 (C), 160.3 (CHO), 159.8 (C), 153.7 (C), 139.4 (C), 133.8 (C), 132.8 (CH), 129.3 (CH), 127.3 (CH), 120.6 (C), 112.7 (C), 95.9 (CH), 58.5 (CH), 56.5 (CH_3_), 56.2 (CH_3_), 51.2 (CH_2_), 45.6 (CH_2_), 42.4 (CH_2_). MS (EI, *m*/*z* (%)): 549 (M^+^, 100), 343 (37), 315 (37), 178 (32), 42 (36). Anal. calcd. for C_22_H_24_Cl_3_N_3_O_5_S: C, 48.14; H, 4.41; N, 7.66; S, 5.84. Found: C, 48.02; H, 4.52; N, 7.70; S, 5.71.

#### 3.2.12. General Procedure for the Synthesis of Pyrazoline–Sulfonamide Hybrids **23a-d**

A mixture of the corresponding chalcone **(16-18)b** or **21b** (0.27 mmol) and hydrazine monohydrate (5.3 mmol) in ethanol (1.0 mL) was heated at reflux for 0.67 h. The reaction mixture was cooled, then 1.0 mL of acetic anhydride was carefully added and stirred overnight. The product was filtered and washed with small portions of water and ethanol. Compounds **23a-d** did not require further purification.

##### 5-(1-Acetyl-5-(4-chlorophenyl)-4,5-dihydro-1*H*-pyrazol-3-yl)-2-methoxybenzenesulfonamide (**23a**)

White solid; 71% yield; m.p. 229–232 °C. FTIR (ATR) υ(cm^−1^): 3384 and 3281 (N-H), 3049 (C-H Ar), 1675 (C=O), 1605 (C=N), 1591 (C=C), 1153 (S=O). ^1^H NMR (400 MHz, DMSO-*d*_6_) δ ppm 8.17 (d, *J* = 2.4 Hz, 1H, H_6A_), 7.87 (dd, *J* = 8.7, 2.4 Hz, 1H, H_4A_), 7.37 (d, *J* = 8.2 Hz, 2H, H_2D_), 7.27 (d, *J* = 8.7 Hz, 1H, H_3A_), 7.24–7.16 (m, 4H, NH_2_ and H_3D_), 5.53 (dd, *J* = 11.6, 4.3 Hz, 1H, H_X_), 3.94 (s, 3H, OCH_3_), 3.85 (dd, *J* = 18.0, 11.6 Hz, 1H, H_M_), 3.09 (dd, *J* = 18.0, 4.3 Hz, 1H, H_A_), 2.28 (s, 3H, CH_3_). ^13^C NMR (101 MHz, DMSO-*d*_6_) δ ppm 167.8 (C), 157.7 (C), 153.5 (C), 141.4 (C), 132.6 (CH), 132.0 (C), 131.9 (C), 128.8 (CH), 127.7 (CH), 125.8 (CH), 123.0 (C), 113.2 (CH), 59.2 (CH), 56.7 (CH_3_), 42.1 (CH_2_), 21.9 (CH_3_). MS (EI, *m/z* (%)): 407/409 (M^+^/M+2^+^, 34/13), 365 (100), 254 (23), 213 (23), 57 (25), 43 (79). Anal. calcd. for C_18_H_18_ClN_3_O_4_S: C, 53.01; H, 4.45; N, 10.30; S, 7.86. Found: C, 52.98; H, 4.33; N, 10.51; S, 7.92.

##### 5-(L-acetyl-5-(4-chlorophenyl)-4,5-dihydro-1*H*-pyrazol-3-yl)-2-methoxy-*N*-(pyridin-3-yl)benzenesulfonamide (**23b**)

White solid; 80% yield; m.p. 265–268 °C. FTIR (ATR) υ(cm^−1^): 3196 (N-H), 3027 (C-H Ar), 1646 (C=O), 1606 (C=N), 1596 (C=C), 1156 (S=O). ^1^H NMR (400 MHz, DMSO-*d*_6_) δ ppm 10.42 (s, 1H, NH), 8.32 (s, 1H, H_2B_), 8.24–8.16 (m, 2H, H_6A_ and H_6B_), 7.87 (d, *J* = 8.7 Hz, 1H, H_4A_), 7.51 (d, *J* = 8.3 Hz, 1H, H_4B_), 7.36 (d, *J* = 8.0 Hz, 2H, H_3D_), 7.30–7.24 (m, 2H, H_3A_ and H_5B_), 7.20 (d, *J* = 8.0 Hz, 2H, H_2D_), 5.51 (dd, *J* = 12.0, 4.8 Hz, 1H, H_X_), 3.90 (s, 3H, OCH_3_), 3.82 (dd, *J* = 18.1, 12.0 Hz, 1H, H_M_), 3.12 (dd, *J* = 18.1, 4.8 Hz, 1H, H_A_), 2.28 (s, 3H, CH_3_). ^13^C NMR (101 MHz, DMSO-*d*_6_) δ ppm 167.5 (C), 157.5 (C), 152.9 (C), 145.2 (CH), 141.6 (CH), 141.2 (C), 134.4 (C), 133.8 (CH), 131.7 (C), 128.6 (CH), 127.7 (CH), 127.6 (CH), 127.3 (CH), 126.9 (C), 124.0 (CH), 123.3 (C), 113.4 (CH), 59.1 (CH), 56.6 (CH_3_), 41.9 (CH_2_), 21.7 (CH_3_). MS (EI, *m*/*z* (%)): 484/485 (M^+^/M+2^+^, 10/4), 373 (17), 331 (36), 153 (18), 94 (56), 43 (100). Anal. calcd. for C_23_H_21_ClN_4_O_4_S: C, 56.96; H, 4.36; N, 11.55; S, 6.61. Found: C, 57.06; H, 4.25; N, 11.30; S, 6.69.

##### 5-(L-acetyl-5-(4-chlorophenyl)-4,5-dihydro-1*H*-pyrazol-3-yl)-2,4-dimethoxybenzenesulfonamide (**23c**)

White solid; 86% yield; m.p. 159–161 °C. FTIR (ATR) υ(cm^−1^): 3441 and 3352 (N-H), 3021 (C-H Ar), 1654 (C=O), 1602 (C=N), 1582 (C=C), 1157 (S=O). ^1^H NMR (400 MHz, DMSO-*d*_6_) δ ppm 8.23 (s, 1H, H_6A_), 7.38 (d, *J* = 8.5 Hz, 2H, H_3D_), 7.19 (d, *J* = 8.5 Hz, 2H, H_2D_), 7.08 (s, 2H, NH_2_), 6.82 (s, 1H, H_3D_), 5.48 (dd, *J* = 11.8, 4.4 Hz, 1H, H_X_), 3.98 (s, 3H, 4A-OCH_3_), 3.93–3.85 (m, 4H, 2A-OCH_3_ and H_M_), 3.08 (dd, *J* = 18.6, 4.4 Hz, 1H, H_A_), 2.26 (s, 3H, CH_3_). ^13^C NMR (101 MHz, DMSO-*d*_6_) δ ppm 167.3 (C), 162.1 (C), 159.0 (C), 152.6 (C), 141.5 (C), 131.7 (C), 128.6 (CH), 128.2 (CH), 127.4 (CH), 124.1 (C), 111.2 (C), 97.3 (CH), 58.7 (CH), 56.6 (CH_3_), 56.5 (CH_3_), 45.1 (CH_2_), 21.7 (CH_3_). MS (EI, *m*/*z* (%)): 437/439 (M^+^/M+2^+^, 27/10), 395 (45), 284 (13), 243 (14), 57 (27), 43 (100). Anal. calcd. for C_19_H_20_ClN_3_O_5_S: C, 52.12; H, 4.60; N, 9.60; S, 7.32. Found: C, 52.25; H, 4.51; N, 9.72; S, 7.19.

##### 5-(L-acetyl-5-(4-chlorophenyl)-4,5-dihydro-1*H*-pyrazol-3-yl)-*N*,*N*-bis(2-chloroethyl)-2,4-dimethoxybenzenesulfonamide (**23d**)

White solid; 90% yield; m.p. 196–198 °C. FTIR (ATR) υ(cm^−1^): 2976 (C-H Ar), 1643 (C=O), 1600 (C=N), 1576 (C=C), 1140 (S=O). ^1^H NMR (400 MHz, CDCl_3_) δ ppm 8.45 (s, 1H, H_6A_), 7.28 (d, *J* = 8.1 Hz, 2H H_3D_), 7.16 (d, *J* = 8.1 Hz, 2H, H_2D_), 6.48 (s, 1H, H_3A_), 5.49 (dd, *J* = 11.9, 4.7 Hz, 1H, H_X_), 4.00 (s, 3H, 4A-OCH_3_), 3.91 (s, 3H, 2A-OCH_3_), 3.82 (dd, *J* = 18.5, 11.9 Hz, 1H, H_M_), 3.65 (s, 8H, N-CH_2_ and Cl-CH_2_), 3.20 (dd, *J* = 18.5, 4.7 Hz, 1H, H_A_), 2.40 (s, 3H, CH_3_). ^13^C NMR (101 MHz, CDCl_3_) δ 169.1 (C), 163.1 (C), 159.6 (C), 151.9 (C), 140.7 (C), 133.4 (C), 132.7 (CH), 129.1 (CH), 127.2 (CH), 120.5 (C), 113.3 (C), 95.9 (CH), 59.5 (CH), 56.5 (CH_3_), 56.2 (CH_3_), 51.3 (CH_2_), 45.4 (CH_2_), 42.4 (CH_2_), 22.1 (CH_3_). MS (EI, *m*/*z* (%)): 563 (7), 519 (12), 243 (8), 71 (17), 43 (100). Anal. calcd. for C_23_H_26_Cl_3_N_3_O_5_S: C, 49.08; H, 4.66; N, 7.47; S, 5.70. Found: C, 49.15; H, 4.39; N, 7.60; S, 5.48.

#### 3.2.13. General Procedure for the Synthesis of Pyrazoline–Sulfonamide Hybrids **24a-b**

A mixture of the respective chalcone **14a-b** (0.21 mmol) and hydrazine monohydrate (4.2 mmol) in ethanol (1.0 mL) was stirred at room temperature for 3 h, CCD analysis revealed consumption of the precursor and formation of several products. Subsequently, 1.0 mL of formic acid was slowly added and stirred at room temperature for an additional 4 h; water was added to the mixture and the precipitate formed was filtered and washed with water. Both products were purified by CC using silica gel as stationary phase, and CHCl_3_:MeOH (15:1) mixture was employed as mobile phase for compound **24a** and a DCM:MeOH (20:1) mixture for compound **24b**.

##### 5-(5-(4-Chlorophenyl)-1-formyl-4,5-dihydro-1*H*-pyrazol-3-yl)-*N*’-isonicotinoyl-2-methoxybenzenesulfonohydrazide (**24a**)

Pale yellow solid; 73% yield; m.p. 156–158 °C. FTIR (ATR) υ(cm^−1^): 3214 (N-H), 3038 (C-H Ar), 2841 (-(C=O)-H), 1655 (C=O), 1649 (C=O), 1603 (C=N), 1163 (S=O). ^1^H NMR (400 MHz, DMSO-*d*_6_) δ ppm 10.95 (s, 1H, S-NH), 9.88 (s, 1H, C-NH), 8.87 (s, 1H, CHO), 8.69 (d, *J* = 6.1 Hz, 2H, H_2C_), 8.19 (d, *J* = 2.3 Hz, 1H, H_6A_), 7.89 (dd, *J* = 8.7, 2.3 Hz, 1H, H_4A_), 7.58 (d, *J* = 6.1 Hz, 2H, H_3C_), 7.38 (d, *J* = 8.5 Hz, 2H, H_3D_), 7.30 (d, *J* = 8.7 Hz, 1H, H_3A_), 7.23 (d, *J* = 8.5 Hz, 2H, H_2D_), 5.51 (dd, *J* = 11.8, 5.0 Hz, 1H, H_X_), 3.96 (s, 3H, OCH_3_), 3.88 (dd, *J* = 18.1, 11.8 Hz, 1H, H_M_), 3.18 (dd, *J* = 18.1, 5.0 Hz, 1H, H_A_). ^13^C NMR (101 MHz, DMSO-*d*_6_) δ ppm 164.1 (C), 159.7 (CHO), 159.0 (C), 154.8 (C), 150.4 (CH), 140.1 (C), 138.9 (C), 133.7 (CH), 132.0 (C), 128.6 (CH), 127.8 (CH), 127.7 (C), 127.5 (CH), 122.2 (C), 121.2 (CH), 113.4 (CH), 58.0 (CH), 56.8 (CH_3_), 42.0 (CH_2_). MS (EI, *m*/*z* (%)): 513/515 (M^+^/M+2^+^, 14/6), 314 (43), 123 (43), 106 (95), 85 (100), 47 (78). Anal. calcd. for C_23_H_20_ClN_5_O_5_S: C, 53.75; H, 3.92; N, 13.63; S, 6.24. Found: C, 53.96; H, 3.85; N, 13.41; S, 6.20.

##### 5-(5-(4-Chlorophenyl)-1-formyl-4,5-dihydro-1*H*-pyrazol-3-yl)-*N*’-isonicotinoyl-2,4-dimethoxybenzenesulfonohydrazide (**24b**)

White solid; 56% yield; m.p. 166–168 °C. FTIR (ATR) υ(cm^−1^): 3166 (N-H), 2945 (C-H Ar), 2829 (-(C=O)-H), 1654 (C=O), 1647 (C=O), 1601 (C=N), 1167 (S=O). ^1^H NMR (400 MHz, DMSO-*d*_6_) δ ppm 10.95 (s, 1H, S-NH), 9.74 (s, 1H, C-NH), 8.86 (s, 1H, CHO), 8.70 (d, *J* = 5.4 Hz, 2H, H_2C_), 8.22 (s, 1H, H_6A_), 7.59 (d, *J* = 5.4 Hz, 2H, H_3C_), 7.37 (d, *J* = 8.3 Hz, 2H, H_3D_), 7.20 (d, *J* = 8.3 Hz, 2H, H_2D_), 6.81 (s, 1H, H_3A_), 5.45 (dd, *J* = 11.6, 4.5 Hz, 1H, H_X_), 3.98 (s, 3H, 4A-OCH_3_), 3.93–3.85 (m, 4H, 2A-OCH_3_ and H_M_), 3.10 (dd, *J* = 18.7, 4.5 Hz, 1H, H_A_). ^13^C NMR (101 MHz, DMSO-*d*_6_) δ ppm 164.1 (C), 163.2 (C), 160.8 (C), 159.7 (CHO), 154.0 (CH), 150.4 (C), 140.4 (C), 139.0 (C), 132.0 (C), 130.3 (CH), 128.7 (CH), 127.6 (CH), 121.3 (CH), 119.5 (C), 110.8 (C), 97.5 (CH), 57.7 (CH), 57.0 (CH_3_), 56.6 (CH_3_), 45.2 (CH_2_). MS (EI, *m*/*z* (%)): 544 (M^+^, 0.1), 531 (48), 460 (53), 341 (51), 43 (100). Anal. calcd. for C_24_H_22_ClN_5_O_6_S: C, 52.99; H, 4.08; N, 12.87; S, 5.89. Found: C, 53.05; H, 4.13; N, 12.66; S, 5.71.

#### 3.2.14. General Procedure for the Synthesis of Pyrazoline–Sulfonamide Hybrids **25a-b**

A mixture of the respective chalcone **14a-b** (0.21 mmol) and hydrazine monohydrate (4.2 mmol) in ethanol (1.0 mL) was stirred at room temperature for 3 h. Next, 1.0 mL of acetic anhydride was carefully added and stirred at room temperature for additional 4 h; water was added to the reaction mixture and the precipitate was filtered and washed with water. The products were purified by CC on silica gel, using a CHCl_3_:MeOH (15:1) mixture as eluent for compound **25a** and a DCM:MeOH (20:1) mixture for compound **25b**.

##### 5-(1-Acetyl-5-(4-chlorophenyl)-4,5-dihydro-1*H*-pyrazol-3-yl)-*N*’-isonicotinoyl-2-methoxybenzenesulfonohydrazide (**25a**)

Beige solid; 51% yield; m.p. 146–148 °C. FTIR (ATR) υ(cm^−1^): 3207 (N-H), 3014 (C-H Ar), 1655 (C=O), 1638 (C=O), 1603 (C=N), 1164 (S=O). ^1^H NMR (400 MHz, DMSO-*d*_6_) δ ppm 10.95 (s, 1H, S-NH), 9.87 (s, 1H, C-NH), 8.69 (d, *J* = 5.7 Hz, 2H, H_2C_), 8.16 (d, *J* = 2.3 Hz, 1H, H_6A_), 7.89 (dd, *J* = 8.7, 2.3 Hz, 1H, H_4A_), 7.59 (d, *J* = 5.7 Hz, 2H, H_3C_), 7.35 (d, *J* = 8.1 Hz, 2H, H_3D_), 7.29 (d, *J* = 8.7 Hz, 1H, H_3A_), 7.18 (d, *J* = 8.1 Hz, 2H, H_2D_), 5.51 (dd, *J* = 11.9, 4.7 Hz, 1H, H_X_), 3.96 (s, 3H, OCH_3_), 3.83 (dd, *J* = 18.1, 11.9 Hz, 1H, H_M_), 3.10 (dd, *J* = 18.1, 4.7 Hz, 1H, H_A_), 2.27 (s, 3H, CH_3_). ^13^C NMR (101 MHz, DMSO-*d*_6_) δ ppm 167.4 (C), 164.1 (C), 158.8 (C), 153.0 (C), 150.3 (CH), 141.2 (C), 138.9 (C), 133.5 (CH), 131.7 (C), 128.5 (CH), 127.8 (C), 127.5 (CH), 127.4 (CH), 122.6 (C), 121.2 (CH), 113.4 (CH), 58.9 (CH), 56.8 (CH), 41.9 (CH_2_), 21.7 (CH_3_). MS (EI, *m*/*z* (%)): 527/529 (M^+^/M+2^+^, 0.5/0.2), 328 (39), 286 (64), 106 (64), 78 (65), 43 (100). Anal. calcd. for C_24_H_22_ClN_5_O_5_S: C, 54.60; H, 4.20; N, 13.26; S, 6.07. Found: C, 54.79; H, 4.07; N, 13.35; S, 6.14.

##### 5-(L-acetyl-5-(4-chlorophenyl)-4,5-dihydro-1*H*-pyrazol-3-yl)-*N*’-isonicotinoyl-2,4-dimethoxybenzenesulfonohydrazide (**25b**)

White solid; 52% yield; m.p. 159–160 °C. FTIR (ATR) υ(cm^−1^): 3235 (N-H), 2927 (C-H Ar), 1675 (C=O), 1626 (C=O), 1600 (C=N), 1162 (S=O). ^1^H NMR (400 MHz, DMSO-*d*_6_) δ ppm 10.97 (s, 1H, S-NH), 9.77 (s, 1H, C-NH), 8.73–8.69 (m, 2H, H_2C_), 8.22 (s, 1H, H_6A_), 7.63–7.59 (m, 2H, H_3C_), 7.37 (d, *J* = 8.3 Hz, 2H, H_3D_), 7.17 (d, *J* = 8.3 Hz, 2H, H_2D_), 6.82 (s, 1H, H_3A_), 5.47 (dd, *J* = 11.9, 4.5 Hz, 1H, H_X_), 3.99 (s, 3H, 4A-OCH_3_), 3.93–3.82 (m, 4H, 2A-OCH_3_ and H_M_), 3.05 (dd, *J* = 18.7, 4.5 Hz, 1H H_A_), 2.26 (s, 3H, CH_3_). ^13^C NMR (101 MHz, DMSO-*d*_6_) δ ppm 167.3 (C), 164.1 (C), 163.1 (C), 160.7 (C), 152.3 (C), 150.4 (CH), 141.5 (C), 139.1 (C), 131.7 (C), 130.3 (CH), 128.6 (CH), 127.4 (CH), 121.3 (CH), 119.4 (C), 111.2 (C), 97.4 (CH), 58.7 (CH), 56.9 (CH_3_), 56.6 (CH_3_), 45.1 (CH_2_), 21.7 (CH_3_). MS (EI, *m*/*z* (%)): 558 (M^+^, 0.1), 358 (60), 316 (55), 106 (53), 43 (100). Anal. calcd. for C_25_H_24_ClN5O_6_S: C, 53.81; H, 4.34; N, 12.55; S, 5.75. Found: C, 53.69; H, 4.30; N, 12.32; S, 5.93.

#### 3.2.15. General Procedure for the Synthesis of Carbothioamide–Sulfonamide Hybrids **27a-f**

To a mixture of the corresponding chalcone **(16-21)b** (0.24 mmol) and thiosemicarbazide **26** (0.26 mmol) in ethanol (1.0 mL), 3 drops of 50% *w/v* NaOH were added and stirred at 60 °C. After completion of the reaction, water was added, the medium was neutralized with aqueous 10% *v/v* HCl, and the solid formed was filtered and washed with water. Purification of the products was carried out by CC on silica gel, using a CHCl_3_:MeOH (15:1) mixture as the mobile phase.

##### 5-(4-Chlorophenyl)-3-(4-methoxy-3-sulfamoylphenyl)-4,5-dihydro-1*H*-pyrazole-1-carbothioamide (**27a**)

Beige solid; 36% yield; m.p. 206–208 °C. FTIR (ATR) υ(cm^−1^): 3503 and 3384 (N-H), 3326 (N-H), 3080 (C-H Ar), 3256 (N-H), 1604 (C=N), 1567 (C=C), 1162 (S=O). ^1^H NMR (400 MHz, DMSO-*d*_6_) δ ppm 8.20 (d, *J* = 2.3 Hz, 1H, H_6A_), 8.07–8.03 (m, 2H, C-NH and H_4A_), 7.94 (s, 1H, C-NH), 7.39 (d, *J* = 8.1 Hz, 2H, H_3D_), 7.31 (d, *J* = 8.7 Hz, 1H, H_3A_), 7.21–7.14 (m, 4H, H_2D_ and NH_2_), 5.95 (dd, *J* = 11.4, 3.5 Hz, 1H, H_X_), 4.01–3.88 (m, 4H, H_M_ and OCH_3_), 3.14 (dd, *J* = 17.9, 3.5 Hz, 1H, H_A_). ^13^C NMR (101 MHz, DMSO-*d*_6_) δ 176.0 (C), 157.7 (C), 154.0 (C), 141.9 (C), 132.6 (CH), 131.7 (C), 131.4 (C), 128.5 (CH), 127.3 (CH), 126.3 (CH), 122.6 (C), 113.1 (CH), 62.4 (CH), 56.5 (CH_3_), 42.2 (CH_2_). MS (EI, *m/z* (%)): 424/426 (M^+^/M+2^+^, 5/2), 365 (16), 211 (22), 71 (54), 57 (100), 43 (85). Anal. calcd. for C_17_H_17_ClN_4_O_3_S_2_: C, 48.05; H, 4.03; N, 13.19; S, 15.09. Found: C, 47.96; H, 4.12; N, 13.27; S, 15.11.

##### 5-(4-Chlorophenyl)-3-(4-methoxy-3-(*N*-(pyridin-3-yl)sulfamoyl)phenyl)-4,5-dihydro-1*H*-pyrazole-1-carbothioamide (**27b**)

Pale yellow solid; 33% yield; m.p. 236–238 °C. FTIR (ATR) υ(cm^−1^): 3433 and 3352 (N-H), 3069 (C-H Ar), 3269 (N-H), 1601 (C=N), 1584 (C=C), 1162 (S=O). ^1^H NMR (400 MHz, DMSO-*d*_6_) δ ppm 10.36 (s, 1H, S-NH), 8.32 (s, 1H, H_2B_), 8.26 (s, 1H, H_6A_), 8.20 (d, *J* = 4.8 Hz, 1H, H_6B_), 8.05–7.97 (m, 3H, H_4A_ and C-NH_2_), 7.50 (d, *J* = 8.3 Hz, 1H, H_4B_), 7.36 (d, *J* = 8.1 Hz, 2H, H_3D_), 7.28–7.23 (m, 2H, H_3A_ and H_5B_), 7.14 (d, *J* = 8.1 Hz, 2H, H_2D_), 5.89 (dd, *J* = 11.2, 3.7 Hz, 1H, H_X_), 3.94–3.84 (m, 4H, H_M_ and OCH_3_), 3.13 (dd, *J* = 18.0, 3.7 Hz, 1H, H_A_). ^13^C NMR (101 MHz, DMSO-*d*_6_) δ ppm 176.1 (C), 157.7 (C), 153.5 (C), 145.0 (CH), 141.9 (C), 141.4 (CH), 134.4 (C), 134.1 (CH), 131.4 (C), 128.5 (CH), 128.4 (CH), 127.4 (CH), 126.9 (CH), 126.8 (C), 123.9 (CH), 123.1 (C), 113.3 (CH), 62.4 (CH), 56.6 (CH_3_), 42.1 (CH_2_). MS (EI, *m*/*z* (%)): 502/504 (M^+^/M+2^+^, 2/1), 468 (13), 441 (74), 413 (25), 331 (100), 290 (26). Anal. calcd. for C_22_H_20_ClN_5_O_3_S_2_: C, 52.64; H, 4.02; N, 13.95; S, 12.77. Found: C, 52.37; H, 4.15; N, 14.09; S, 12.48.

##### 5-(4-Chlorophenyl)-3-(3-((2-isonicotinoylhydrazineyl)sulfonyl)-4-methoxyphenyl)-4,5-dihydro-1*H*-pyrazole-1-carbothioamide (**27c**)

Pale yellow solid; 35% yield; m.p. 216–218 °C. FTIR (ATR) υ(cm^−1^): 3459 and 3339 (N-H), 3237 (N-H), 3082 (C-H Ar), 1689 (C=O), 1604 (C=N), 1584 (C=C), 1166 (S=O). ^1^H NMR (400 MHz, DMSO-*d*_6_) δ ppm 10.93 (s, 1H, S-NH), 9.85 (s, 1H, C-NH), 8.67 (d, *J* = 5.2 Hz, 2H, H_2C_), 8.21 (d, *J* = 2.2 Hz, 1H, H_6A_), 8.07–7.92 (m, 3H, H_4A_ and NH_2_), 7.58 (d, *J* = 5.2 Hz, 2H, H_3C_), 7.34 (d, *J* = 8.1 Hz, 2H, H_3D_), 7.29 (d, *J* = 8.8 Hz, 1H, H_3A_), 7.11 (d, *J* = 8.1 Hz, 2H, H_2D_), 5.89 (dd, *J* = 11.6, 3.5 Hz, 1H, H_X_), 3.97 (s, 3H, OCH_3_), 3.88 (dd, *J* = 18.1, 11.6 Hz, 1H, H_M_), 3.10 (dd, *J* = 18.1, 3.5 Hz, 1H, H_A_). ^13^C NMR (101 MHz, DMSO-*d*_6_) δ ppm 176.0 (C), 164.2 (C), 159.0 (C), 153.8 (C), 150.3 (CH), 141.9 (C), 138.9 (C), 133.9 (CH), 131.4 (C), 128.4 (CH), 127.95 (CH), 127.87 (C), 127.3 (CH), 122.4 (C), 121.3 (CH), 113.4 (CH), 62.4 (CH), 56.8 (CH_3_), 42.2 (CH_2_). MS (EI, *m*/*z* (%)): 545 (M^+^, 0.1), 211 (22), 106 (49), 78 (60), 60 (82), 43 (100). Anal. calcd. for C_23_H_21_ClN_6_O_4_S_2_: C, 50.69; H, 3.88; N, 15.42; S, 11.76. Found: C, 50.80; H, 3.95; N, 15.36; S, 11.61.

##### 5-(4-Chlorophenyl)-3-(2,4-dimethoxy-5-sulfamoylphenyl)-4,5-dihydro-1*H*-pyrazole-1-carbothioamide (**27d**)

Pale yellow solid; 34% yield; m.p. 223–225 °C. FTIR (ATR) υ(cm^−1^): 3431 and 3255 (N-H), 3067 (C-H Ar), 1601 (C=N), 1576 (C=C), 1151 (S=O). ^1^H NMR (400 MHz, DMSO-*d*_6_) δ ppm 8.30 (s, 1H, H_6A_), 8.00 (s, 1H, C-NH), 7.65 (s, 1H, C-NH), 7.37 (d, *J* = 8.2 Hz, 2H, H_3D_), 7.14 (d, *J* = 8.2 Hz, 2H, H_2D_), 7.05 (s, 2H, S-NH_2_), 6.80 (s, 1H, H_3A_), 5.87 (dd, *J* = 11.4, 3.2 Hz, 1H, H_X_), 4.02–3.93 (m, 4H, 4A-CH_3_ and H_M_), 3.91 (s, 3H, 2A-CH_3_), 3.09 (dd, *J* = 18.7, 3.2 Hz, 1H, H_A_). ^13^C NMR (101 MHz, DMSO-*d*_6_) δ ppm 175.8 (C), 162.3 (C), 159.2 (C), 153.7 (C), 142.0 (C), 131.4 (C), 128.53 (CH), 128.47 (CH), 127.2 (CH), 124.3 (C), 110.8 (C), 97.2 (CH), 62.1 (CH), 56.6 (CH_3_), 56.5 (CH_3_), 45.5 (CH_2_). MS (EI, *m*/*z* (%)): 454/456 (M^+^/M+2^+^, 6/3), 395 (17), 127 16), 85 (22), 60 (51), 43 (100). Anal. calcd. for C_18_H_19_ClN_4_O_4_S_2_: C, 47.52; H, 4.21; N, 12.32; S, 14.09. Found: C, 47.36; H, 4.11; N, 12.49; S, 14.07.

##### 3-(5-(*N*,*N*-bis(2-Chloroethyl)sulfamoyl)-2,4-dimethoxyphenyl)-5-(4-chlorophenyl)-4,5-dihydro-1*H*-pyrazole-1-carbothioamide (**27e**)

Beige solid; 34% yield; m.p. 196–198 °C. FTIR (ATR) υ(cm^−1^): 3405 and 3254 (N-H), 3152 (C-H Ar), 1595 (C=N), 1579 (C=C), 1148 (S=O). ^1^H NMR (400 MHz, CDCl_3_) δ ppm 8.46 (s, 1H, H_6A_), 7.30 (d, *J* = 8.4 Hz, 2H, H_3D_), 7.20–7.05 (m, 3H, H_2D_ and C-NH), 6.47 (s, 1H, H_3A_), 6.08 (s, 1H, C-NH), 5.94 (dd, *J* = 11.5, 3.5 Hz, 1H, H_X_), 4.01 (s, 3H, 4A-OCH_3_), 3.96–3.87 (m, 4H, 2A-OCH_3_ and H_M_), 3.65 (s, 8H, N-CH_2_ and Cl-CH_2_), 3.25 (dd, *J* = 18.5, 3.5 Hz, 1H, H_A_). ^13^C NMR (101 MHz, CDCl_3_) δ ppm 176.7 (C), 163.5 (C), 160.0 (C), 154.0 (C), 140.6 (C), 133.4 (C), 132.7 (CH), 129.2 (CH), 127.1 (CH), 120.7 (C), 112.4 (C), 95.9 (CH), 63.0 (CH), 56.6 (CH_3_), 56.3 (CH_3_), 51.2 (CH_2_), 46.1 (CH_2_), 42.4 (CH_2_). MS (EI, *m*/*z* (%)): 580 (M^+^, 3), 211 (32), 102 (59), 50 (100), 42 (94). Anal. calcd. for C_22_H_25_Cl_3_N_4_O_4_S_2_: C, 45.56; H, 4.35; N, 9.66; S, 11.06. Found: C, 45.72; H, 4.21; N, 9.45; S, 11.24.

##### 5-(4-Chlorophenyl)-3-(5-((2-isonicotinoylhydrazineyl)sulfonyl)-2,4-dimethoxyphenyl)-4,5-dihydro-1*H*-pyrazole-1-carbothioamide (**27f**)

Yellow solid; 60% yield; m.p. 180–182 °C. FTIR (ATR) υ(cm^−1^): FTIR (ATR) υ(cm^−1^): 3554 and 3449 (N-H), 3317 (N-H), 3154 (C-H Ar), 1677 (C=N), 1582 (C=C), 1159 (S=O). ^1^H NMR (400 MHz, DMSO-*d*_6_) δ ppm 10.91 (s, 1H, S-NH), 9.69 (s, 1H, C-NH), 8.68 (d, *J* = 5.7 Hz, 2H, H_2C_), 8.32 (s, 1H, H_6A_), 8.00 (s, 1H, S=C-NH), 7.74 (s, 1H, S=C-NH), 7.59 (d, *J* = 5.7 Hz, 2H, H_3C_), 7.33 (d, *J* = 8.2 Hz, 2H, H_3D_), 7.10 (d, *J* = 8.2 Hz, 2H, H_2D_), 6.79 (s, 1H, H_3A_), 5.83 (dd, *J* = 11.4, 3.3 Hz, 1H, H_X_), 3.99 (s, 3H, 4A-OCH_3_), 3.94–3.86 (m, 4H, 2A-OCH_3_ and H_M_), 3.05 (dd, *J* = 18.6, 3.3 Hz, 1H, H_A_). ^13^C NMR (101 MHz, DMSO-*d*_6_) δ 175.8 (C), 164.2 (C), 163.2 (C), 160.8 (C), 153.3 (C), 150.3 (CH), 142.0 (C), 139.0 (C), 131.3 (C), 130.5 (CH), 128.4 (CH), 127.2 (CH), 121.3 (CH), 119.8 (C), 110.9 (C), 97.4 (CH), 62.0 (CH), 56.9 (CH_3_), 56.5 (CH_3_), 45.5 (CH_2_). MS (EI, *m*/*z* (%)): 575 (M^+^, 0.6), 473 (10), 315 (30), 106 (82), 43 (100). Anal. calcd. for C_24_H_23_ClN_6_O_5_S_2_: C, 50.13; H, 4.03; N, 14.61; S, 11.15. Found: C, 50.06; H, 4.24; N, 14.60; S, 11.39.

### 3.3. Anticancer Activity

All compounds selected by the US National Cancer Institute (NCI) were initially tested at a single dose (10^−5^ M) on the full panel of 60 cancer cell lines represented by leukemia, melanoma, lung, colon, central nervous system (CNS), ovarian, kidney, prostate and breast cancer. Only compounds that were able to satisfy the predetermined inhibition criteria against a minimum number of cell lines proceeded to five-dose assays (100, 10, 1.0, 1.0, 0.1 and 0.01 μM).

In these studies, human cancer cell lines were cultured in a RPMI-1640 medium containing 5% fetal bovine serum and 2 mM of *L*-glutamine. Following the protocol for a typical assay, cells were inoculated into 96-well plates and incubated at 37 °C, 5% CO_2_, 95% air, and 100% relative humidity for 24 h prior to the addition of the compounds to be tested. After 24 h, two plates of each cell line were fixed in situ with trichloroacetic acid (TCA) to represent a measure of the cell population for each cell line at the time of sample addition (Tz). Compounds were dissolved in DMSO to 400-fold the maximum desired concentration for analysis and subsequently diluted to twice the maximum desired concentration with medium containing 50 μg/mL gentamicin. An additional series of four dilutions at 10-fold concentration was performed to provide a total of five concentrations of the compound plus the control. Aliquots of 100 μL of these dilutions were taken and added to 96-well plates already containing 100 μL of the medium, resulting in the final required concentrations of the sample. The plates were incubated for 48 h at the same conditions. For adherent cells, the assay was terminated with the addition of cold TCA. Cells were fixed in situ by the slow addition of 50 μL of 50% (*w/v*) cold ATC (final concentration, 10% TCA) and incubated for 60 min at 4 °C. The supernatant was discarded, and the plates were washed five times with tap water and air dried. A quantity of 100 μL of 0.4% (*w/v*) sulforhodamine B (SRB) solution in 1% acetic acid was added to each well, and each plate was incubated for 10 min at room temperature. Upon completion of staining, unbound pigment was removed by five washes with 1% acetic acid and the plates were air-dried. The stains were solubilized with 10 mM Trizma base and absorbance was recorded on an automated plate reader at a wavelength of 515 nm. Using the absorbance measurements [time zero (Tz), growth control in the absence of the sample and growth test in the presence of the sample at the five concentration levels (Ti)], the percent growth was calculated for each sample at the concentration levels. The percent growth was calculated as: [(Ti − Tz)/(C − Tz)] × 100 for concentrations for which Ti > Tz, and [(Ti − Tz)/Tz] × 100 for concentrations for which Ti < Tz. Two dose-response parameters were calculated for each compound. The 50% growth inhibition (GI_50_) was calculated from [(Ti − Tz)/(C − Tz)] × 100 = 50, which is the sample concentration at which the net increase in protein is 50% lower in treated cells (as measured by SRB staining) compared to the net increase in protein seen in control cells and the LC_50_ (sample concentration at which a 50% reduction in protein measured at the end of treatment compared to the beginning), indicating a net loss of cells; calculated from [(Ti − Tz)/Tz] × 100 = −50. Values were calculated for each of these two parameters if the activity level was reached; however, if the effect was not reached or was exceeded, the value for that parameter was expressed as greater or less than the maximum or minimum of the concentration tested [67].

### 3.4. Antituberculosis Activity 

The agar dilution spot culture growth inhibition assay (SPOTi) [61] was performed to evaluate the minimum inhibitory concentration (MIC) values of the synthesized compounds against *Mycobacterium bovis* BCG and *Mycobacterium tuberculosis* H37Rv (ATCC 27294). A stock solution of 100 mg/mL in DMSO of each compound was prepared and then dilutions were made in DMSO until reaching concentrations of 10, 5, 1, 0.5, 0.1 and 0.01 mg/mL. Initially, each compound was evaluated at a concentration of 10 mg/L. The compounds that showed antitubercular activity at this concentration were evaluated at lower concentrations (5, 1, 0.5, 0.1 and 0.01 mg/L). Antituberculosis activity was assessed in a 24-well plate, and 2 μL of the DMSO dilution from each compound was dispensed into each well. Two mL of Middlebrook 7H10 culture medium (HiMedia, Mumbai, India) supplemented with glycerol 0.5% and ADNaCl (Albumin, Dextrose and Sodium Chloride) for *M. bovis* BCG*,* or with glycerol 0.5% with OADC (10% oleic acid, albumin, dextrose and catalase) for *M. tuberculosis* were added to each well to reach desired concentration of the compounds in solid medium. Dilutions of the bacterial cultures were prepared at a cell density of 10^6^ CFU/mL in Middlebrook 7H9 from strains of *M. bovis* BCG and *M. tuberculosis* H_37_Rv grown for 4 weeks in supplemented Middlebrook 7H9 medium at a temperature of 37 °C. A volume of 2 μL of the inoculum was carefully dispensed into the middle of each well and the plates were incubated at 37 °C for 2–3 weeks. Isoniazid was included as a positive control at concentrations of 10, 1, 0.1, 0.1, 0.05 and 0.01 mg/L. The plates were carefully observed, and MIC values were recorded as the lowest concentration of compound that completely inhibited bacterial growth upon visual inspection [68]. The experiment was performed in triplicate.

Growth curves were performed for *M. tuberculosis* H37Rv in liquid medium Middlebrook 7H9 supplemented with glycerol 0.5% with OADC (10% oleic acid, albumin, dextrose and catalase). The compounds **20e** and **20f** were added from the start of the experiment at the desired concentration from the prepared dilutions in DMSO. The day of the start of the experiment and every 7 days until day 21, an aliquot of 1 mL was removed from the medium and optical density was measured at 600 nm using an equipment Multiskan^TM^ FC Microplate Photometer. In addition, every day of measurement of optical density, an aliquot of the liquid culture was removed, ten-fold diluted to 10^−4^ with supplemented Middlebrook 7H9 medium, and each ten-fold dilution plated in Petri dishes by triplicate in supplemented Middlebrook 7H10 agar media without compounds or antibiotics. After 3 weeks of incubation the number of colonies were counted in each Petri dish.

Compounds displaying MIC values < 10 mg/L against *M. tuberculosis* H_37_Rv were also evaluated against six resistant strains of *M. tuberculosis* using the same agar dilution method: An orphan strain, a rifampicin-resistant strain (ATCC 35838), an isoniazid-resistant strain (ATCC 35822), and three multi-resistant strains (Haarlem, LAM9 SIT 42, Beijing). Levofloxacin was used as a positive control and DMSO as a negative control. 

### 3.5. Cytotoxicity on Vero Cell Line 

The VERO African green monkey kidney cell line (ATCC CCL-81) was cultured in Gibco RPMI Media 1640 culture medium, supplemented with 10% Fetal Bovine Serum (FBS), and 0.1% antibiotics (penicillin, streptomycin) 50 μg/mL. The cells were kept at 37 °C, 5% CO_2_ and 100% relative humidity, until obtaining a 70% confluence. 

To evaluate the cytotoxic effect of the compounds, a cell viability test was performed, using the MTT reductase assay [69], which consisted of sowing in a plate of 96 wells, a cell density of 104 in each well with supplemented medium. The cells were then treated (in triplicate) with compounds that had an MIC less than or equal to 10μg/mL. The cells were exposed for 24 h to each compound in different concentrations (0.1, 0.5, 1, 5, 10, 20, 50, and 100 μM). After 24 h the compound was removed and the cells were washed with PBS 0.01M, then 150 μL of freshly prepared MTT (0.25 mg/mL) was added to each well and incubated for 3–4 h, until the presence of formazan was noticed in the negative control, then the MTT was removed and 100 μL of DMSO was added to dissolve the formazan. For this to dissolve, it was left at room temperature for 10 min. Next, a reading at 560 nm in a FLUOROstar^®^ Omega multiplied reader (BMG Labtech, Ortenberg, Germany) was performed. IC_50_ values were determined by interpolation from mean absorbance data of 100% viability (negative control) [70].

### 3.6. Synergism

Synergism assays were performed to compare the MICs of antituberculosis drugs alone and in combination with the compounds that presented lower MICs against *M. tuberculosis* H37Rv (**20a** and **20f**). The experimental protocol known as a “checkerboard” assay described by Ying et al. in 2021 [71] was employed for this purpose. The synergism assays were carried out in 96-well plates with Middlebrook 7H9 medium supplemented with OADC. The drugs and compounds were evaluated at different ranges of concentrations according to their potency: rifampin 0.048 mg/L to 0.78 mg/L, isoniazid 0.006 mg/L to 1 mg/L, levofloxacin 0.063 mg/L to 1 mg/L, amikacin 0.188 mg/L to 3 mg/L, and compounds **20e** and **20f** from 0.625 mg/L to 20 mg/L. Plate readings were performed in a Multiskan^TM^ FC multiplate photometer (Thermo Scientific^TM^, Singapore) at 540 nm. The fractional inhibitory concentration index (FICI) was calculated using the formula: FICI = (MICAB/MICA) + (MICBA/MICB), where MICAB is the MIC of A in the presence of B, and MICBA is the MIC of B in the presence of A, and MICA is the MIC of A, and MICB is the MIC of B. Synergism is suggested by a FICI index ≤ 0.5, whereas a FICI index ≥ 4.0 suggests antagonism. A FICI index between 0.5 and 4.0, suggests no interaction between the active ingredients [72].

## 4. Conclusions

Five new series of chalcone–sulfonamide hybrids **(16-20) a-f** were synthesized via Claisen–Schmidt condensation of sulfonamides **8a-b**, **10**, **12*,*** and **14a-b** with aromatic aldehydes **15a-f**. Twelve pyrazolines **(22-23)a-d** and **(24-25)a-b** and six carbothioamides **27a-f** were obtained from 4-Cl-substituted chalcones of each series. All hybrids were preliminarily subjected to in vitro anticancer screening against 60 NCI human cancer cell lines at a single concentration of 10 μM. Chalcones **17a-c** and **18e** were the most active with mean values of GI % of 71.79%, 94.14%, 81.17%, and 100.00%, respectively, and a 25.48% of lethality for **18e**. A subsequent screening of these compounds at five concentration levels revealed remarkable growth inhibition values against LOX IMVI (Melanoma*)* with GI_50_ of 0.34 μM, 0.73 μM, and 0.54 μM for **17a-c**. The entire leukemia panel was highly sensitive to compound **18e** displaying GI_50_ values between 0.99–2.52 μM. Anticancer trial results suggest that compounds with similar structures to **17a-c** and **18e** could be important templates for the future design and development of potential antiproliferative agents. Antituberculosis activity was measured against *M. tuberculosis* H37Rv and chalcones **17a**, **17f**, **19a**, **19d**, **20a**, and **20d-f** displaying MIC values between 8.97–28.79 μM. From additional studies performed against the Vero cell line, the tested compounds showed high cytotoxic effects indicating that structures should be optimized in order to improve selectivity.

## Figures and Tables

**Table 1:** In vitro cytotoxic activity recorded for hybrids **17a-c** and **18e** against the panel of 60 cancer cell lines in five-dose screen, expressed as IC_50_ and LC_50_ in micromolar (μM) ^a^.

Panel/Cell Line	Compound							
	17a		17b		17c		18e	
	IC_50_^b^	LC_50_^c^	IC_50_	LC_50_	IC_50_	LC_50_	IC_50_	LC_50_
**Leukemia**								
**CCRF-CEM**	3.93	>100	1.52	>100	3.99	>100	1.69	>100
**HL-60(TB)**	2.27	>100	1.91	>100	2.52	>100	1.60	>100
**K-562**	1.50	>100	1.85	>100	2.43	>100	1.30	>100
**MOLT-4**	10.1	>100	2.83	>100	3.07	>100	2.04	>100
**RPMI-8226**	2.98	>100	1.86	>100	3.20	>100	2.52	>100
**SR**	1.73	>100	2.11	>100	2.26	>100	0.99	>100
**Non-Small Cell Lung Cancer**								
**A549/ATCC**	10.5	49.0	3.17	>100	2.75	34.8	3.11	>100
**EKVX**	14.2	53.6	3.88	>100	4.33	43.3	2.69	>100
**HOP-62**	9.51	51.9	2.5	25.9	2.90	29.5	3.77	>100
**HOP-92**	2.06	37.1	1.46	24.9	2.30	35.4	>100	>100
**NCI-H226**	10.0	69.2	2.56	>100	4.14	90.8	2.61	>100
**NCI-H322M**	10.7	47.6	2.31	38.7	2.87	34.7	3.00	>100
**NCI-H460**	1.95	51.2	2.84	>100	2.24	44.3	1.95	>100
**NCI-H522**	6.56	49.7	1.63	7.14	2.48	32.5	1.66	8.59
**Colon Cancer**								
**COLO 205**	10.6	54.2	1.66	5.88	3.33	41.7	4.46	8.39
**HCC-2998**	12.5	54.7	2.41	22.3	2.39	22.3	3.54	6.35
**HCT-116**	1.49	67.2	0.75	5.13	30.6	58.0	2.82	6.47
**HCT-15**	3.55	57.5	1.46	>100	2.56	74.8	3.98	>100
**HT29**	4.02	42.5	2.18	9.97	3.50	64.7	5.92	>100
**KM12**	3.31	62.3	1.82	>100	3.13	>100	4.25	9.40
**SW-620**	2.68	48.5	2.49	95.9	3.43	75.8	25.1	>100
**CNS Cancer**								
**SF-268**	5.88	62.7	3.75	>100	3.20	>100	7.70	>100
**SF-295**	15.7	58.2	3.88	57.6	3.89	43.2	35.4	>100
**SF-539**	2.44	30.1	2.14	12.6	2.17	14.4	3.61	7.51
**SNB-19**	14.0	58.0	3.17	>100	3.65	45.3	5.71	45.7
**SNB-75**	2.08	26.9	1.50	6.95	1.84	18.2	8.53	>100
**U251**	2.92	39.2	2.18	>100	2.66	32.7	3.68	7.61
**Melanoma**								
**LOX IMVI**	0.34	41.4	0.73	7.03	0.54	76.2	2.89	5.79
**MALME-3M**	13.9	61.9	2.79	87.4	8.55	59.5	7.18	>100
**M14**	7.51	60.5	1.47	6.70	3.25	56.0	5.45	>100
**MDA-MB-435**	2.12	35.9	2.08	29.3	2.27	25.4	7.51	>100
**SK-MEL-2**	12.6	55.9	11.5	57.2	11.5	55.7	4.65	27.6
**SK-MEL-28**	12.5	51.0	3.03	33.4	4.09	40.8	3.54	6.88
**SK-MEL-5**	17.6	59.7	4.93	43.0	7.81	49.4	3.64	7.19
**UACC-257**	9.96	47.8	5.46	71.6	3.86	41.9	3.89	9.14
**UACC-62**	12.2	59.4	2.67	40.8	3.63	46.2	4.94	>100
**Ovarian Cancer**								
**IGROV1**	9.74	77.0	2.95	>100	3.56	72.4	9.05	>100
**OVCAR-3**	2.71	38.3	3.51	66.8	3.05	>100	6.62	>100
**OVCAR-4**	11.8	55.9	3.54	>100	3.66	53.2	>100	>100
**OVCAR-5**	13.2	53.7	3.18	39.0	2.57	27.2	6.80	>100
**OVCAR-8**	11.3	51.3	3.01	>100	2.17	15.8	>100	>100
**NCI/ADR-RES**	15.5	>100	3.97	>100	5.25	>100	>100	>100
**SK-OV-3**	14.4	52.9	2.80	35.4	3.18	32.5	13.0	>100
**Renal Cancer**								
**786-0**	4.82	63.2	1.90	9.83	3.85	>100	5.66	>100
**A498**	16.9	55.8	18.6	62.2	16.2	58.1	7.23	65.5
**ACHN**	6.99	44.8	3.16	33.4	3.86	37.3	6.16	>100
**CAKI-1**	1.86	39.5	1.89	32.9	2.42	35.2	>100	>100
**RXF 393**	1.90	30.0	1.62	6.89	2.24	28.8	2.83	6.44
**SN12C**	3.49	43.8	2.48	60.2	2.95	38.8	13.0	>100
**TK-10**	12.9	51.5	2.91	33.4	3.41	39.6	75.0	>100
**UO-31**	4.76	43.5	2.36	33.1	3.46	38.1	2.92	5.51
**Prostate Cancer**								
**PC-3**	5.95	50.4	1.60	20.8	3.42	55.1	>100	>100
**DU-145**	1.88	38.1	3.44	42.2	4.23	65.1	14.8	>100
**Breast Cancer**								
**MCF7**	0.97	62.5	4.11	40.3	2.48	54.1	4.36	35.5
**MDA-MB-231/ATCC**	11.9	61.6	18.9	>100	3.41	60.0	>100	>100
**HS 578T**	5.89	>100	>100	>100	3.50	>100	>100	>100
**BT-549**	2.65	40.6	2.73	5.97	2.18	31.6	2.92	6.24
**T-47D**	2.09	51.6	12.4	64.6	2.32	44.8	>100	>100
**MDA-MB-468**	1.20	43.9	3.08	6.54	1.96	54.3	3.66	8.84

^a^ Data obtained from NCI’s in vitro disease-oriented human cancer cell lines screen in μM. ^b^ IC_50_ indicates compound concentration resulting in 50% reduction in net protein content (as measured by SRB staining) in control cells during drug incubation, determined at five concentration levels (100, 10, 1.0, 0.1, and 0.01 μM). ^c^ LC_50_ is a parameter of cytotoxicity that reflects the concentration required to kill 50% of the cells.

**Table 2:** Anti-TB and cytotoxic activity for compounds **17a**, **17f**, **19a**, **19d**, **20a**, **20d-f**.

Compound	MIC Against *M. bovis* BCG (µM)	MIC Against *M. tuberculosis* H_37_Rv (µM)	Cytotoxicity IC_50_ on Vero Cell Line (µM)	Selectivity Index (SI) (SI = IC_50_/MIC)
**17a**	>29	29	1.06 ± 1.21	0.04
**17f**	>25	25	1.70 ± 1.74	0.07
**19a**	>23	23	6.32 ± 0.82	0.28
**19d**	>21	21	2.19 ± 0.68	0.10
**20a**	11	11	6.05 ± 1.39	0.28
**20d**	>20	20	1.00 ± 1.20	0.05
**20e**	9.0	9.0	0.26 ± 0.69	0.03
**20f**	20	9.8	2.55 ± 0.93	0.26
**Isoniazid**	0.36	0.36	nd	

**Table 3:** Antituberculosis activity of resistant strains of *Mycobacterium tuberculosis*, expressed in Minimum Inhibitory Concentration (MIC) in micromolar (µM) units.

Compound	a	b	c	d	e	f
**17a**	29	>29	>29	29	>29	29
**17f**	>2	>2	>2	>2	>25	>25
**19a**	>23	>23	23	23	>23	<11
**19d**	21	>21	21	21	>21	21
**20a**	>21	>21	21	<11	>21	<11 *
**20d**	>20	>20	>20	>20	>20	20
**20e**	>18	>18	<9.0 *	<9.0 *	>18	<9.0 *
**20f**	>20	>20	<10	20	>20	<10 *
**Levofloxacin**	2.8	2.8	2.8	2.8	2.8	2.8

* Represents compounds with activity below (10 mg/L). **a**. *M. tuberculosis* Orphan strain. **b.** *M. tuberculosis* Haarlem. **c.** *M. tuberculosis* LAM9 SIT 42. **d.** *M. tuberculosis* Beijing. **e.** *M. tuberculosis* ATCC 35822. **f.** *M. tuberculosis* ATCC 35838.

**Table 4:** MIC values of the compounds before and after combinations with antibiotics and the FICI index in M. tuberculosis H37Rv.

Combination	MIC mg/L		FICI Index
	Alone	Combination	
RIF	0.19	0.048	1.25
**20a**	5	5	
INH	0.012	0.006	0.75
**20a**	5	1.25	
LVX	0.5	0.5	1.25
**20a**	5	1.25	
AMK	1.5	1.5	2.00
**20a**	5	5	
RIF	0.39	0.048	0.37 *
**20f**	5	1.25	
INH	0.012	0.006	1.00
**20f**	5	2.5	
LVX	0.5	0.25	1.00
**20f**	5	2.5	
AMK	1.5	1.5	2.00
**20f**	5	5	

* Values of FICI ≤ 0.5 indicate synergistic activity and FICI > 0.5–4.0 indicate no interaction between the combined compounds.

## Data Availability

Not applicable.

## Supplementary Materials

The following supporting information can be downloaded at: https://www.mdpi.com/article/10.3390/ijms232012589/s1.

## References

[1] Santucci C., Carioli G., Bertuccio P., Malvezzi M., Pastorino U., Boffetta P., Negri E., Bosetti C., La Vecchia C. (2020). Progress in Cancer Mortality, Incidence, and Survival: A Global Overview. Eur. J. Cancer Prev..

[2] Shalini, Kumar V. (2021). Have Molecular Hybrids Delivered Effective Anti-Cancer Treatments and What Should Future Drug Discovery Focus On?. Expert Opin. Drug Discov..

[3] Global Tuberculosis Report. https://www.who.int/teams/global-tuberculosis-programme/tb-reports/global-tuberculosis-report-2021.

[4] Peloquin C.A., Davies G.R. (2021). The Treatment of Tuberculosis. Clin. Pharmacol. Ther..

[5] Furin J., Cox H., Pai M. (2019). Tuberculosis. Lancet.

[6] Natarajan A., Beena P.M., Devnikar A.V., Mali S. (2020). A Systemic Review on Tuberculosis. Indian J. Tuberc..

[7] Apaydın S., Török M. (2019). Sulfonamide Derivatives as Multi-Target Agents for Complex Diseases. Bioorg. Med. Chem. Lett..

[8] Scozzafava A., Carta F., Supuran C.T. (2013). Secondary and Tertiary Sulfonamides: A Patent Review. Expert Opin. Ther. Pat..

[9] Zhao C., Rakesh K.P., Ravidar L., Fang W.Y., Qin H.L. (2019). Pharmaceutical and Medicinal Significance of Sulfur (SVI)-Containing Motifs for Drug Discovery: A Critical Review. Eur. J. Med. Chem..

[10] Catarro M., Serrano J.L., Ramos S.S., Silvestre S., Almeida P. (2019). Nimesulide Analogues: From Anti-Inflammatory to Antitumor Agents. Bioorg. Chem..

[11] Roell D., Baniahmad A. (2011). The Natural Compounds Atraric Acid and N-Butylbenzene-Sulfonamide as Antagonists of the Human Androgen Receptor and Inhibitors of Prostate Cancer Cell Growth. Mol. Cell. Endocrinol..

[12] Carta F., Supuran C.T., Scozzafava A. (2014). Sulfonamides and Their Isosters as Carbonic Anhydrase Inhibitors. Future Med. Chem..

[13] Kawai T., Kazuhiko I., Takaya N., Yamaguchi Y., Kishii R., Kohno Y., Kurasaki H. (2017). Sulfonamide-Based Non-Alkyne LpxC Inhibitors as Gram-Negative Antibacterial Agents. Bioorg. Med. Chem. Lett..

[14] Tassini S., Sun L., Lanko K., Crespan E., Langron E., Falchi F., Kissova M., Armijos-Rivera J.I., Delang L., Mirabelli C. (2017). Discovery of Multitarget Agents Active as Broad-Spectrum Antivirals and Correctors of Cystic Fibrosis Transmembrane Conductance Regulator for Associated Pulmonary Diseases. J. Med. Chem..

[15] Thareja S., Kokil G.R., Aggarwal S., Bhardwaj T.R., Kumar M. (2010). Sulphonamides as Inhibitors of Protein Tyrosine Phosphatase 1B: A Three-Dimensional Quantitative Structure-Activity Relationship Study Using Self-Organizing Molecular Field Analysis Approach. Chem. Pharm. Bull..

[16] Supuran C.T., Capasso C. (2015). The η-Class Carbonic Anhydrases as Drug Targets for Antimalarial Agents. Expert Opin. Ther. Targets.

[17] Singh P., Anand A., Kumar V. (2014). Recent Developments in Biological Activities of Chalcones: A Mini Review. Eur. J. Med. Chem..

[18] Zhang M.R., Jiang K., Yang J.L., Shi Y.P. (2020). Flavonoids as Key Bioactive Components of Oxytropis Falcata Bunge, a Traditional Anti-Inflammatory and Analgesic Tibetan Medicine. Nat. Prod. Res..

[19] Sadula A., Peddaboina U.R., Prameela Subhashini N.J. (2015). Synthesis and Characterization of Novel Chalcone Linked Imidazolones as Potential Antimicrobial and Antioxidant Agents. Med. Chem. Res..

[20] Wang L., Yang X., Zhang Y., Chen R., Cui Y., Wang Q. (2019). Anti-Inflammatory Chalcone-Isoflavone Dimers and Chalcone Dimers from Caragana Jubata. J. Nat. Prod..

[21] Kontogiorgis C., Mantzanidou M., Hadjipavlou-Litina D. (2008). Chalcones and Their Potential Role in Inflammation. Mini-Rev. Med. Chem..

[22] Mateeva N., Eyunni S.V.K., Redda K.K., Ononuju U., Hansberry T.D., Aikens C., Nag A. (2017). Functional Evaluation of Synthetic Flavonoids and Chalcones for Potential Antiviral and Anticancer Properties. Bioorg. Med. Chem. Lett..

[23] Mahapatra D.K., Bharti S.K. (2016). Therapeutic Potential of Chalcones as Cardiovascular Agents. Life Sci..

[24] Mahapatra D.K., Asati V., Bharti S.K. (2015). Chalcones and Their Therapeutic Targets for the Management of Diabetes: Structural and Pharmacological Perspectives. Eur. J. Med. Chem..

[25] Gomes M.N., Braga R.C., Grzelak E.M., Neves B.J., Muratov E., Ma R., Klein L.L., Cho S., Oliveira G.R., Franzblau S.G. (2017). QSAR-Driven Design, Synthesis and Discovery of Potent Chalcone Derivatives with Antitubercular Activity. Eur. J. Med. Chem..

[26] Ejaz S.A., Saeed A., Siddique M.N., un Nisa Z., Khan S., Lecka J., Sévigny J., Iqbal J. (2017). Synthesis, Characterization and Biological Evaluation of Novel Chalcone Sulfonamide Hybrids as Potent Intestinal Alkaline Phosphatase Inhibitors. Bioorg. Chem..

[27] Gomes M.N., Muratov E.N., Pereira M., Peixoto J.C., Rosseto L.P., Cravo P.V.L., Andrade C.H., Neves B.J. (2017). Chalcone Derivatives: Promising Starting Points for Drug Design. Molecules.

[28] Zhuang C., Zhang W., Sheng C., Zhang W., Xing C., Miao Z. (2017). Chalcone: A Privileged Structure in Medicinal Chemistry. Chem. Rev..

[29] Bashir R., Ovais S., Yaseen S., Hamid H., Alam M.S., Samim M., Singh S., Javed K. (2011). Synthesis of Some New 1,3,5-Trisubstituted Pyrazolines Bearing Benzene Sulfonamide as Anticancer and Anti-Inflammatory Agents. Bioorg. Med. Chem. Lett..

[30] Tucker J.W., Chenard L., Young J.M. (2015). Selective Access to Heterocyclic Sulfonamides and Sulfonyl Fluorides via a Parallel Medicinal Chemistry Enabled Method. ACS Comb. Sci..

[31] Kucukoglu K., Oral F., Aydin T., Yamali C., Algul O., Sakagami H., Gulcin I., Supuran C.T., Gul H.I. (2016). Synthesis, Cytotoxicity and Carbonic Anhydrase Inhibitory Activities of New Pyrazolines. J. Enzyme Inhib. Med. Chem..

[32] Yamali C., Gul H.I., Kazaz C., Levent S., Gulcin I. (2020). Synthesis, Structure Elucidation, and in Vitro Pharmacological Evaluation of Novel Polyfluoro Substituted Pyrazoline Type Sulfonamides as Multi-Target Agents for Inhibition of Acetylcholinesterase and Carbonic Anhydrase I and II Enzymes. Bioorg. Chem..

[33] Shaaban M.R., Mayhoub A.S., Farag A.M. (2012). Recent Advances in the Therapeutic Applications of Pyrazolines. Expert Opin. Ther. Pat..

[34] Acharya P.T., Bhavsar Z.A., Jethava D.J., Patel D.B., Patel H.D. (2021). A Review on Development of Bio-Active Thiosemicarbazide Derivatives: Recent Advances. J. Mol. Struct..

[35] Tilekar K., Upadhyay N., Meyer-Almes F.J., Loiodice F., Anisimova N.Y., Spirina T.S., Sokolova D.V., Smirnova G.B., Choe J.Y., Pokrovsky V.S. (2020). Synthesis and Biological Evaluation of Pyrazoline and Pyrrolidine-2,5-Dione Hybrids as Potential Antitumor Agents. ChemMedChem.

[36] Montoya A., Quiroga J., Abonia R., Nogueras M., Cobo J., Insuasty B. (2014). Synthesis and in Vitro Antitumor Activity of a Novel Series of 2-Pyrazoline Derivatives Bearing the 4-Aryloxy-7-Chloroquinoline Fragment. Molecules.

[37] Chen K., Zhang Y.L., Fan J., Ma X., Qin Y.J., Zhu H.L. (2018). Novel Nicotinoyl Pyrazoline Derivates Bearing N-Methyl Indole Moiety as Antitumor Agents: Design, Synthesis and Evaluation. Eur. J. Med. Chem..

[38] Nehra B., Rulhania S., Jaswal S., Kumar B., Singh G., Monga V. (2020). Recent Advancements in the Development of Bioactive Pyrazoline Derivatives. Eur. J. Med. Chem..

[39] Iñiguez M.A., Punzón C., Cacheiro-Llaguno C., Díaz-Muñoz M.D., Duque J., Cuberes R., Alvarez I., Andrés E.M., Buxens J., Buschmann H. (2010). Cyclooxygenase-Independent Inhibitory Effects on T Cell Activation of Novel 4,5-Dihydro-3 Trifluoromethyl Pyrazole Cyclooxygenase-2 Inhibitors. Int. Immunopharmacol..

[40] Kharbanda C., Alam M.S., Hamid H., Javed K., Bano S., Dhulap A., Ali Y., Nazreen S., Haider S. (2014). Synthesis and Evaluation of Pyrazolines Bearing Benzothiazole as Anti-Inflammatory Agents. Bioorg. Med. Chem..

[41] Faidallah H.M., Al-Mohammadi M.M., Alamry K.A., Khan K.A. (2016). Synthesis and Biological Evaluation of Fluoropyrazolesulfonylurea and Thiourea Derivatives as Possible Antidiabetic Agents. J. Enzyme Inhib. Med. Chem..

[42] Özdemir A., Altintop M.D., Kaplancikli Z.A., Can Ö.D., Özkay Ü.D., Turan-Zitouni G. (2015). Synthesis and Evaluation of New 1,5-Diaryl-3-[4-(Methylsulfonyl)Phenyl]-4,5-Dihydro-1 H-Pyrazole Derivatives as Potential Antidepressant Agents. Molecules.

[43] Insuasty B., Montoya A., Becerra D., Quiroga J., Abonia R., Robledo S., Vélez I.D., Upegui Y., Nogueras M., Cobo J. (2013). Synthesis of Novel Analogs of 2-Pyrazoline Obtained from [(7-Chloroquinolin-4-Yl)Amino]Chalcones and Hydrazine as Potential Antitumor and Antimalarial Agents. Eur. J. Med. Chem..

[44] Fioravanti R., Desideri N., Carta A., Atzori E.M., Delogu I., Collu G., Loddo R. (2017). Inhibitors of Yellow Fever Virus Replication Based on 1,3,5-Triphenyl-4,5-Dihydropyrazole Scaffold: Design, Synthesis and Antiviral Evaluation. Eur. J. Med. Chem..

[45] Hassan G.S., Rahman D.E.A., Saleh D.O., Jaleel G.A.R.A. (2014). Benzofuran-Morpholinomethyl-Pyrazoline Hybrids as a New Class of Vasorelaxant Agents: Synthesis and Quantitative Structure-Activity Relationship Study. Chem. Pharm. Bull..

[46] Shin S.Y., Ahn S., Yoon H., Jung H., Jung Y., Koh D., Lee Y.H., Lim Y. (2016). Colorectal Anticancer Activities of Polymethoxylated 3-Naphthyl-5-Phenylpyrazoline-Carbothioamides. Bioorg. Med. Chem. Lett..

[47] Kim B.S., Shin S.Y., Ahn S., Koh D., Lee Y.H., Lim Y. (2017). Biological Evaluation of 2-Pyrazolinyl-1-Carbothioamide Derivatives against HCT116 Human Colorectal Cancer Cell Lines and Elucidation on QSAR and Molecular Binding Modes. Bioorg. Med. Chem..

[48] Lee Y., Kim B.S., Ahn S., Koh D., Lee Y.H., Shin S.Y., Lim Y. (2016). Anticancer and Structure-Activity Relationship Evaluation of 3-(Naphthalen-2-Yl)-N,5-Diphenyl-Pyrazoline-1-Carbothioamide Analogs of Chalcone. Bioorg. Chem..

[49] Wang H., Zheng J., Xu W., Chen C., Wei D., Ni W., Pan Y. (2017). A New Series of Cytotoxic Pyrazoline Derivatives as Potential Anticancer Agents That Induce Cell Cycle Arrest and Apoptosis. Molecules.

[50] Zaki Y.H., Al-Gendey M.S., Abdelhamid A.O. (2018). A Facile Synthesis, and Antimicrobial and Anticancer Activities of Some Pyridines, Thioamides, Thiazole, Urea, Quinazoline, β-Naphthyl Carbamate, and Pyrano[2,3-d]Thiazole Derivatives. Chem. Cent. J..

[51] Siddiqui N., Alam P., Ahsan W. (2009). Design, Synthesis, and In-Vivo Pharmacological Screening of N,3-(Substituted Diphenyl)-5-Phenyl-1 H-Pyrazoline-1- Carbothioamide Derivatives. Arch. Pharm..

[52] Mathew B., Ahsan M.J. (2014). Carboxamide/Carbothioamide Anticonvulsant Analogues Towards GABA-Aminotransferase- An in Silico Approach. Cent. Nerv. Syst. Agents Med. Chem..

[53] Yusuf M., Jain P. (2014). Synthetic and Biological Studies of Pyrazolines and Related Heterocyclic Compounds. Arab. J. Chem..

[54] Abdelhamid A.O., El Sayed I.E., Hussein M.Z., Mangoud M.M. (2016). Synthesis and Antimicrobial Activity of Some New Thiadiazoles, Thioamides, 5-Arylazothiazoles and Pyrimido[4,5-d][1,2,4]Triazolo[4,3-a]Pyrimidines. Molecules.

[55] Alex J.M., Kumar R. (2014). 4,5-Dihydro-1H-Pyrazole: An Indispensable Scaffold. J. Enzyme Inhib. Med. Chem..

[56] Castaño L.F., Cuartas V., Bernal A., Insuasty A., Guzman J., Vidal O., Rubio V., Puerto G., Lukáč P., Vimberg V. (2019). New Chalcone-Sulfonamide Hybrids Exhibiting Anticancer and Antituberculosis Activity. Eur. J. Med. Chem..

[57] Riaz S., Khan I.U., Bajda M., Ashraf M., Shaukat A., Rehman T.U., Mutahir S., Hussain S., Yar M., Khan I.U. (2015). Pyridine Sulfonamide as a Small Key Organic Molecule for the Potential Treatment of Type-II Diabetes Mellitus and Alzheimer’s Disease: In Vitro Studies against Yeast α-Glucosidase, Acetylcholinesterase and Butyrylcholinesterase. Bioorg. Chem..

[58] Mótyán G., Kovács F., Wölfling J., Gyovai A., Zupkó I., Frank É. (2016). Microwave-Assisted Stereoselective Approach to Novel Steroidal Ring D-Fused 2-Pyrazolines and an Evaluation of Their Cell-Growth Inhibitory Effects in Vitro. Steroids.

[59] Cavallo G., Metrangolo P., Milani R., Pilati T., Priimagi A., Resnati G., Terraneo G. (2016). The Halogen Bond. Chem. Rev..

[60] Zhao L., Wang Y., Cao D., Chen T., Wang Q., Li Y., Xu Y., Zhang N., Wang X., Chen D. (2015). Fragment-Based Drug Discovery of 2-Thiazolidinones as BRD4 Inhibitors: Structure-Based Optimization. J. Med. Chem..

[61] Evangelopoulos D., Bhakta S. (2010). Rapid methods for testing inhibitors of mycobacterial growth. in Antibiotic Resistance Protocols. Methods Mol. Biol..

[62] Brudey K., Driscoll J.R., Rigouts L., Prodinger W.M., Gori A., Al-Hajoj S.A., Allix C., Aristimuño L., Arora J., Baumanis V. (2006). Mycobacterium tuberculosis complex genetic diversity: Mining the fourth international spoligotyping database (SpolDB4) for classification, population genetics and epidemiology. BMC Microbiol..

[63] Puerto G., Erazo L., Wintaco M., Castro C., Ribón W., Guerrero M.I. (2015). Mycobacterium tuberculosis Genotypes Determined by Spoligotyping to Be Circulating in Colombia between 1999 and 2012 and Their Possible Associations with Transmission and Susceptibility to First-Line Drugs. PLoS ONE.

[64] Realpe T., Correa N., Rozo J.C., Ferro B.E., Gómez V., Zapata E., Wellman R., Puerta G., Castro C., Nieto L.M. (2014). Population Structure among Mycobacterium tuberculosis Isolates from Pulmonary Tuberculosis Patients in Colombia. PLoS ONE.

[65] Reynaud Y., Millet J., Rastogi N. (2015). Genetic Structuration, Demography and Evolutionary History of Mycobacterium tuberculosis LAM9 Sublineage in the Americas as Two Distinct Subpopulations Revealed by Bayesian Analyses. PLoS ONE.

[66] Cubillos A., Sandoval A., Ritacco V., López B., Robledo J., Correa N., Hernandez-Neuta I., Zambrano M.M., Del Portillo P. (2010). Genomic signatures of the Haarlem lineage of Mycobacterium tuberculosis: Implications of strain genetic variation in drug and vaccine development. J. Clin. Microbiol..

[67] National Cancer Insititute DCTD Division of Cancer Treatment & Diagnosis. https://dtp.cancer.gov/discovery_development/nci-60/methodology.htm.

[68] Guzman J.D., Mortazavi P.N., Munshi T., Evangelopoulos D., McHugh T.D., Gibbons S., Malkinson J., Bhakta S. (2014). 2-Hydroxy-Substituted Cinnamic Acids and Acetanilides Are Selective Growth Inhibitors of Mycobacterium Tuberculosis. Med. Chem. Comm..

[69] Chen T., Li Q., Guo L., Yu L., Li Z., Guo H., Li H., Zhao M., Chen L., Chen X. (2016). Lower cytotoxicity, high stability, and long-term antibacterial activity of a poly (methacrylic acid)/isoniazid/rifampin nanogel against multidrug-resistant intestinal Mycobacterium tuberculosis. Mater. Sci. Eng. C.

[70] Kabongo P.N., Eloff J.N., Obi C.L., Mcgaw L.J. (2016). Antimycobacterial Activity and Low Cytotoxicity of Leaf Extracts of Some African Anacardiaceae Tree Species. Phytother. Res..

[71] Ying R., Huang X., Gao Y., Wang J., Liu Y., Sha W., Yang H. (2021). In vitro Synergism of Six Antituberculosis Agents Against Drug-Resistant Mycobacterium tuberculosis Isolated from Retreatment Tuberculosis Patients. Infect. Drug Resist..

[72] Caleffi K.R., Maltempe F.G., Siqueira V.L.D., Cardoso R.F. (2013). Fast detection of drug interaction in Mycobacterium tuberculosis by a checkerboard resazurin method. Tuberculosis.

