# Supplementary material for: Synthesis, Anticancer and Antitubercular Properties of New Chalcones and Their Nitrogen-Containing Five-Membered Heterocyclic Hybrids Bearing Sulfonamide Moiety
Source: Int J Mol Sci. 2022 Oct 20;23(20):12589. doi: 10.3390/ijms232012589 (PMC9604400; doi:10.3390/ijms232012589)
Supplement: Supplementary file 1 [file ijms-23-12589-s001.zip › ijms-1967181-supplementary.pdf]

# Synthesis, Anticancer and Antitubercular Properties of New Chalcones and Their Nitrogen-Containing Five-Membered Heterocyclic Hybrids Bearing Sulfonamide Moiety

**Lina Fernanda Castaño [1], Jairo Quiroga [1,2], Rodrigo Abonia [1,2], Daniel Insuasty [3], Oscar M. Vidal [4], Rosalia Seña [3,4], Vivian Rubio [5], Gloria Puerto [5], Manuel Nogueras [6], Justo Cobo [6], Juan Guzman [7], Alberto Insuasty [8,*] and Braulio Insuasty [1,2,*]**

[1] Heterocyclic Compounds Research Group, Department of Chemistry, Universidad del Valle, A.A., Cali 25360, Colombia
[2] Centre for Bioinformatics and Photonics-CIBioFI, Universidad del Valle, A.A., Cali 25360, Colombia
[3] Department of Chemistry and Biology, Basic Sciences Division, Universidad del Norte, Barranquilla 081007, Colombia
[4] Department of Medicine, Health Division, Universidad del Norte, Barranquilla 081007, Colombia
[5] Grupo de Micobacterias, Red TB. Dirección de Investigación en Salud Pública, Instituto Nacional de Salud, Bogotá 111321, Colombia
[6] Department of Inorganic and Organic Chemistry, Universidad de Jaén, 23071 Jaén, Spain
[7] Department of Bioresources, Fraunhofer Institute for Molecular Biology and Applied Ecology, 35392 Giessen, Germany
[8] Nanostructured Functional Materials Research Group, Universidad CESMAG, Pasto 520003, Colombia
* Correspondence: bainsuasty@unicesmag.edu.co (A.I.); braulio.insuasty@correounivalle.edu.co (B.I.)

## SUPPORTING INFORMATION

NMR spectra of synthetized compounds ………………………………………….. File S1
Single Crystal Diffraction Experimental Details……………………………………. Table S1

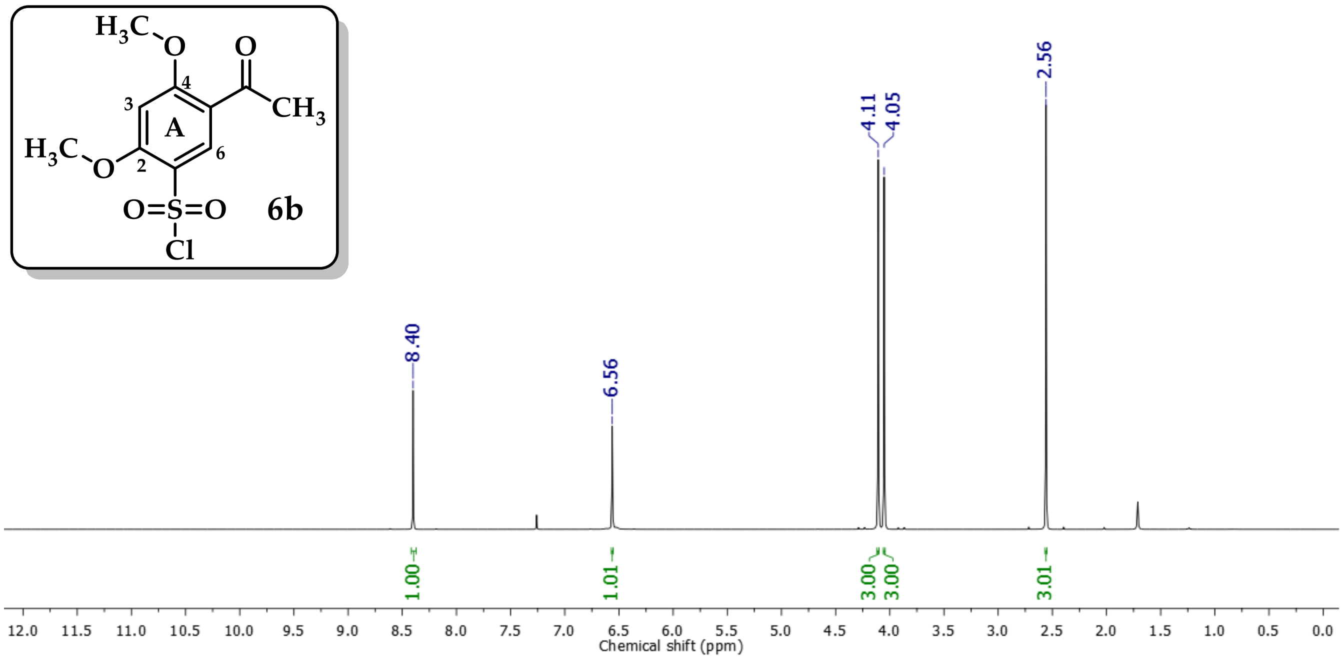


$^{1}$H NMR (400 MHz, $CDCl_3$) spectrum of **6b**.

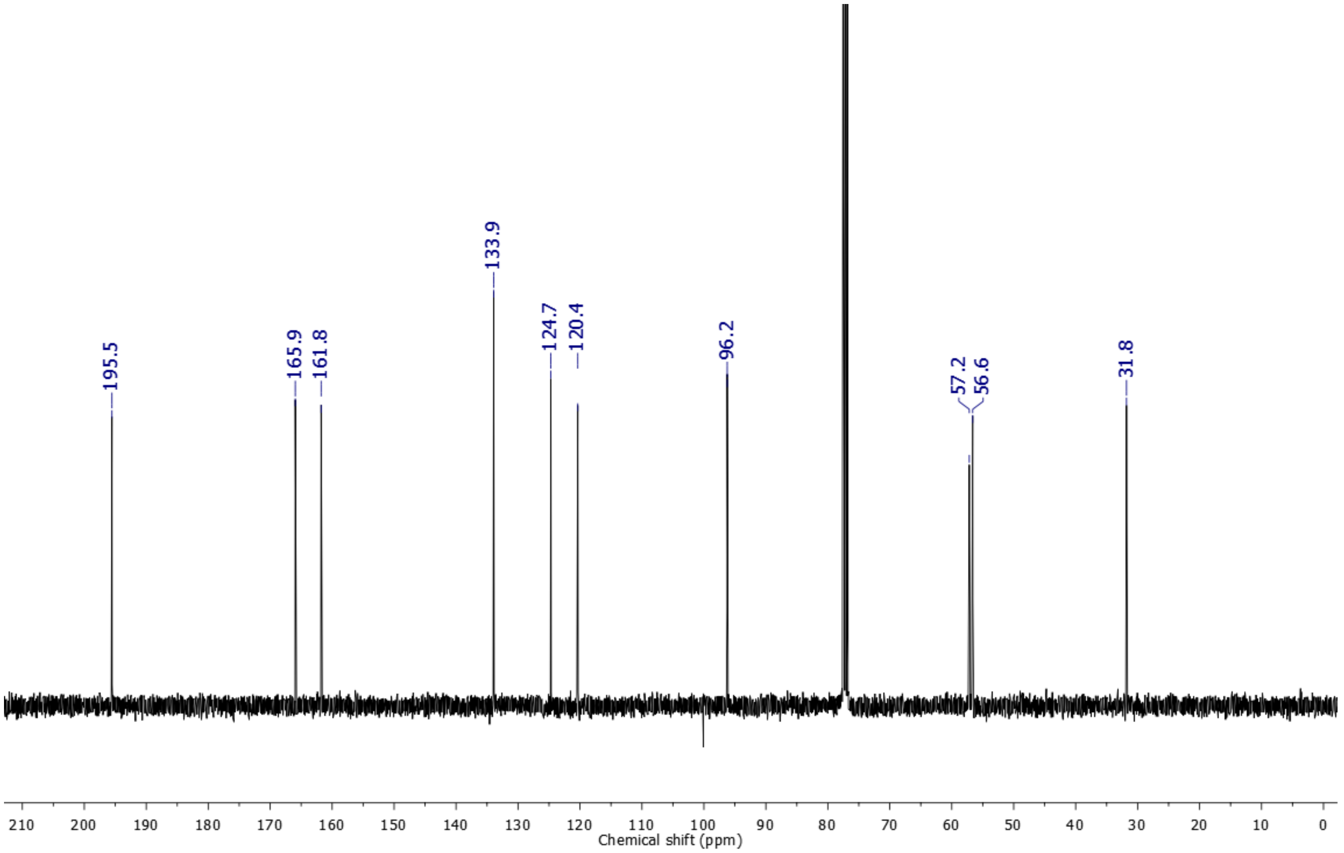


$^{13}$C NMR (101 MHz, $CDCl_3$) spectrum of **6b**.

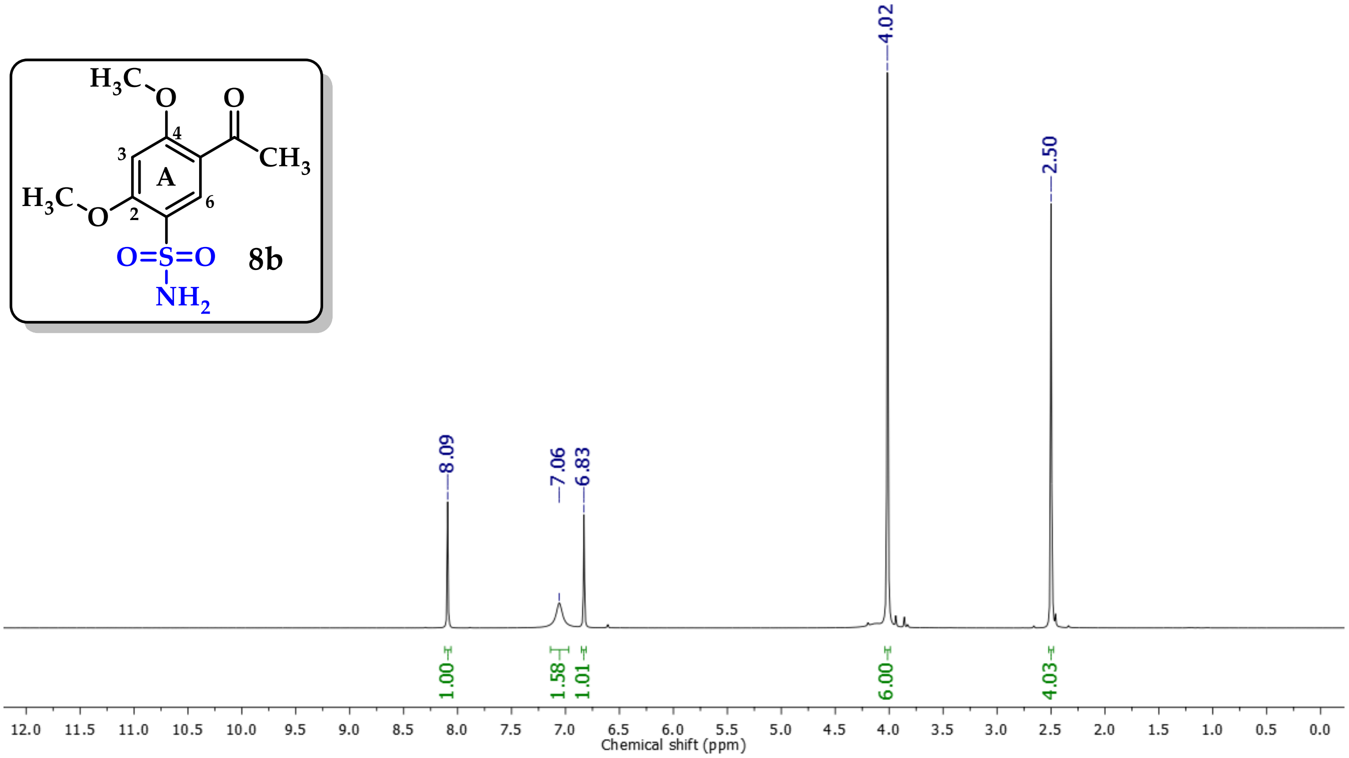


$^{1}$H NMR (400 MHz, DMSO-*d6*) spectrum of **8b**.

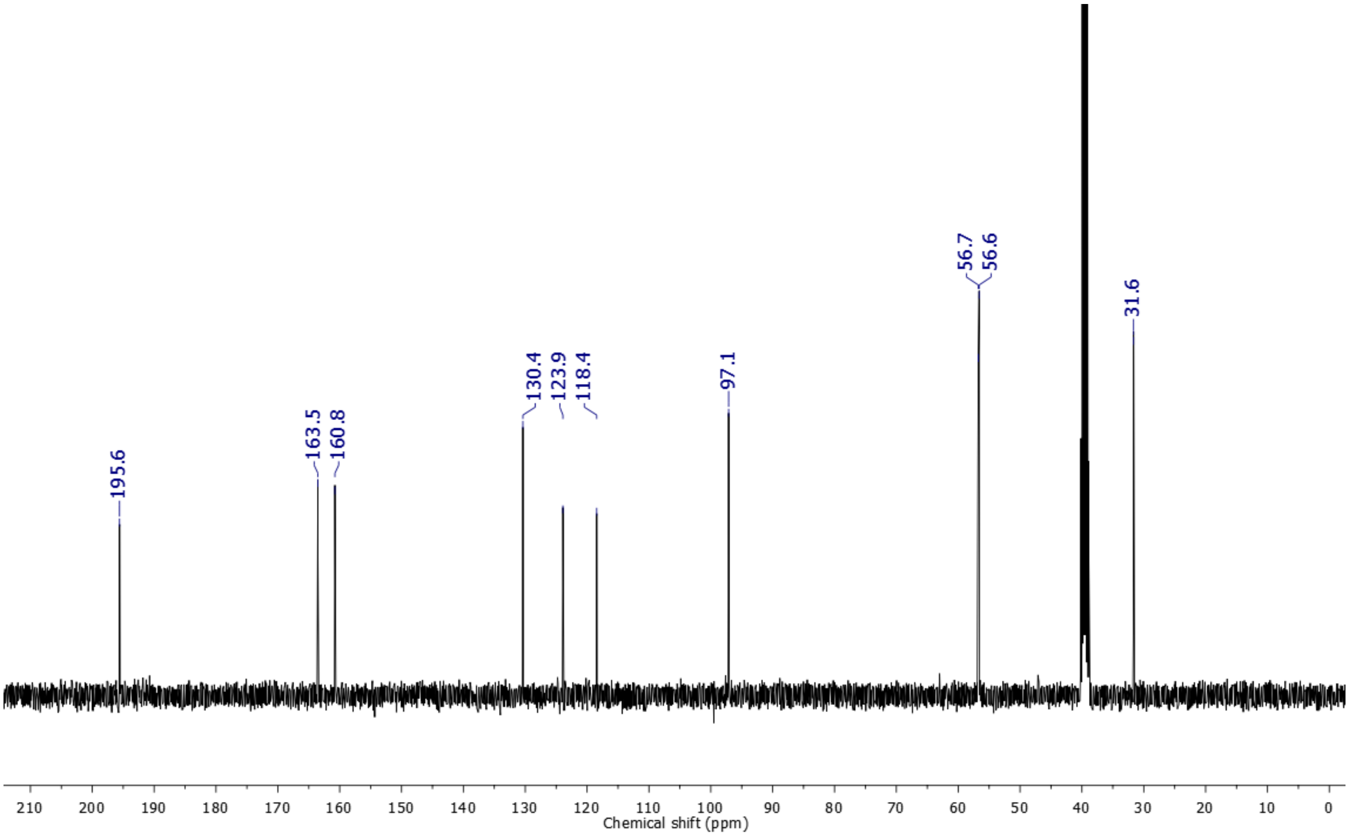


$^{13}$C NMR (101 MHz, DMSO-*d6*) spectrum of **8b**.

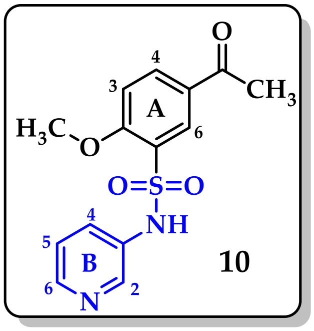


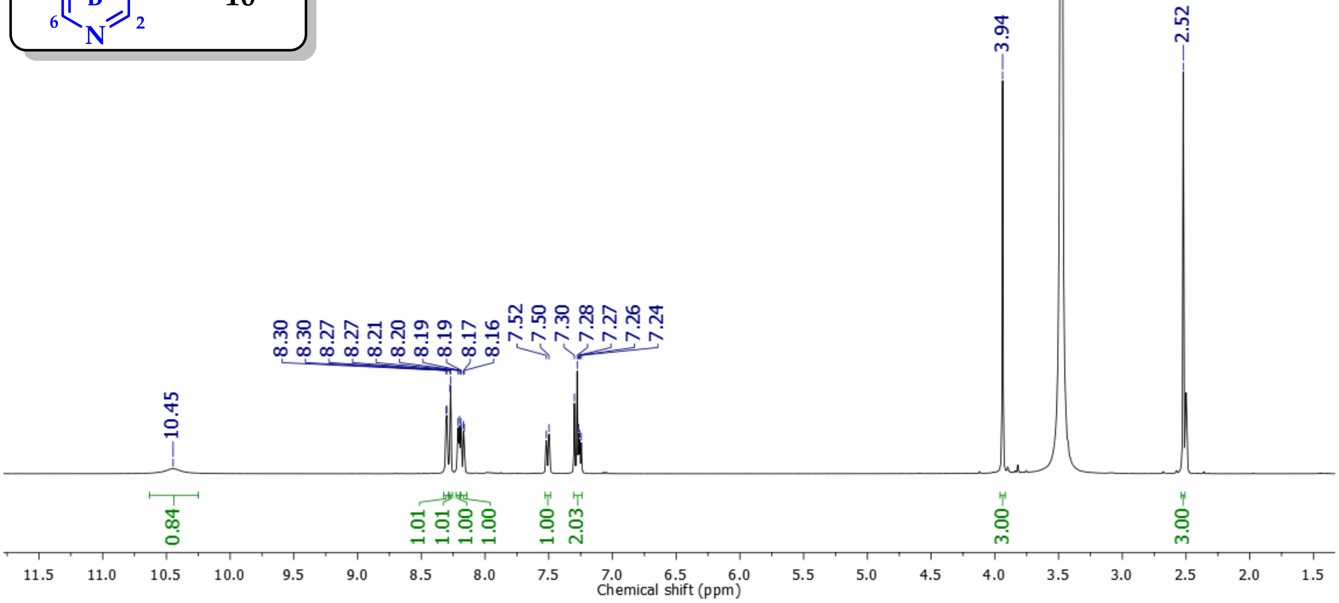


$^{1}$H NMR (400 MHz, DMSO-*d*$_6$) spectrum of **10**.

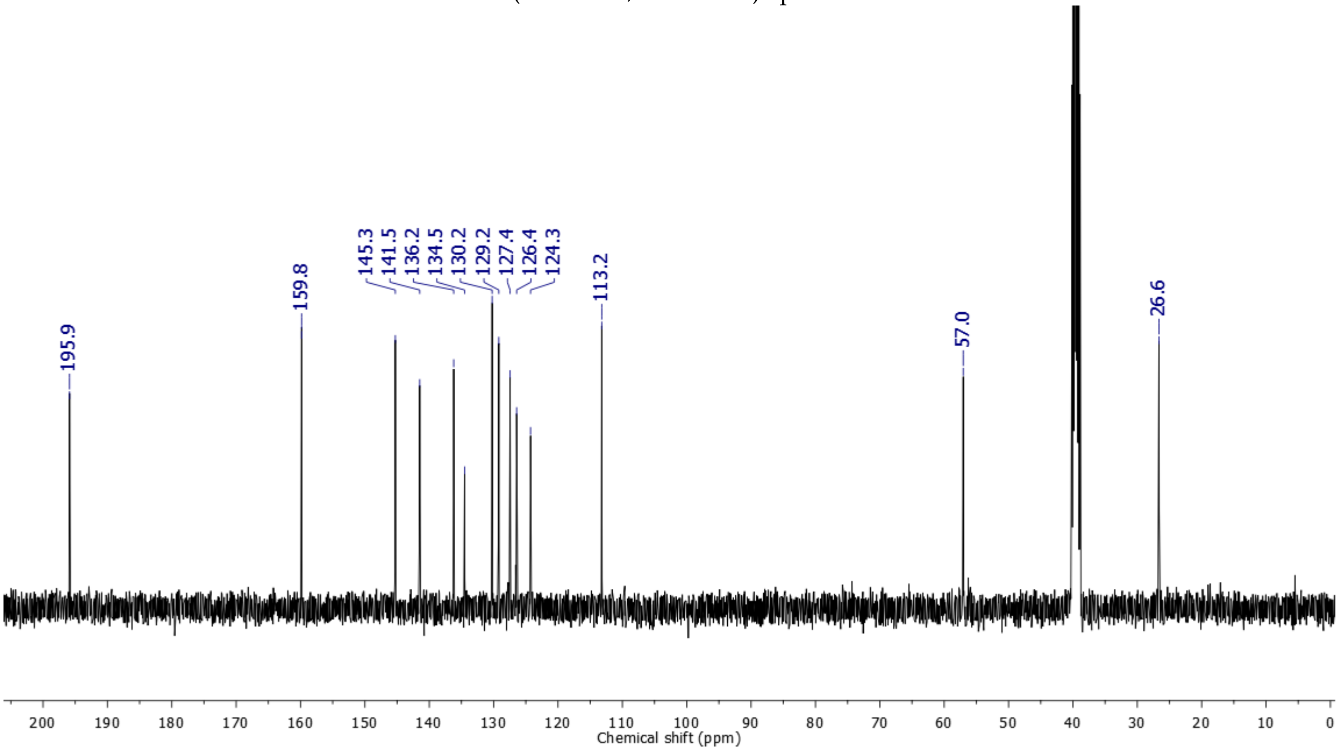


$^{13}$C NMR (101 MHz, DMSO-*d*$_6$) spectrum of **10**.

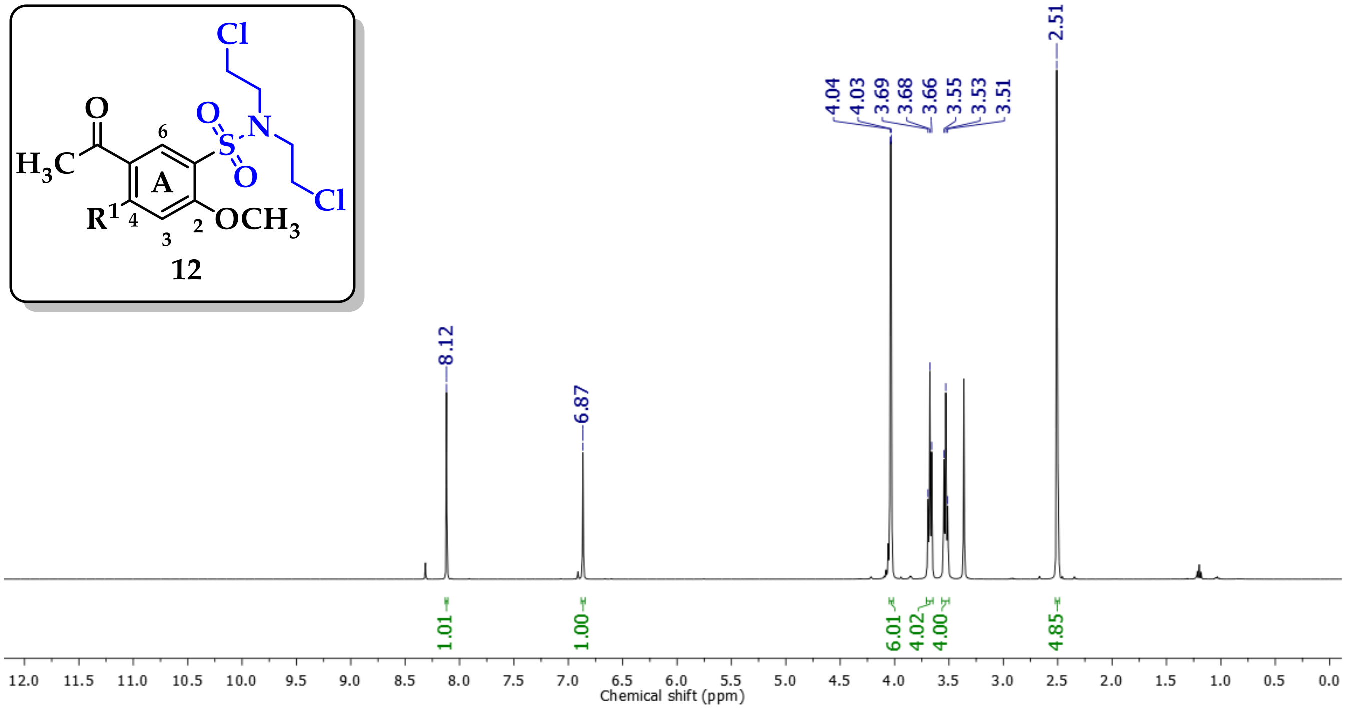


$^1$H NMR (400 MHz, DMSO-$d_6$) spectrum of **12**.

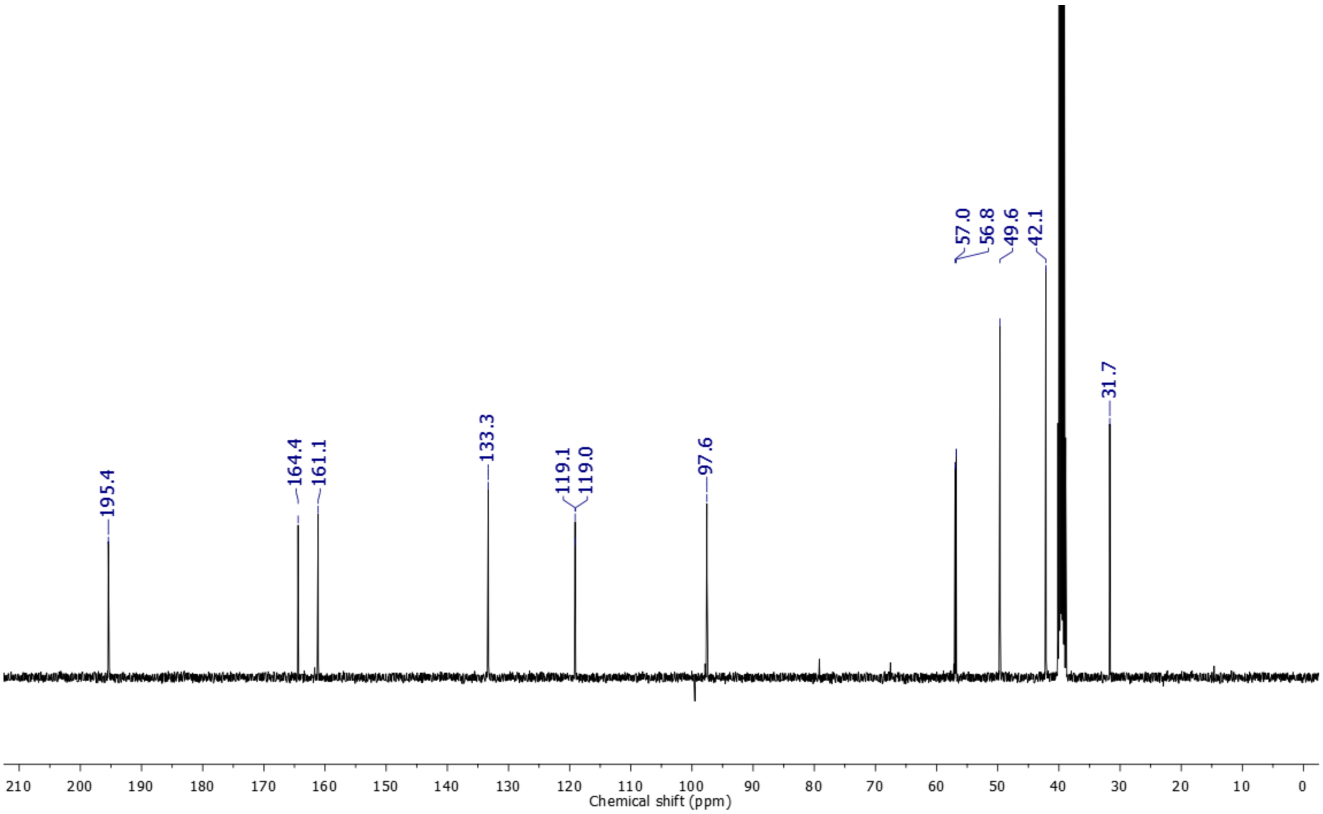


$^{13}$C NMR (101 MHz, DMSO-$d_6$) spectrum of **12**.

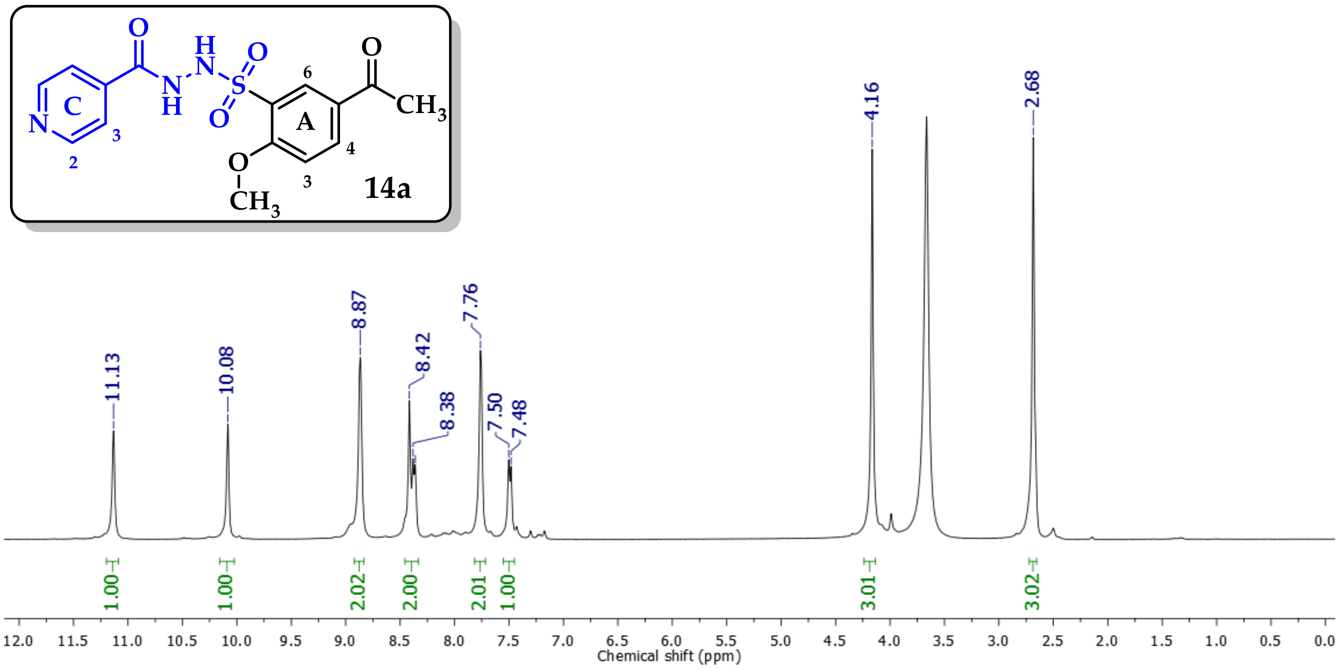


$^{1}H$ NMR (400 MHz, DMSO-$d_6$) spectrum of **14a**.

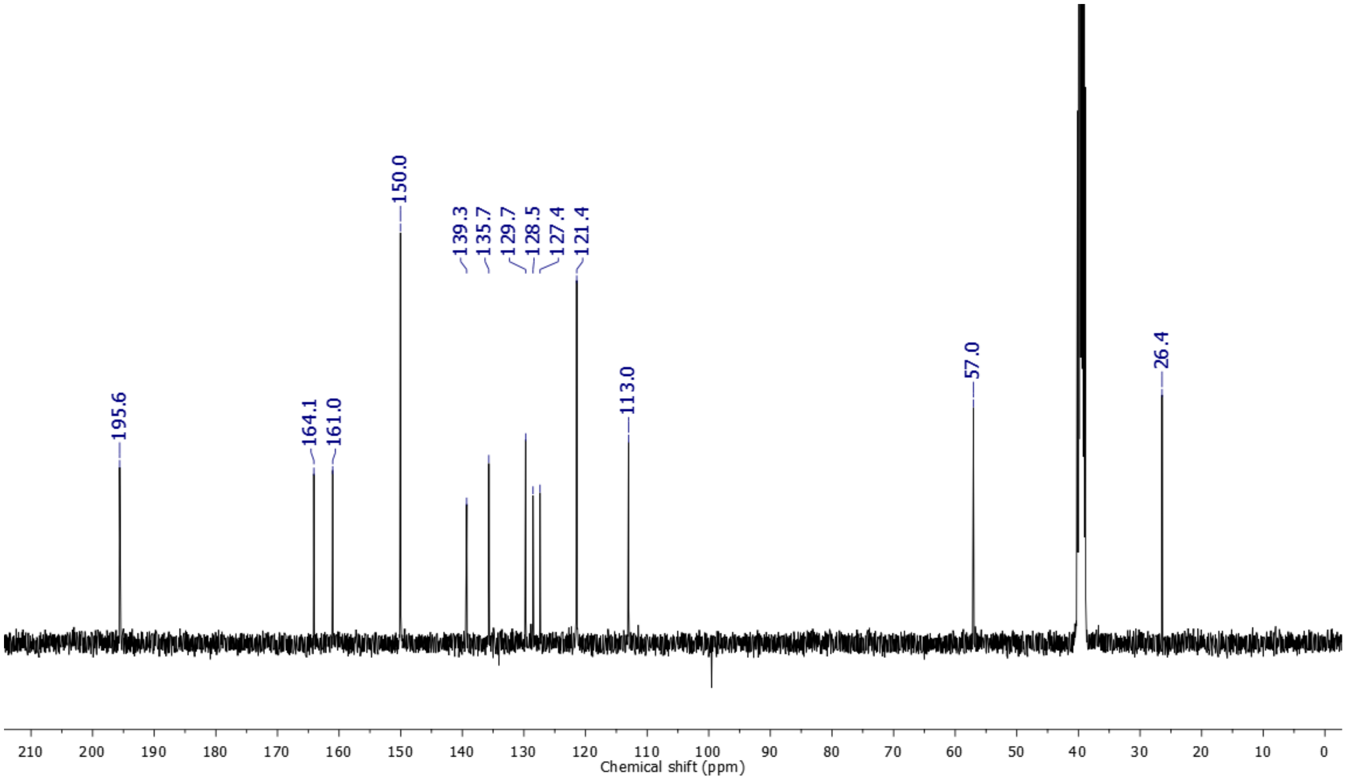


$^{13}C$ NMR (101 MHz, DMSO-$d_6$) spectrum of **14a**.

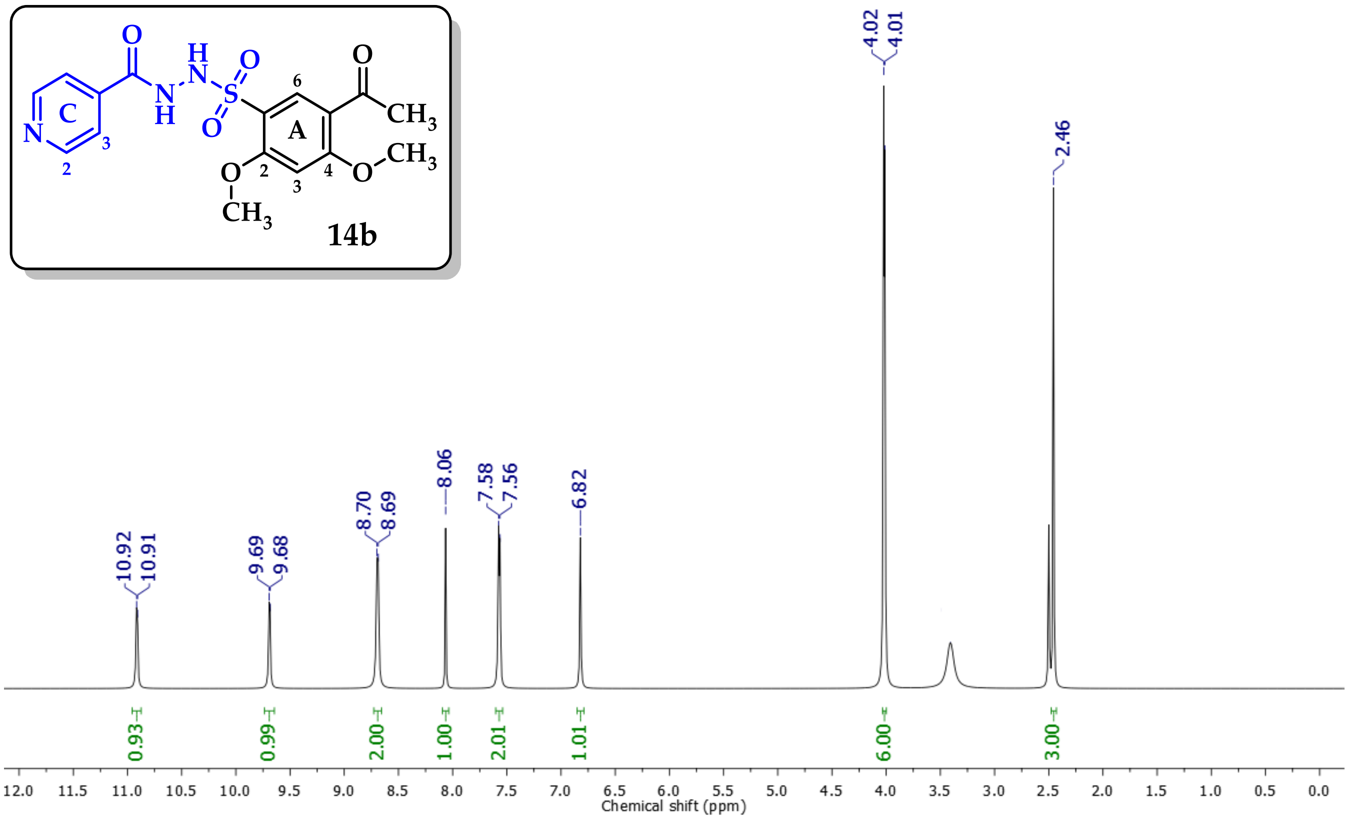


$^{1}$H NMR (400 MHz, DMSO-$d_6$) spectrum of **14b**.

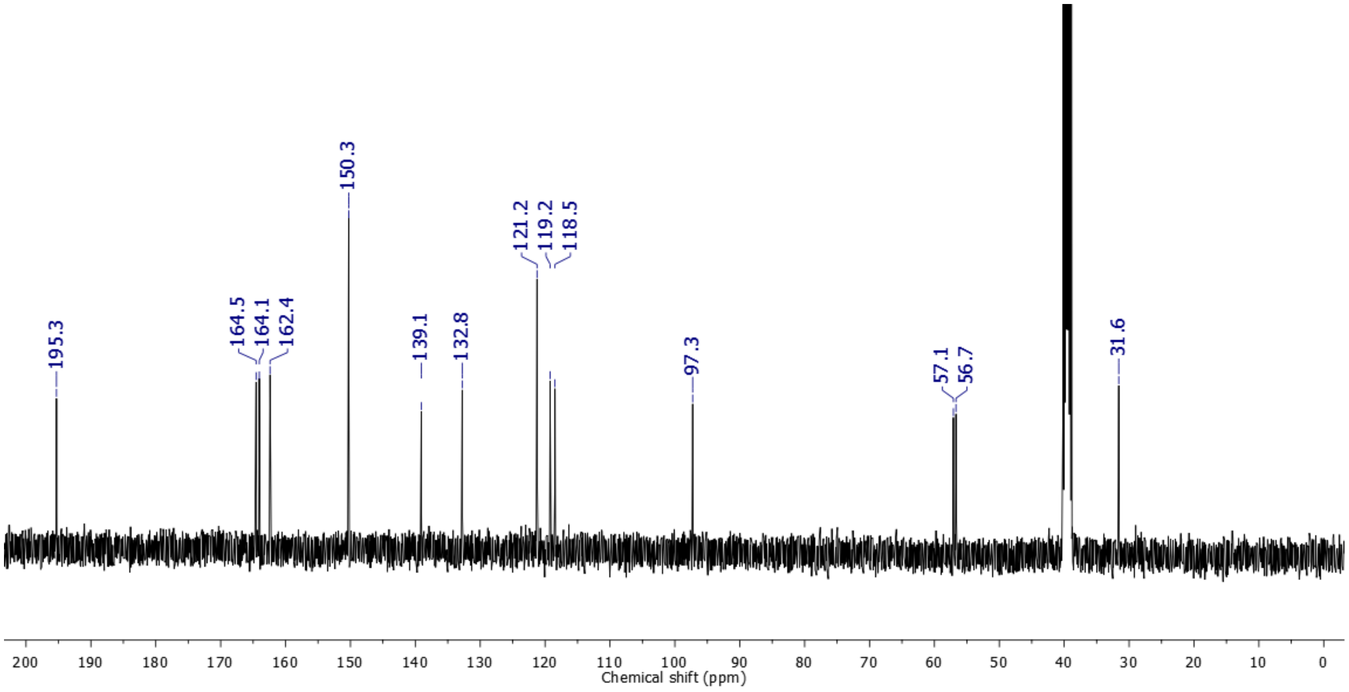


$^{13}$C NMR (101 MHz, DMSO-$d_6$) spectrum of **14b**.

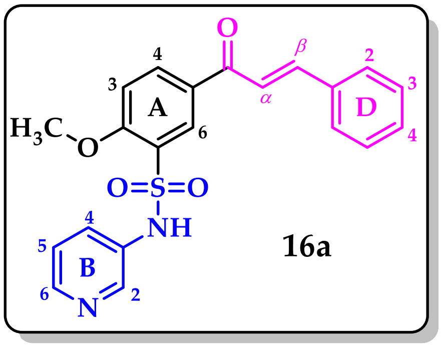


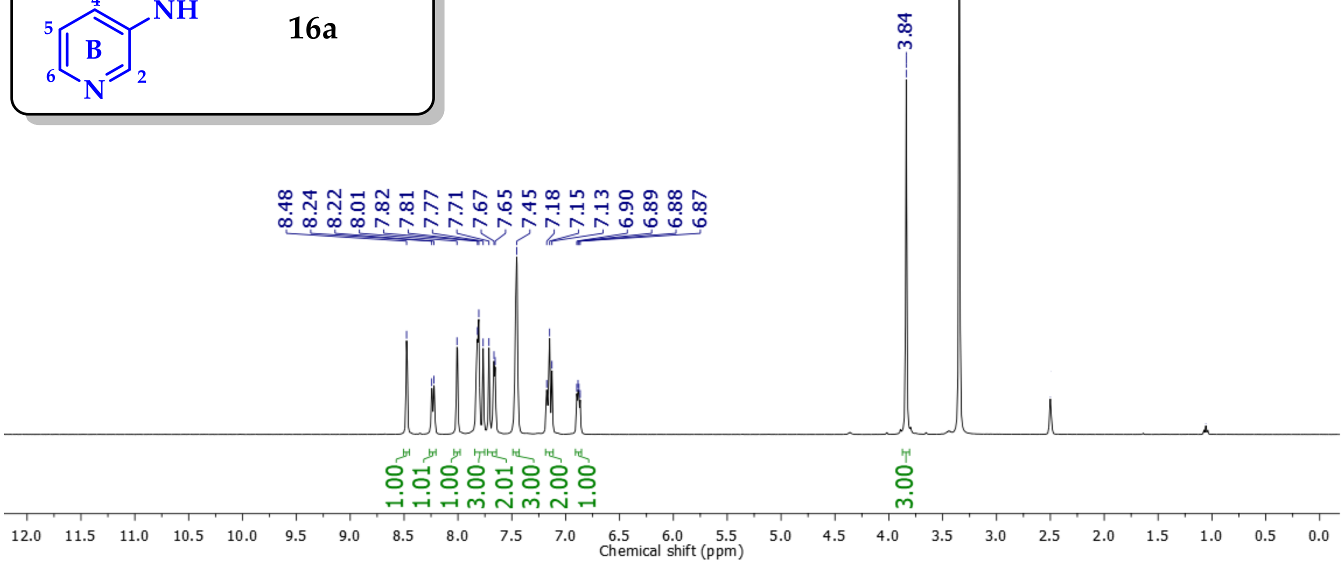


$^{1}$H NMR (400 MHz, DMSO-*d*$_6$) spectrum of **16a**.

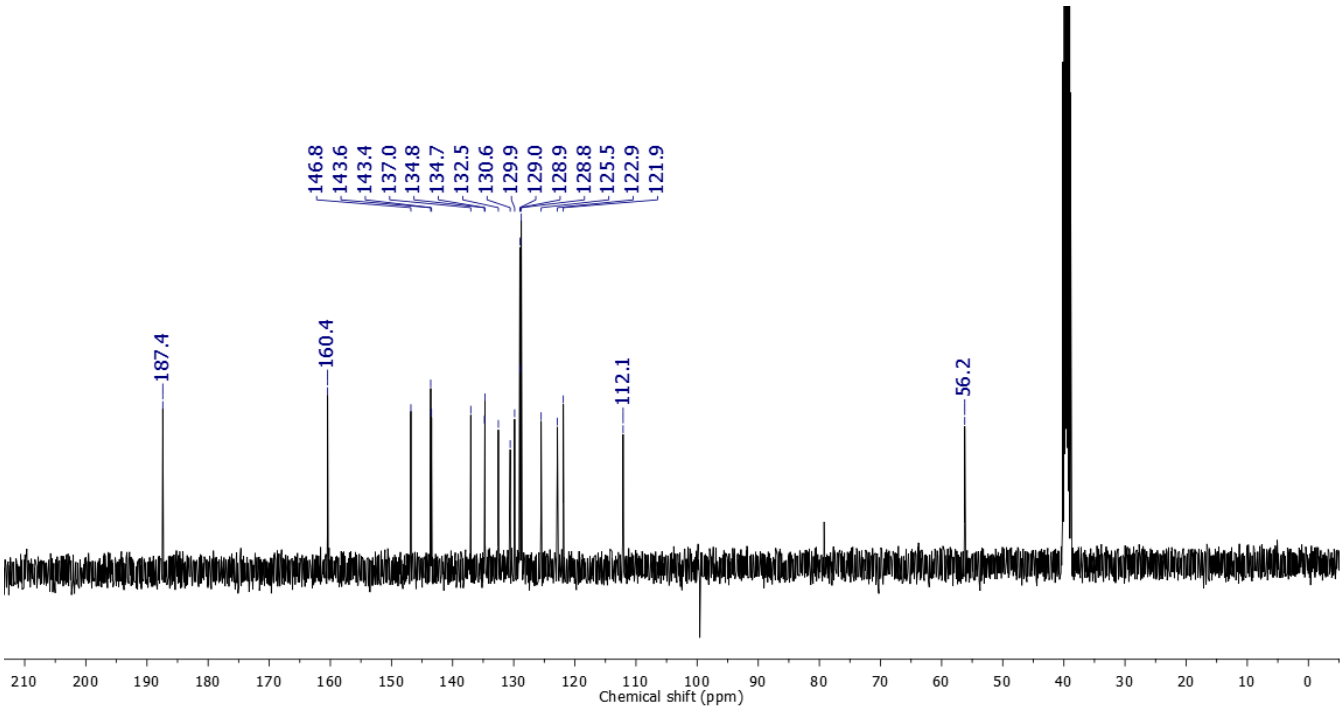


$^{13}$C NMR (101 MHz, DMSO-*d*$_6$) spectrum of **16a**.

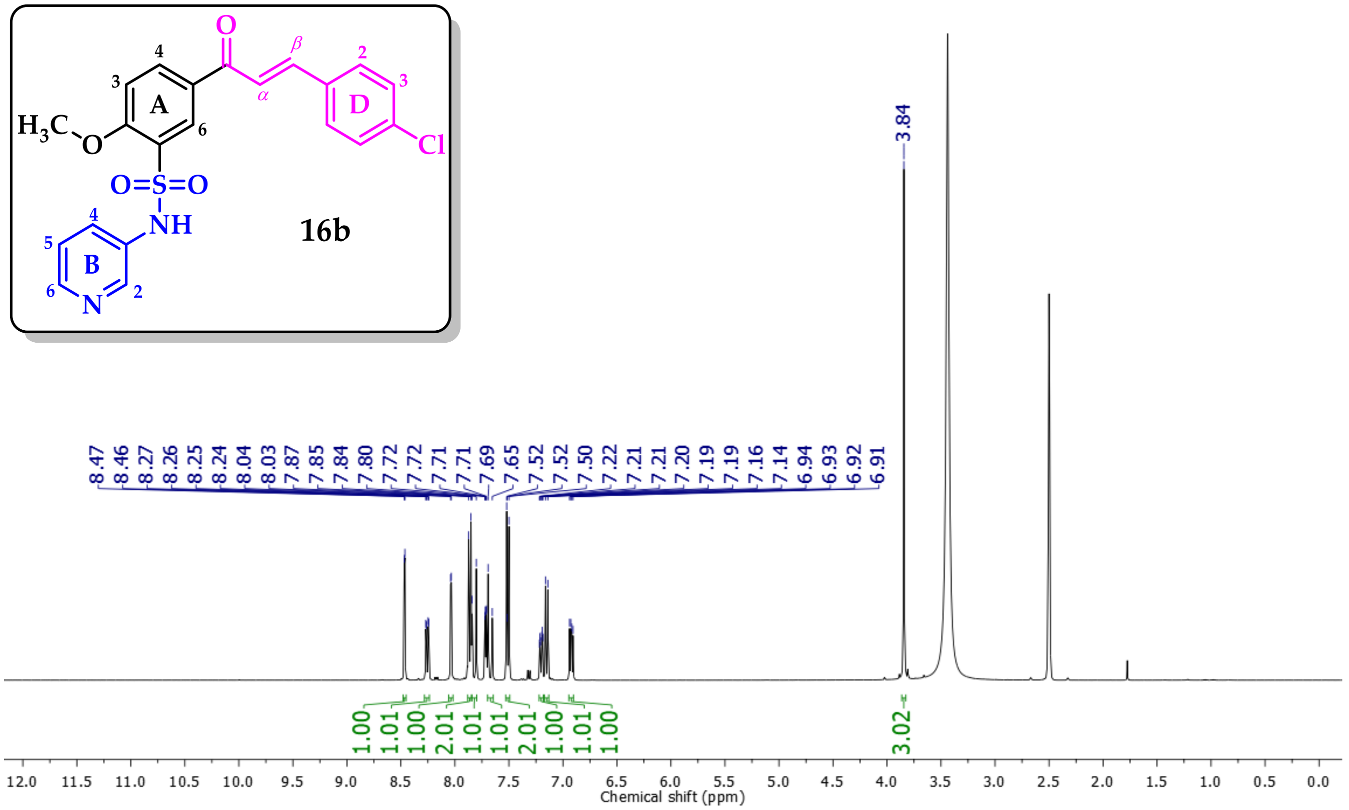


$^{1}$H NMR (400 MHz, DMSO-*d*$_6$) spectrum of **16b**.

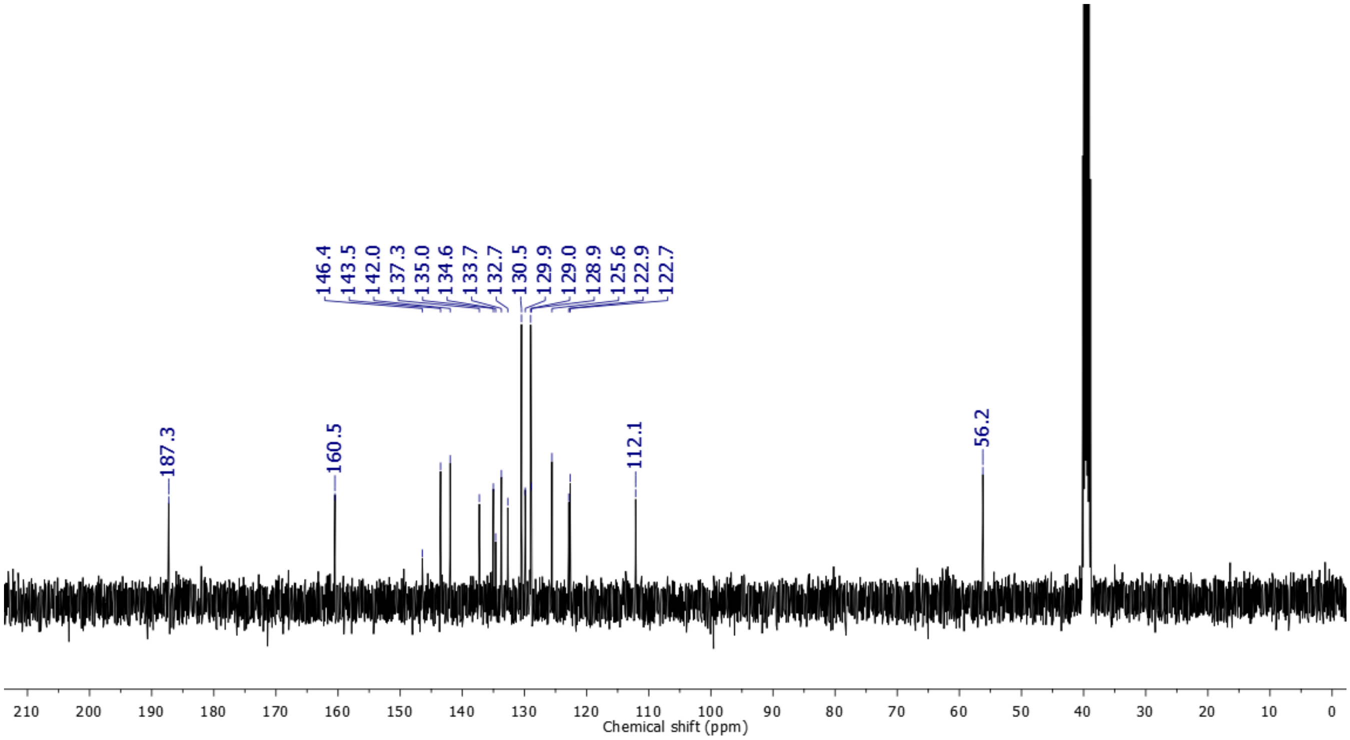


$^{13}$C NMR (101 MHz, DMSO-*d*$_6$) spectrum of **16b**.

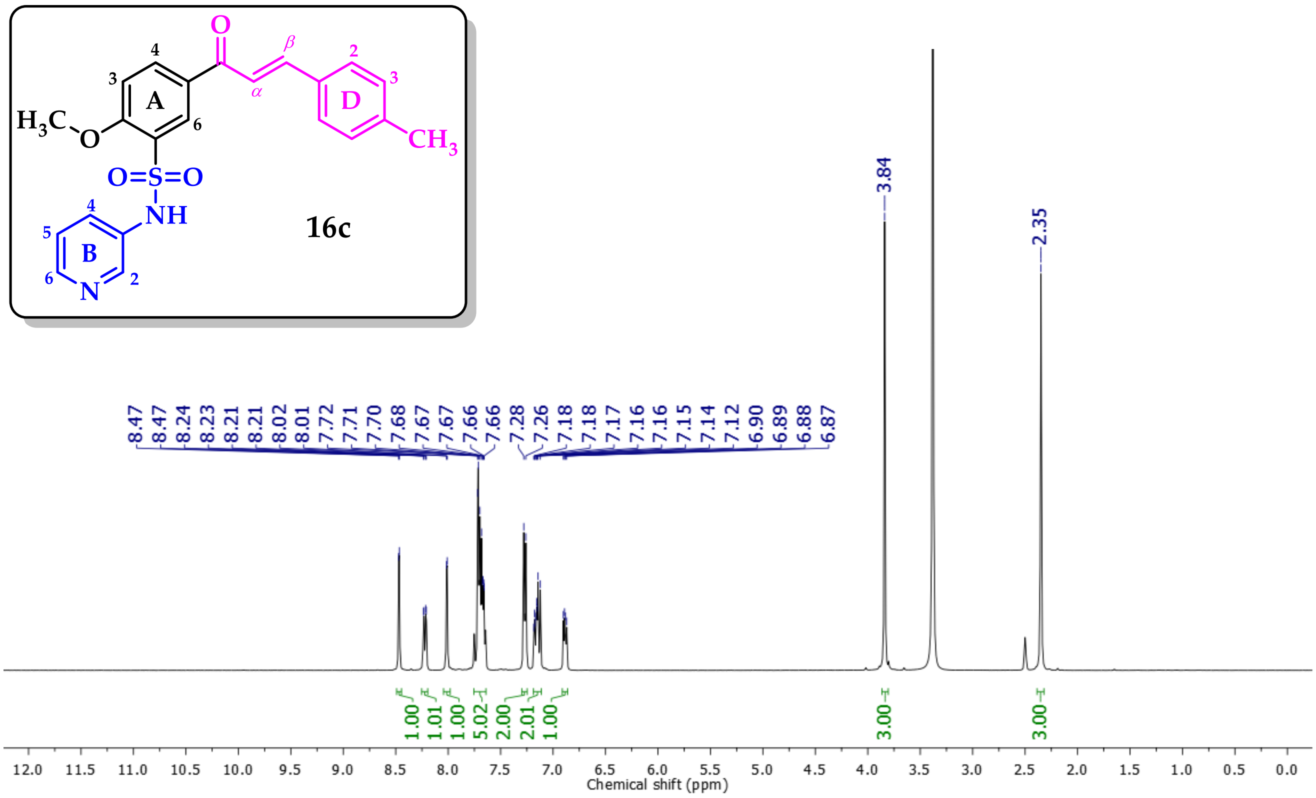


$^{1}H$ NMR (400 MHz, DMSO-$d_6$) spectrum of **16c**.

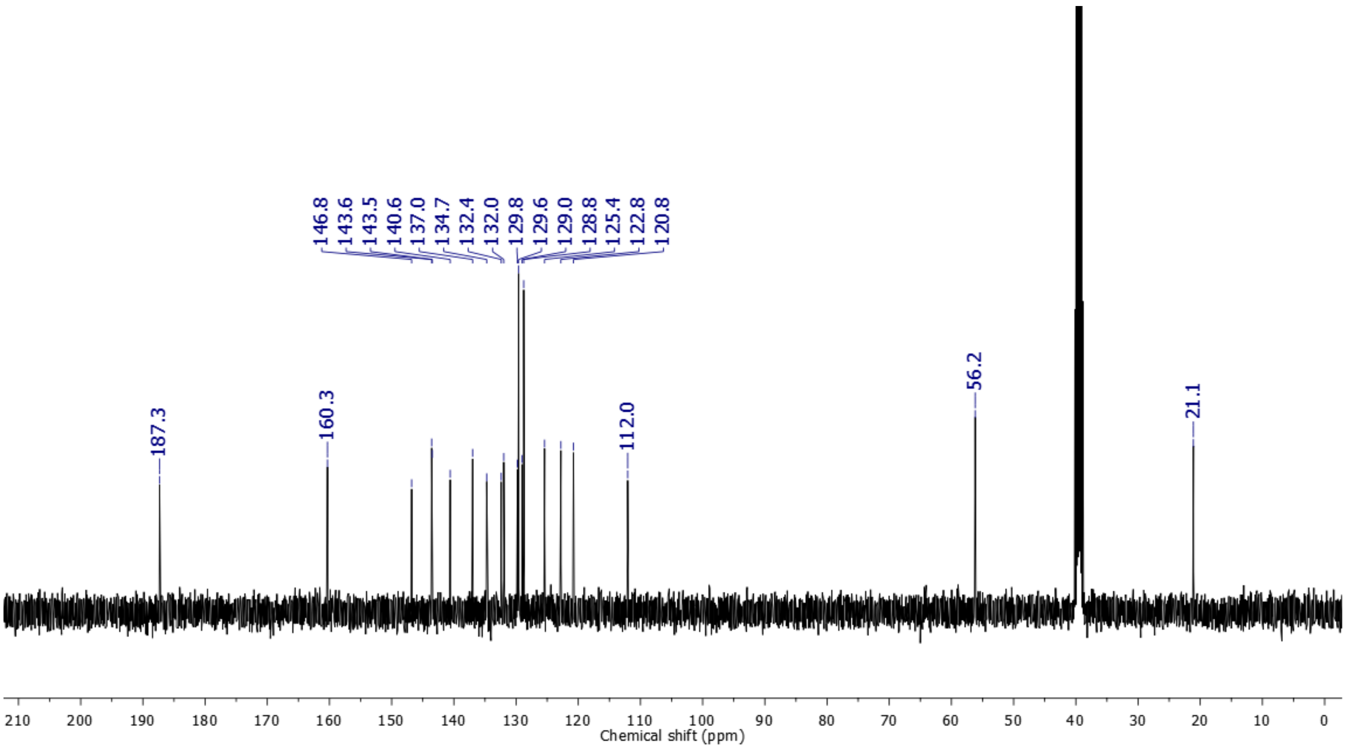


$^{13}C$ NMR (101 MHz, DMSO-$d_6$) spectrum of **16c**.

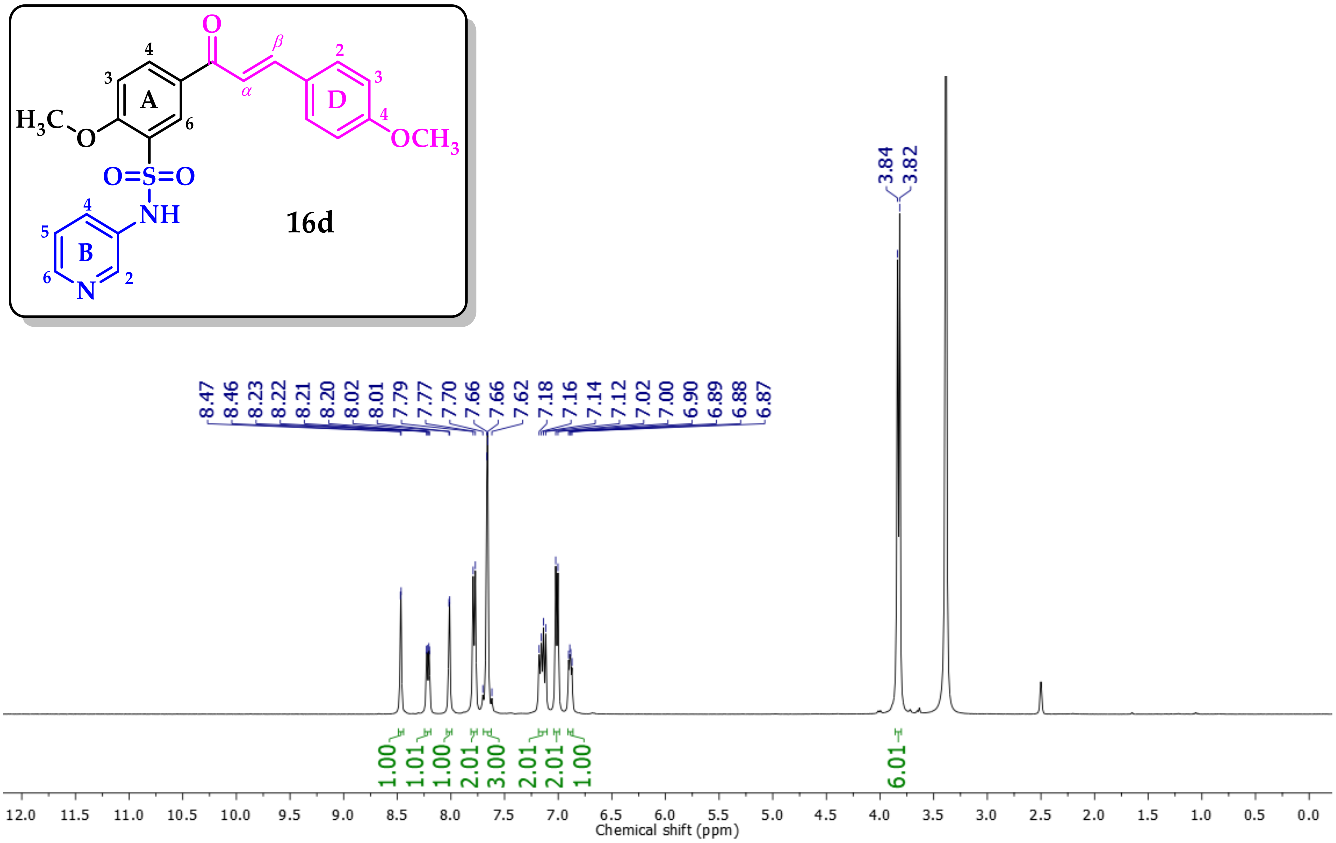


$^{1}H$ NMR (400 MHz, DMSO-$d_6$) spectrum of **16d**.

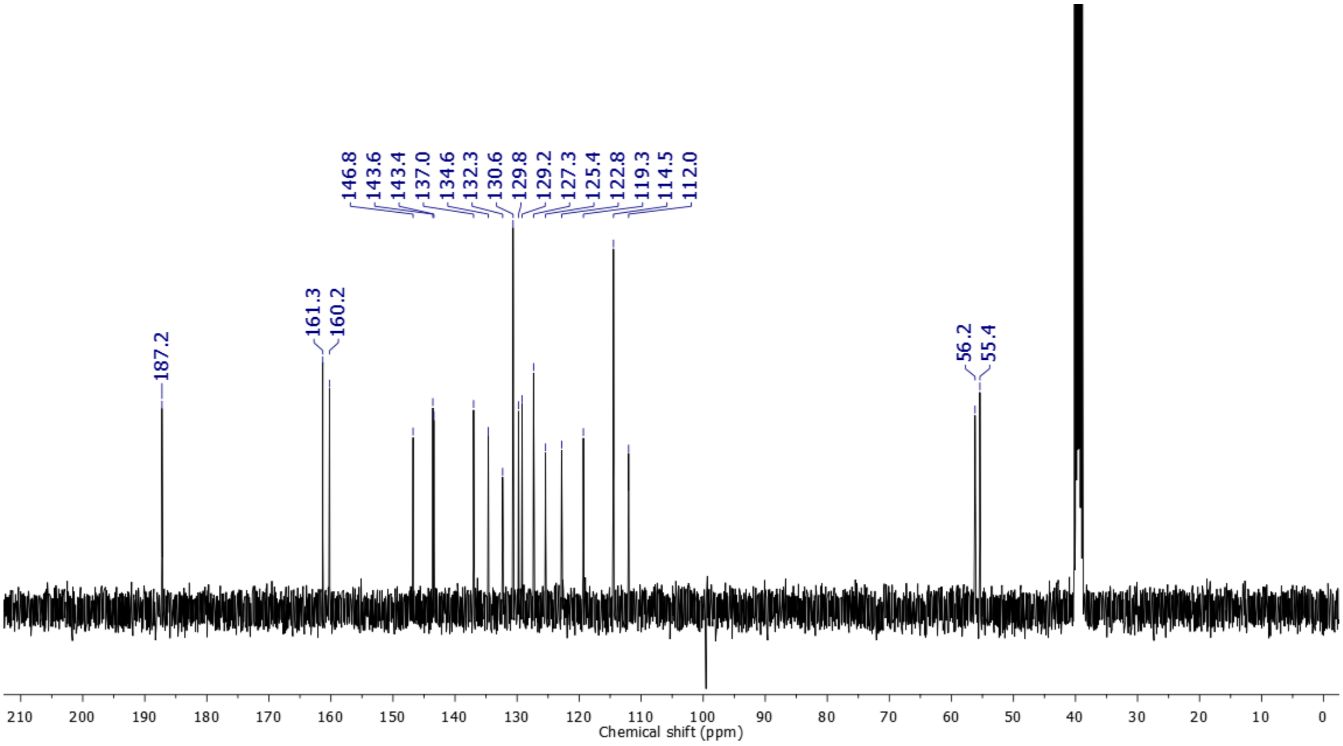


$^{13}C$ NMR (101 MHz, DMSO-$d_6$) spectrum of **16d**.

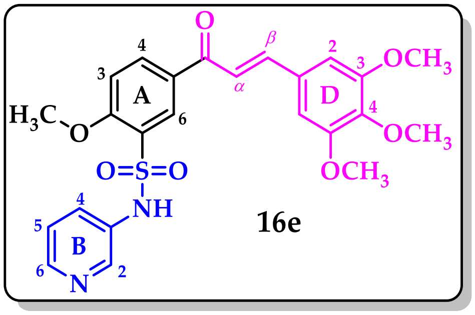


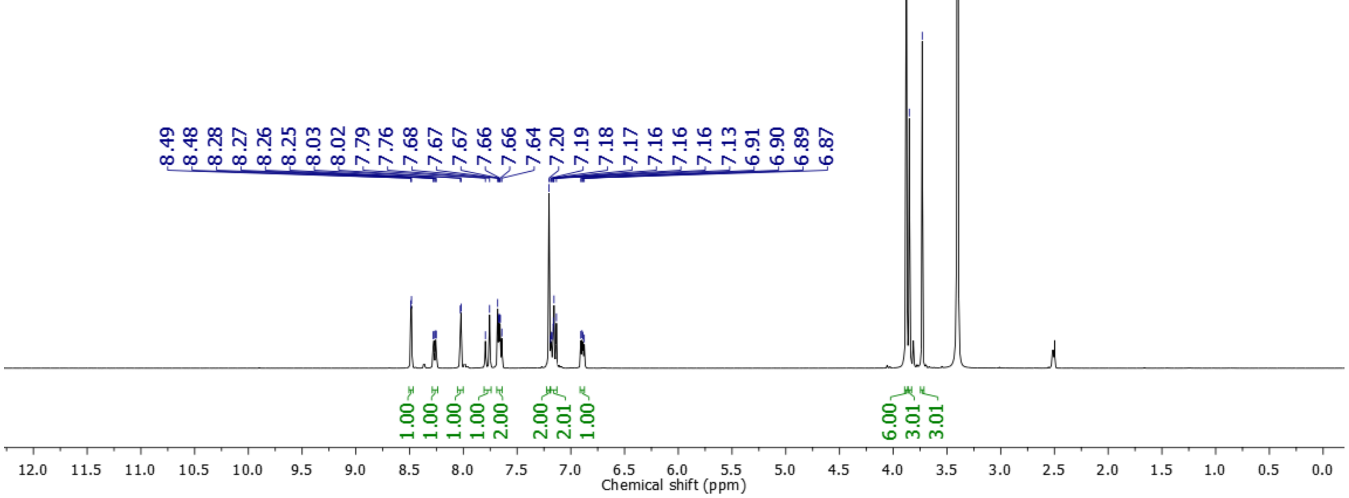


$^{1}$H NMR (400 MHz, DMSO-*d6*) spectrum of **16e**.

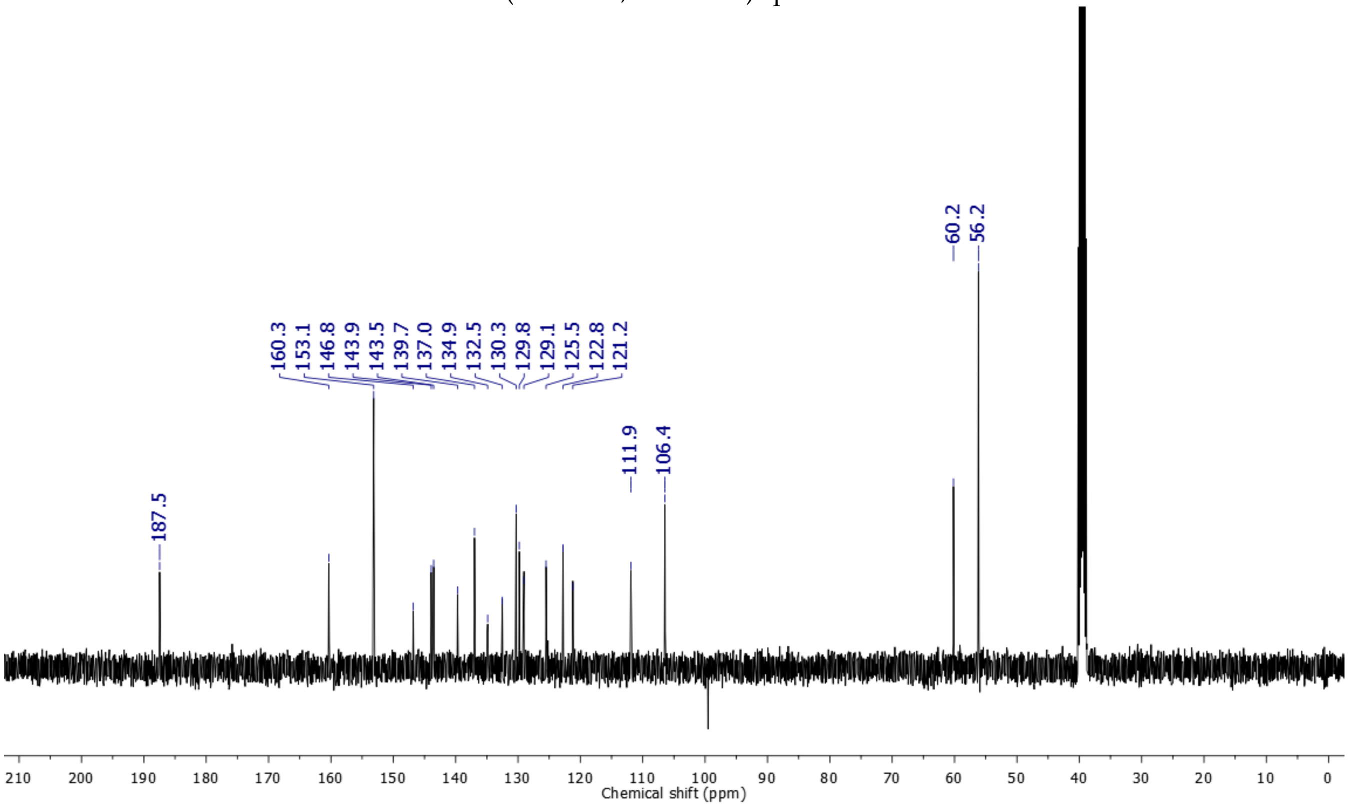


$^{13}$C NMR (101 MHz, DMSO-*d6*) spectrum of **16e**.

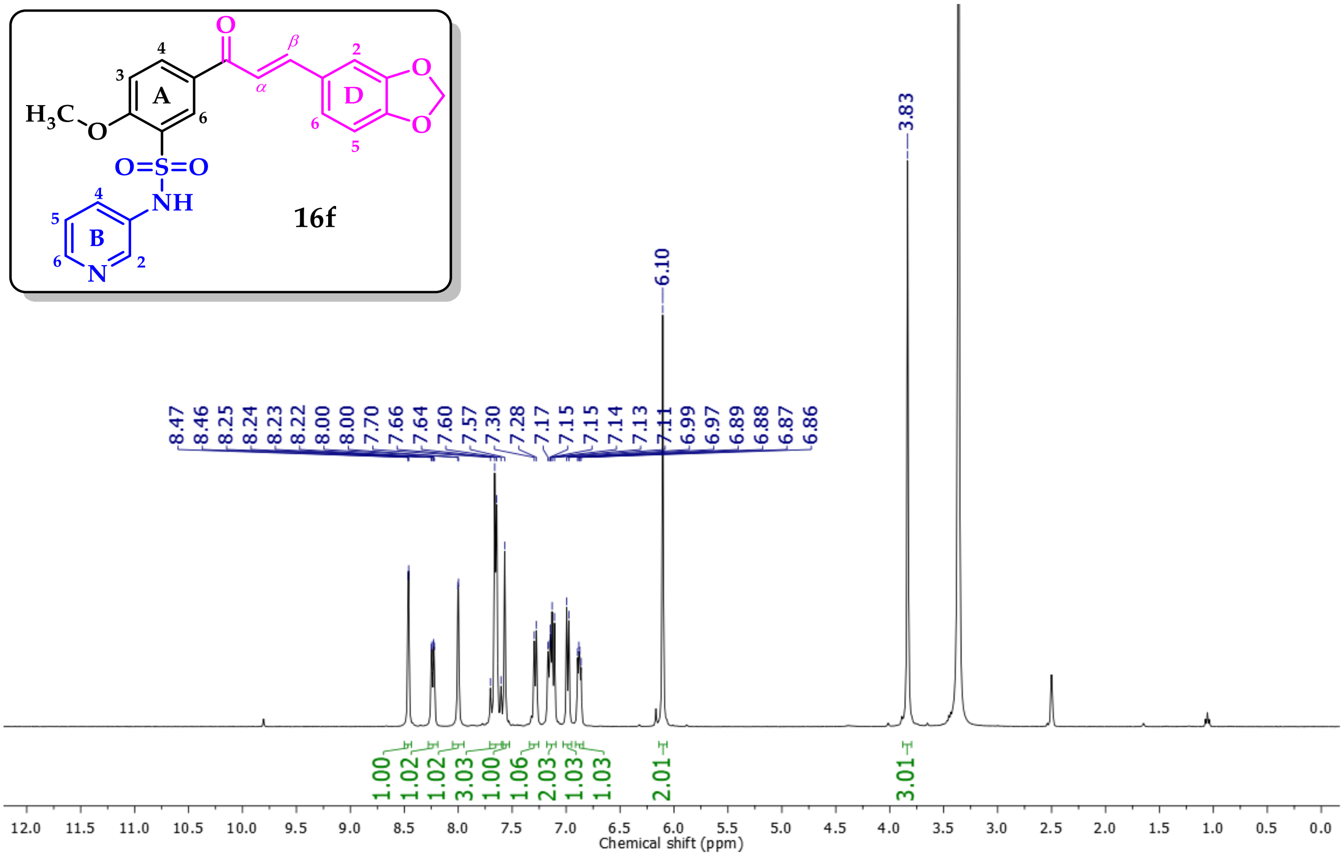


$^{1}$H NMR (400 MHz, DMSO-*d*6) spectrum of **16f**.

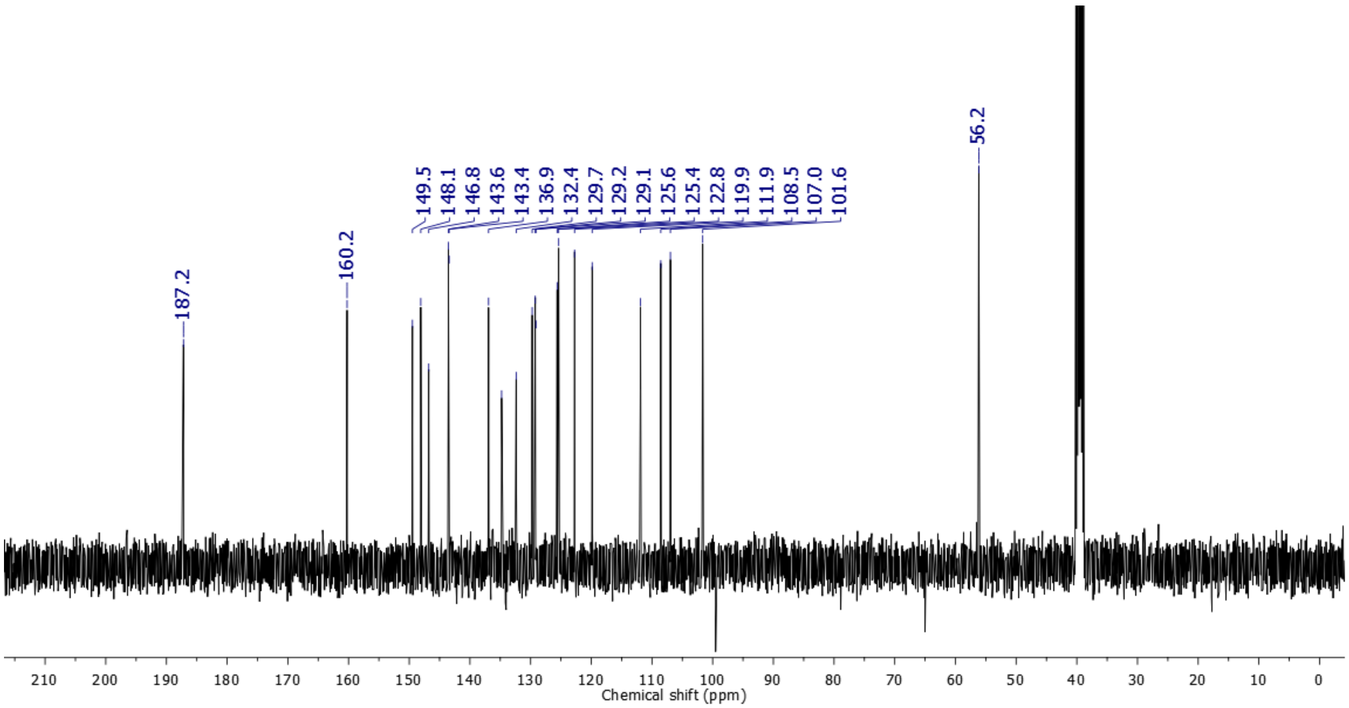


$^{13}$C NMR (101 MHz, DMSO-*d*6) spectrum of **16f**.

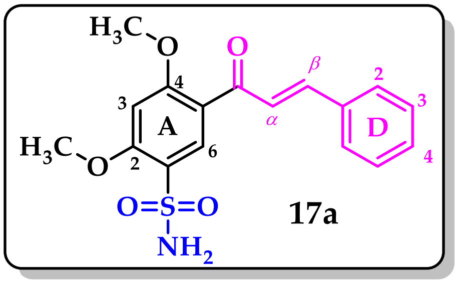


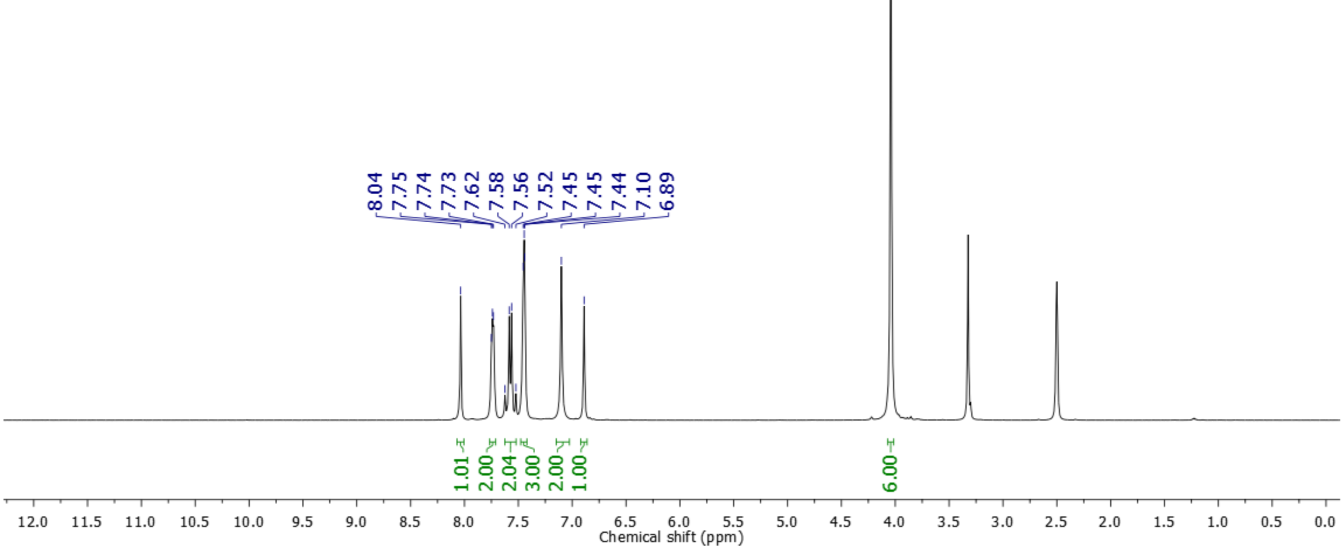


$^1H$ NMR (400 MHz, DMSO-$d_6$) spectrum of **17a**.

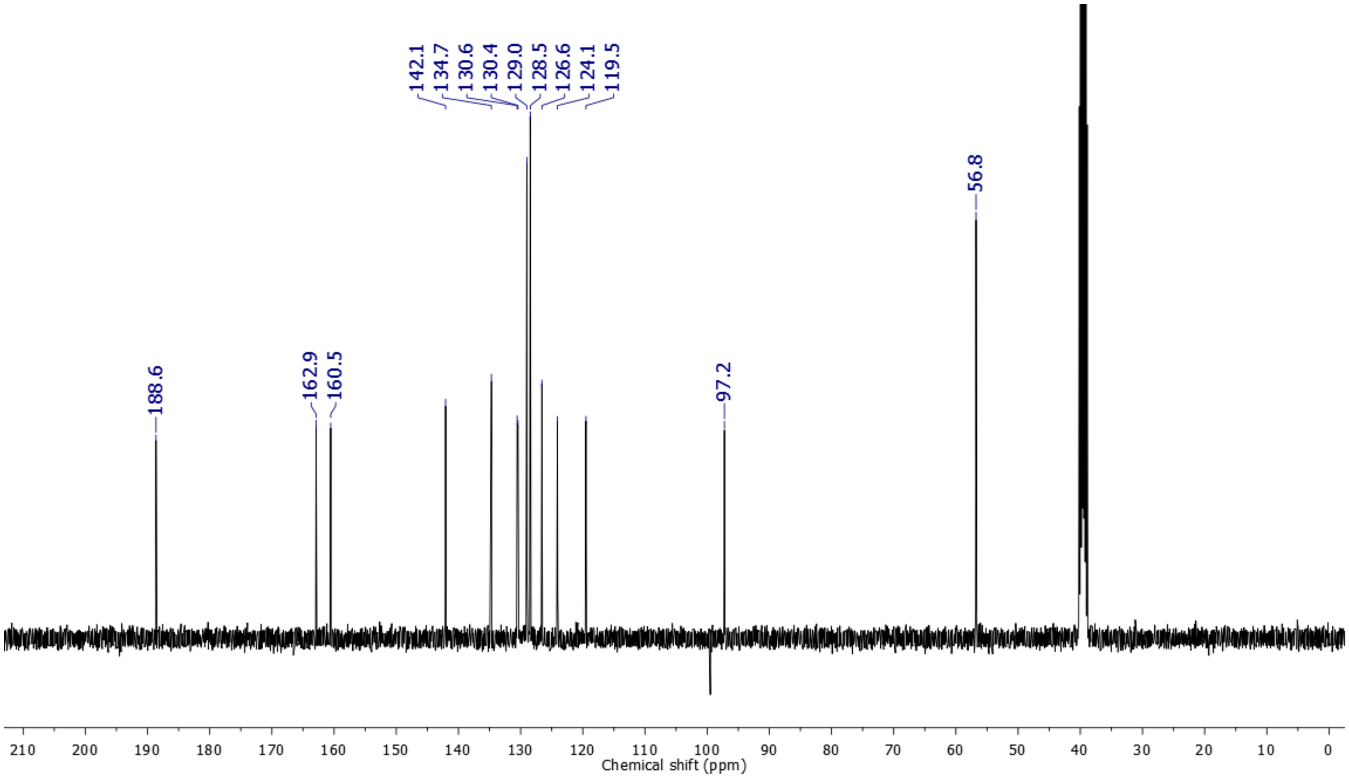


$^{13}C$ NMR (101 MHz, DMSO-$d_6$) spectrum of **17a**.

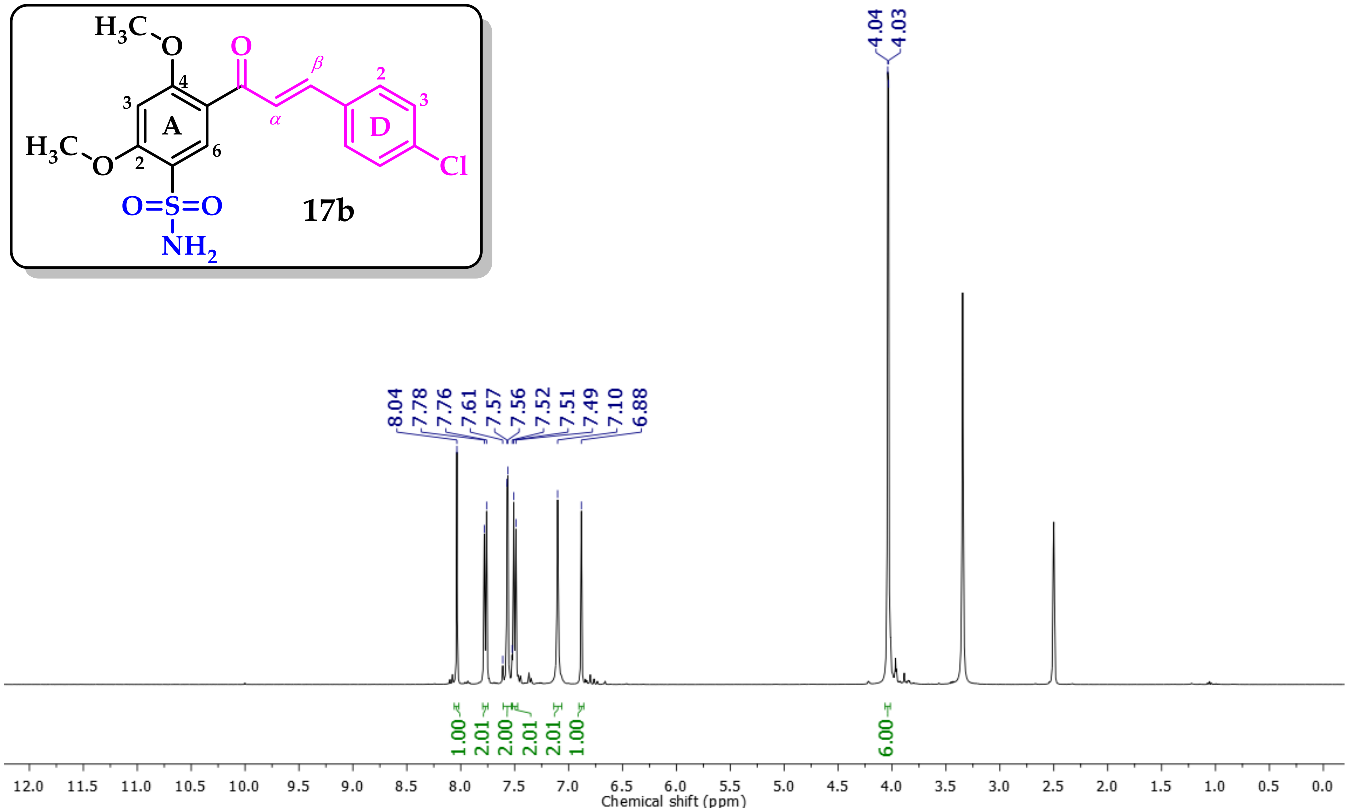


$^{1}$H NMR (400 MHz, DMSO-*d*6) spectrum of **17b**.

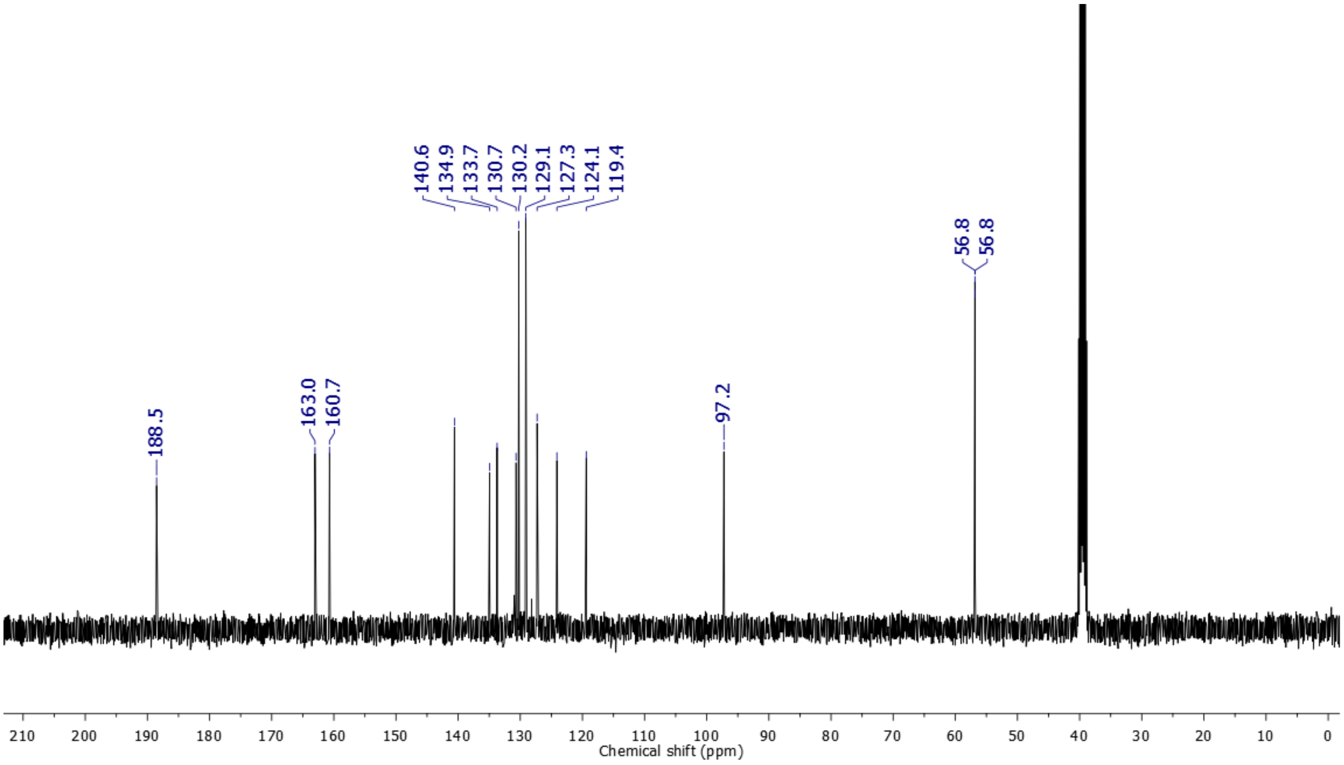


$^{13}$C NMR (101 MHz, DMSO-*d*6) spectrum of **17b**.

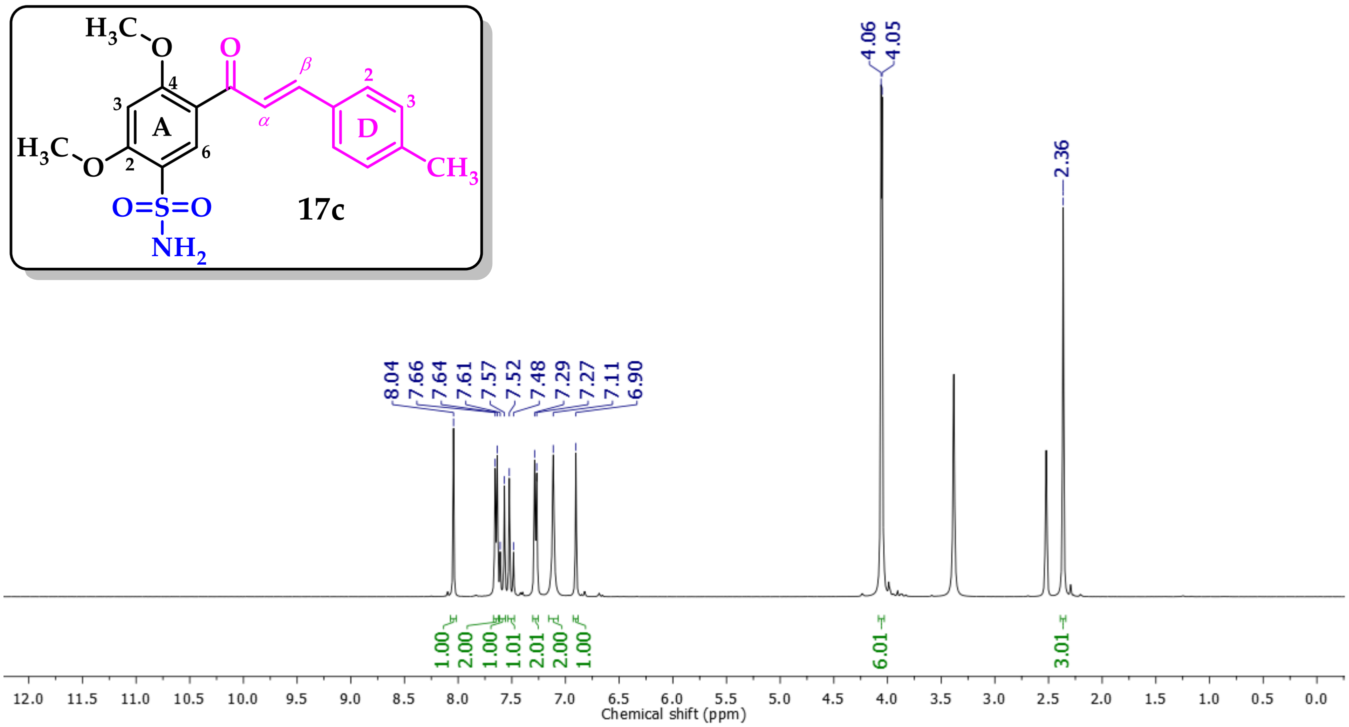


$^{1}$H NMR (400 MHz, DMSO-*d6*) spectrum of **17c**.

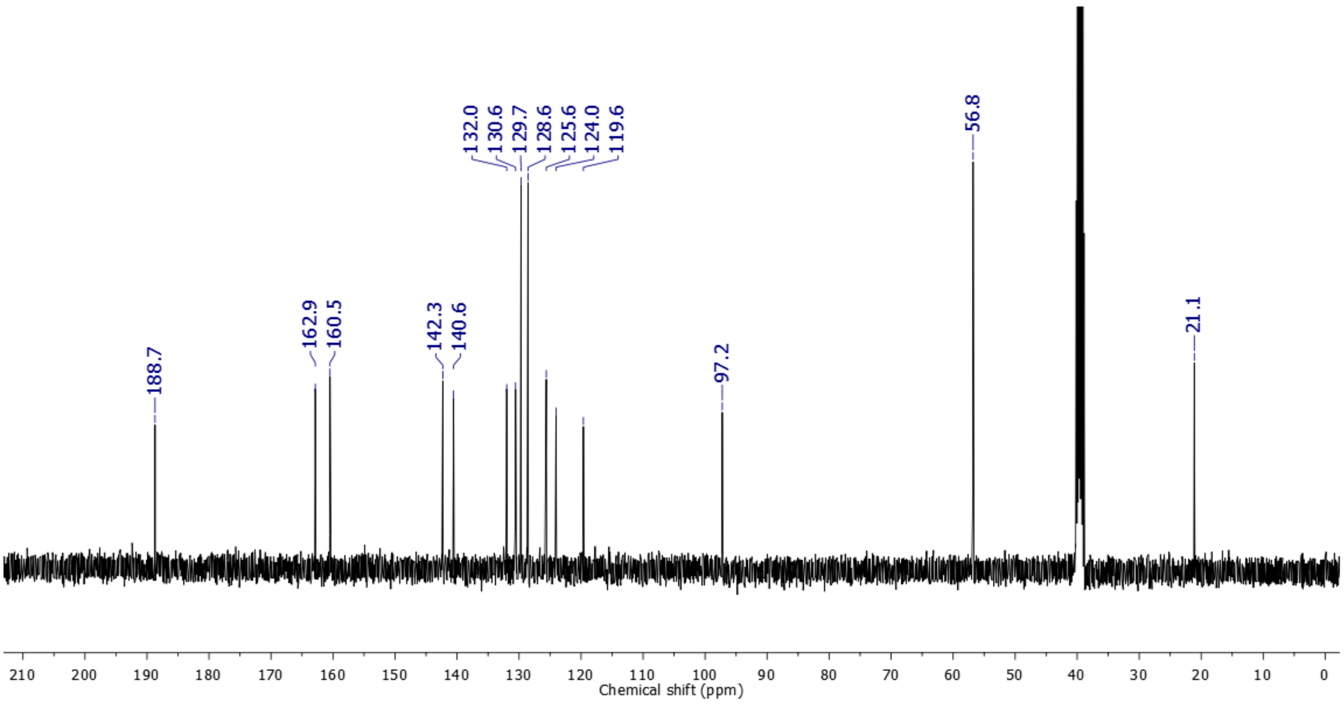


$^{13}$C NMR (101 MHz, DMSO-*d6*) spectrum of **17c**.

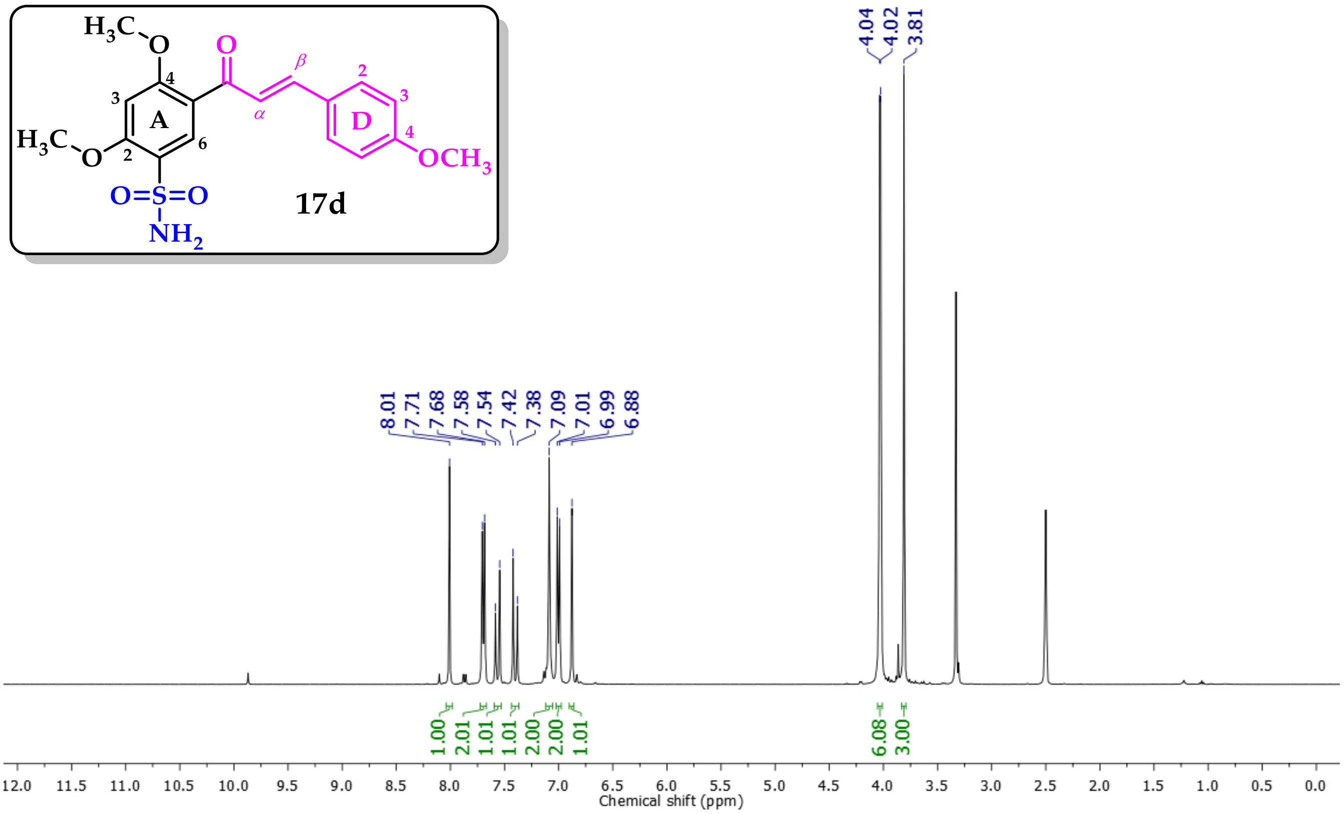


$^{1}H$ NMR (400 MHz, DMSO-$d_6$) spectrum of **17d**.

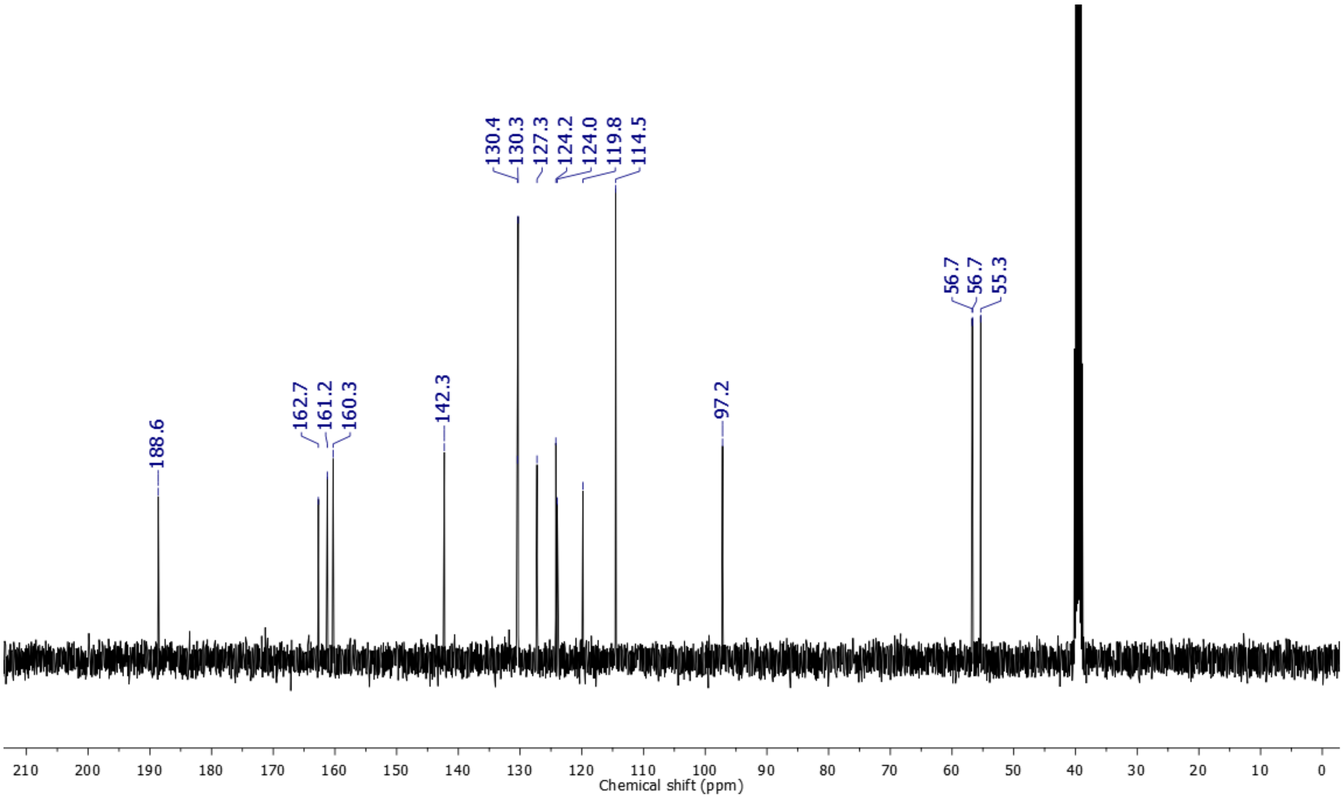


$^{13}C$ NMR (101 MHz, DMSO-$d_6$) spectrum of **17d**.

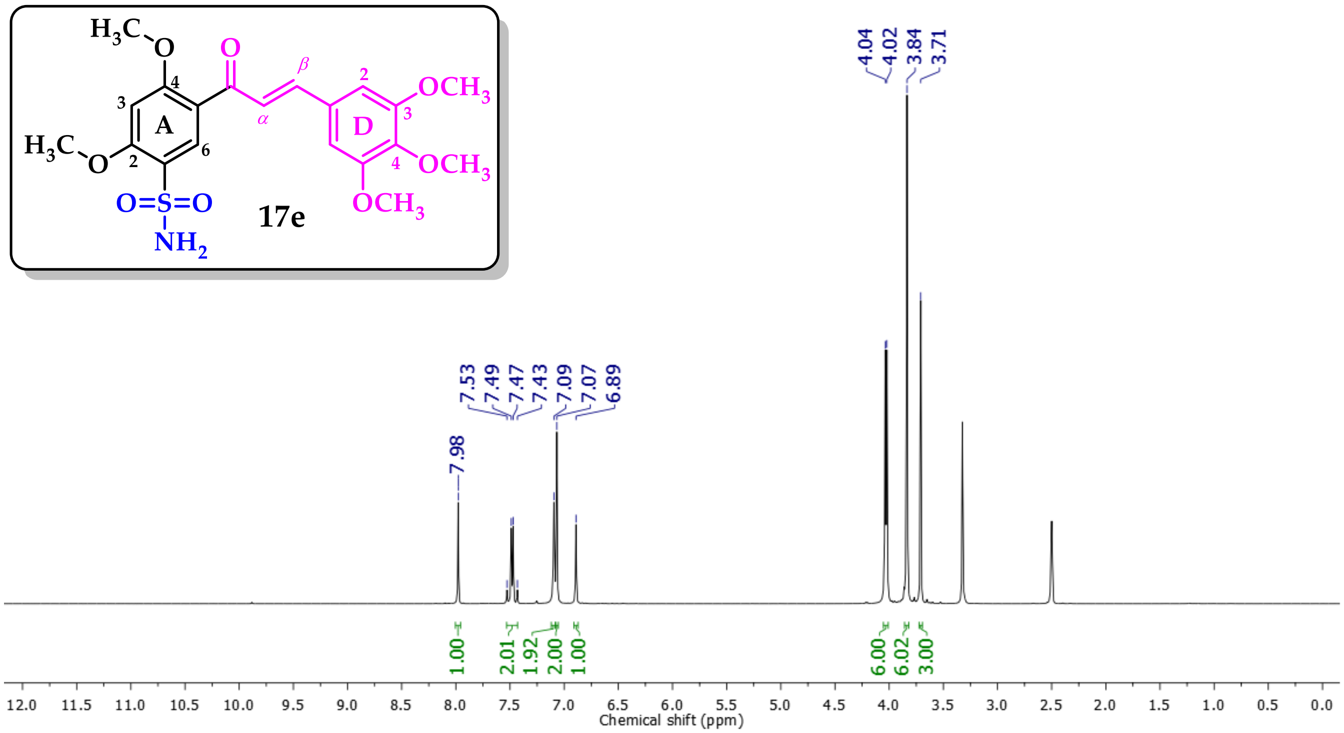


$^{1}H$ NMR (400 MHz, DMSO-$d_6$) spectrum of **17e**.

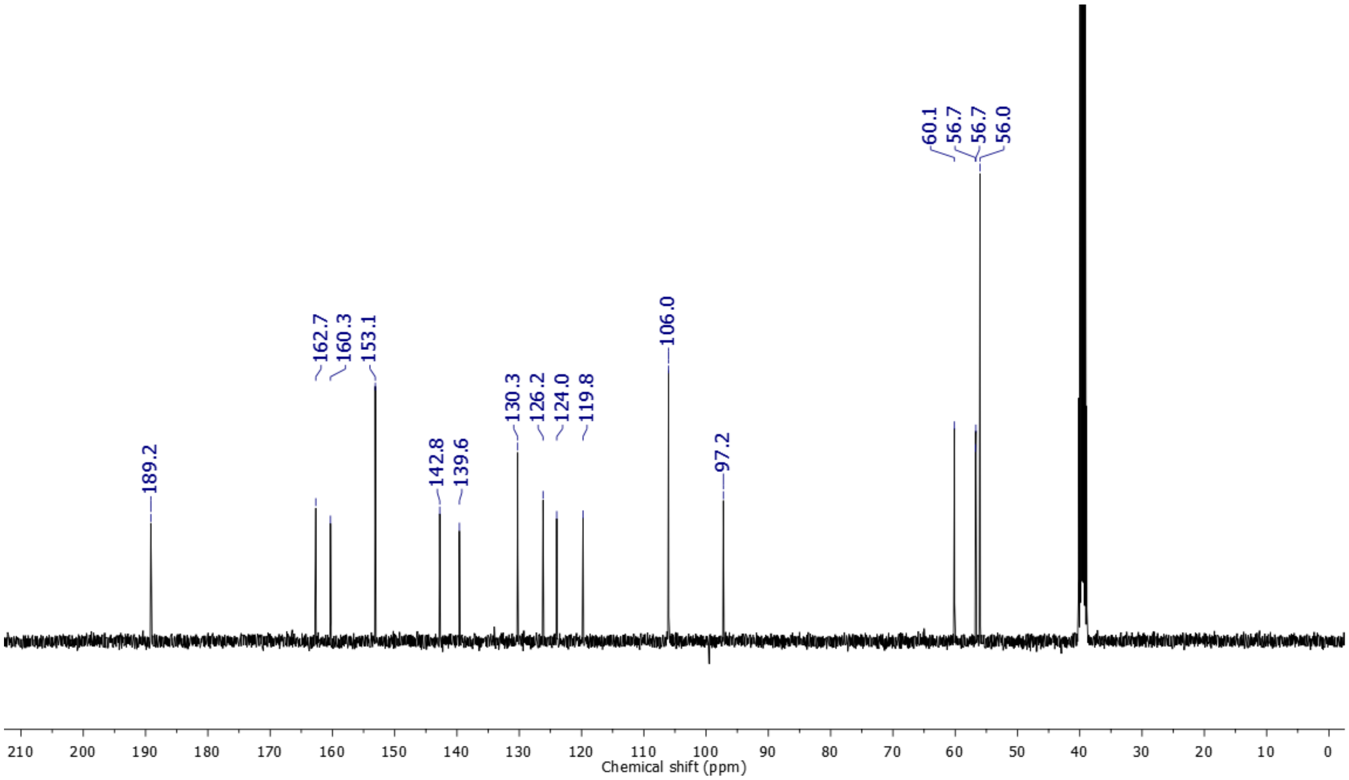


$^{13}C$ NMR (101 MHz, DMSO-$d_6$) spectrum of **17e**.

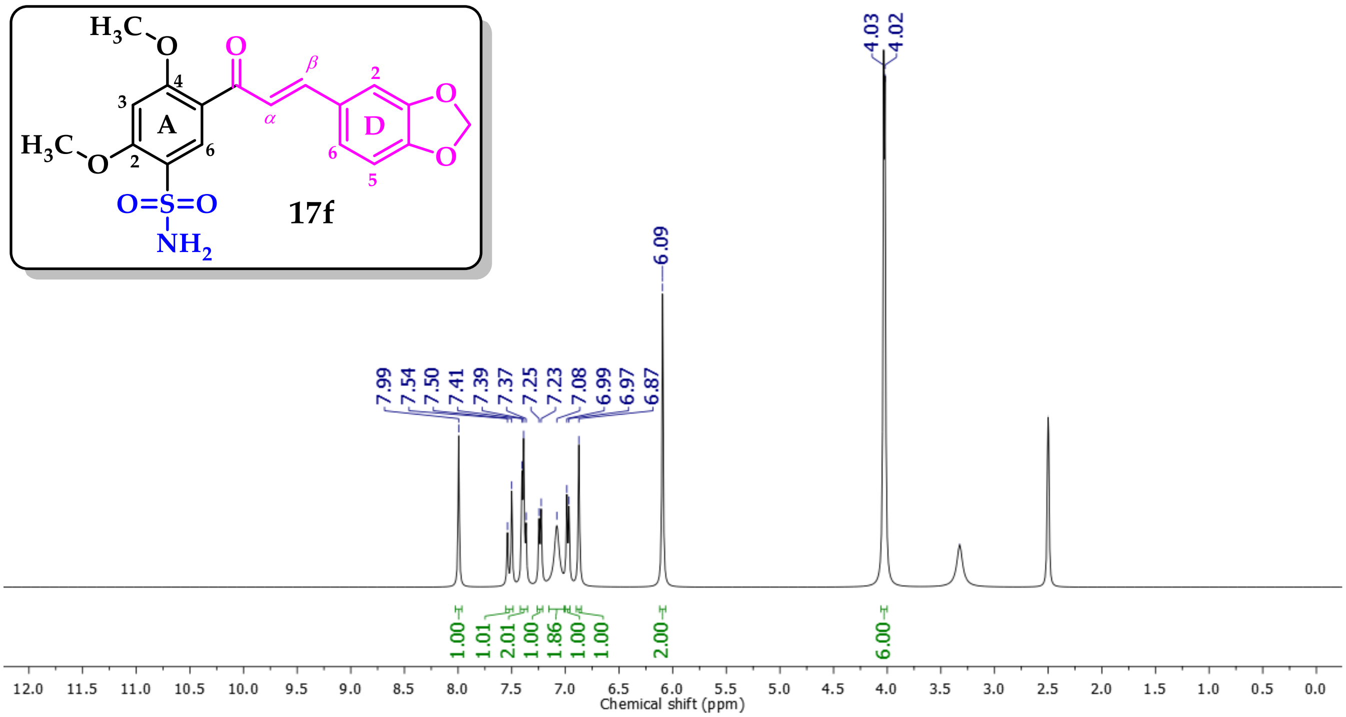


$^{1}H$ NMR (400 MHz, DMSO-*d*₆) spectrum of **17f**.

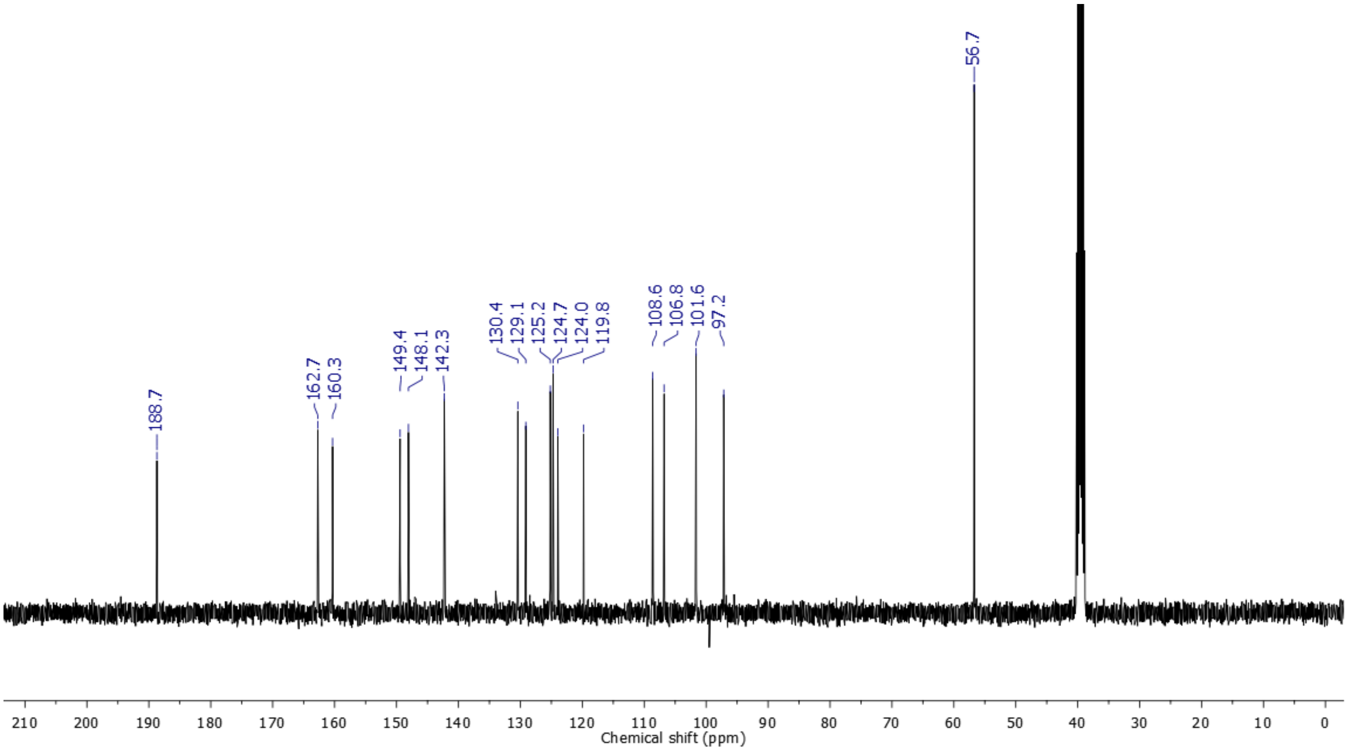


$^{13}C$ NMR (101 MHz, DMSO-$d_6$) spectrum of **17f**.

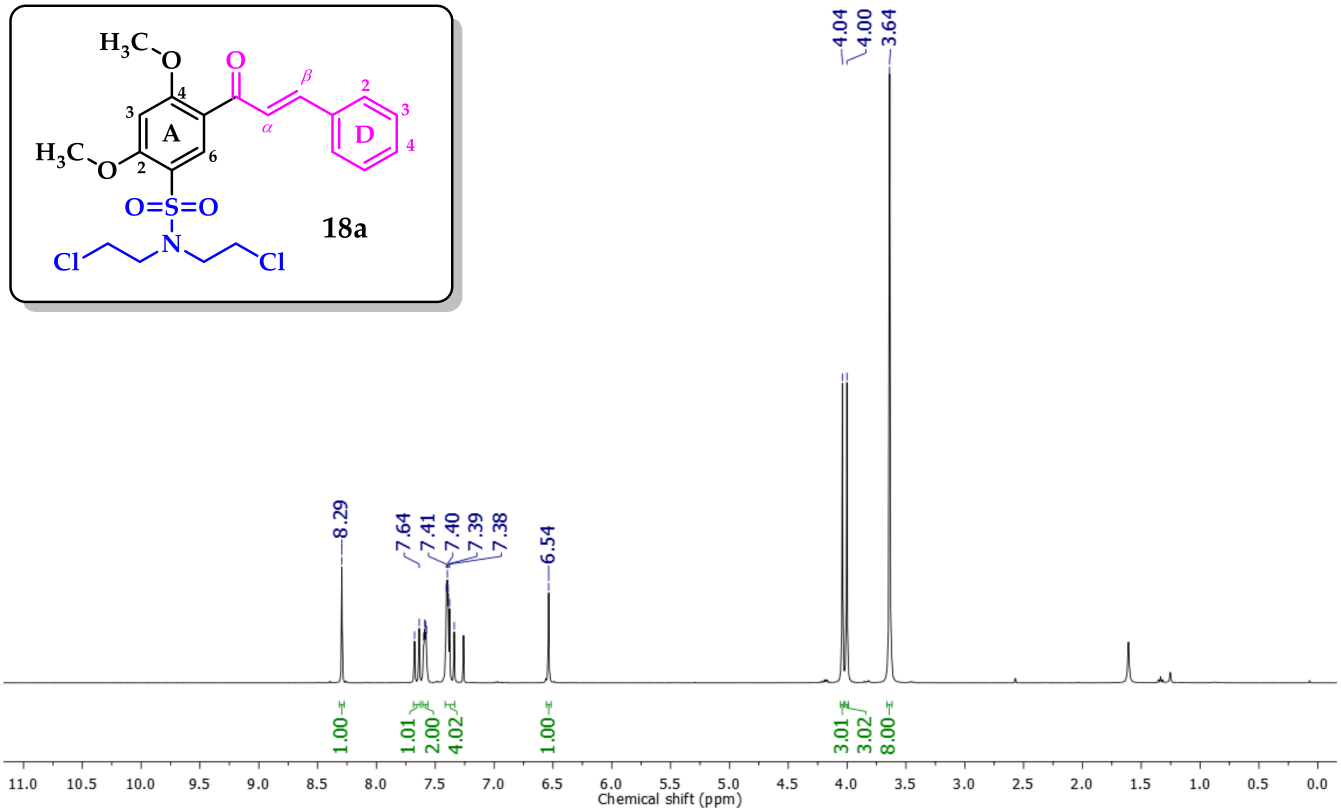


$^{1}$H NMR (400 MHz, CDCl3) spectrum of **18a**.

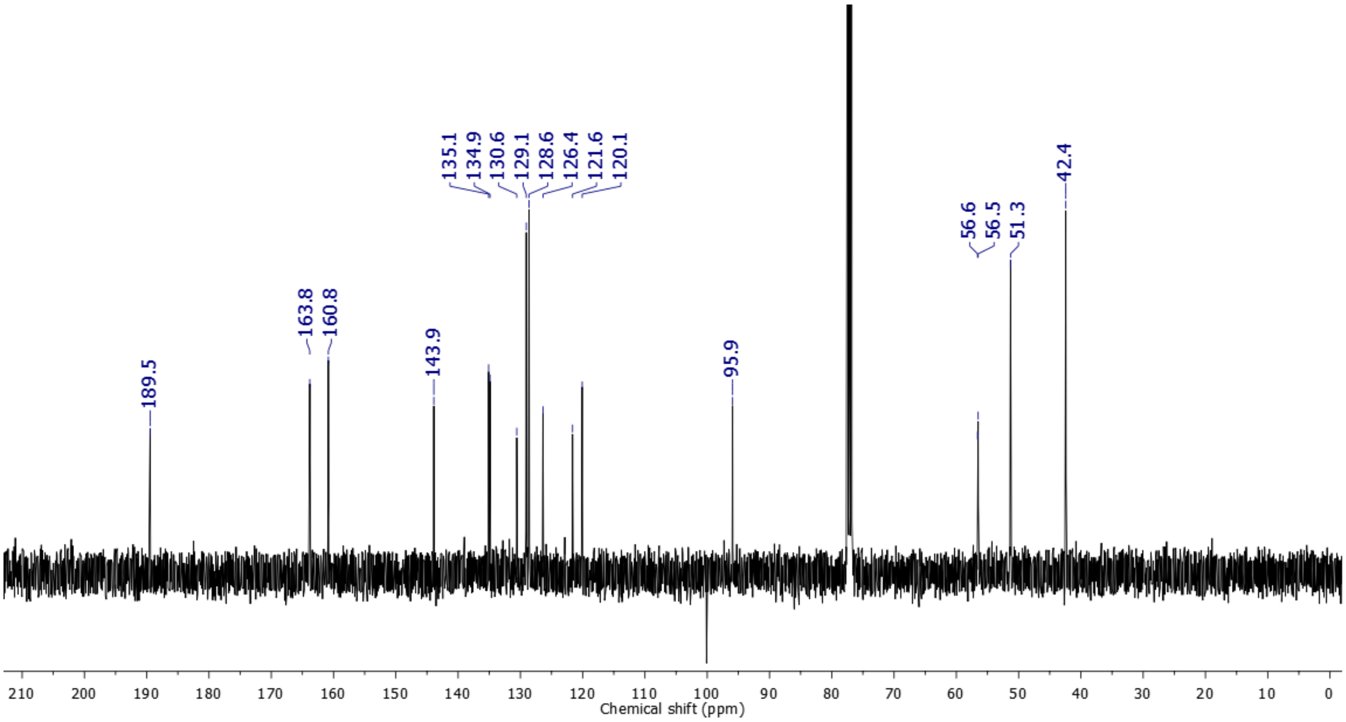


$^{13}$C NMR (101 MHz, CDCl3) spectrum of **18a**.

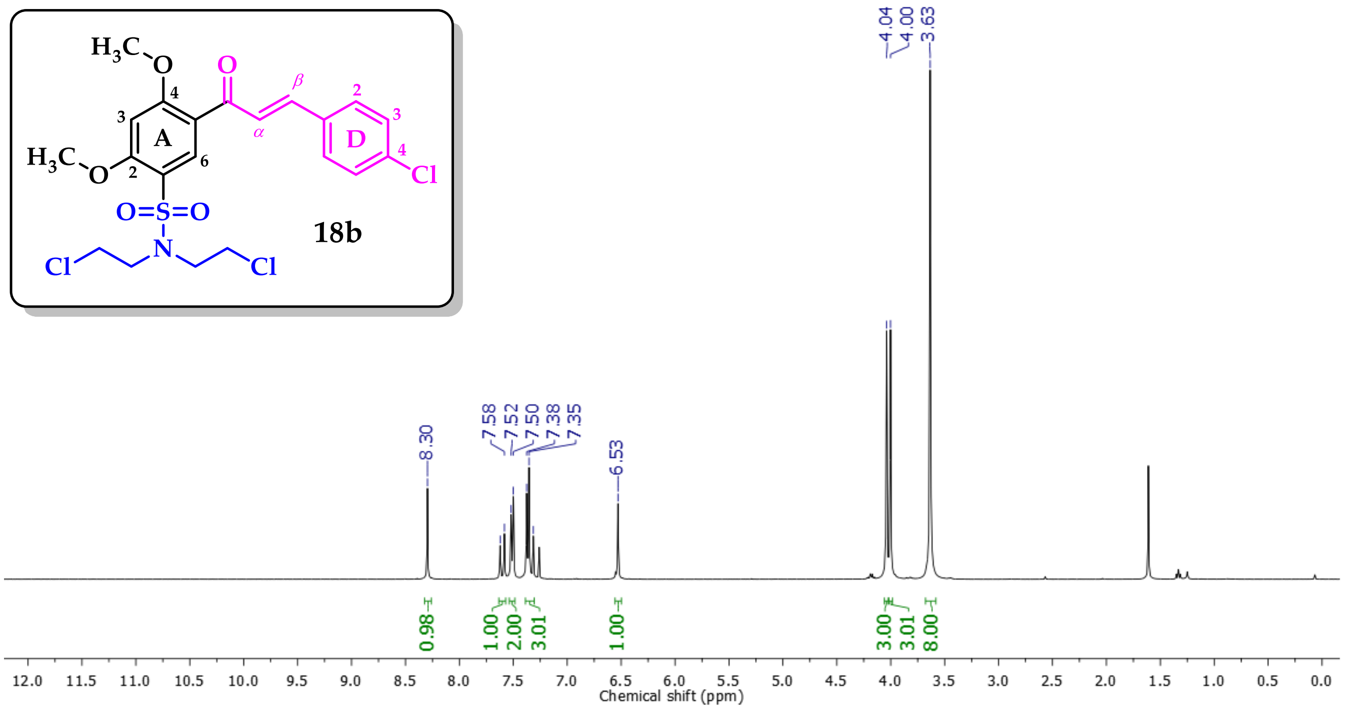


$^{1}H$ NMR (400 MHz, $CDCl_3$) spectrum of **18b**.

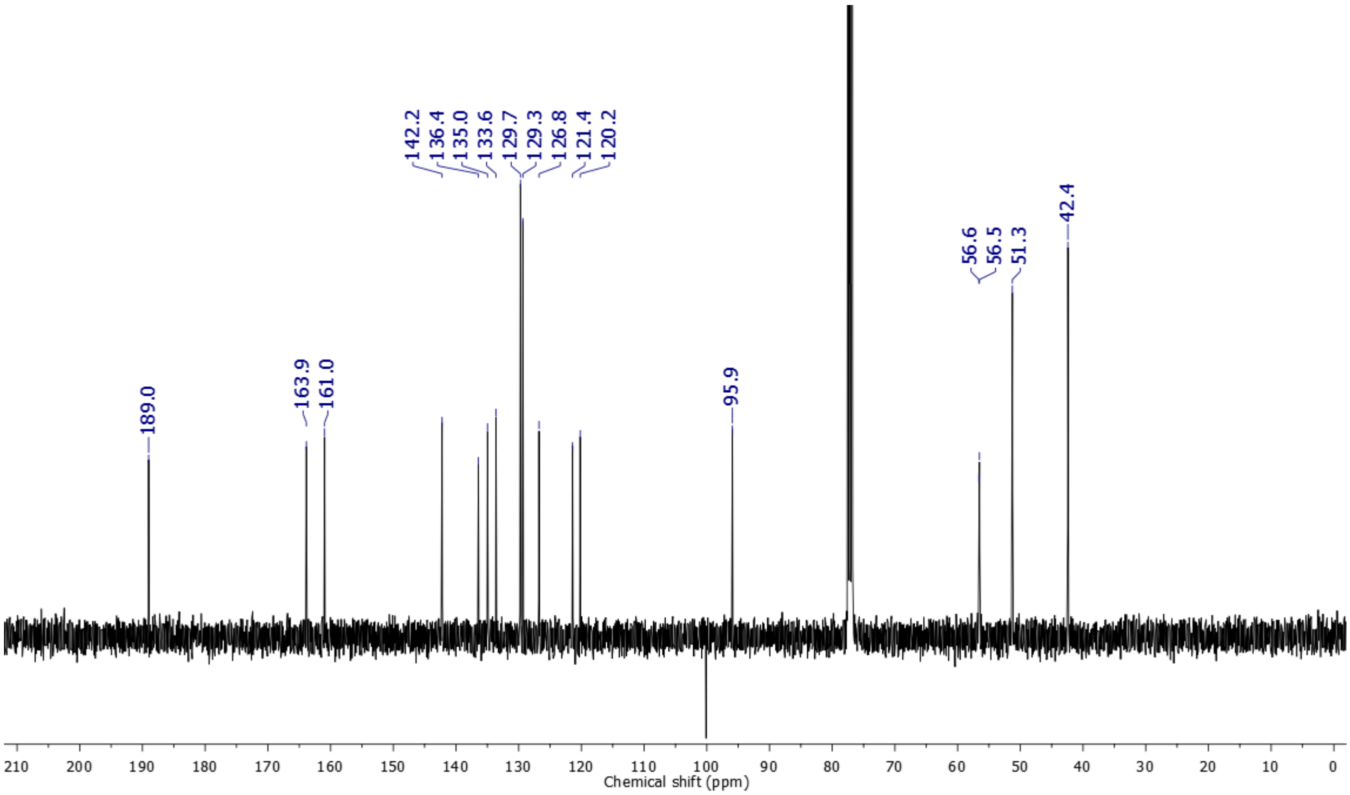


$^{13}C$ NMR (101 MHz, $CDCl_3$) spectrum of **18b**.

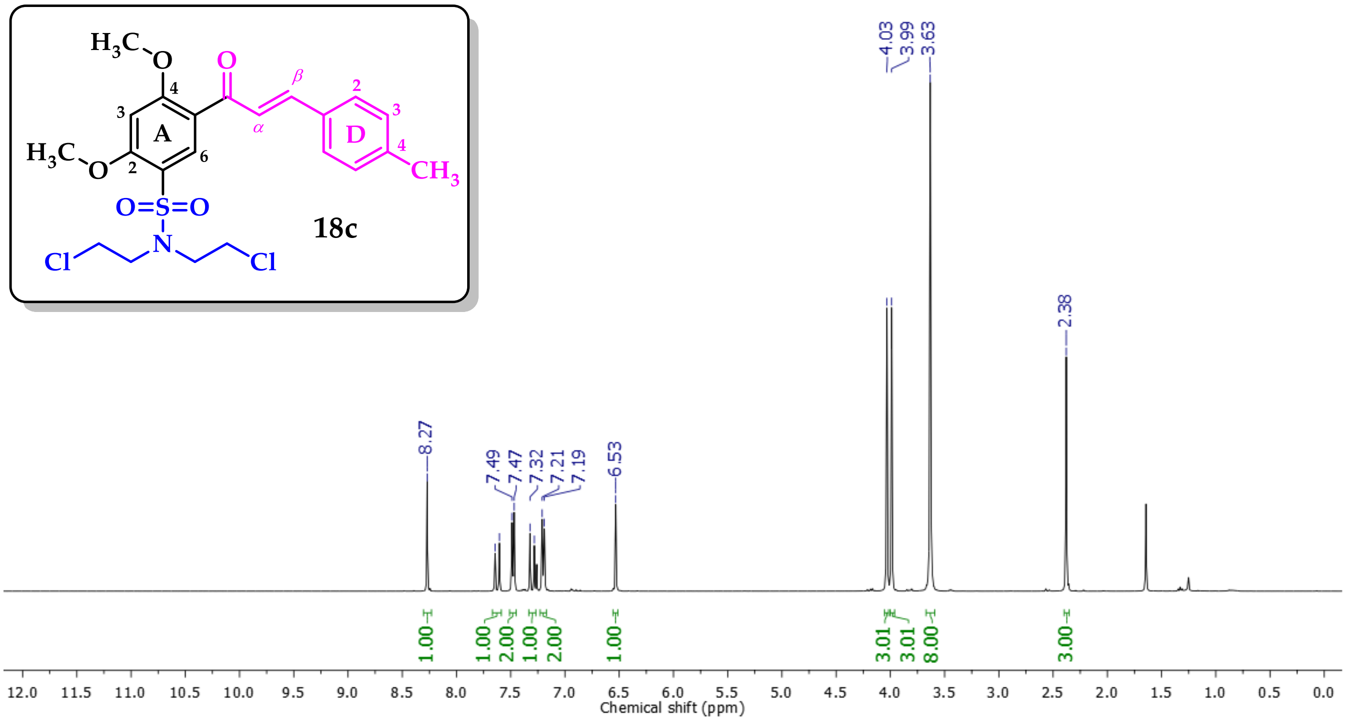


$^{1}H$ NMR (400 MHz, $CDCl_3$) spectrum of **18c**.

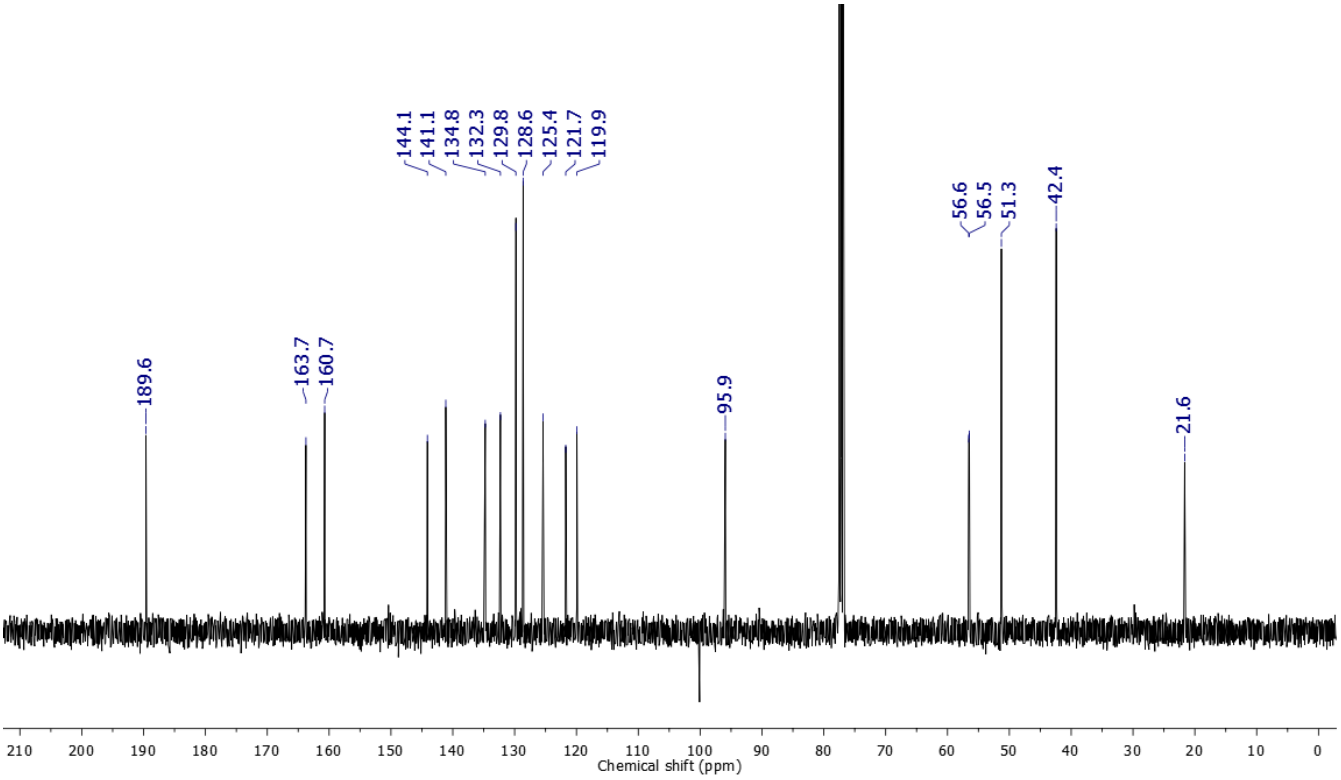


$^{13}C$ NMR (101 MHz, $CDCl_3$) spectrum of **18c**.

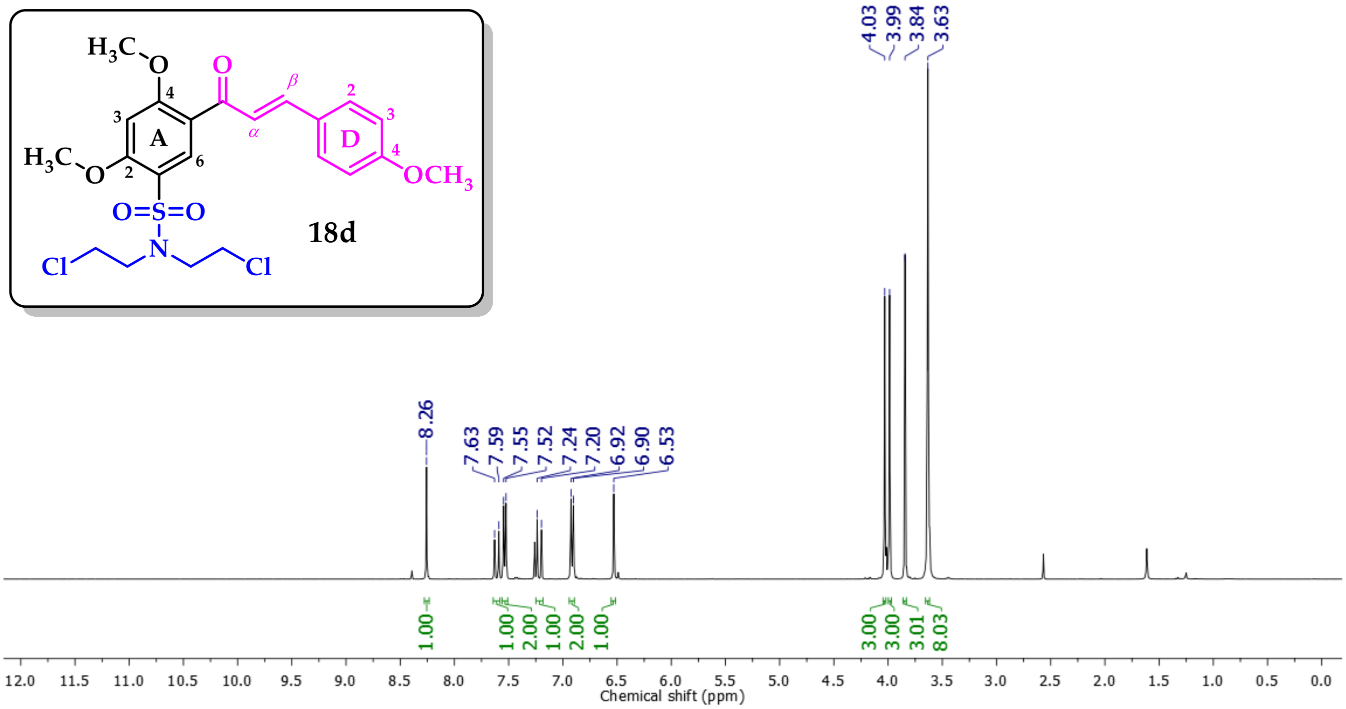


$^{1}$H NMR (400 MHz, CDCl$_3$) spectrum of **18d**.

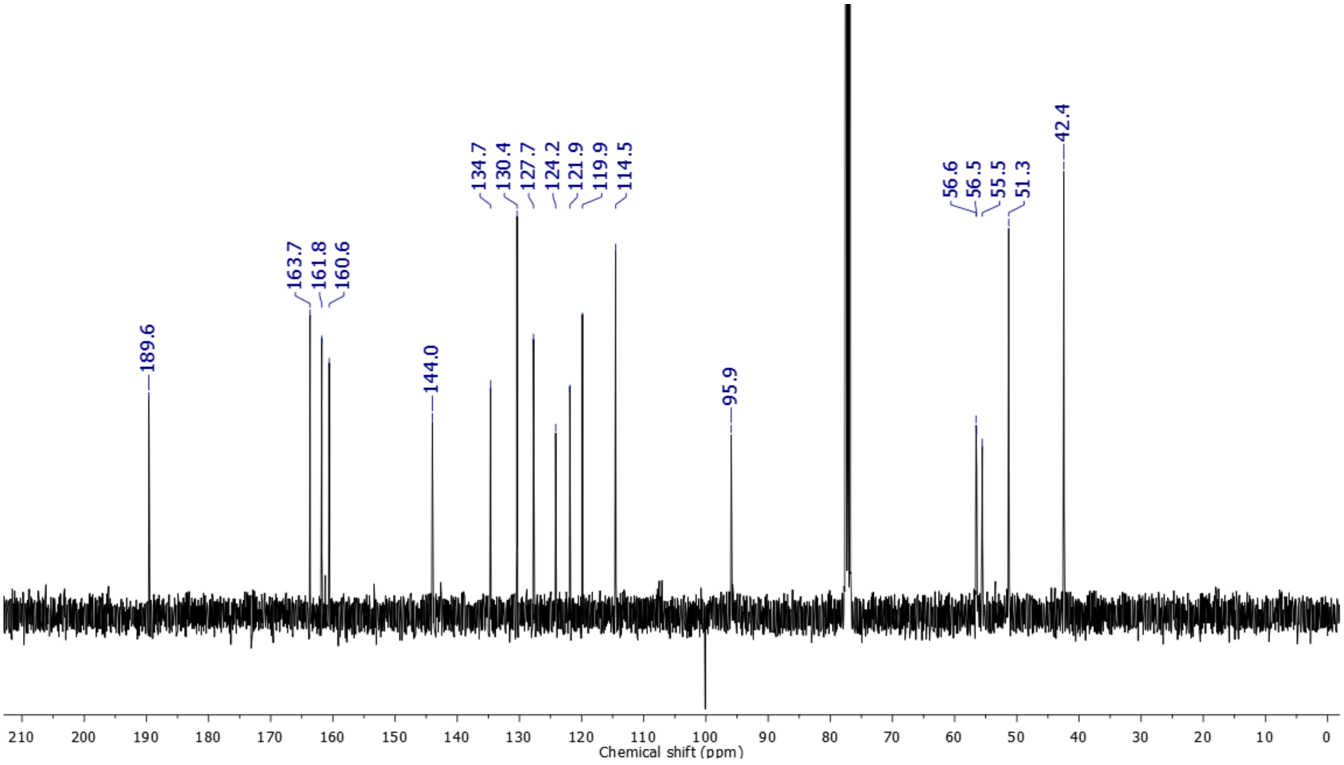


$^{13}$C NMR (101 MHz, CDCl$_3$) spectrum of **18d**.

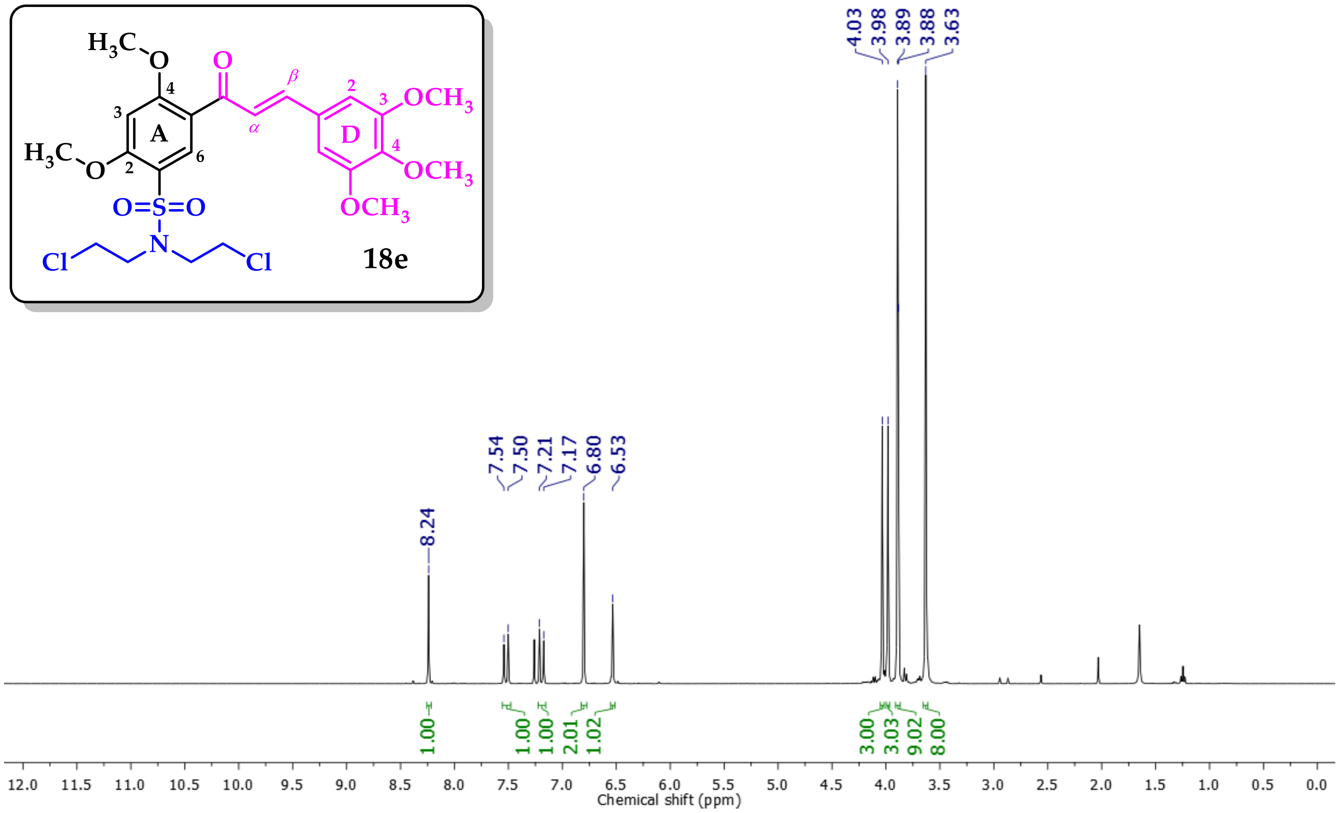


$^{1}$H NMR (400 MHz, CDCl3) spectrum of **18e**.

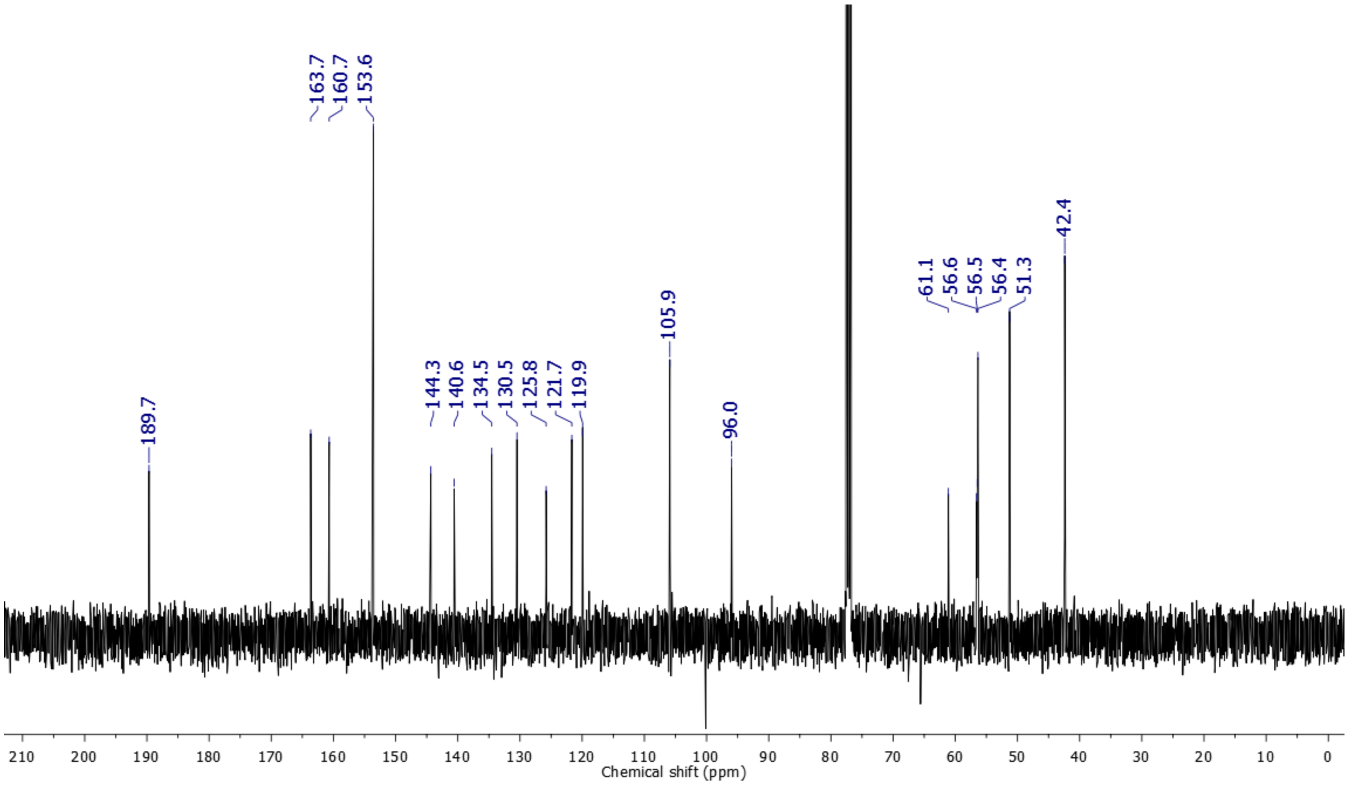


$^{13}$C NMR (101 MHz, CDCl3) spectrum of **18e**.

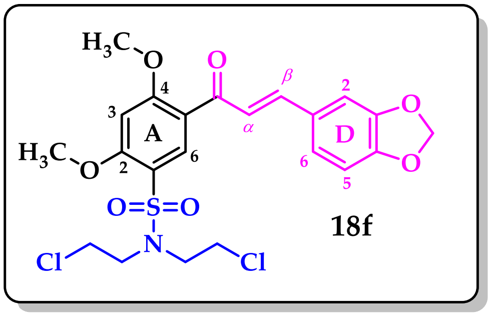


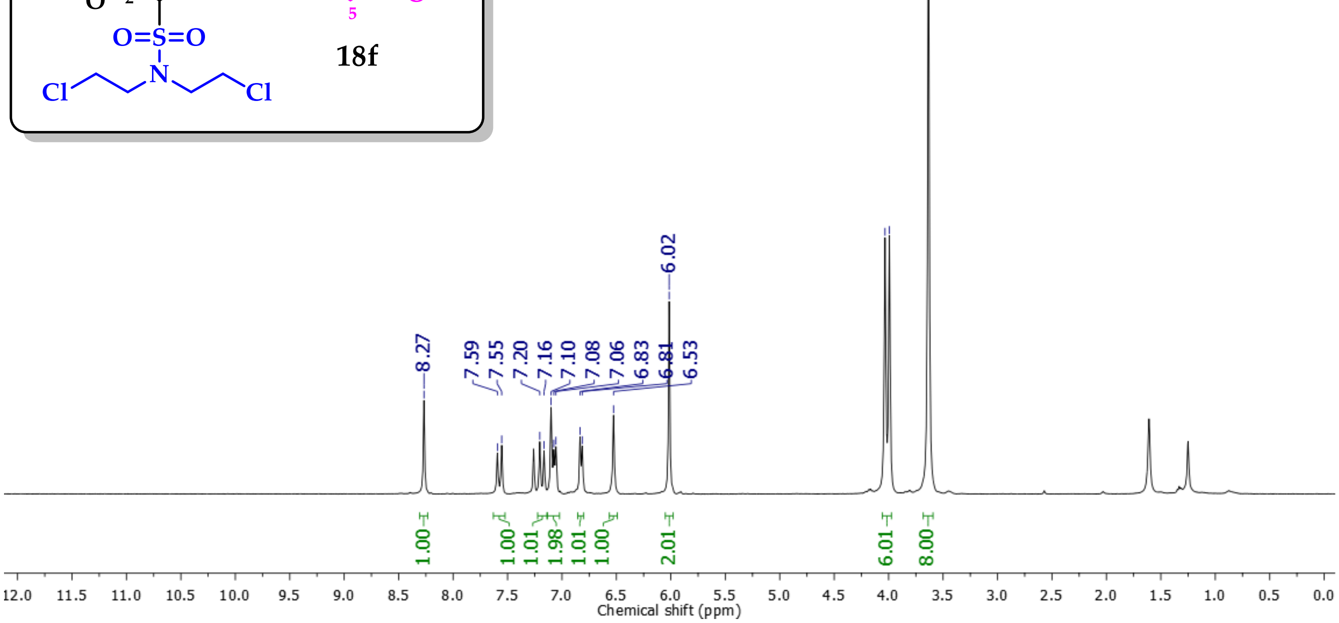


¹H NMR (400 MHz, $CDCl_3$) spectrum of **18f**.

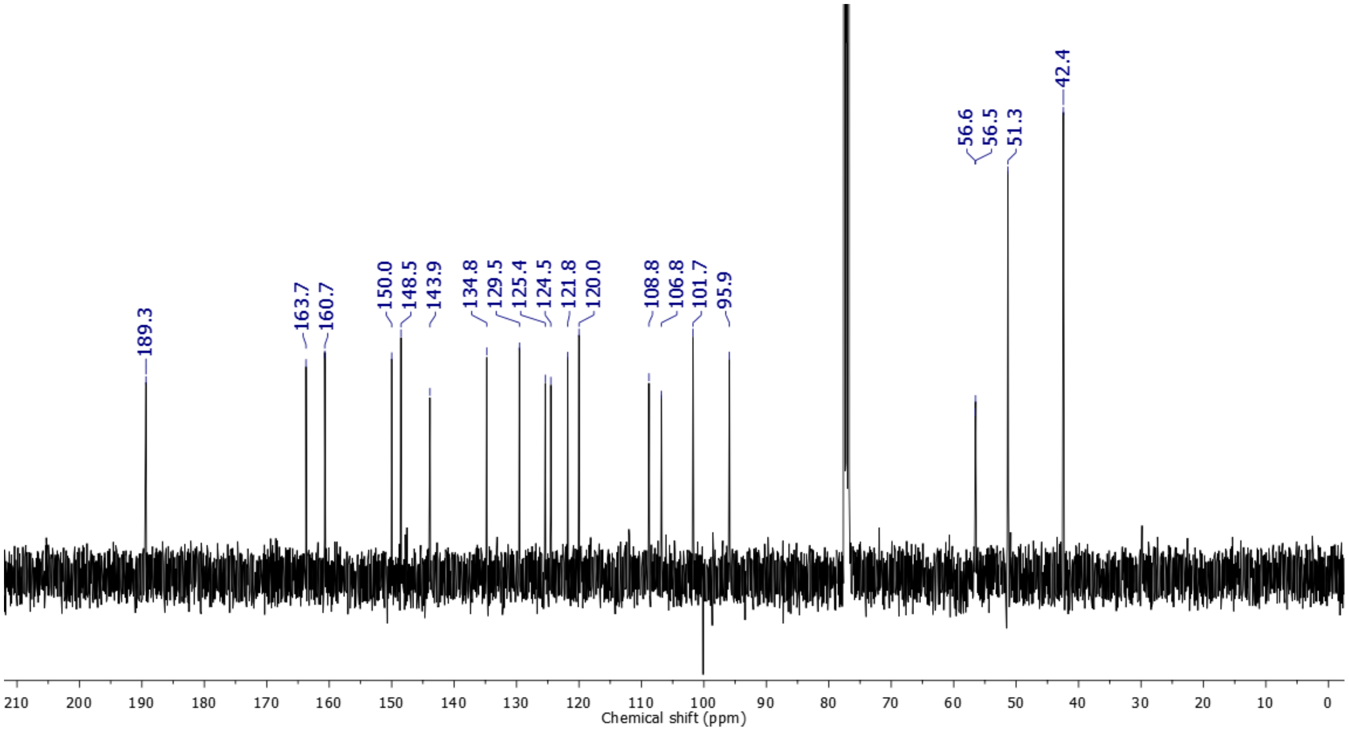


¹³C NMR (101 MHz, $CDCl_3$) spectrum of **18f**.

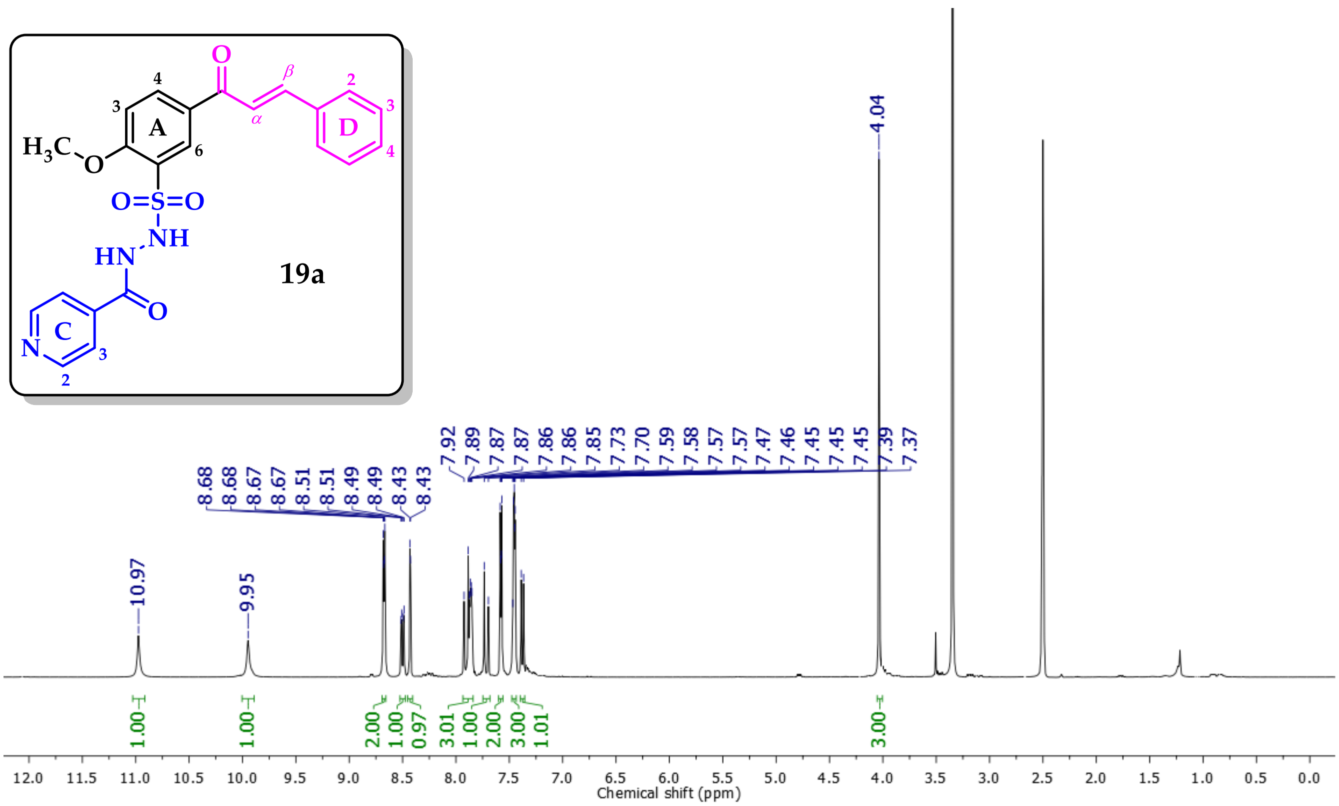


$^{1}$H NMR (400 MHz, DMSO-$d_6$) spectrum of **19a**.

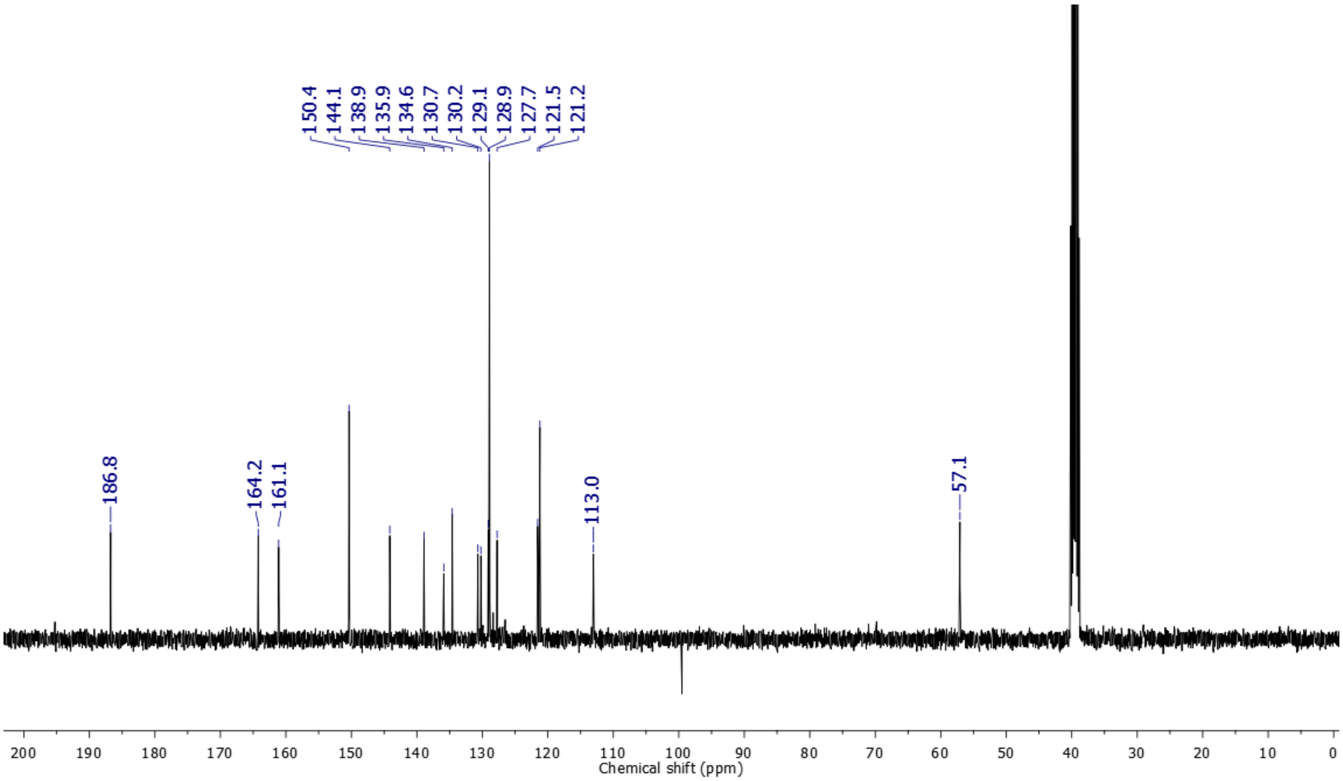


$^{13}$C NMR (101 MHz, DMSO-$d_6$) spectrum of **19a**.

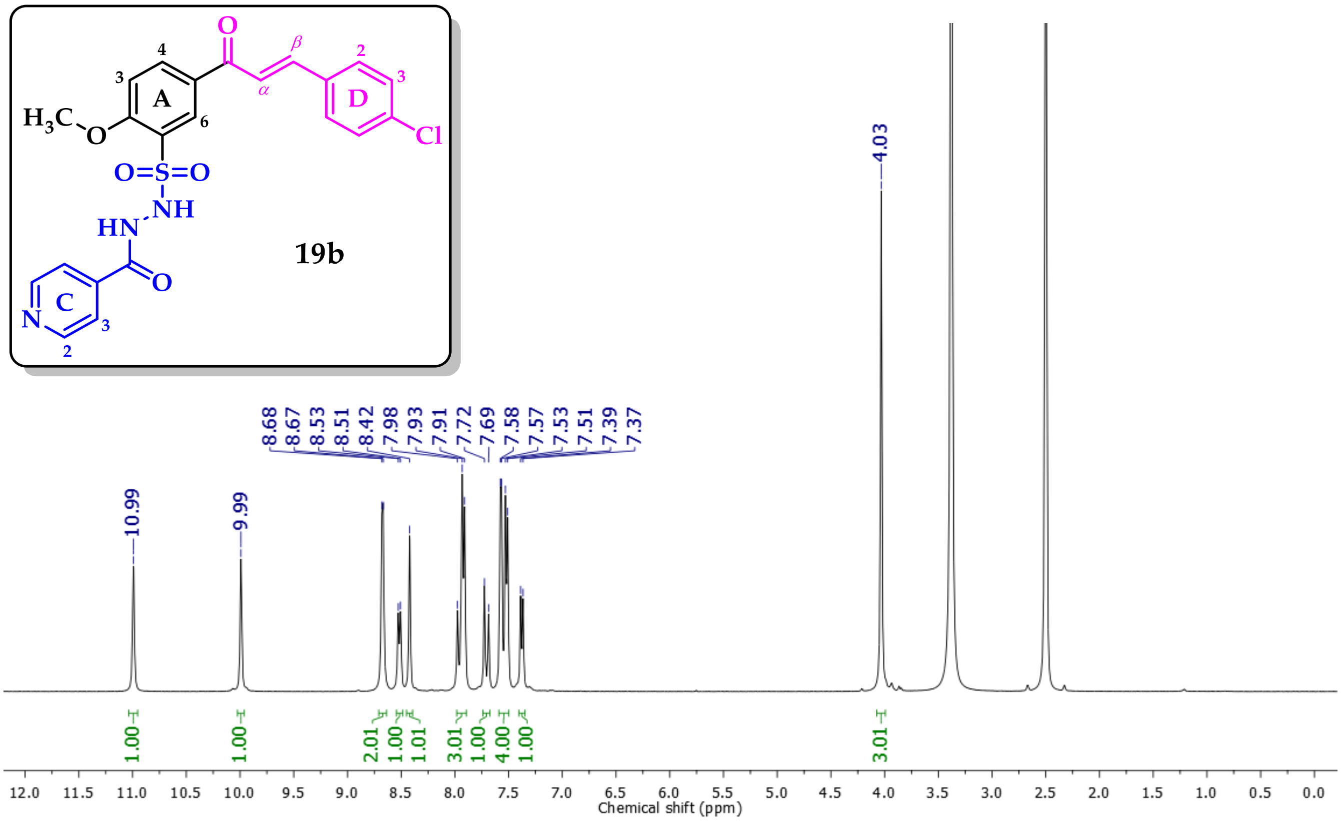


$^{1}$H NMR (400 MHz, DMSO-$d_6$) spectrum of **19b**.

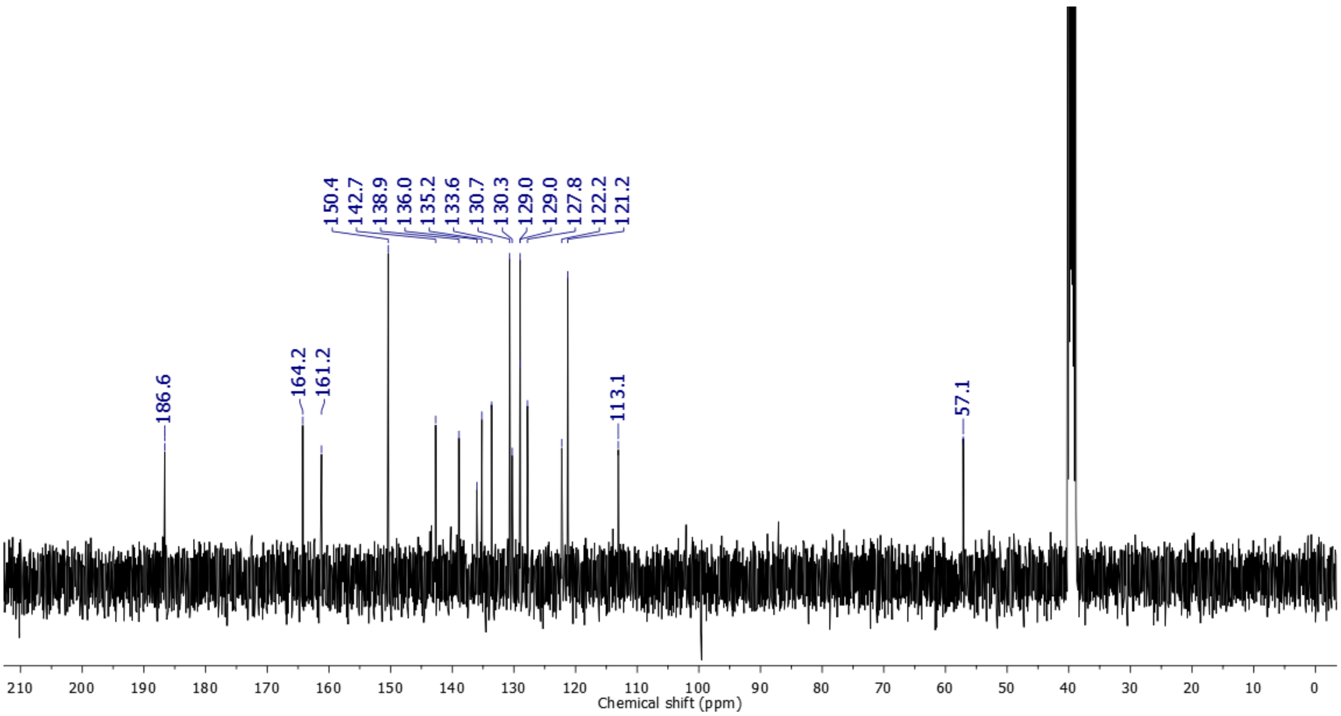


$^{13}$C NMR (101 MHz, DMSO-$d_6$) spectrum of **19b**.

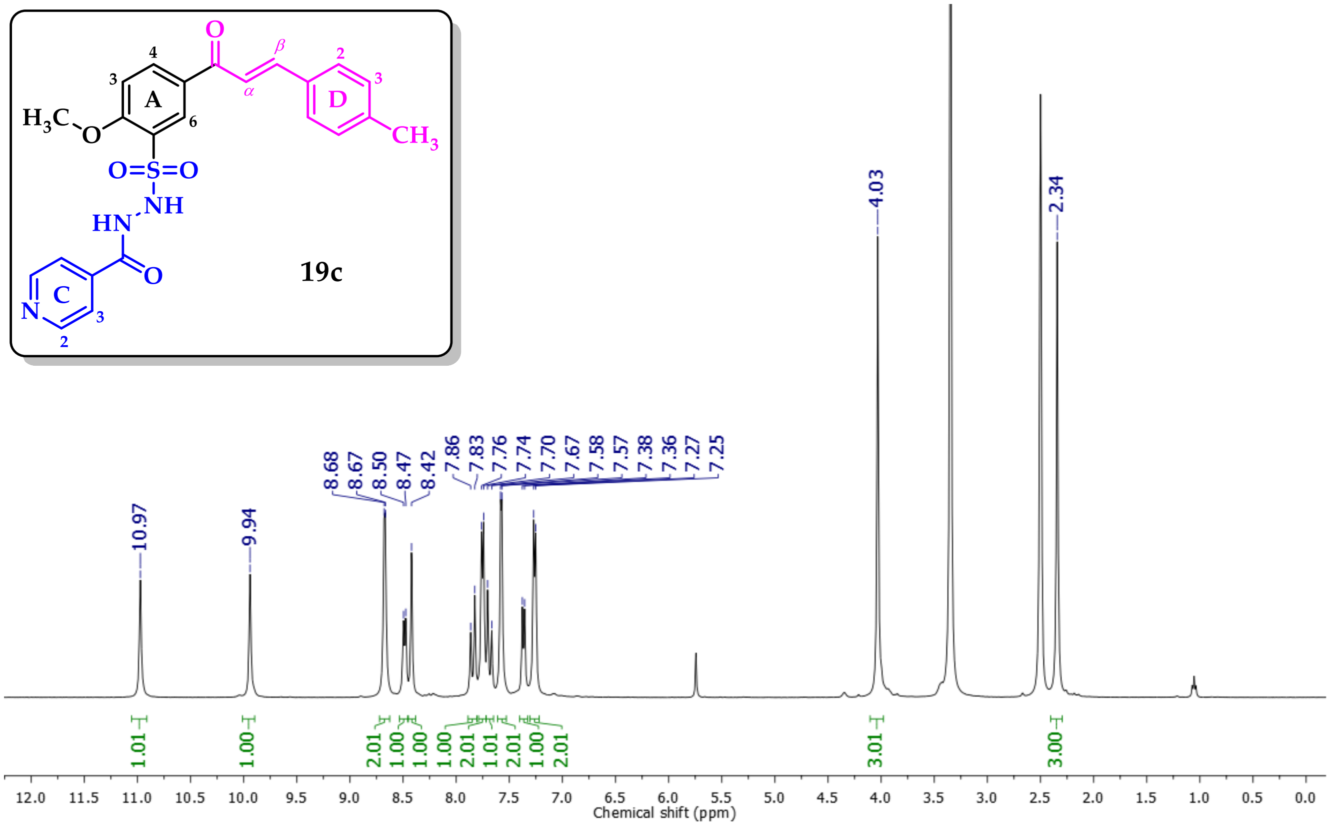


$^{1}$H NMR (400 MHz, DMSO-$d_6$) spectrum of **19c**.

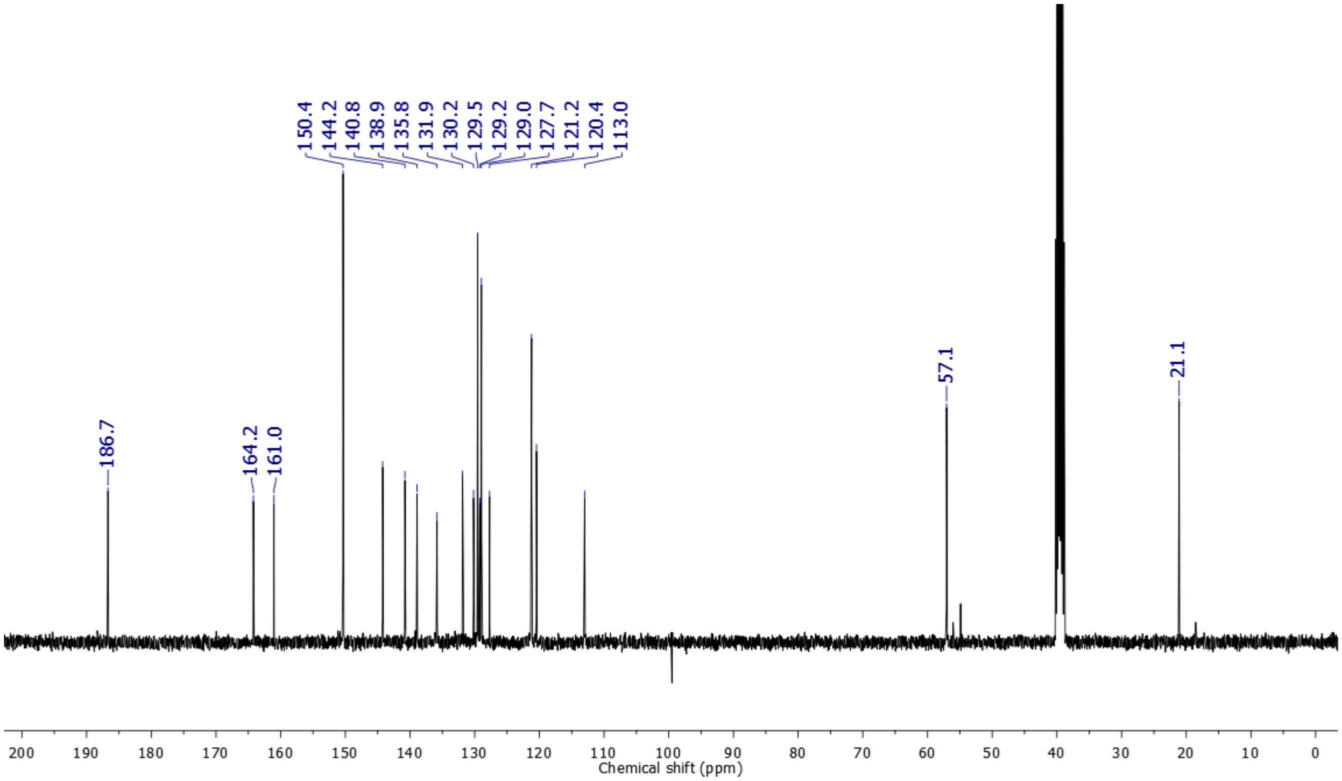


$^{13}$C NMR (101 MHz, DMSO-$d_6$) spectrum of **19c**.

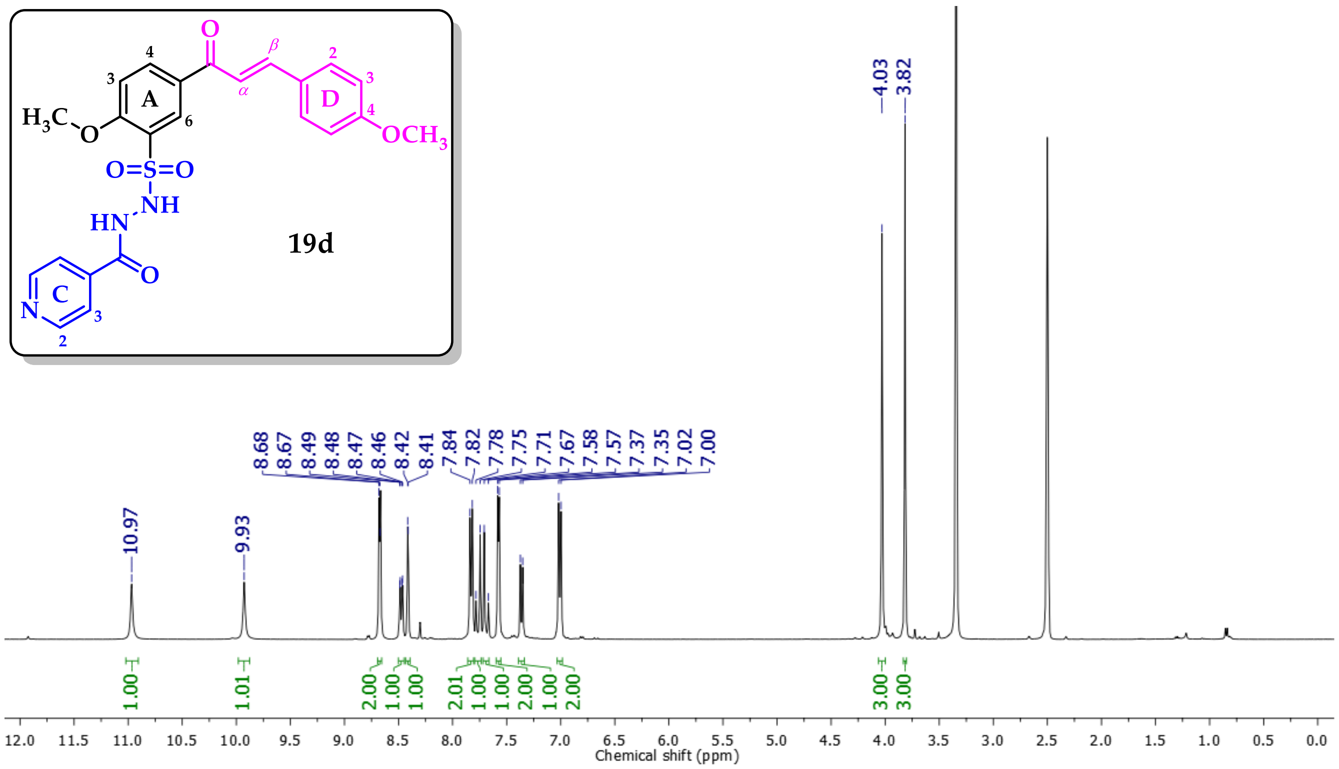


$^{1}$H NMR (400 MHz, DMSO-$d_6$) spectrum of **19d**.

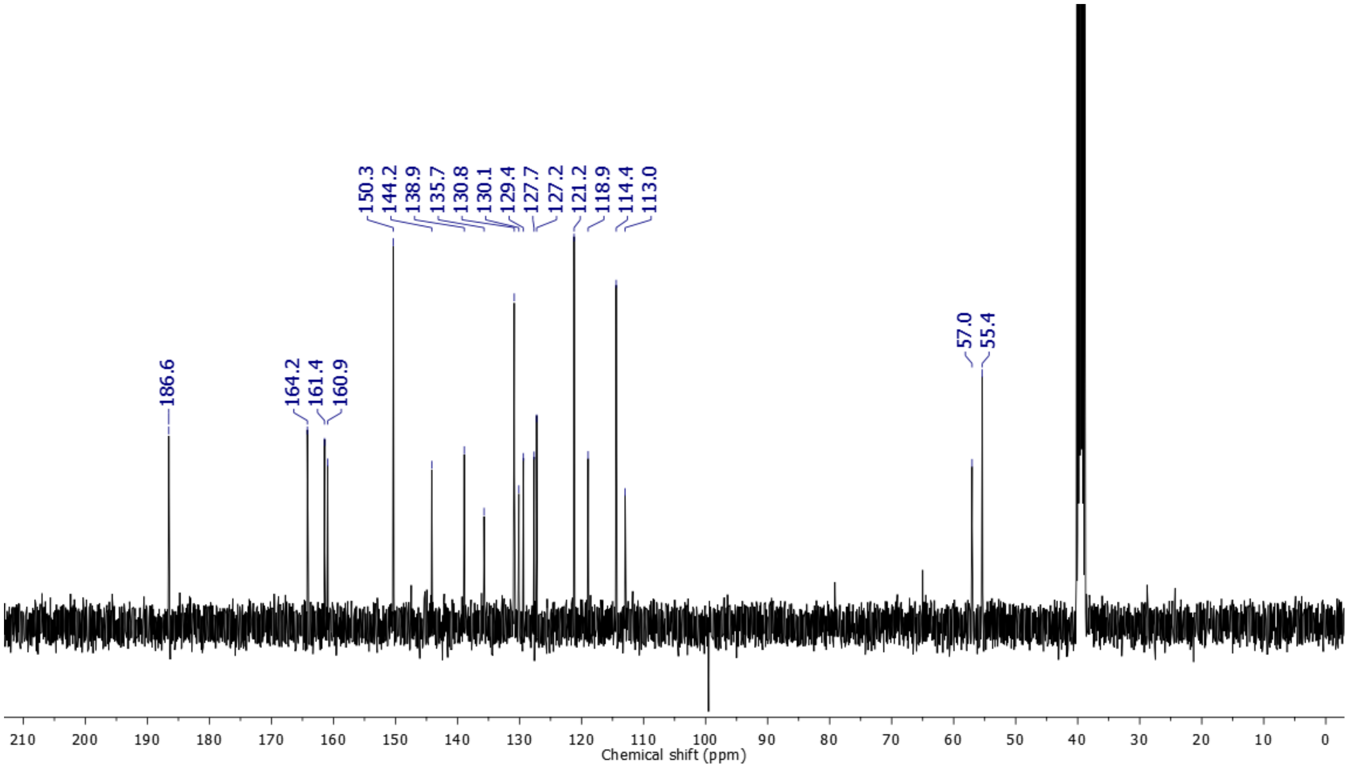


$^{13}$C NMR (101 MHz, DMSO-$d_6$) spectrum of **19d**.

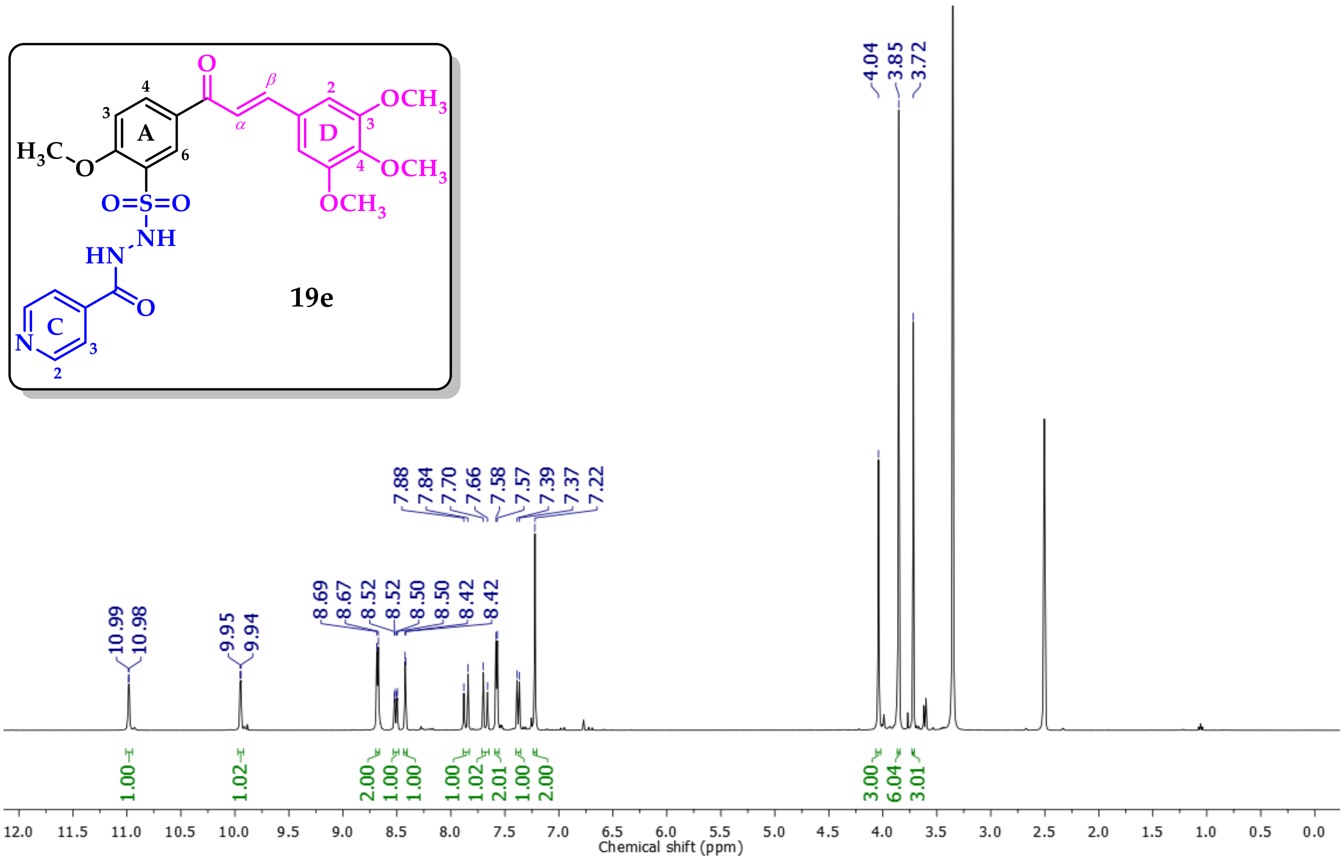


$^{1}$H NMR (400 MHz, DMSO-$d_6$) spectrum of **19e**.

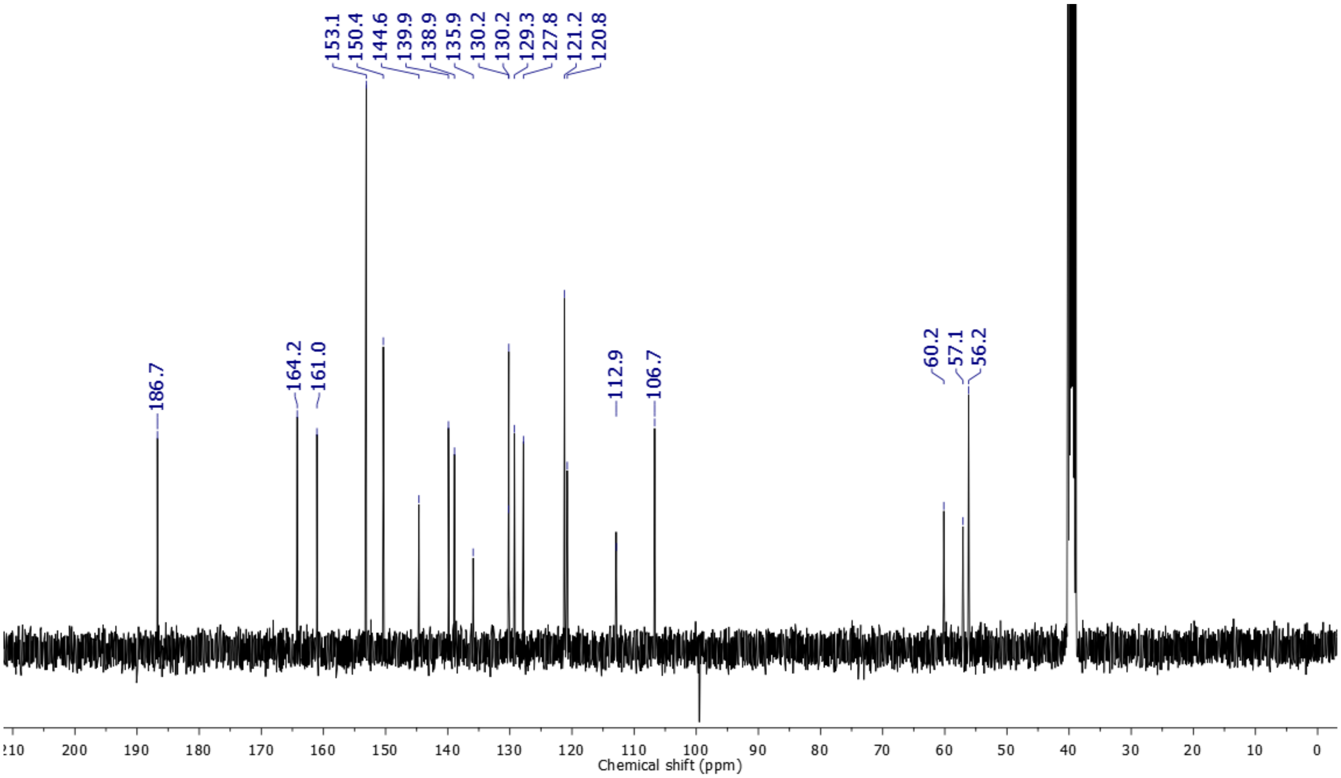


$^{13}$C NMR (101 MHz, DMSO-$d_6$) spectrum of **19e**.

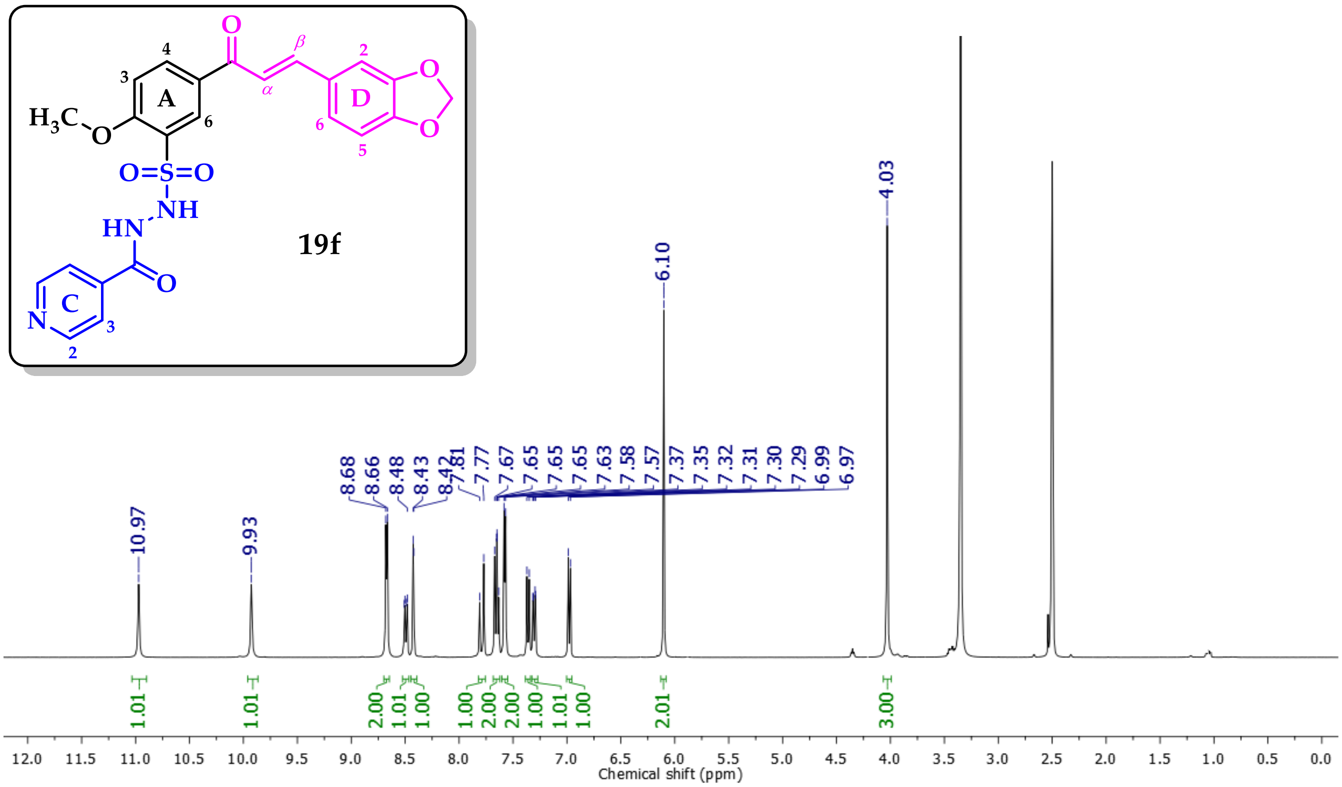


$^{1}H$ NMR (400 MHz, DMSO-*d*6) spectrum of **19f**.

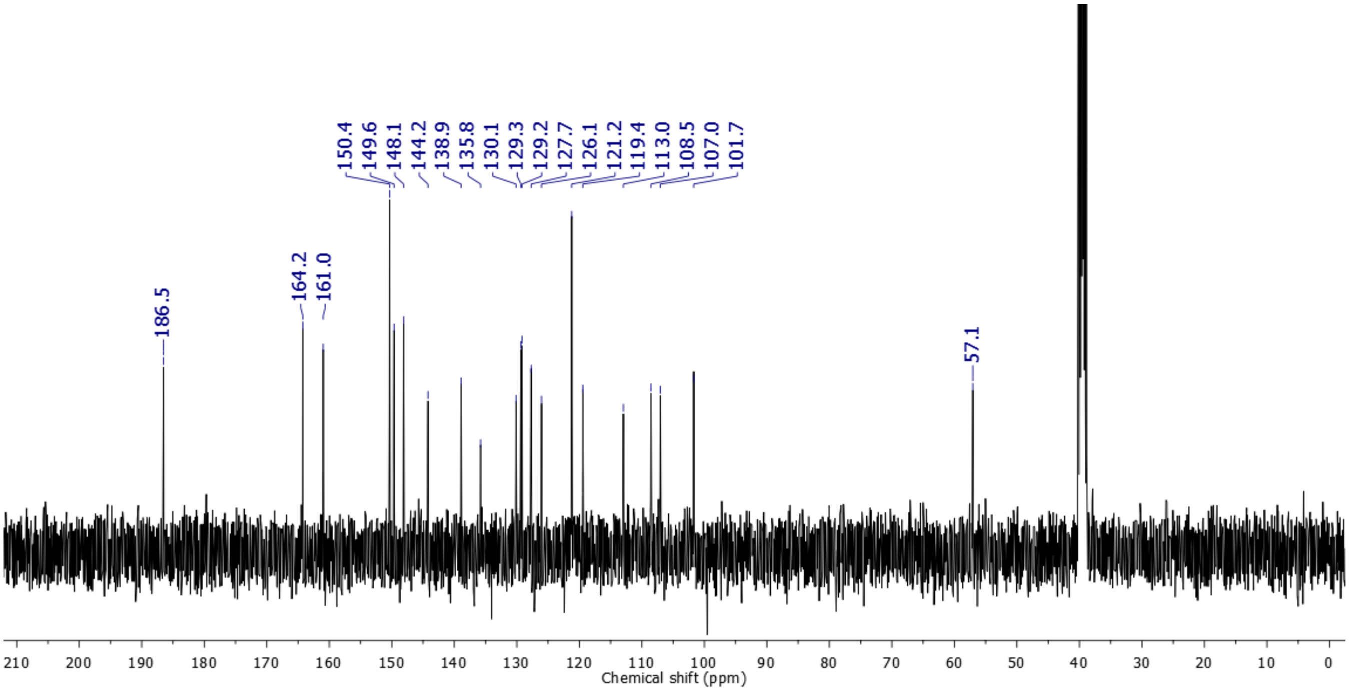


$^{13}C$ NMR (101 MHz, DMSO-*d*6) spectrum of **19f**.

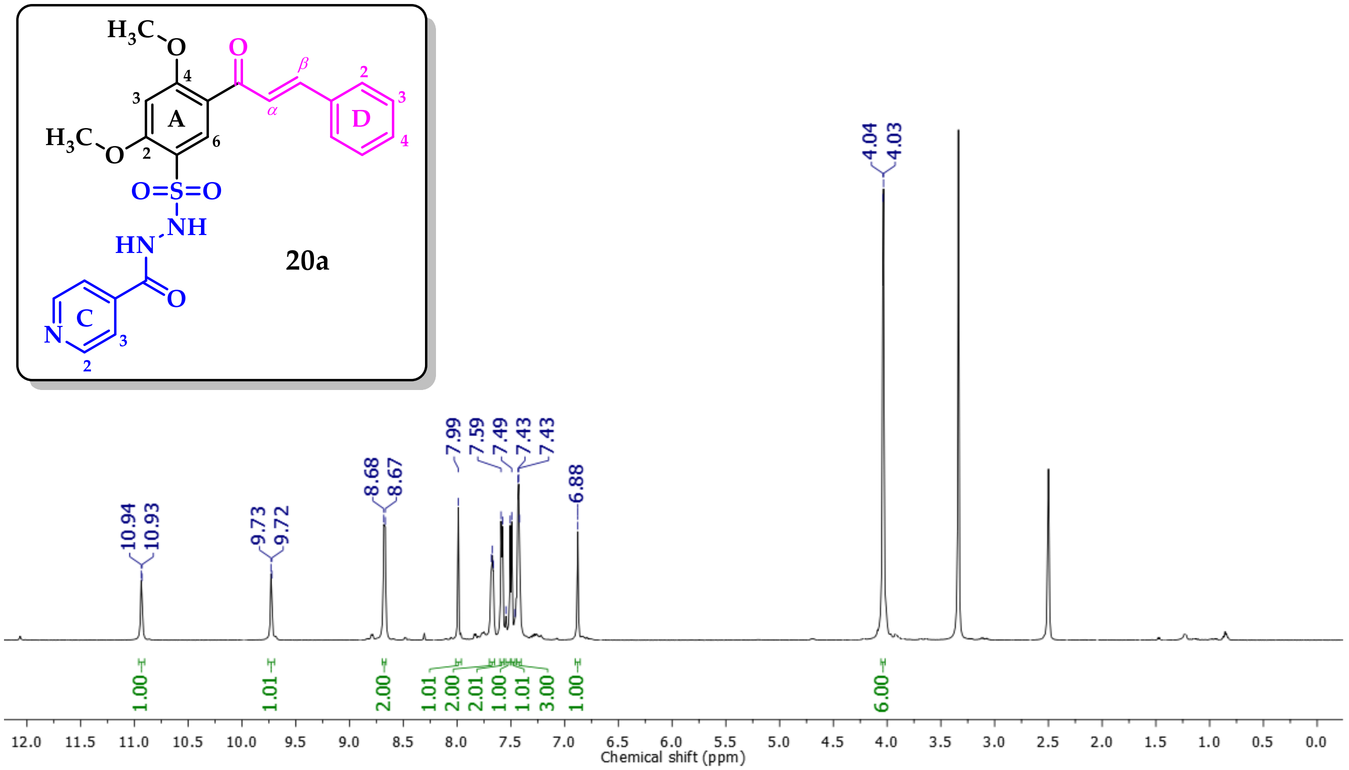


$^{1}H$ NMR (400 MHz, DMSO-*d*6) spectrum of **20a**.

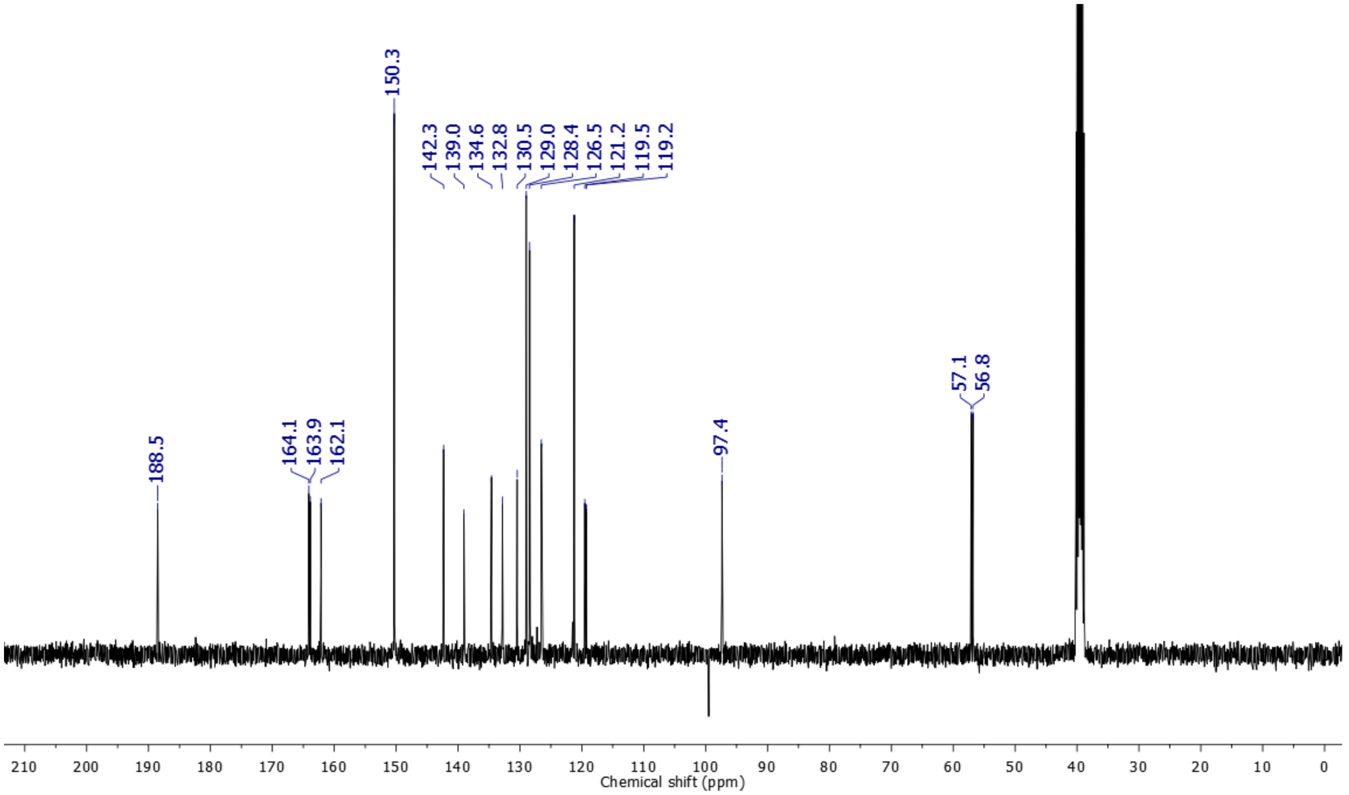


$^{13}C$ NMR (101 MHz, DMSO-*d*6) spectrum of **20a**.

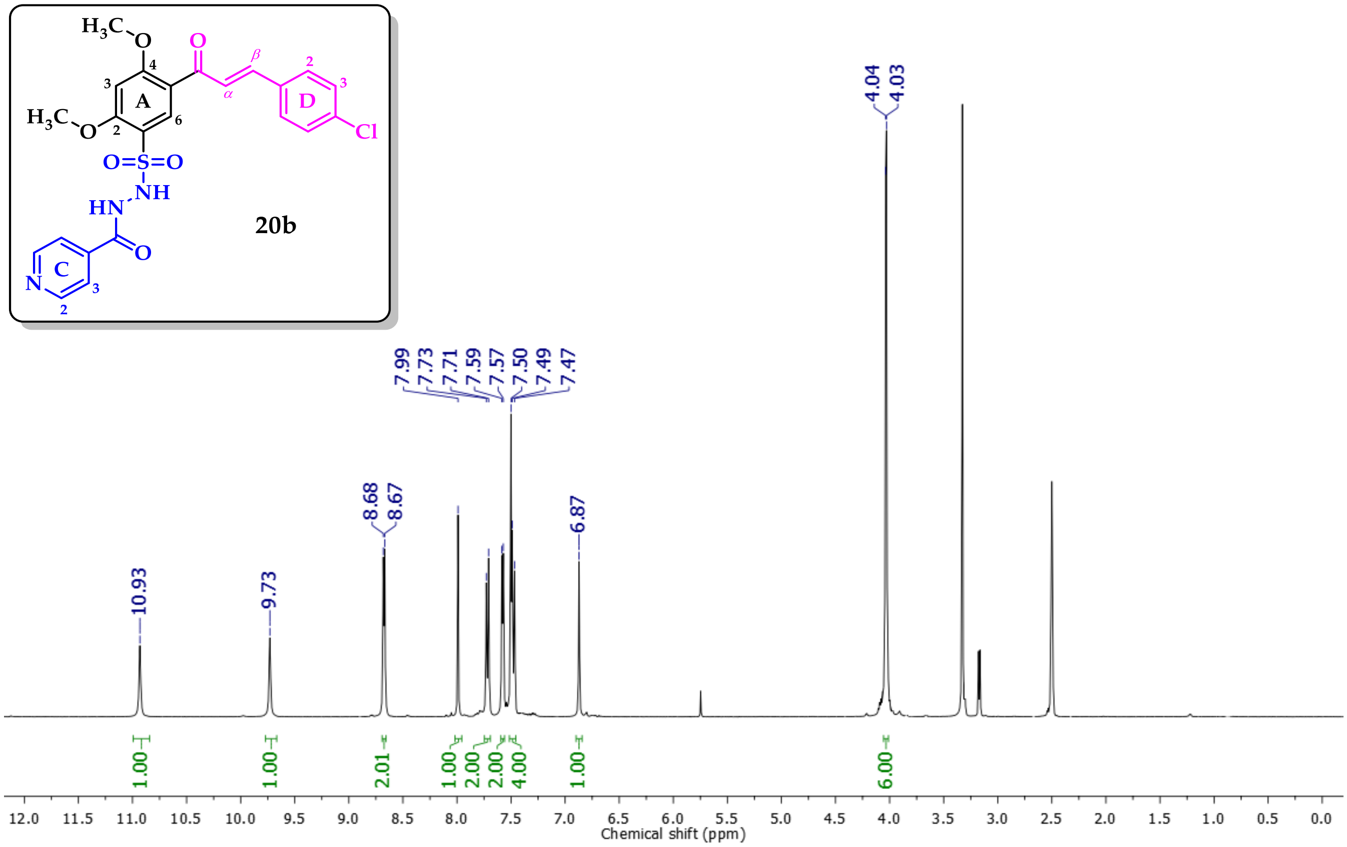

$^{1}H$ NMR (400 MHz, DMSO-$d_6$) spectrum of **20b**.

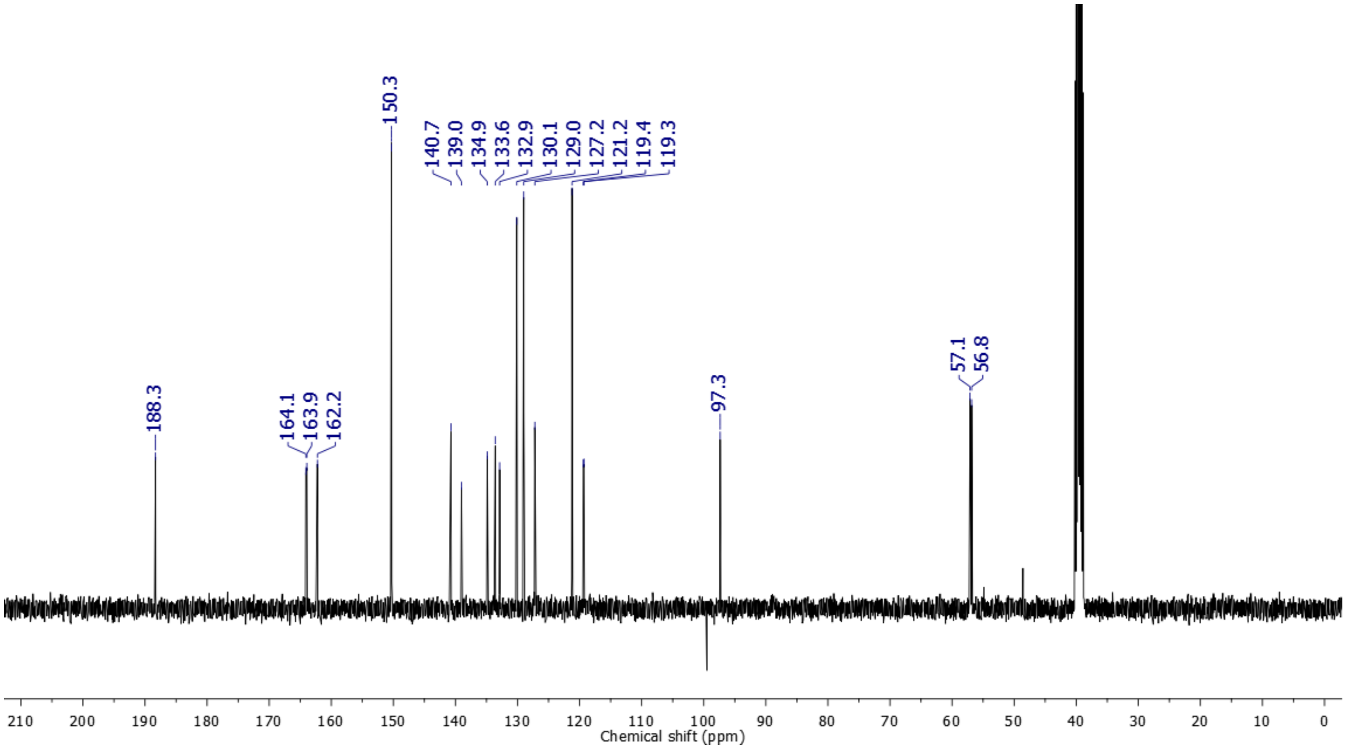

$^{13}C$ NMR (101 MHz, DMSO-$d_6$) spectrum of **20b**.

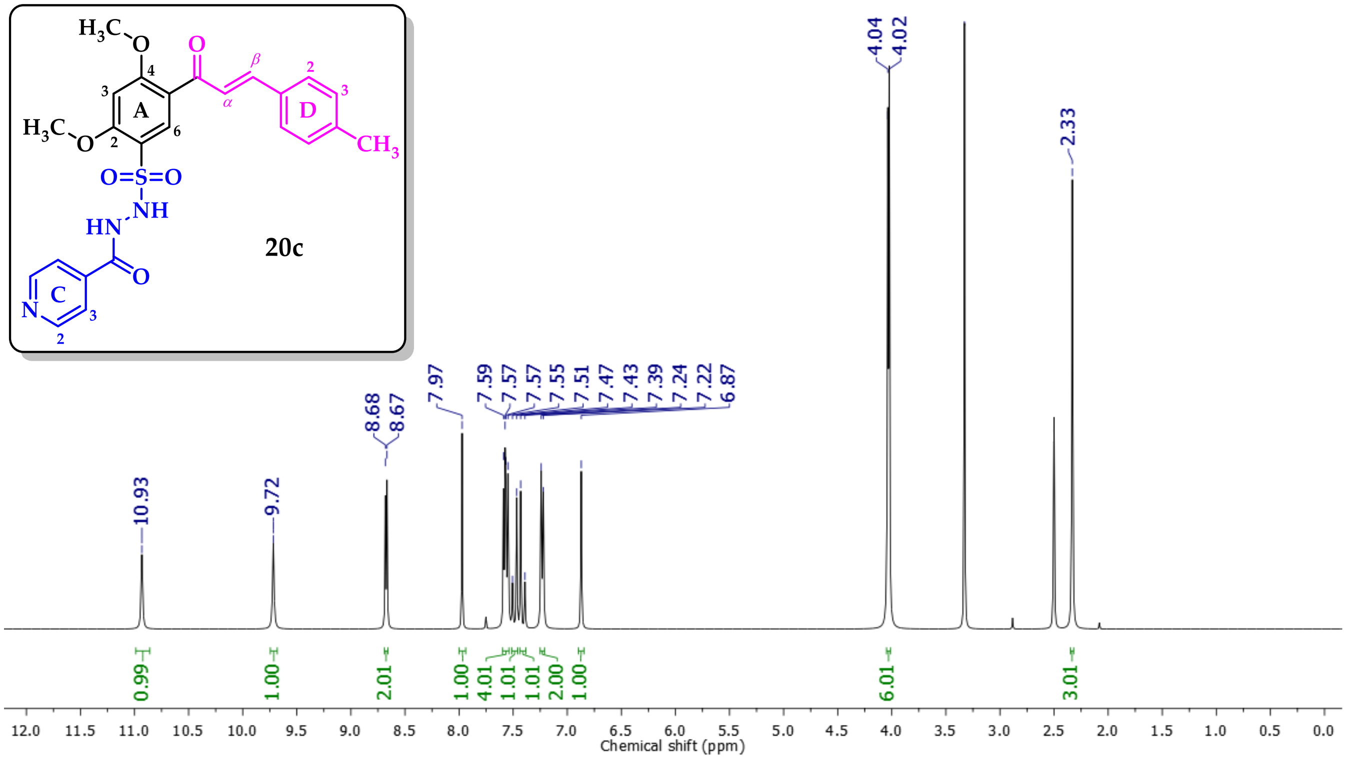

¹H NMR (400 MHz, DMSO-$d_6$) spectrum of **20c**.

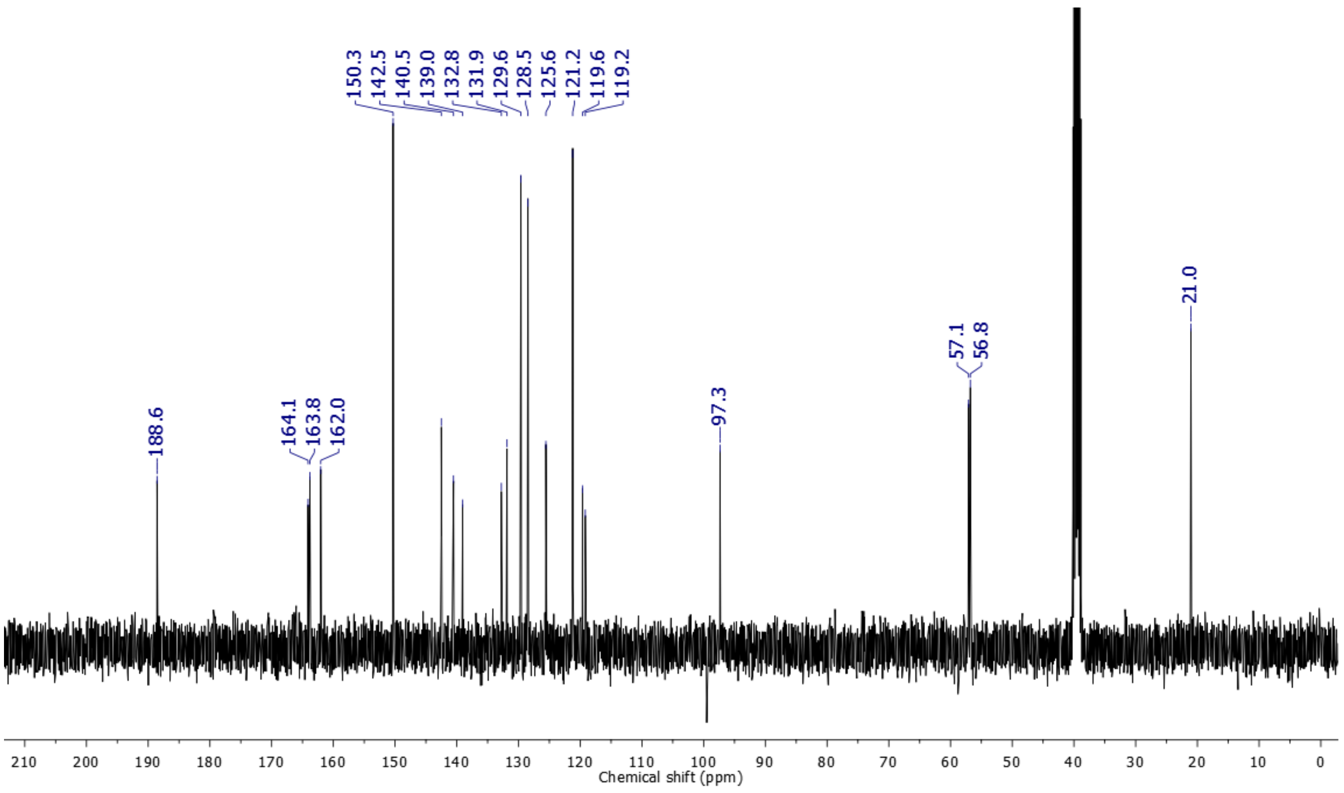

¹³C NMR (101 MHz, DMSO-$d_6$) spectrum of **20c**.

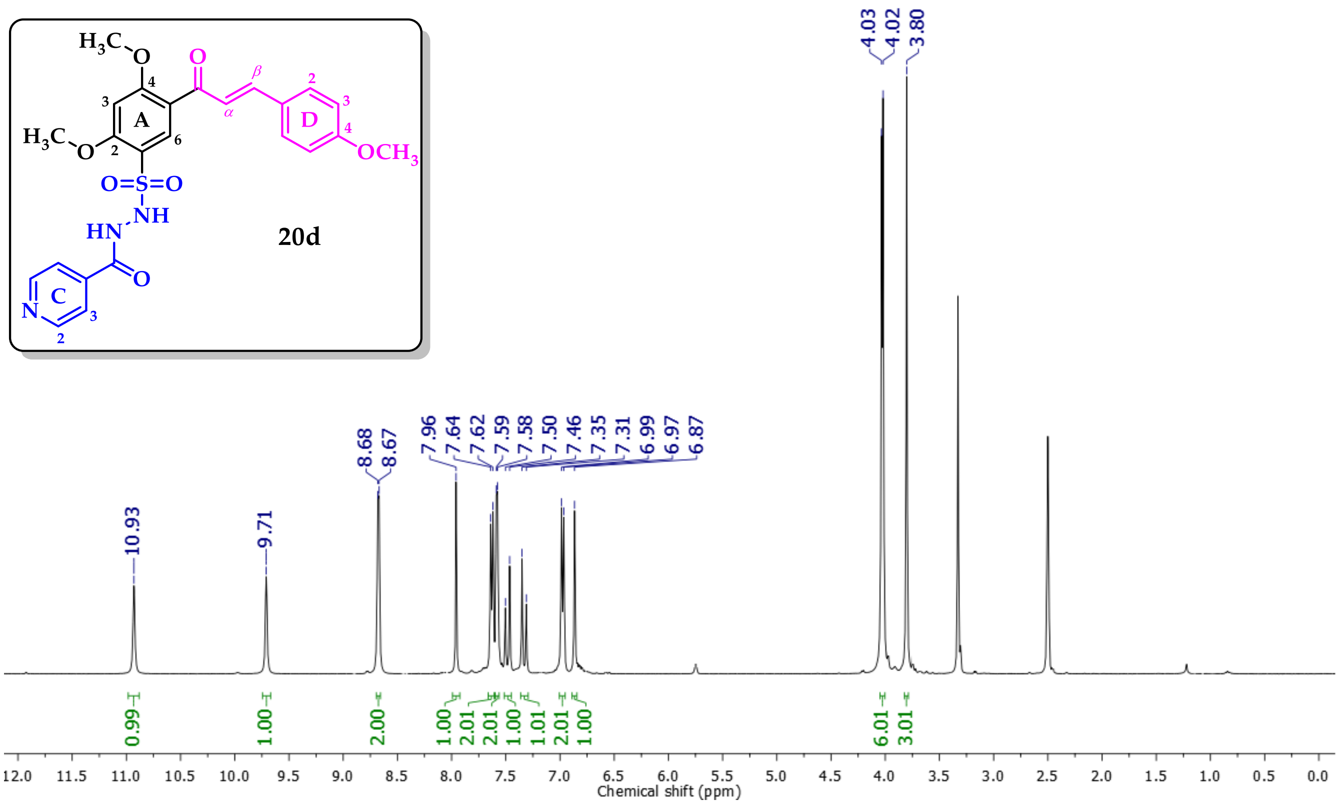


$^{1}$H NMR (400 MHz, DMSO-$d_6$) spectrum of **20d**.

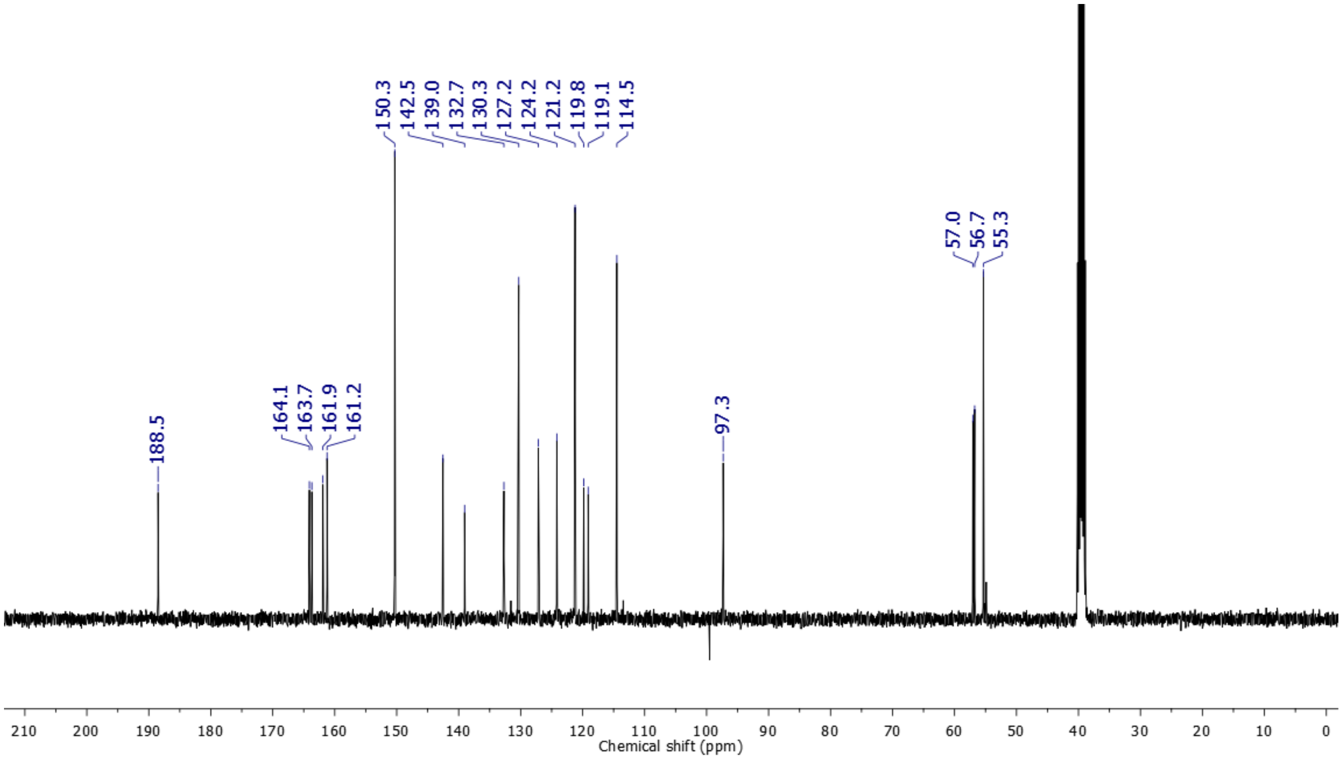


$^{13}$C NMR (101 MHz, DMSO-$d_6$) spectrum of **20d**.

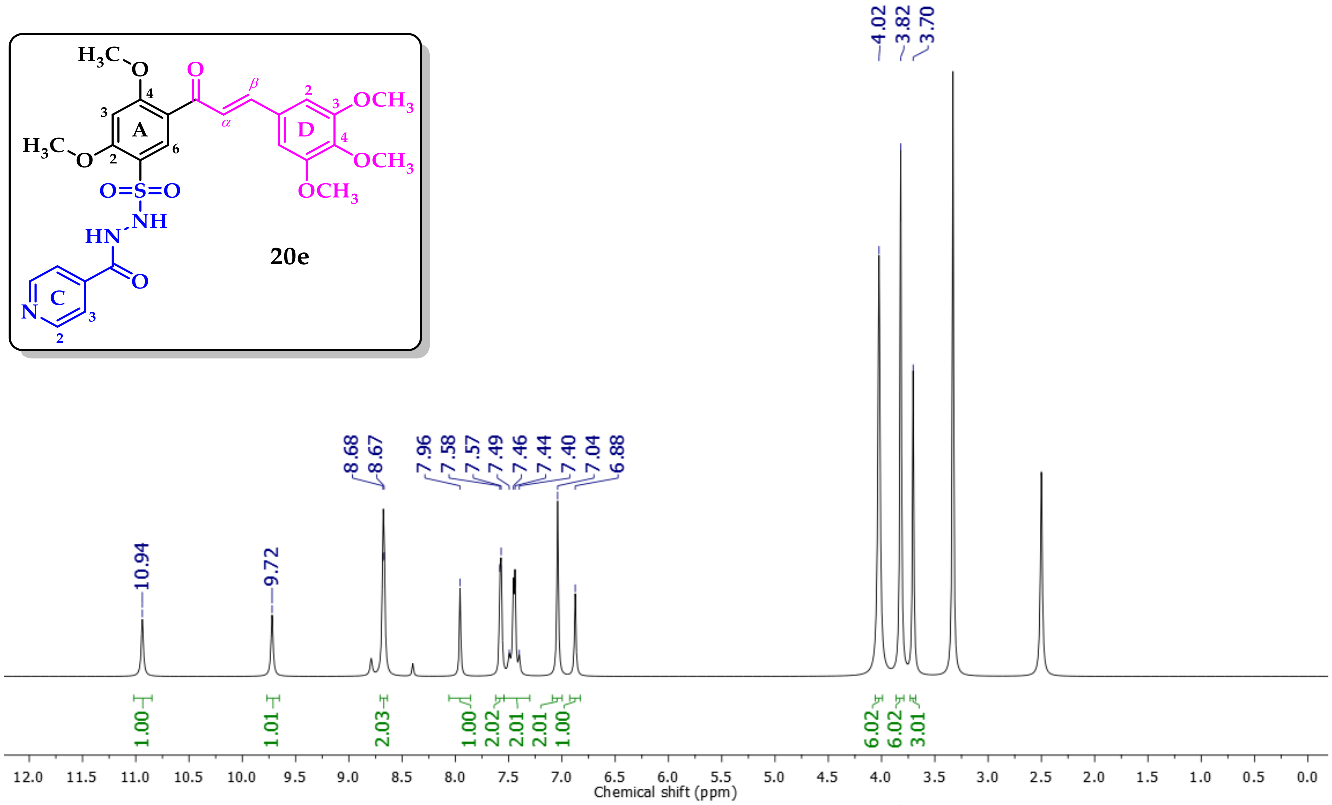


$^{1}$H NMR (400 MHz, DMSO-*d*6) spectrum of **20e**.

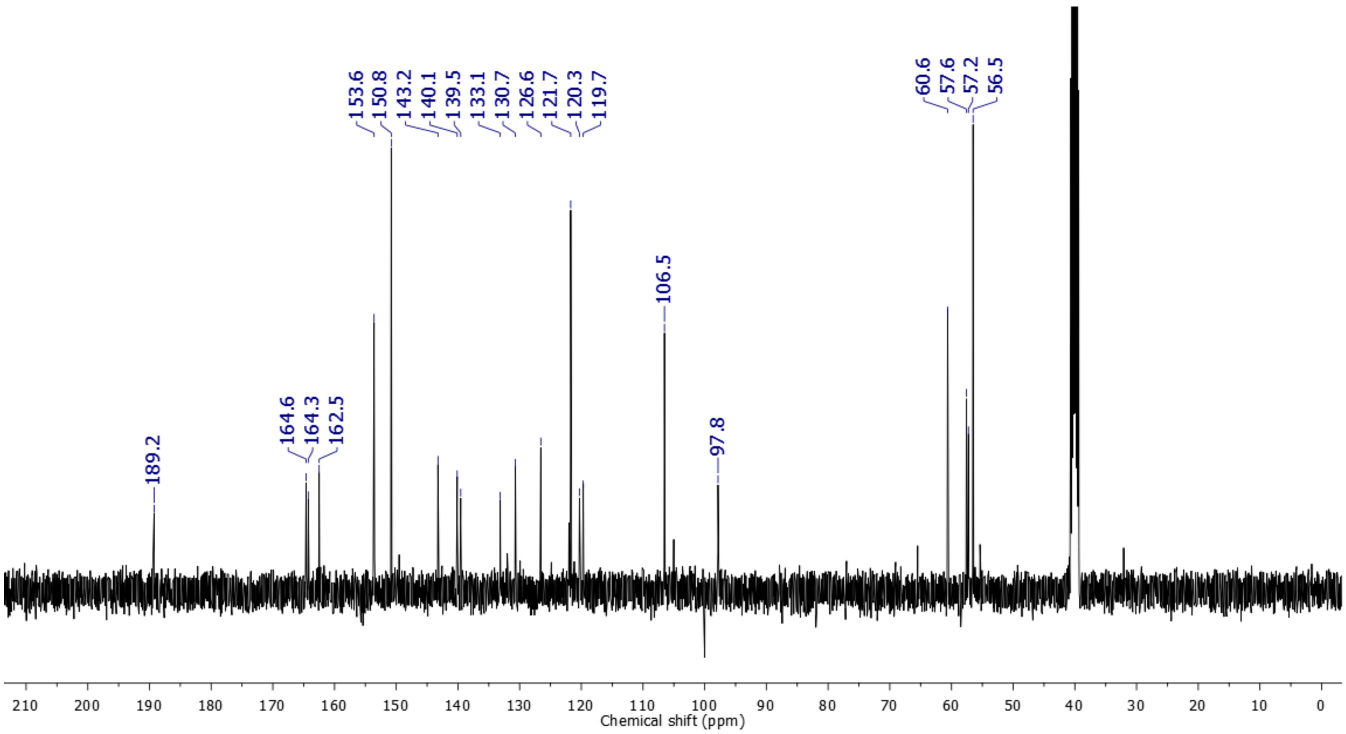


$^{13}$C NMR (101 MHz, DMSO-*d*6) spectrum of **20e**.

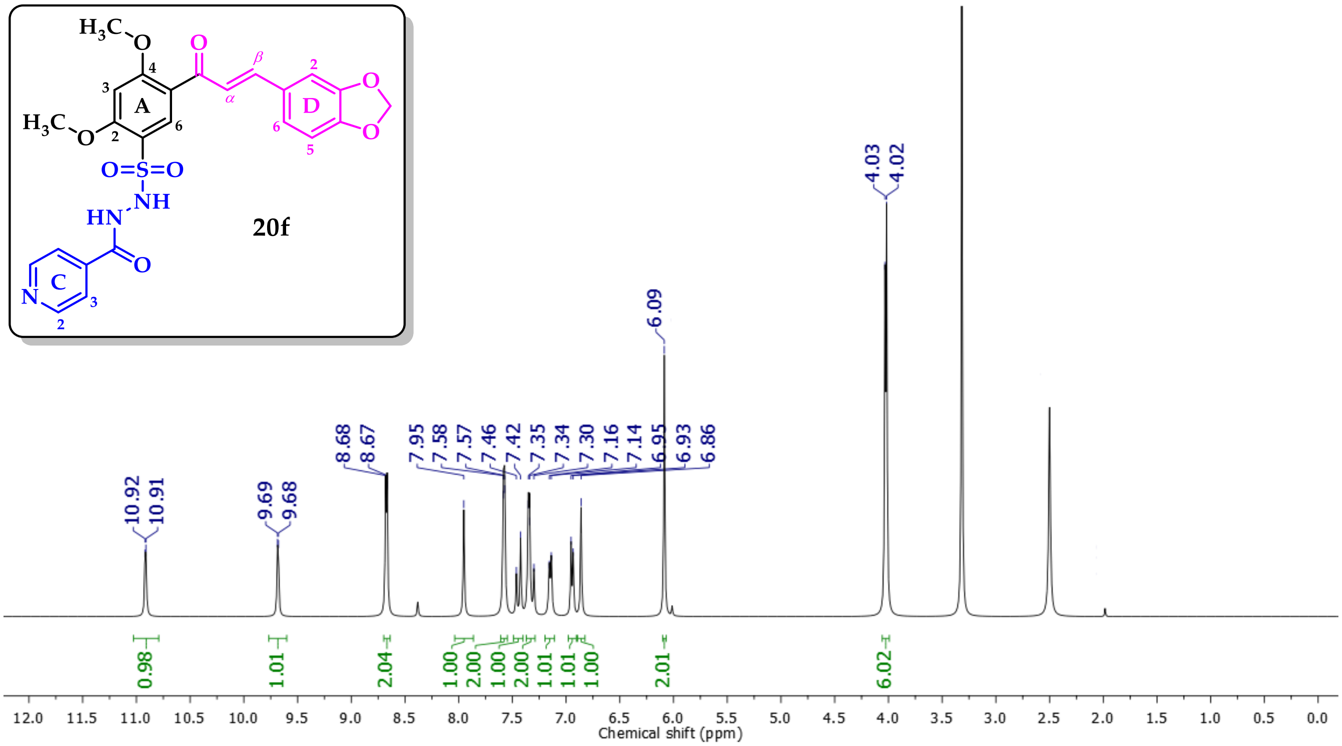


$^{1}H$ NMR (400 MHz, DMSO-*d*$_6$) spectrum of **20f**.

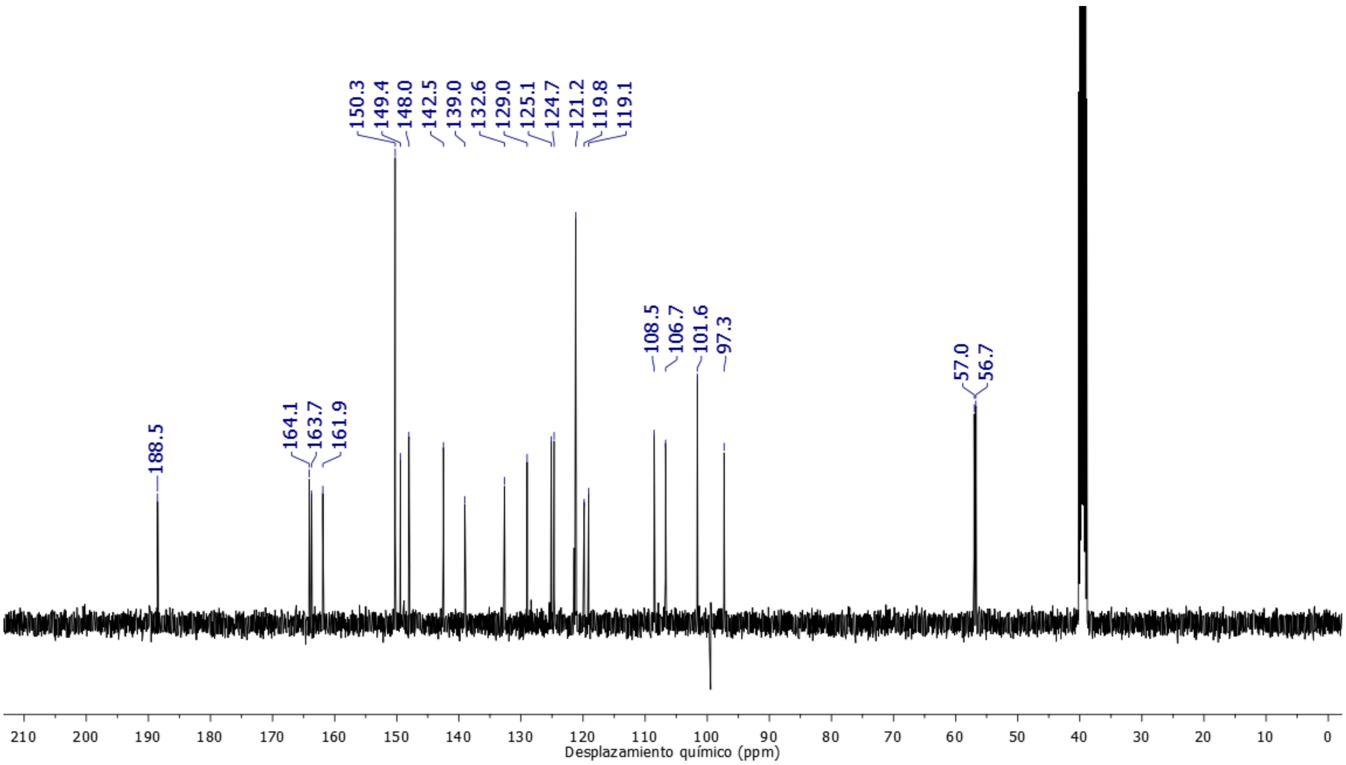


$^{13}C$ NMR (101 MHz, DMSO-*d*$_6$) spectrum of **20f**.

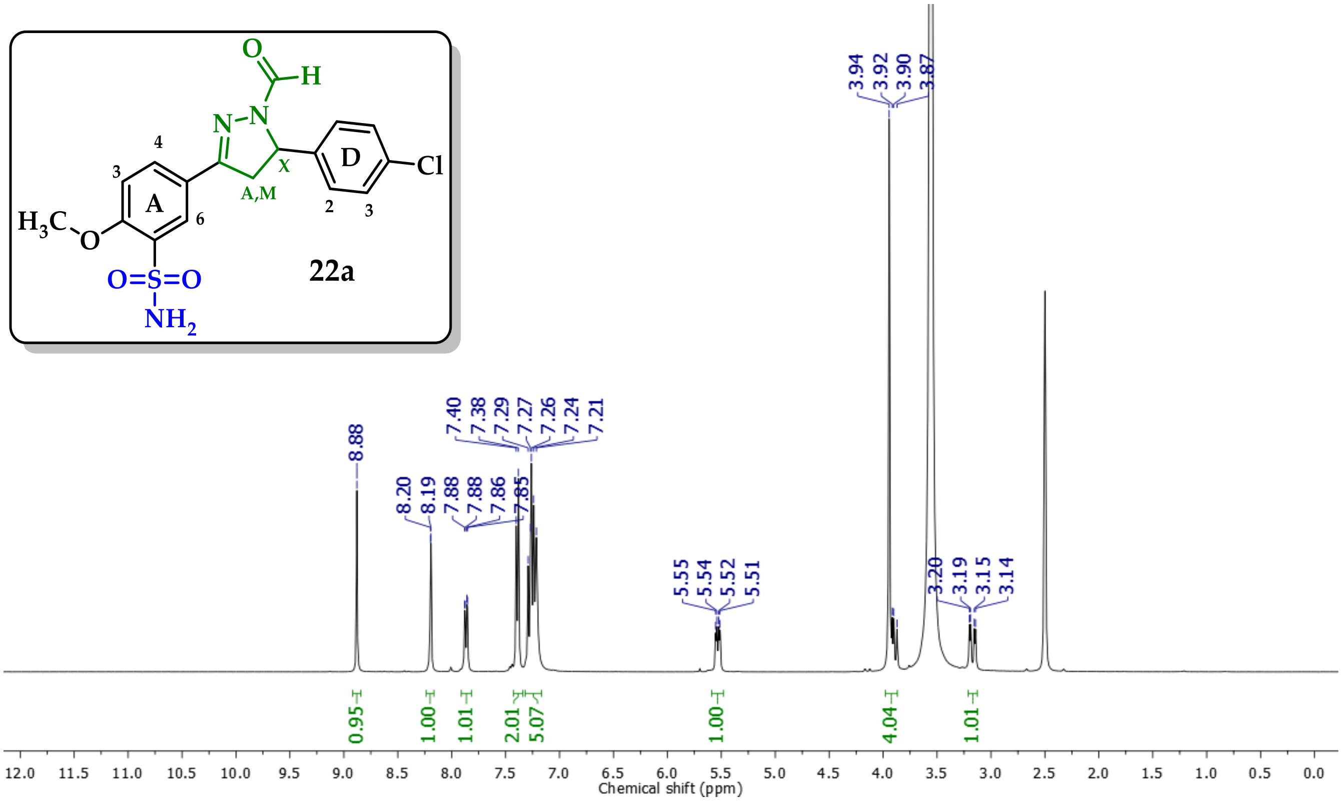


$^{1}$H NMR (400 MHz, DMSO-*d*6) spectrum of **22a**.

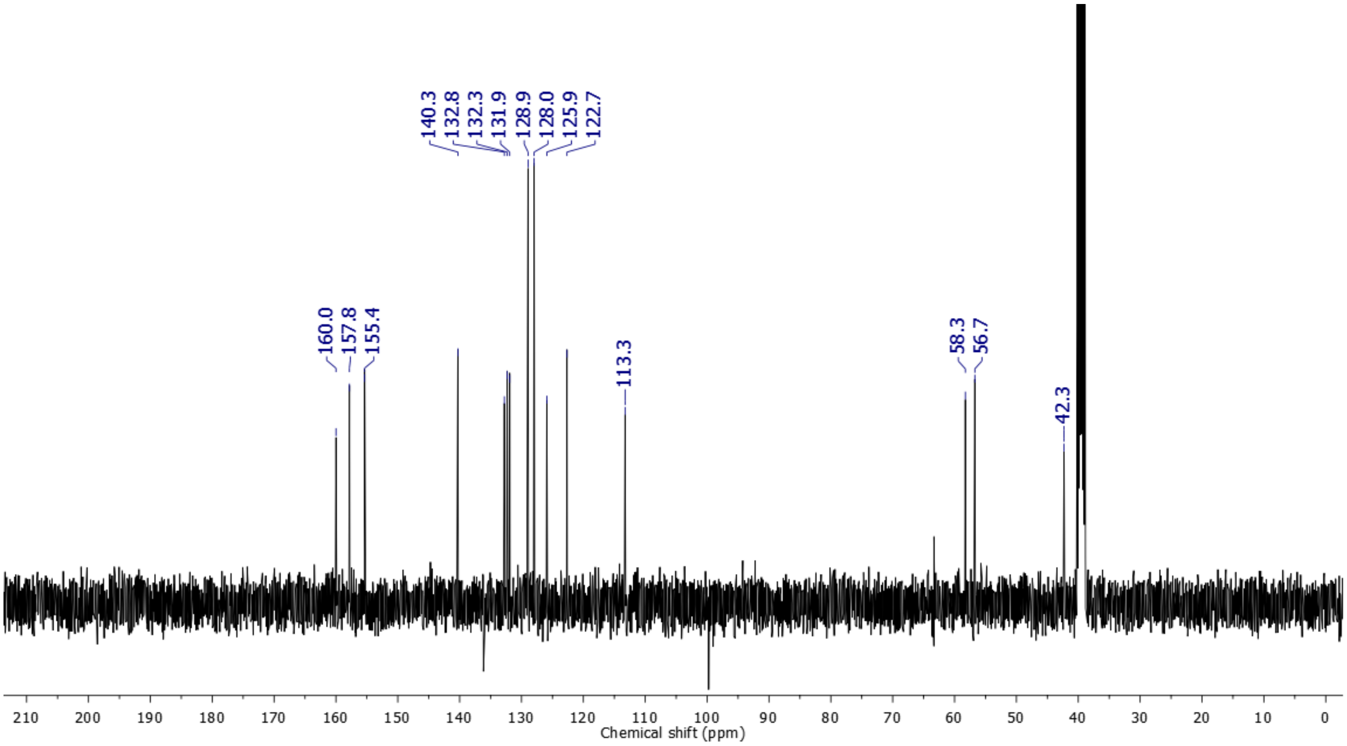


$^{13}$C NMR (101 MHz, DMSO-*d*6) spectrum of **22a**.

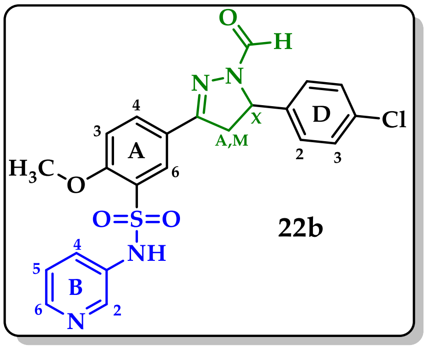


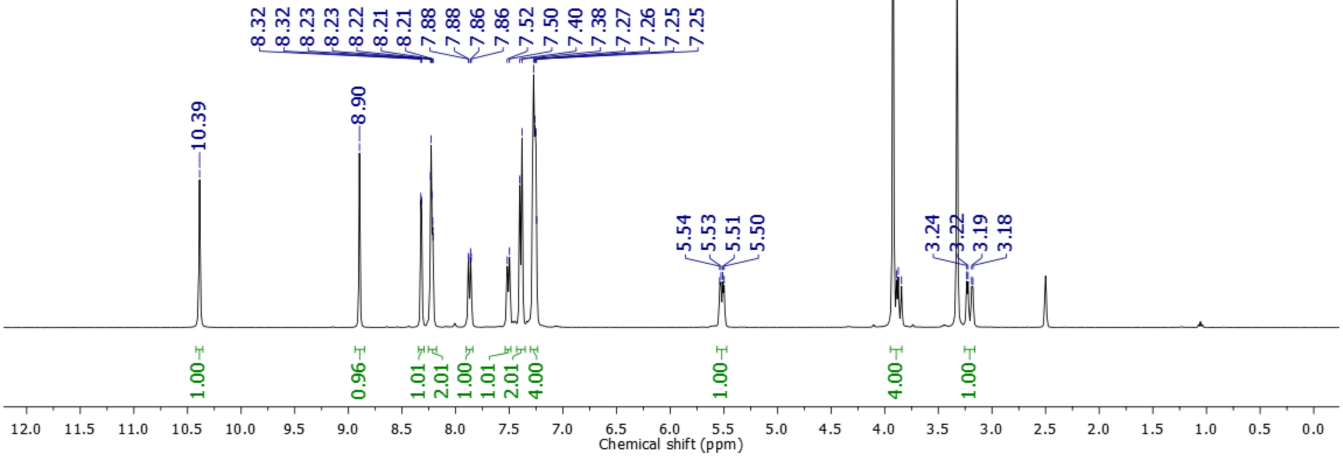


$^{1}$H NMR (400 MHz, DMSO-$d_6$) spectrum of **22b**.

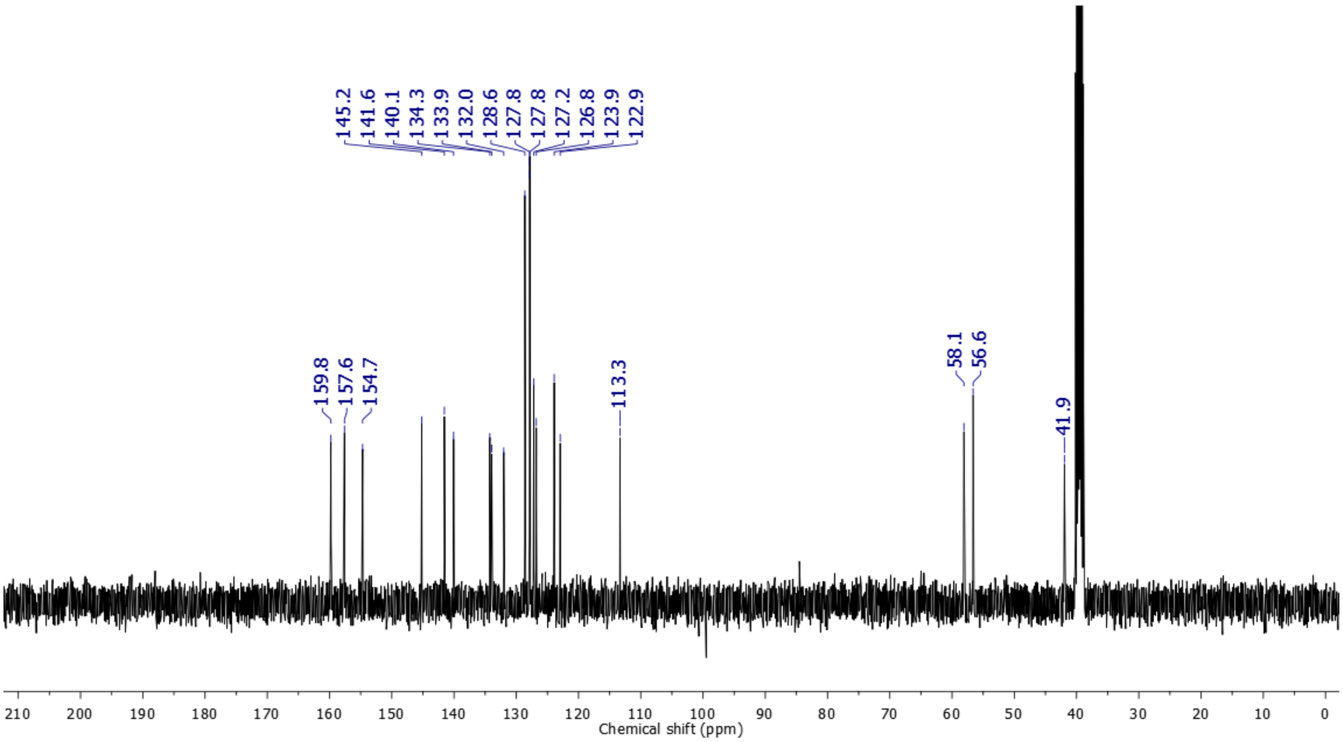


$^{13}$C NMR (101 MHz, DMSO-$d_6$) spectrum of **22b**.

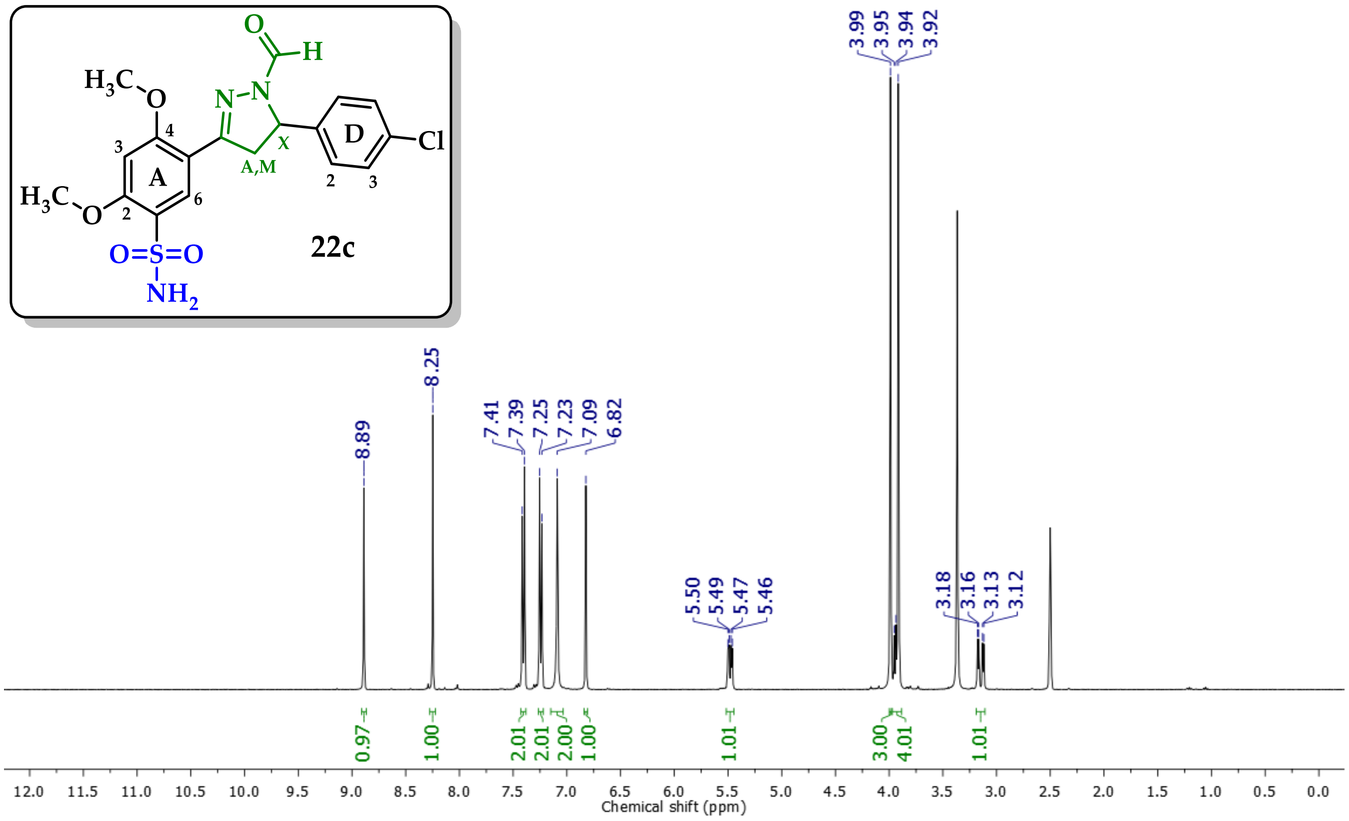


$^{1}H$ NMR (400 MHz, DMSO-$d_6$) spectrum of **22c**.

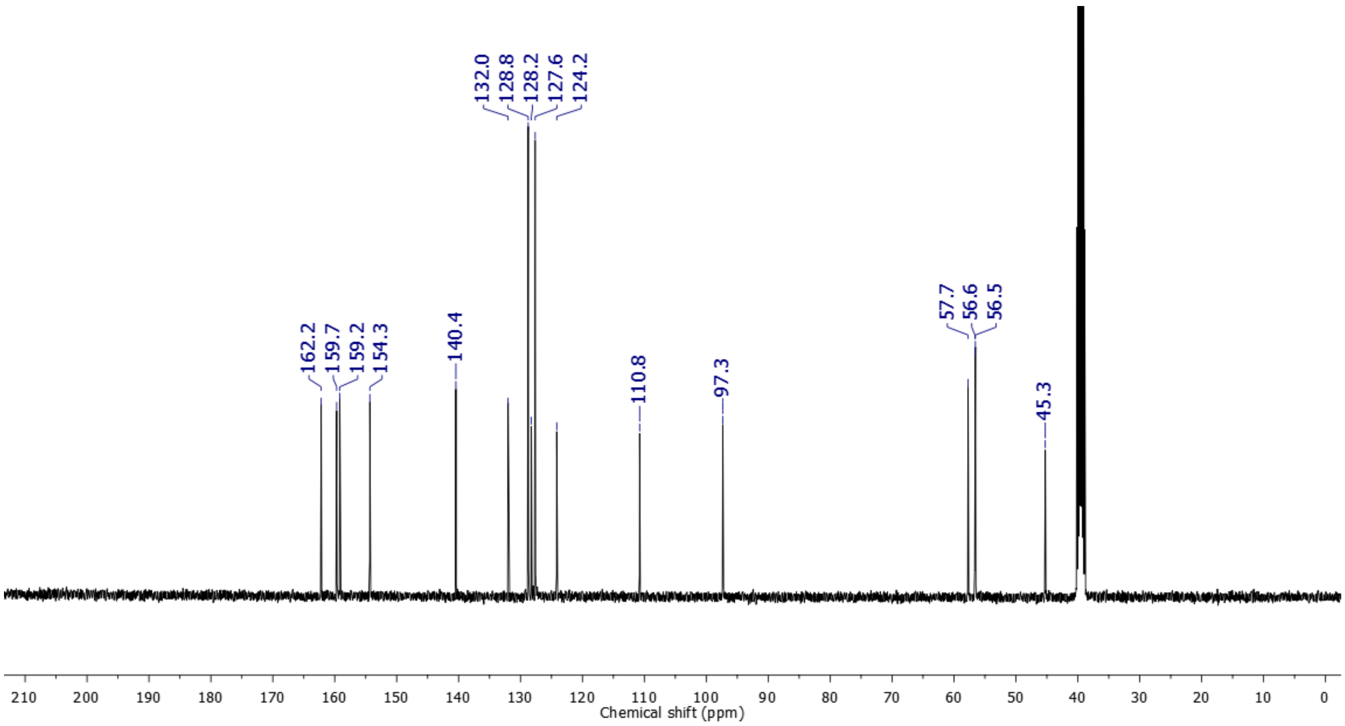


$^{13}C$ NMR (101 MHz, DMSO-$d_6$) spectrum of **22c**.

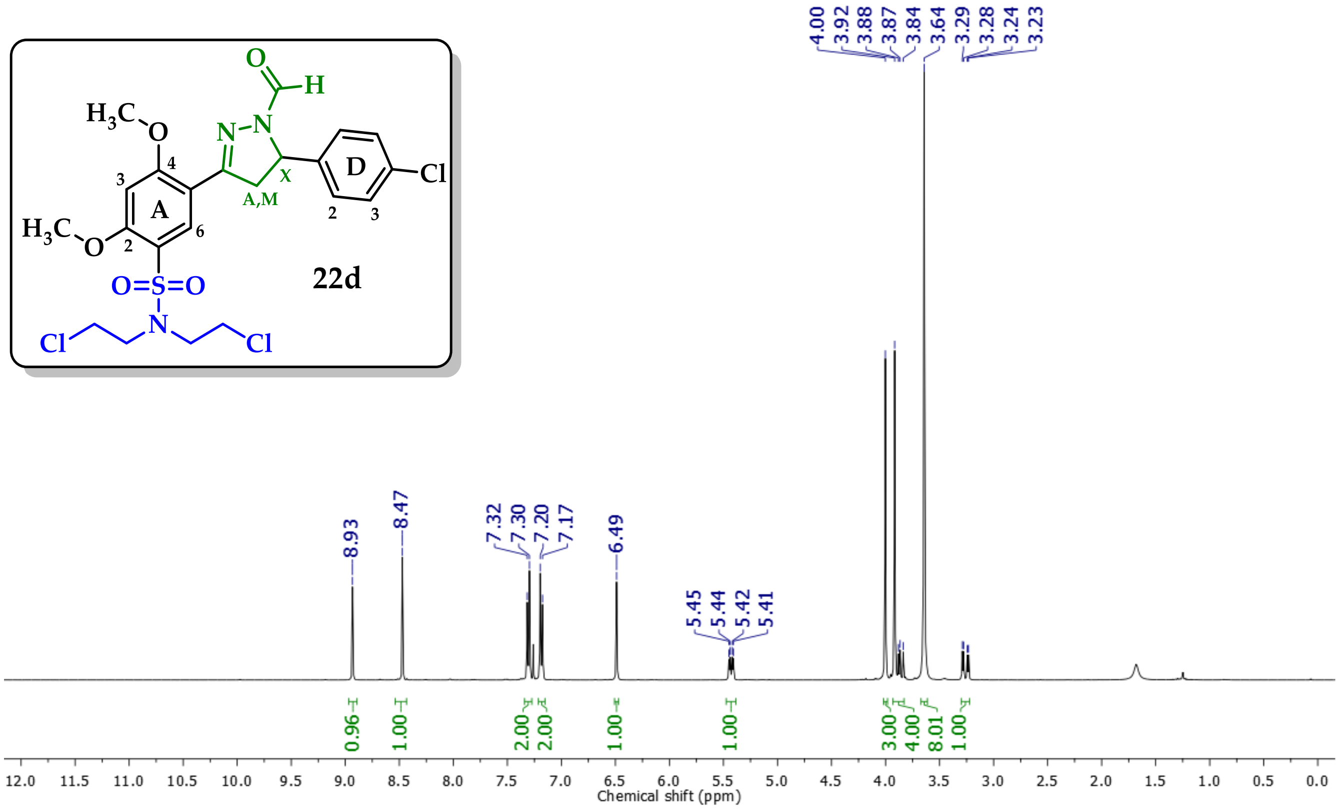


$^{1}$H NMR (400 MHz, CDCl3) spectrum of **22d**.

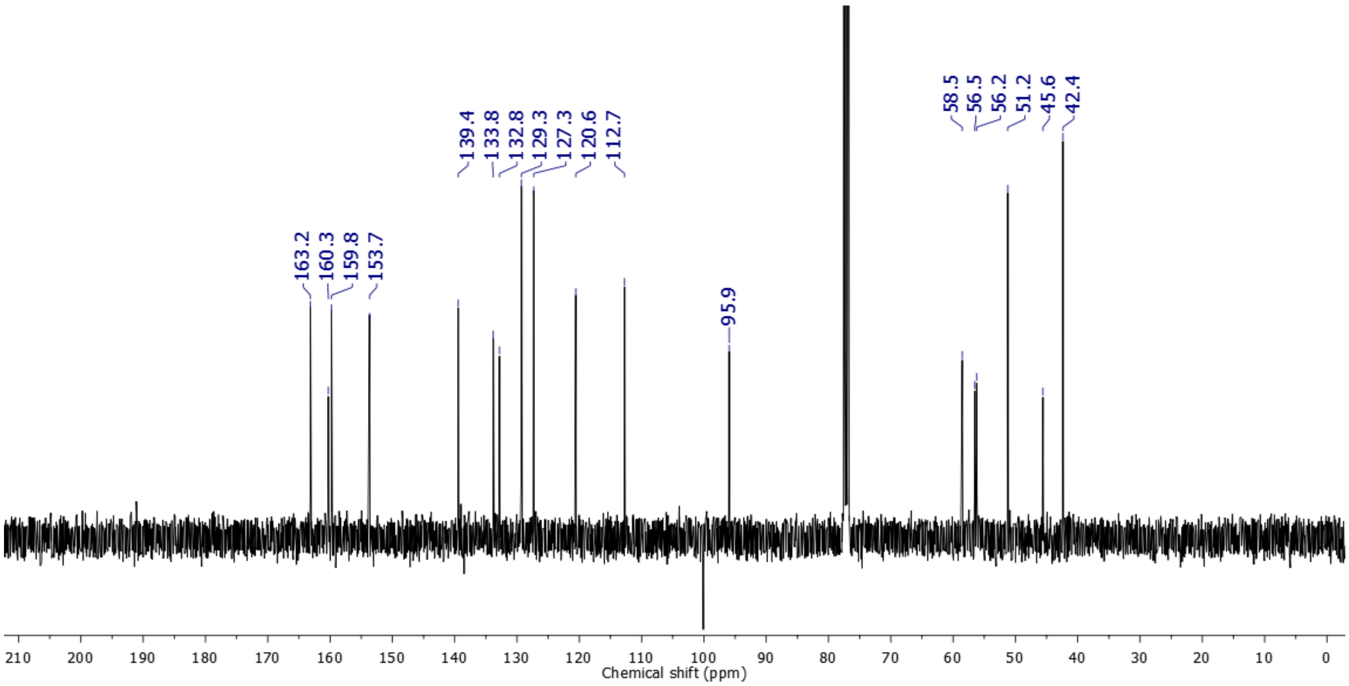


$^{13}$C NMR (101 MHz, CDCl3) spectrum of **22d**.

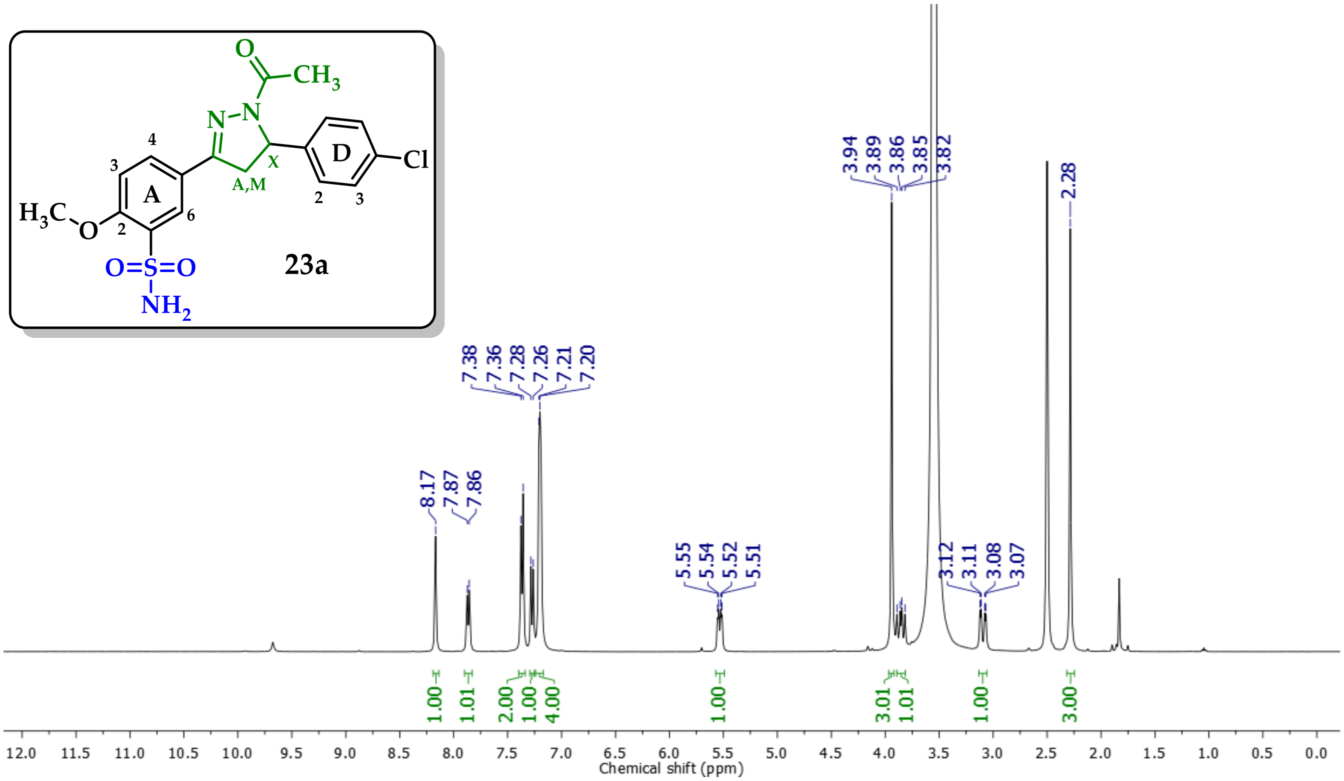


$^{1}$H NMR (400 MHz, DMSO-$d_6$) spectrum of **23a**.

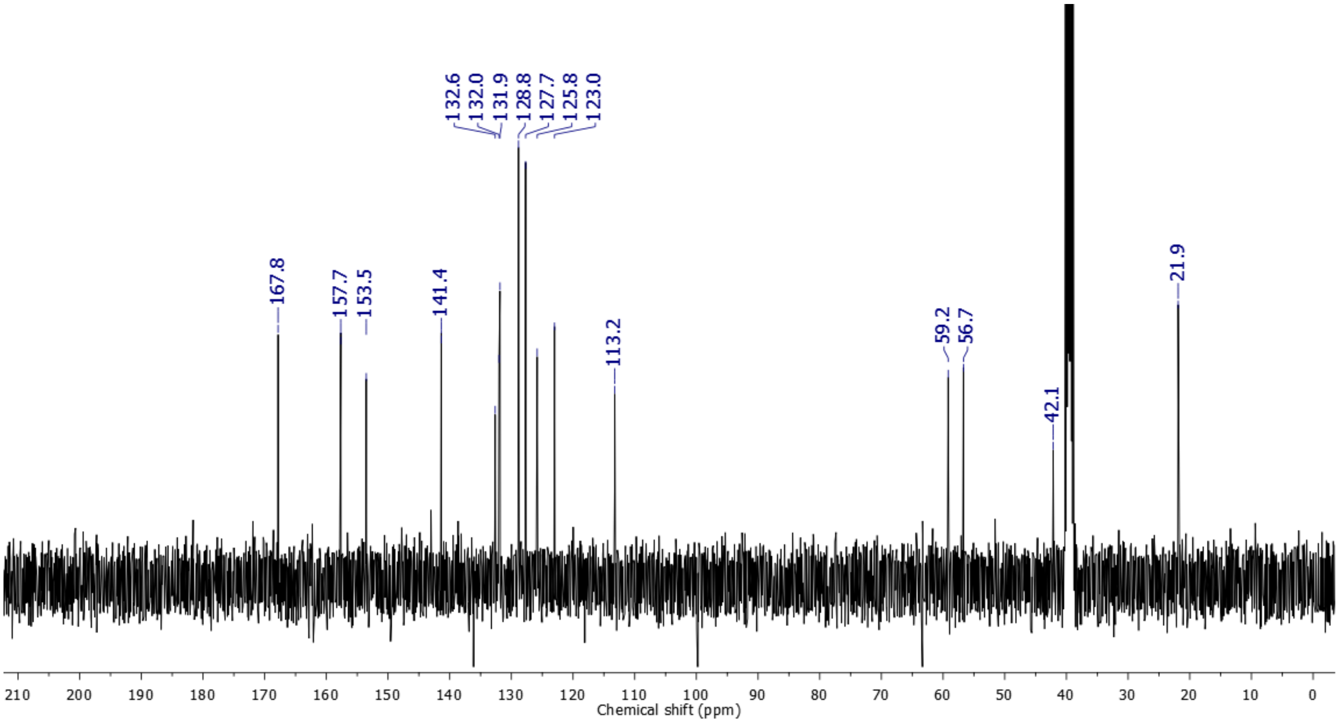


$^{13}$C NMR (101 MHz, DMSO-$d_6$) spectrum of **23a**.

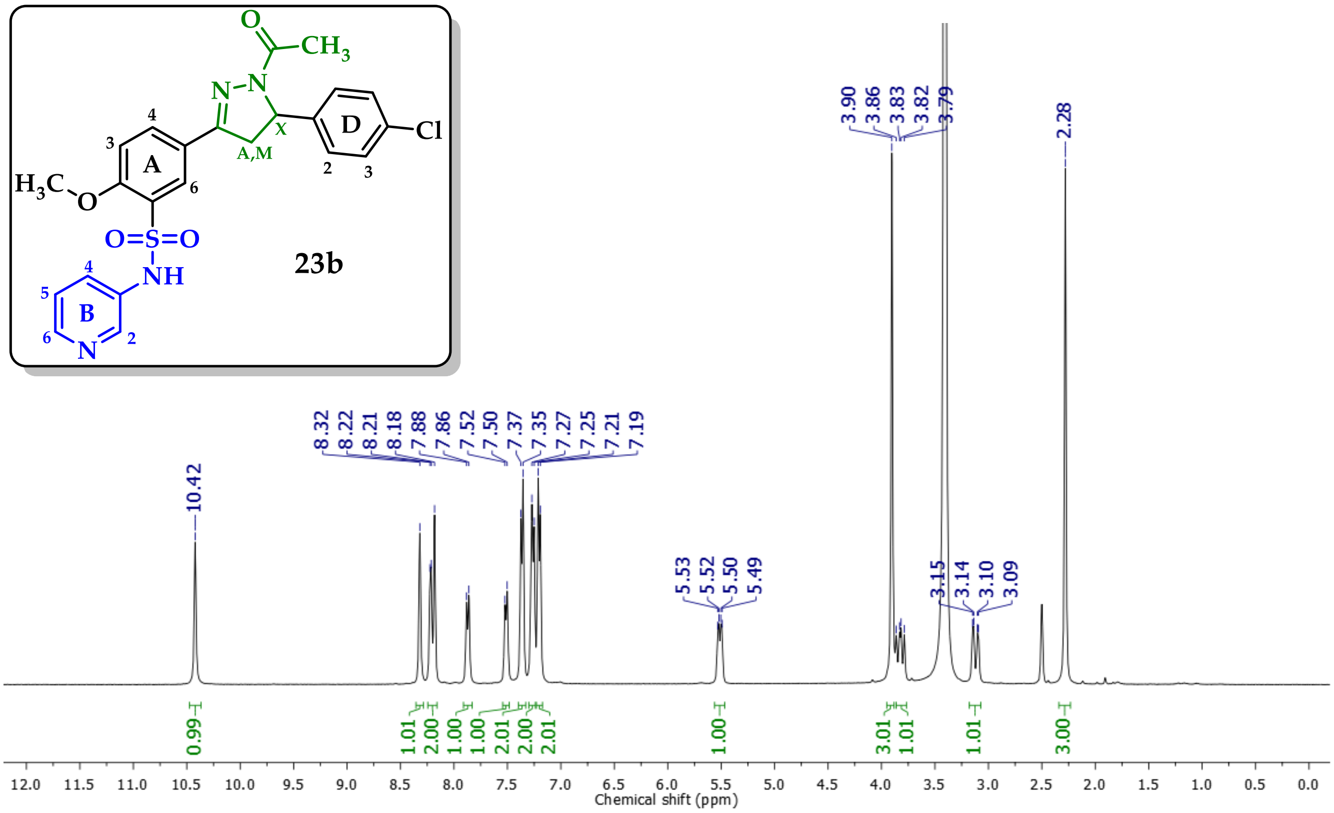


$^{1}$H NMR (400 MHz, DMSO-$d_6$) spectrum of **23b**.

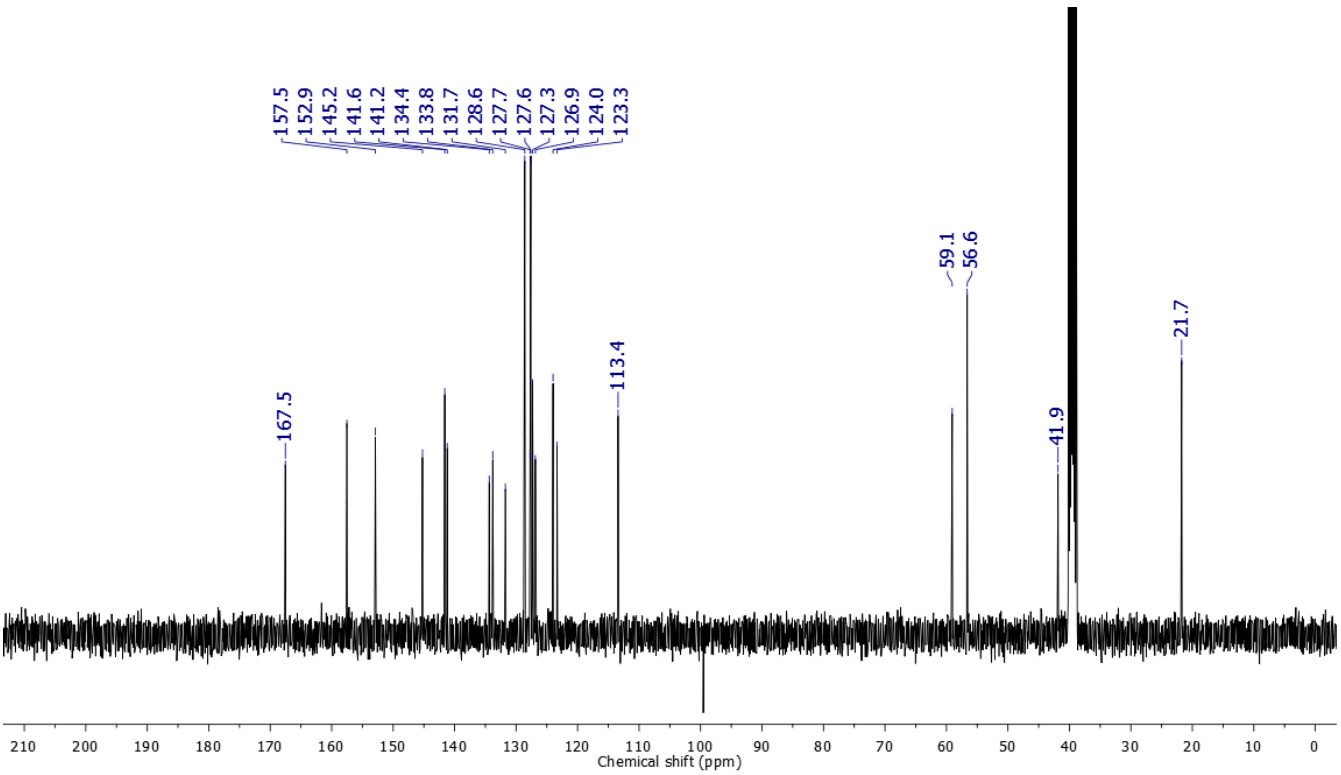


$^{13}$C NMR (101 MHz, DMSO-$d_6$) spectrum of **23b**.

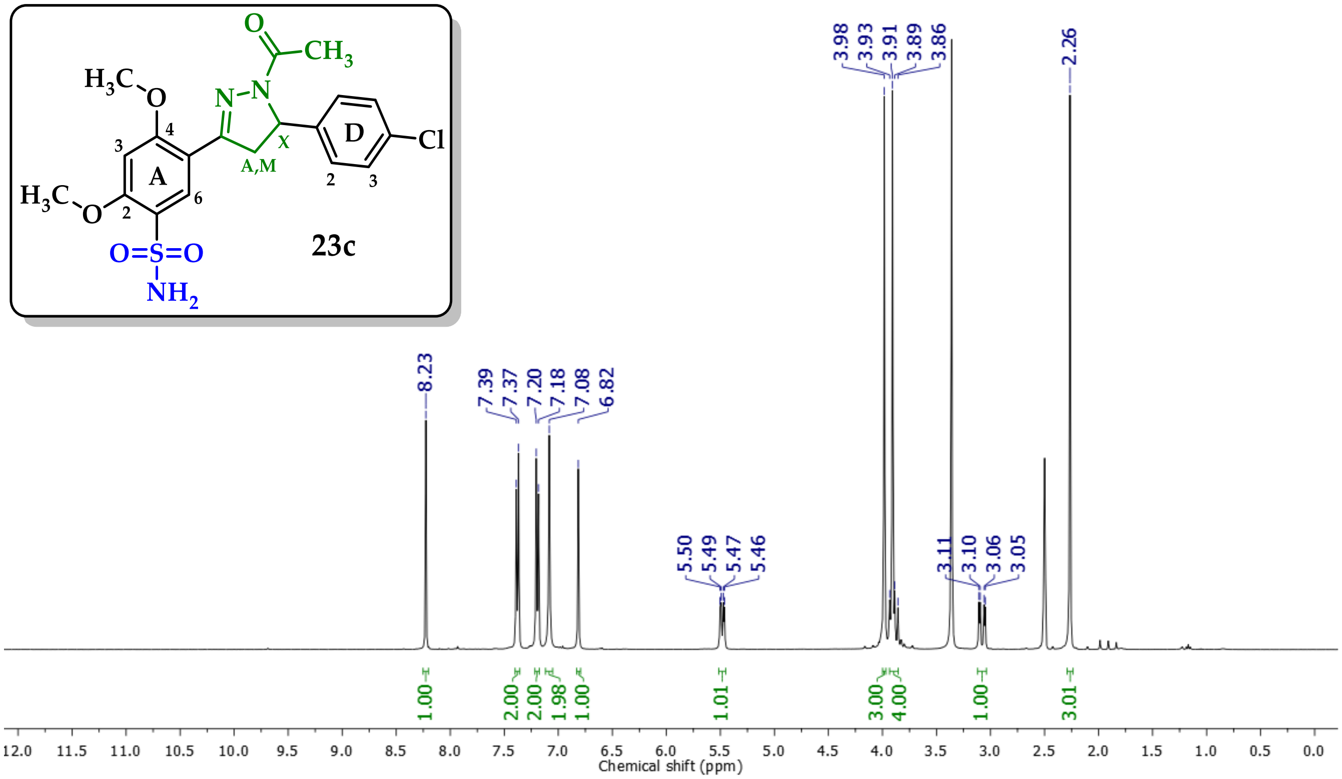


$^{1}H$ NMR (400 MHz, DMSO-$d_6$) spectrum of **23c**.

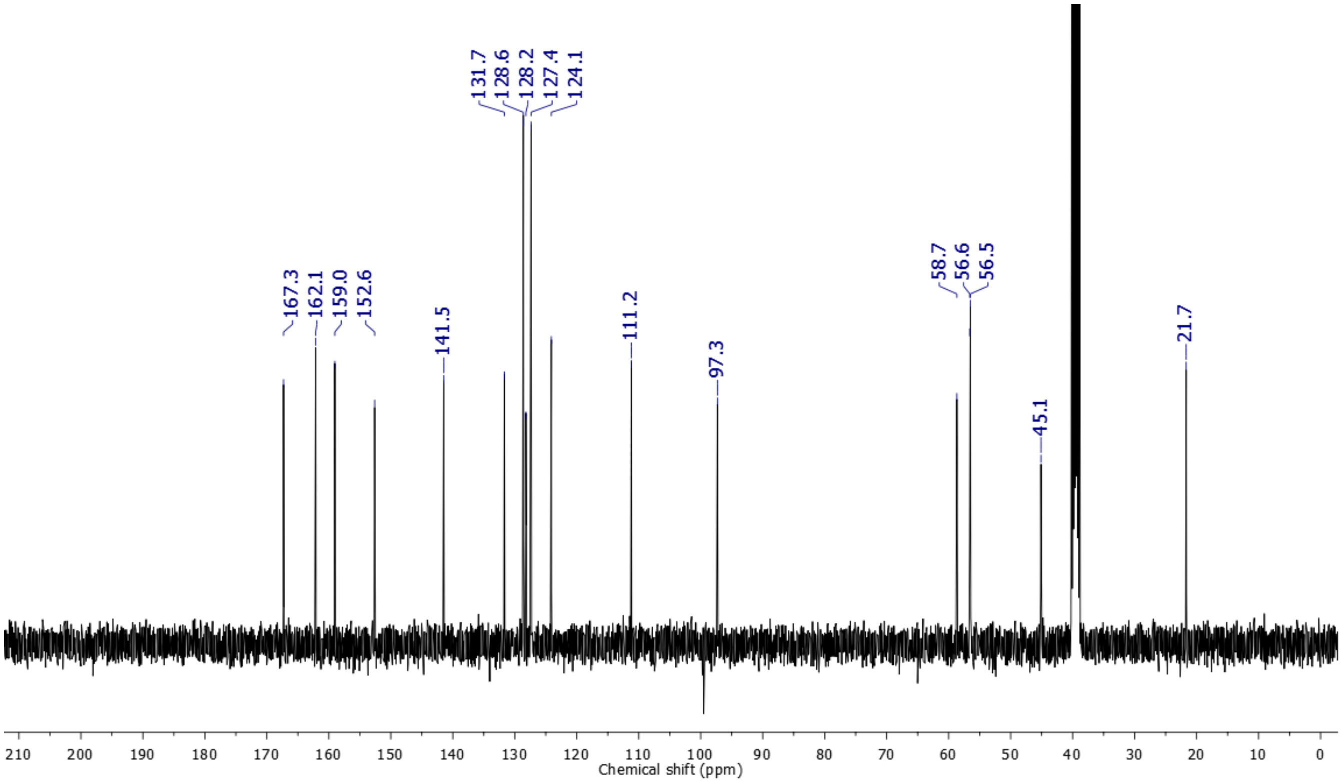


$^{13}C$ NMR (101 MHz, DMSO-$d_6$) spectrum of **23c**.

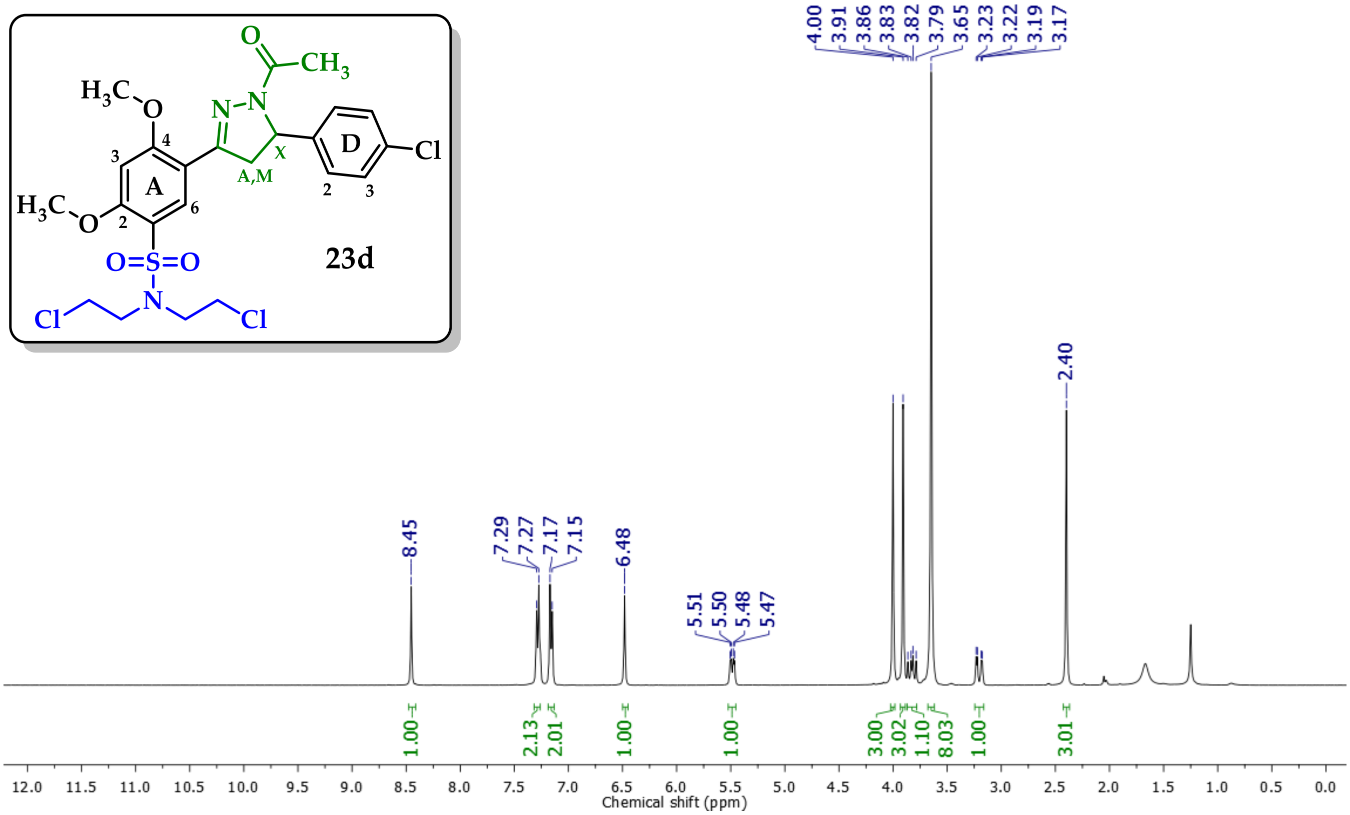


$^{1}H$ NMR (400 MHz, $CDCl_3$) spectrum of **23d**.

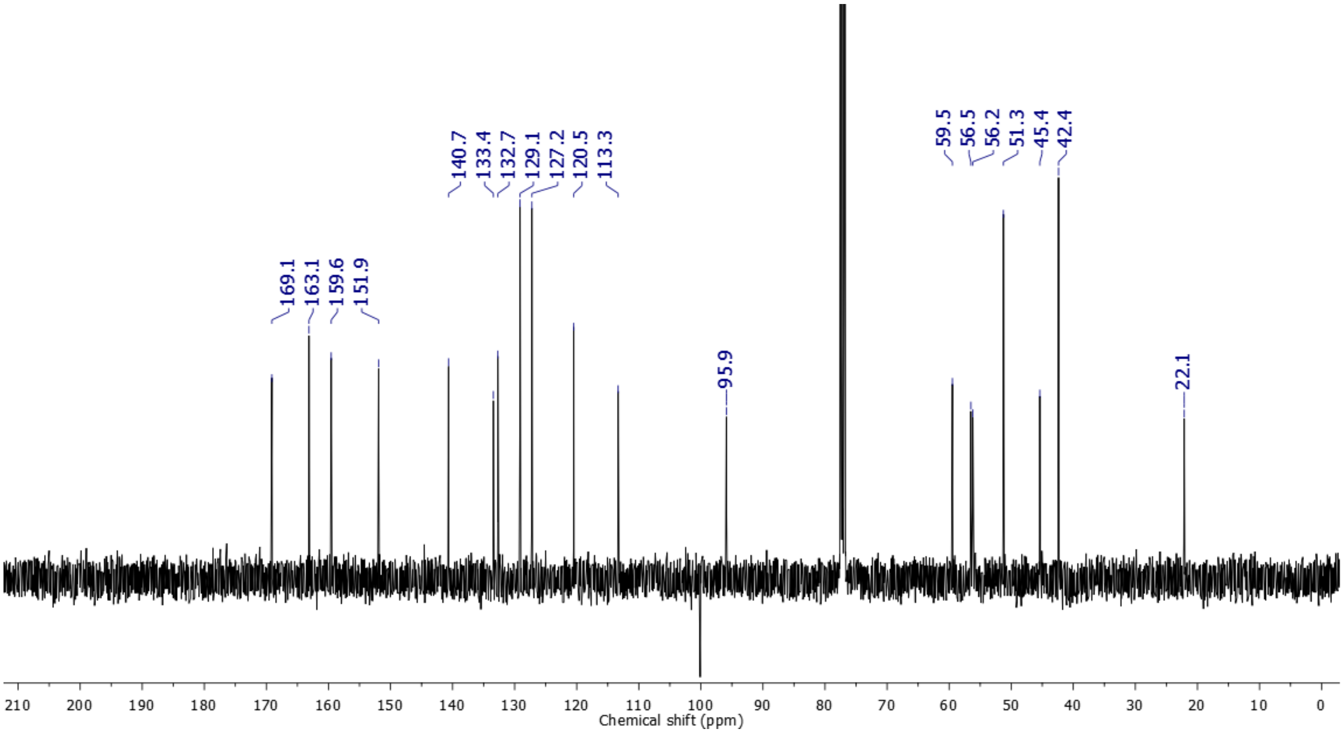


$^{13}C$ NMR (101 MHz, $CDCl_3$) spectrum of **23d**.

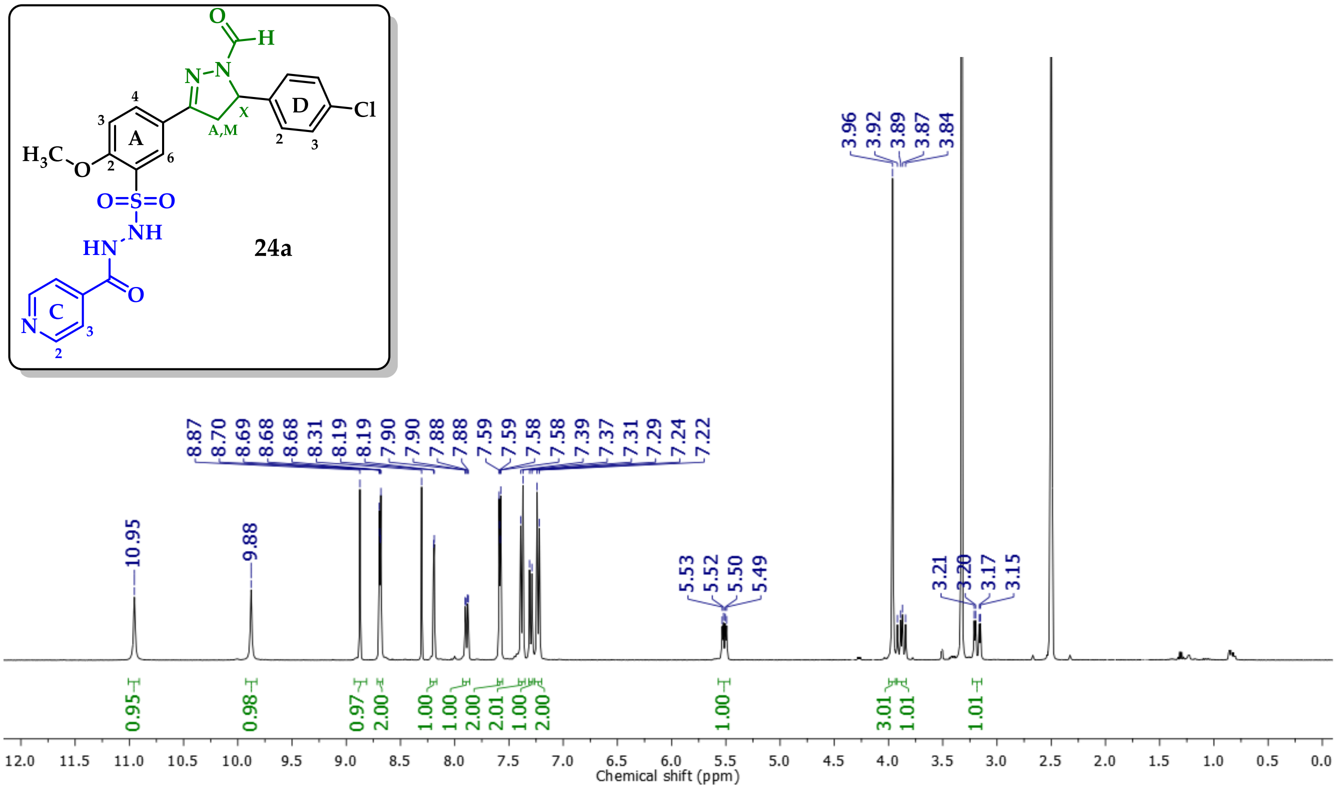


$^{1}$H NMR (400 MHz, DMSO-$d_6$) spectrum of **24a**.

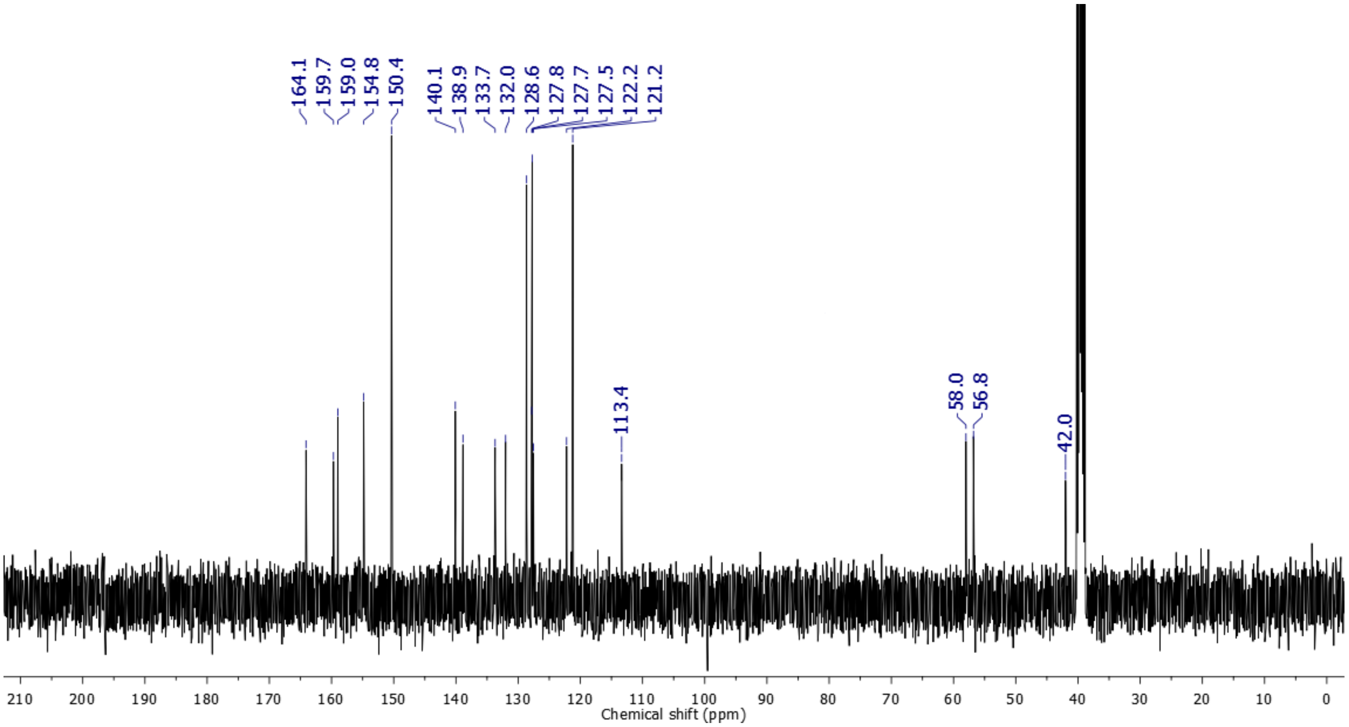


$^{13}$C NMR (101 MHz, DMSO-$d_6$) spectrum of **24a**.

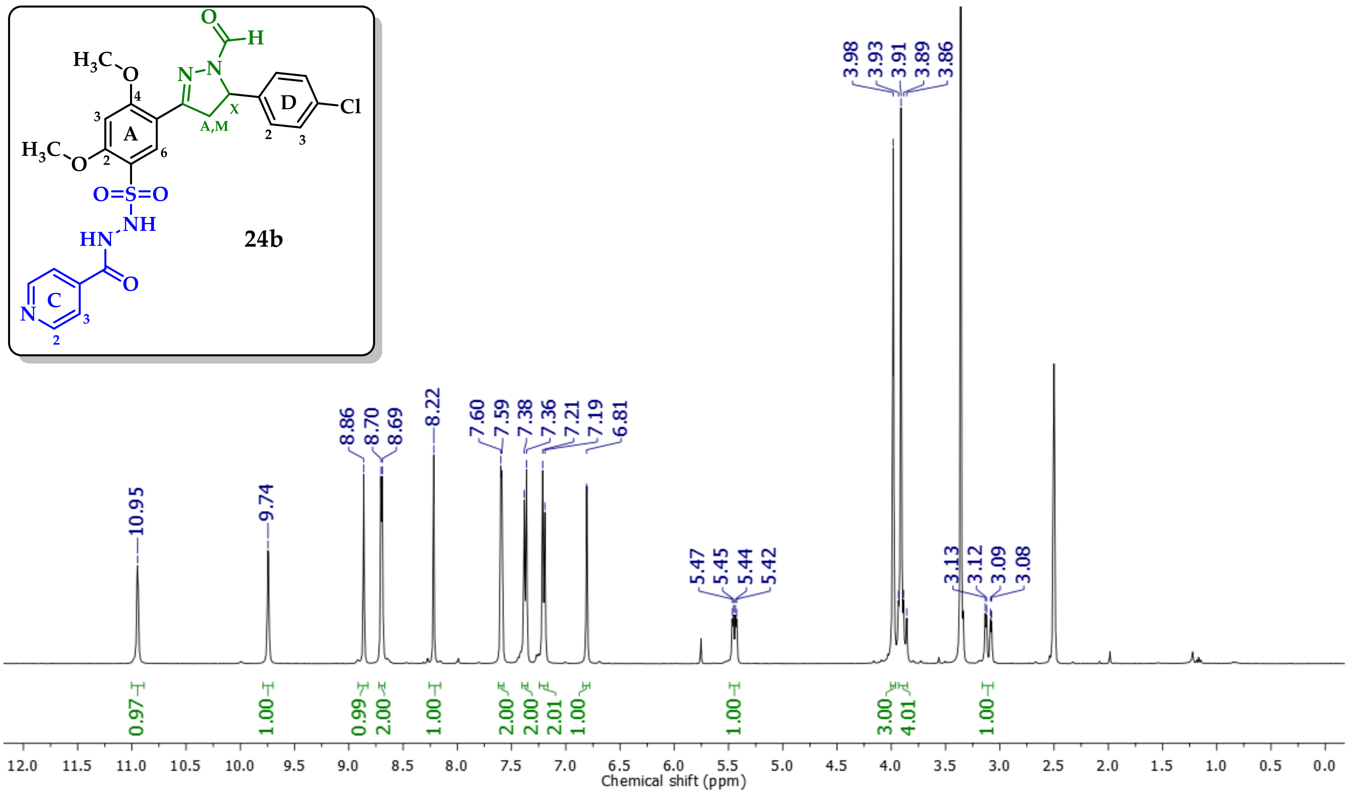


$^{1}H$ NMR (400 MHz, DMSO-$d_6$) spectrum of **24b**.

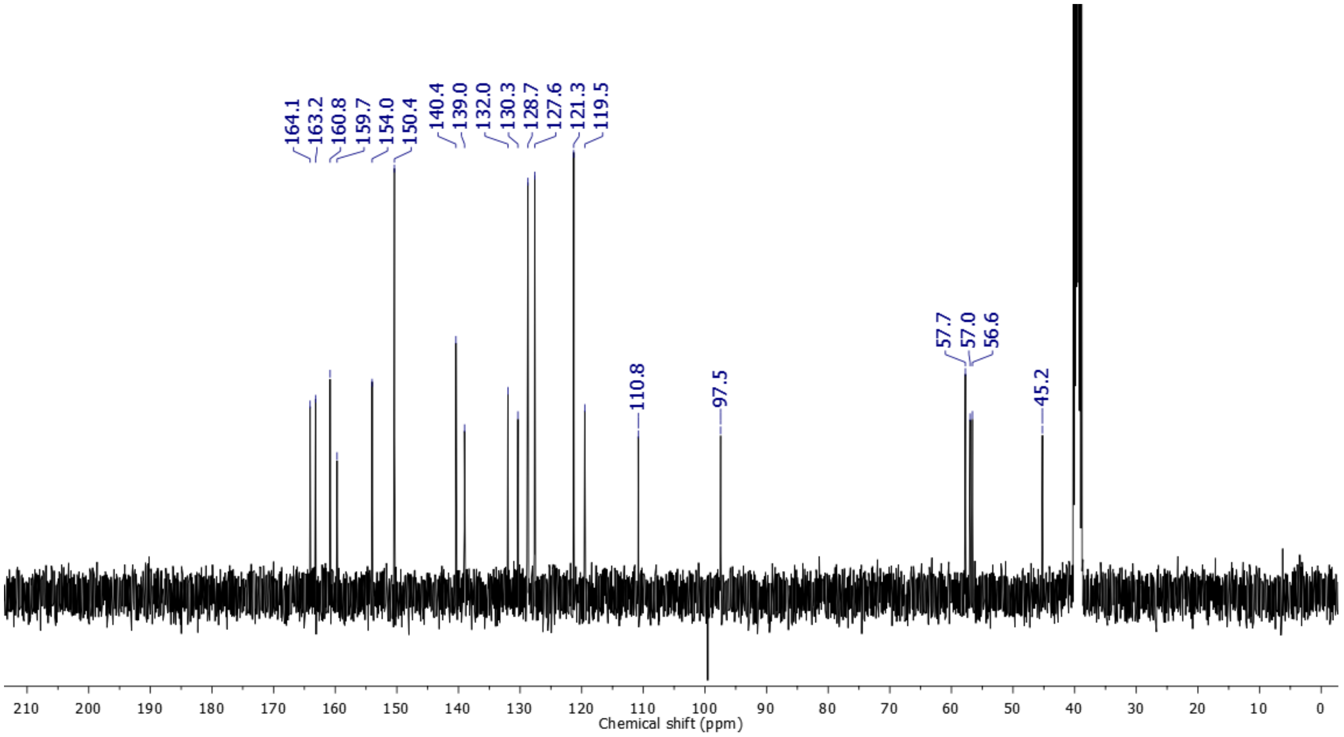


$^{13}C$ NMR (101 MHz, DMSO-$d_6$) spectrum of **24b**.

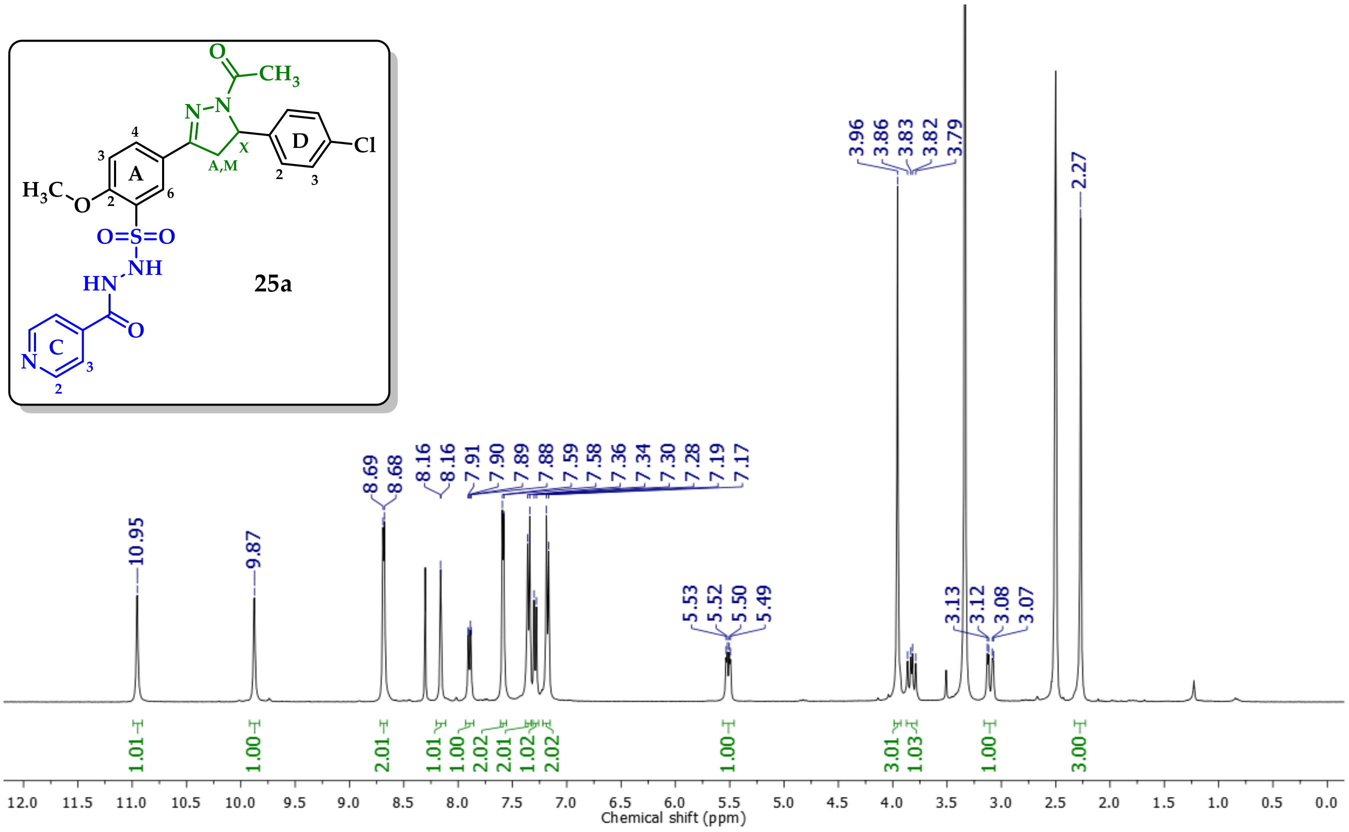


$^{1}$H NMR (400 MHz, DMSO-$d_6$) spectrum of **25a**.

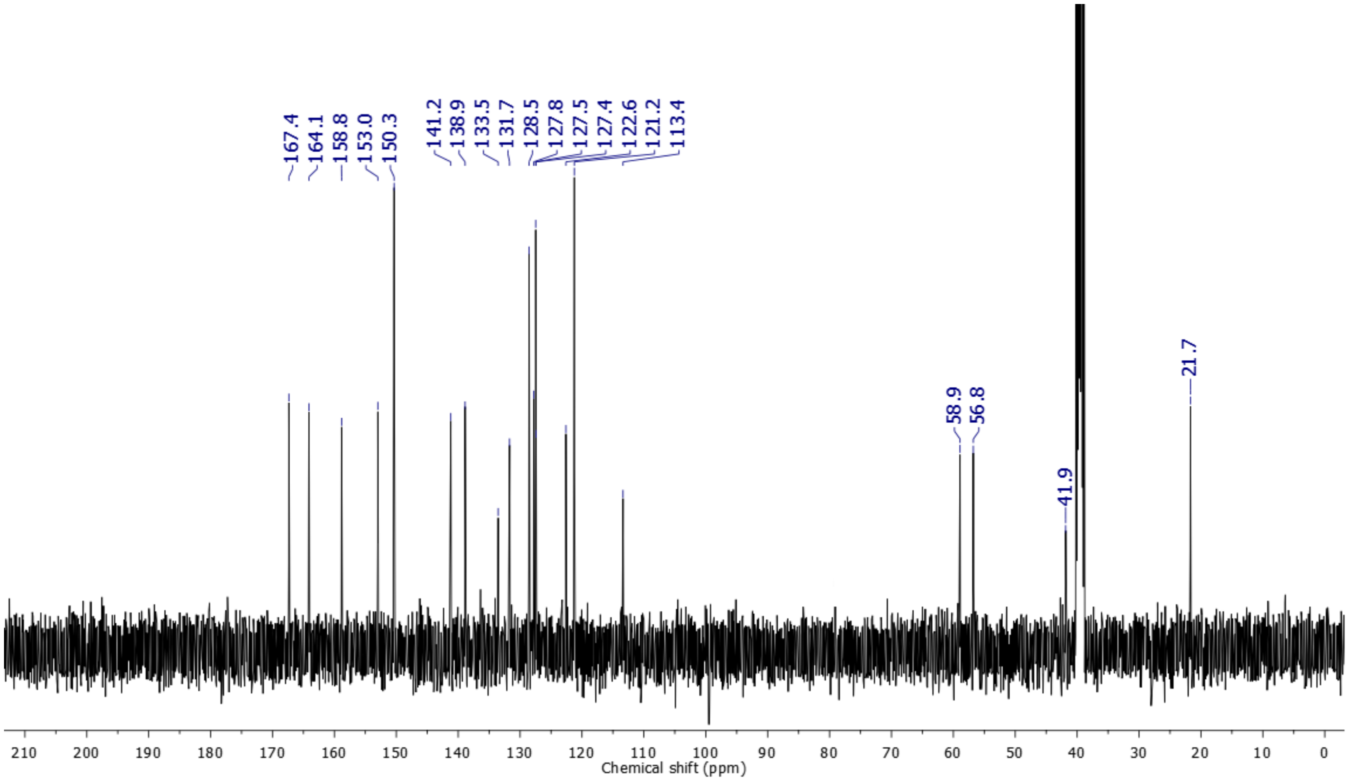

$^{13}$C NMR (101 MHz, DMSO-$d_6$) spectrum of **25a**.

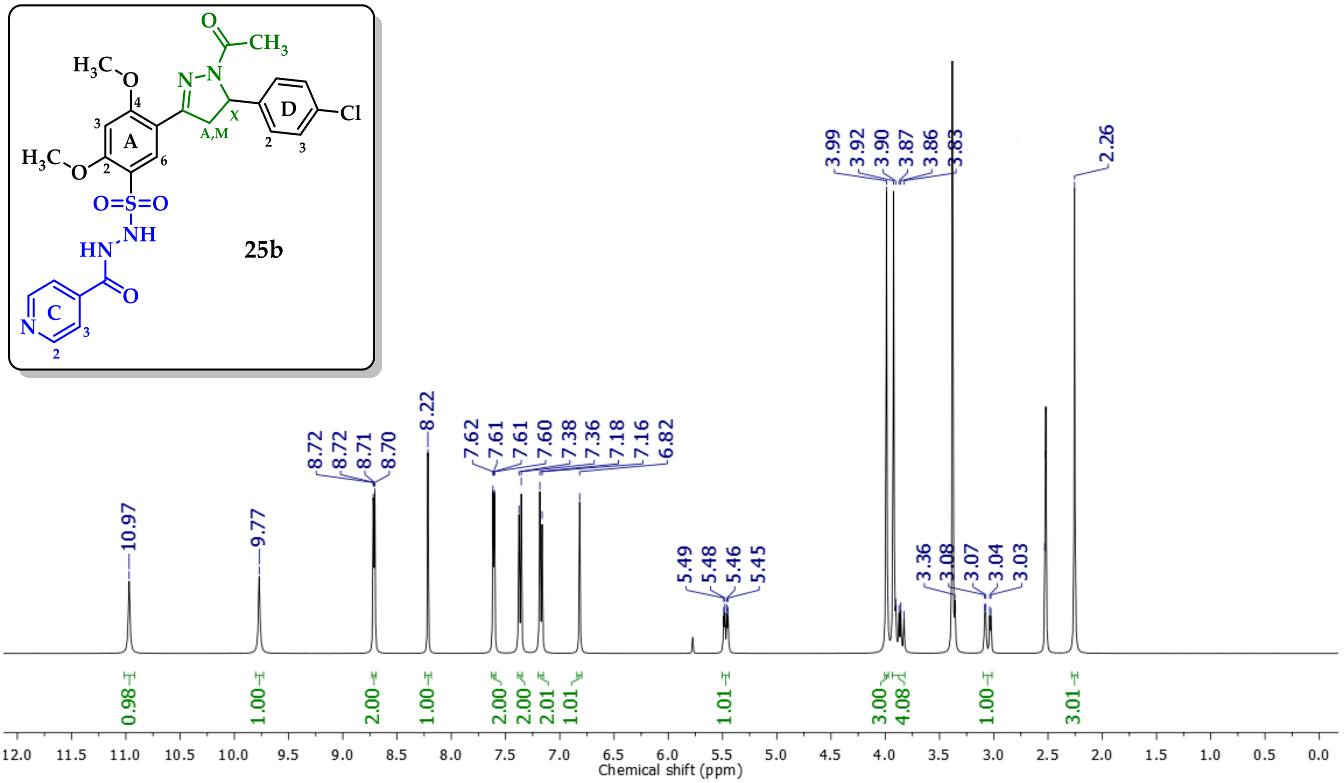


$^{1}$H NMR (400 MHz, DMSO-$d_6$) spectrum of **25b**.

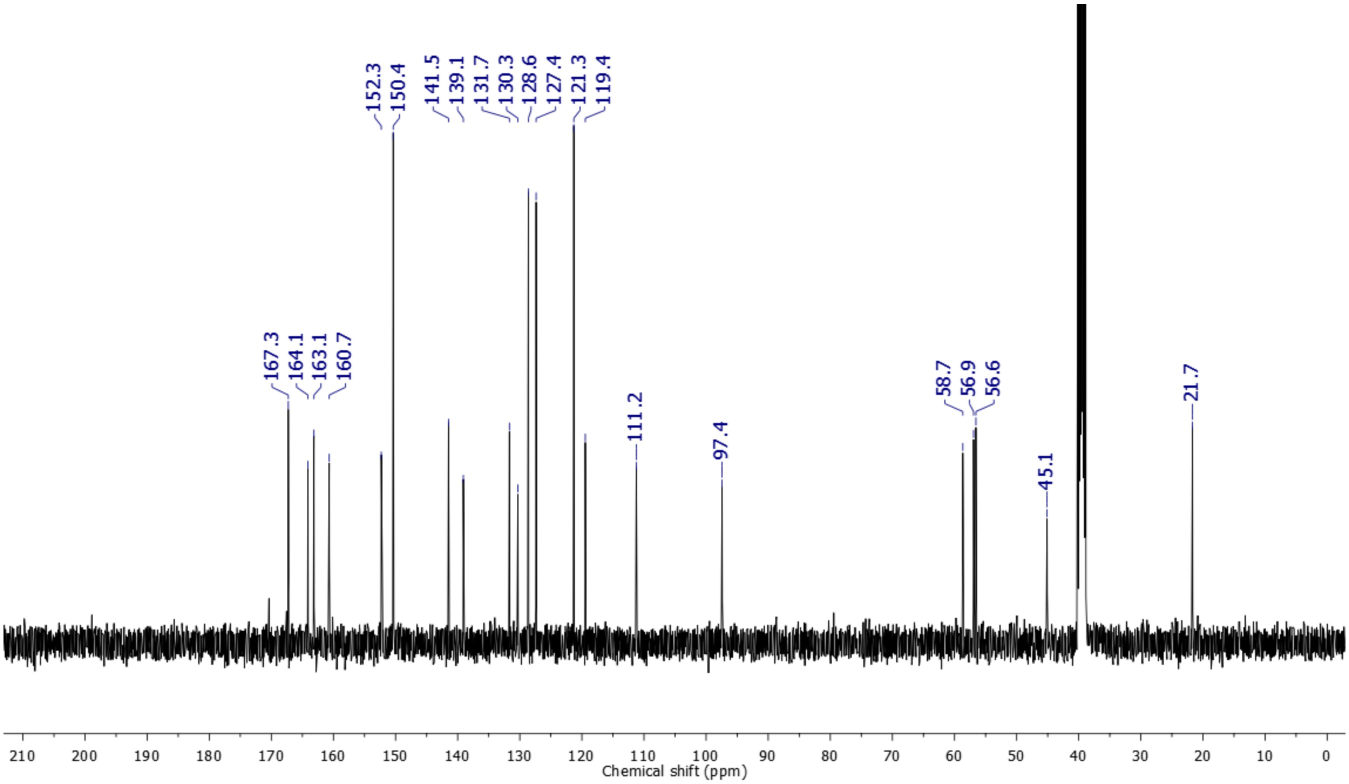

$^{13}$C NMR (101 MHz, DMSO-$d_6$) spectrum of **25b**.

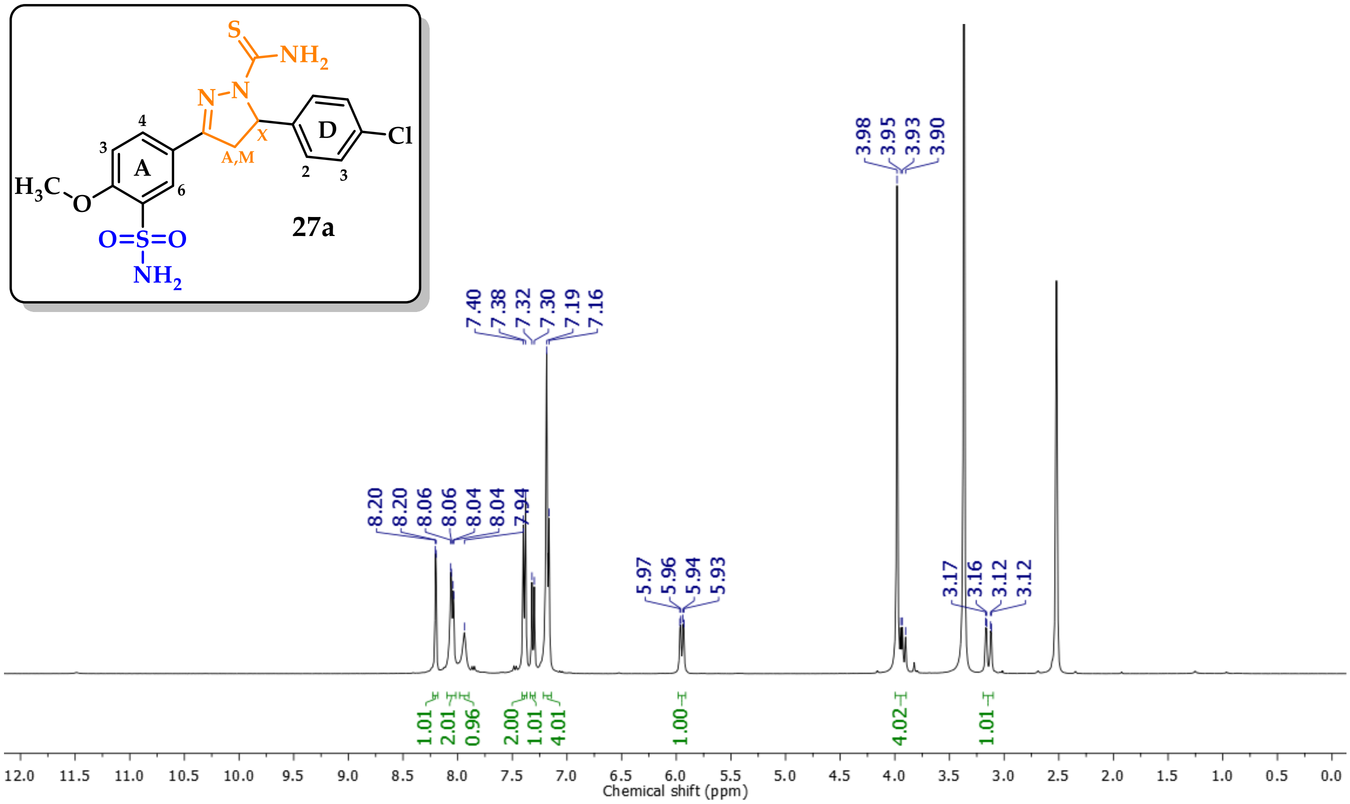


$^{1}$H NMR (400 MHz, DMSO-$d_6$) spectrum of **27a**.

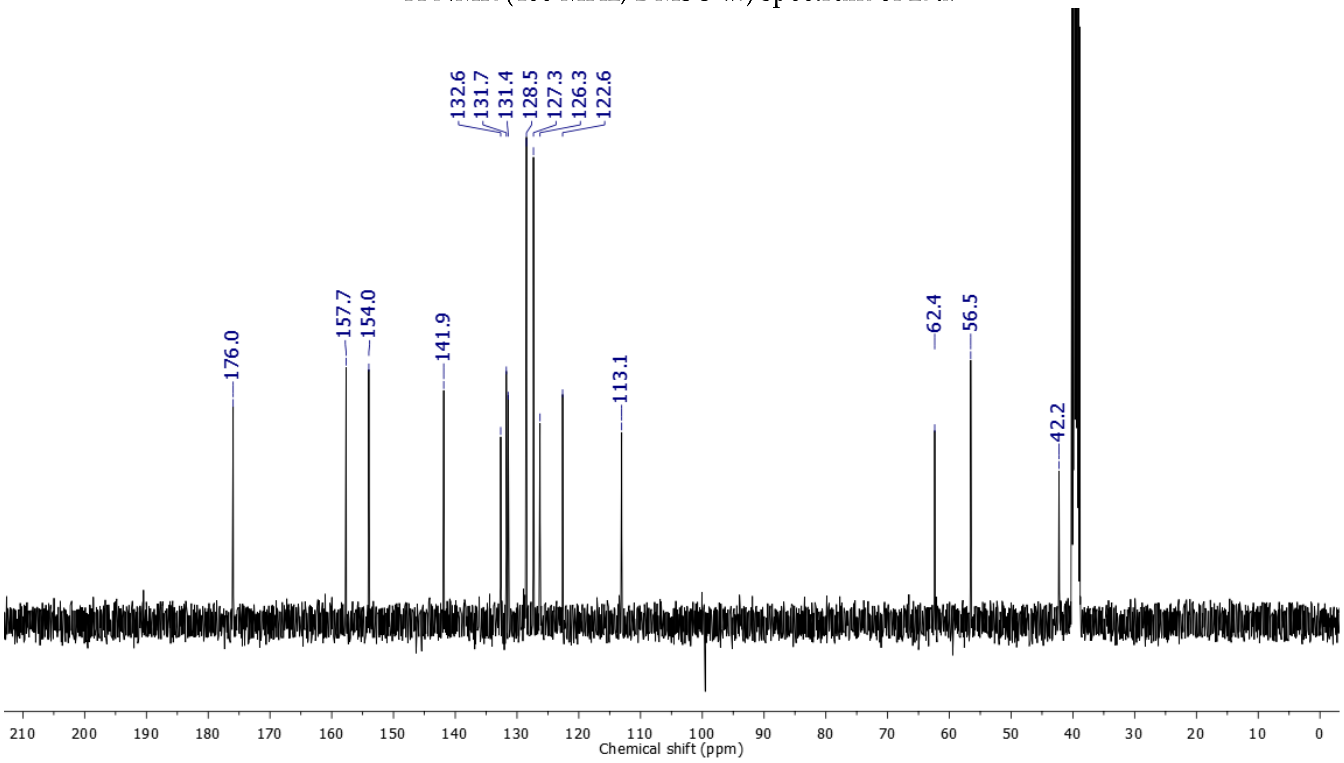


$^{13}$C NMR (101 MHz, DMSO-$d_6$) spectrum of **27a**.

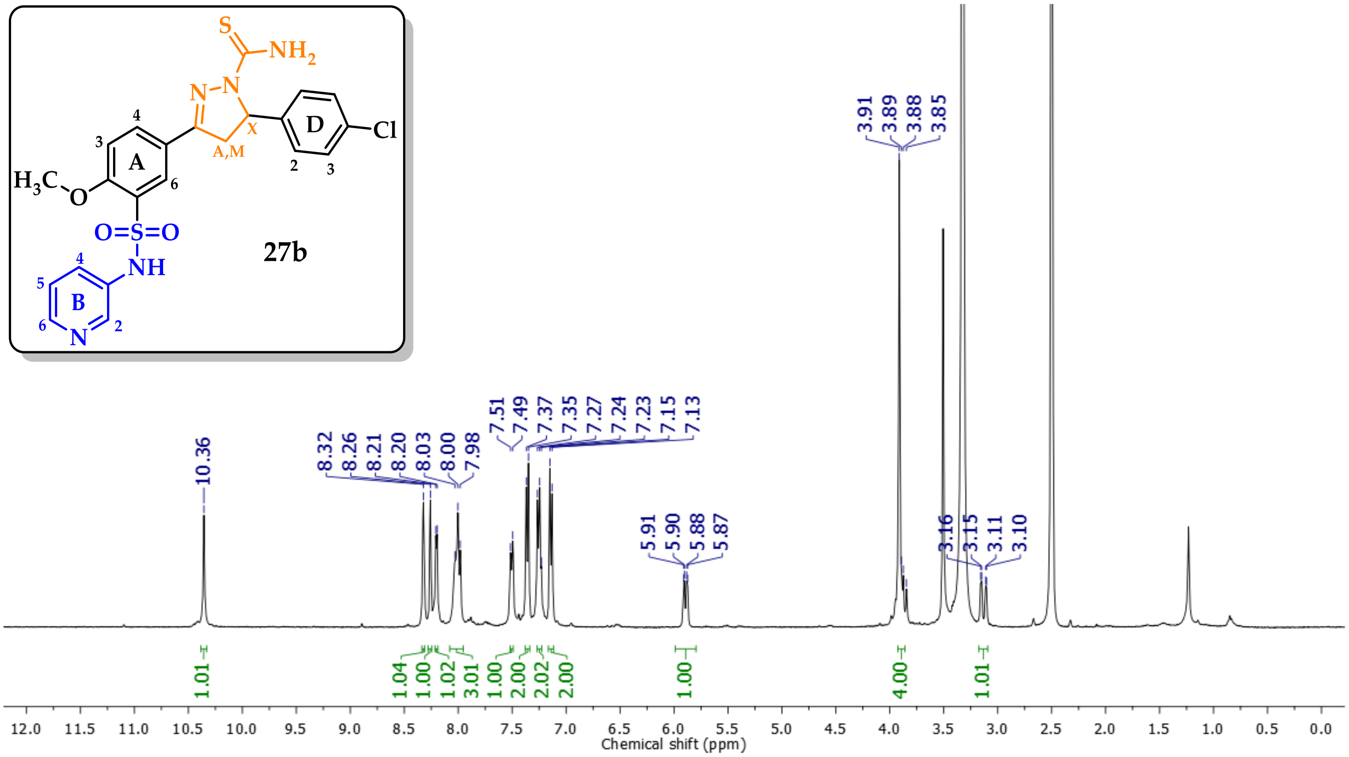


$^{1}$H NMR (400 MHz, DMSO-$d_6$) spectrum of **27b**.

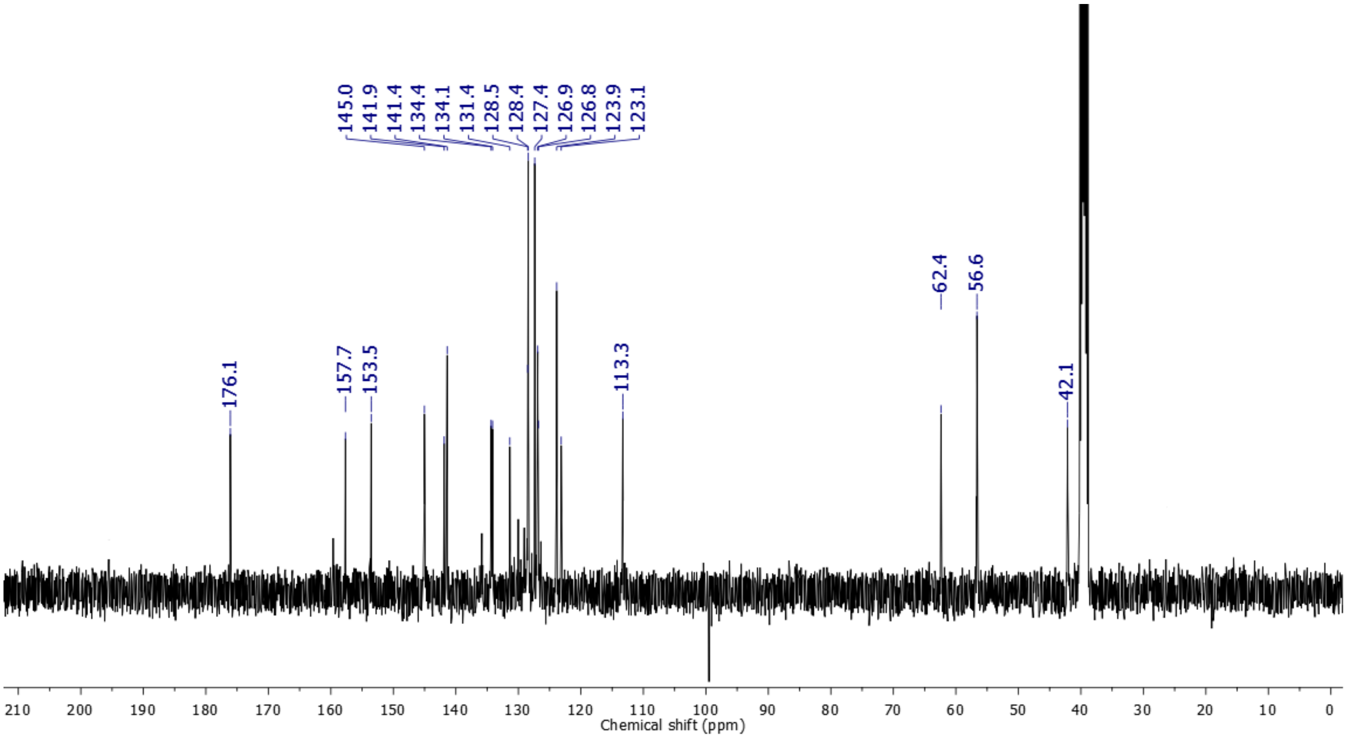


$^{13}$C NMR (101 MHz, DMSO-$d_6$) spectrum of **27b**.

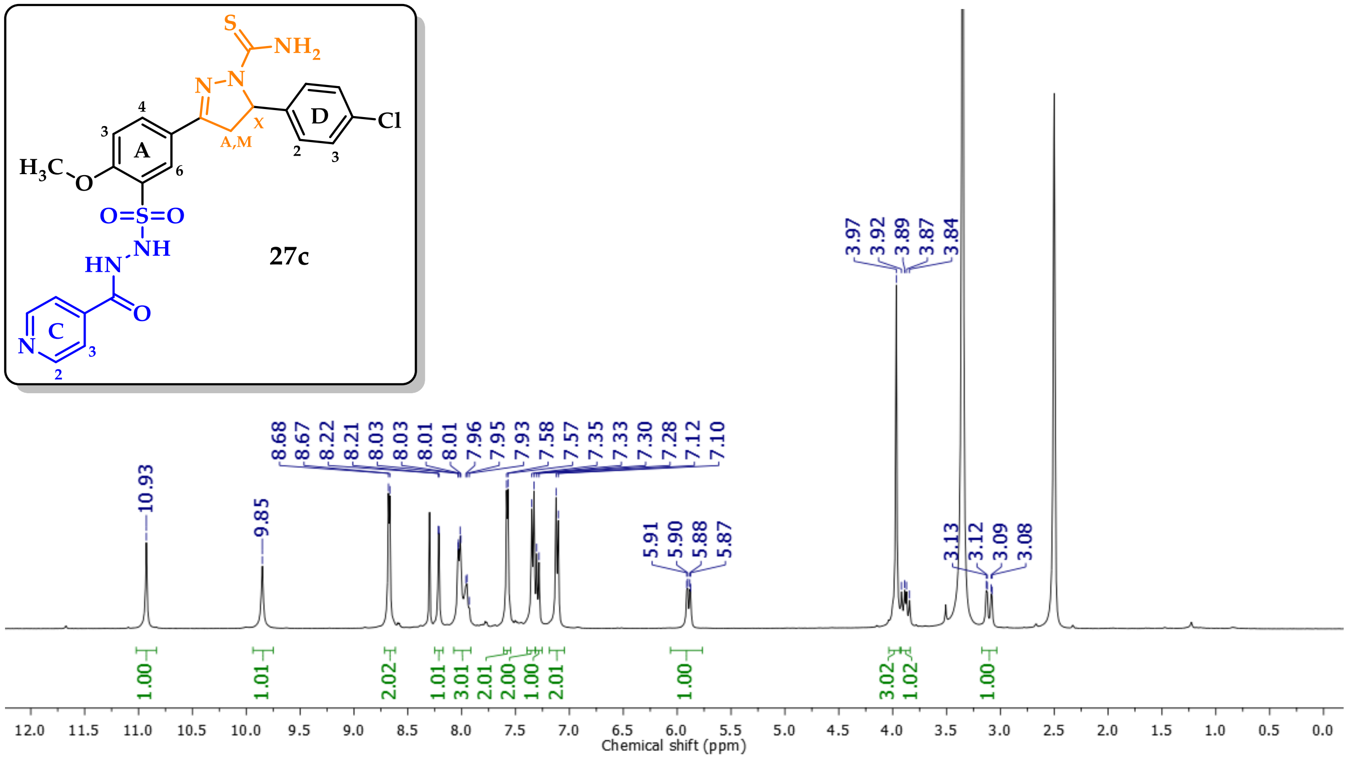


$^1H$ NMR (400 MHz, DMSO-*d*₆) spectrum of **27c**.

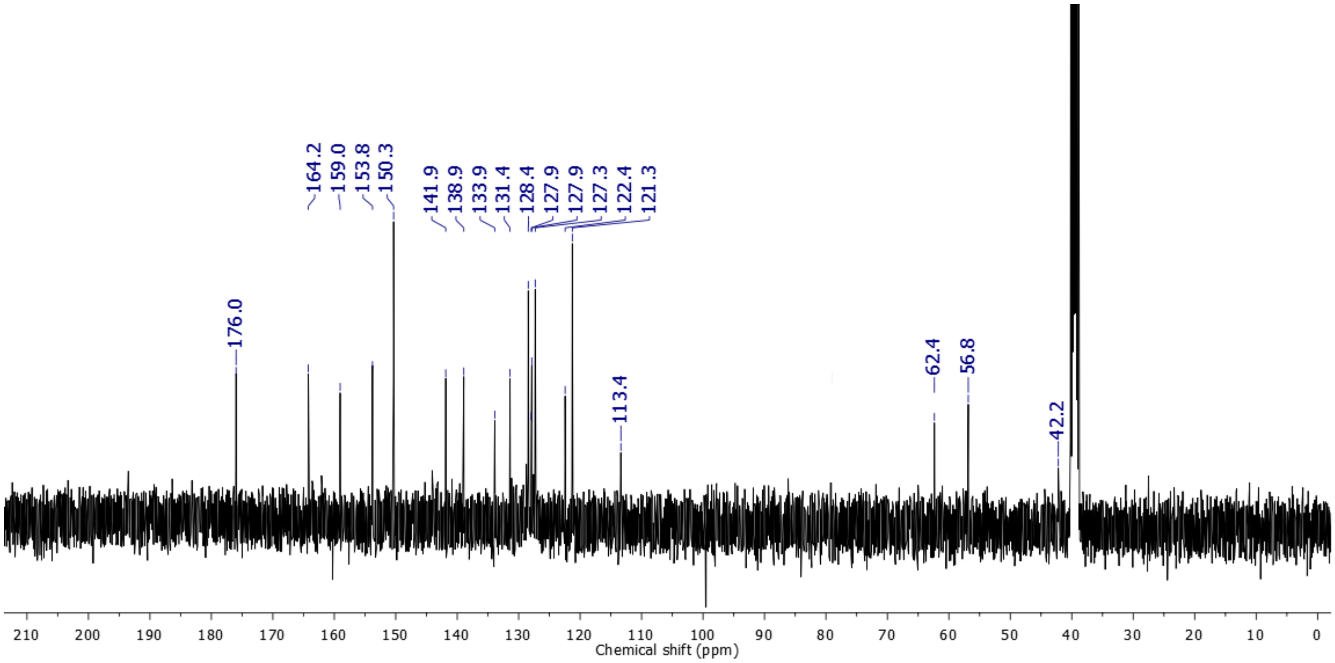


$^{13}C$ NMR (101 MHz, DMSO-*d*₆) spectrum of **27c**.

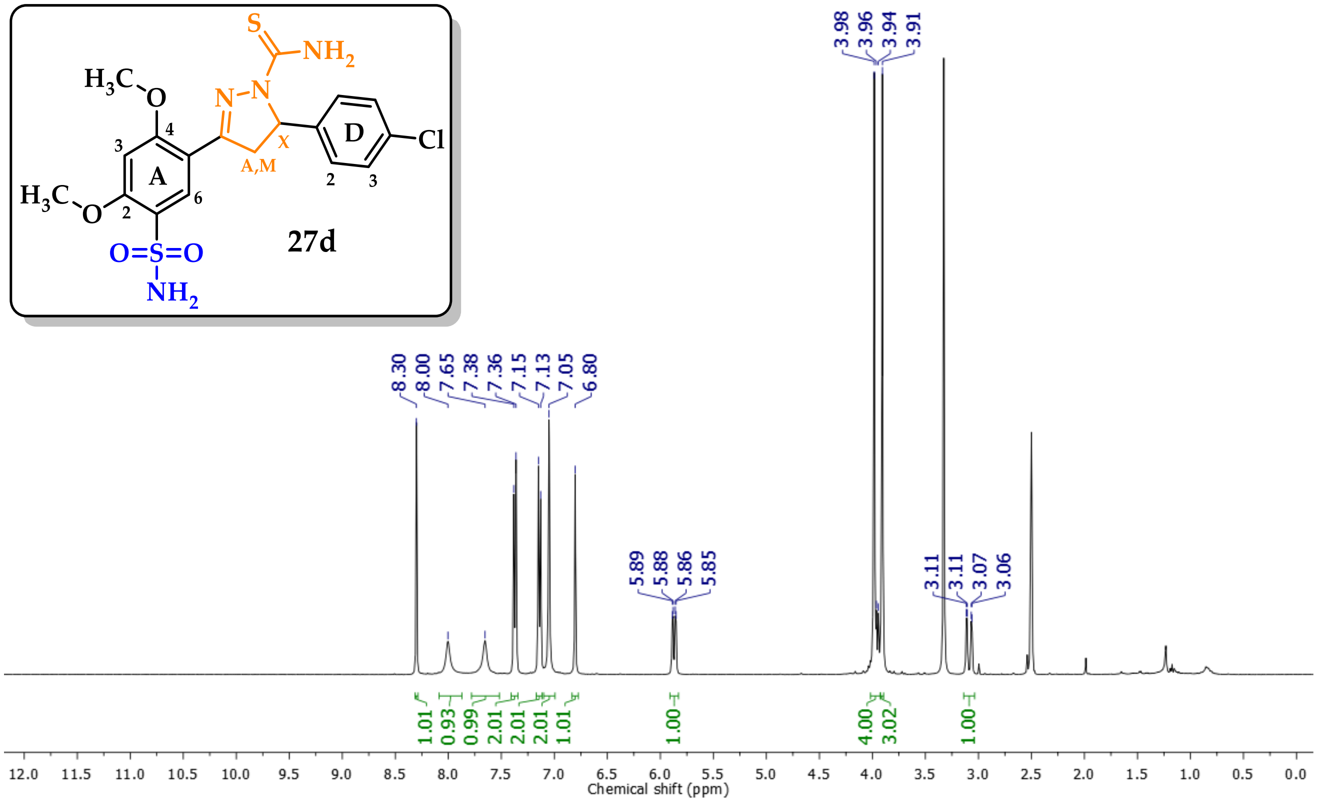


$^{1}$H NMR (400 MHz, DMSO-*d*6) spectrum of **27d**.

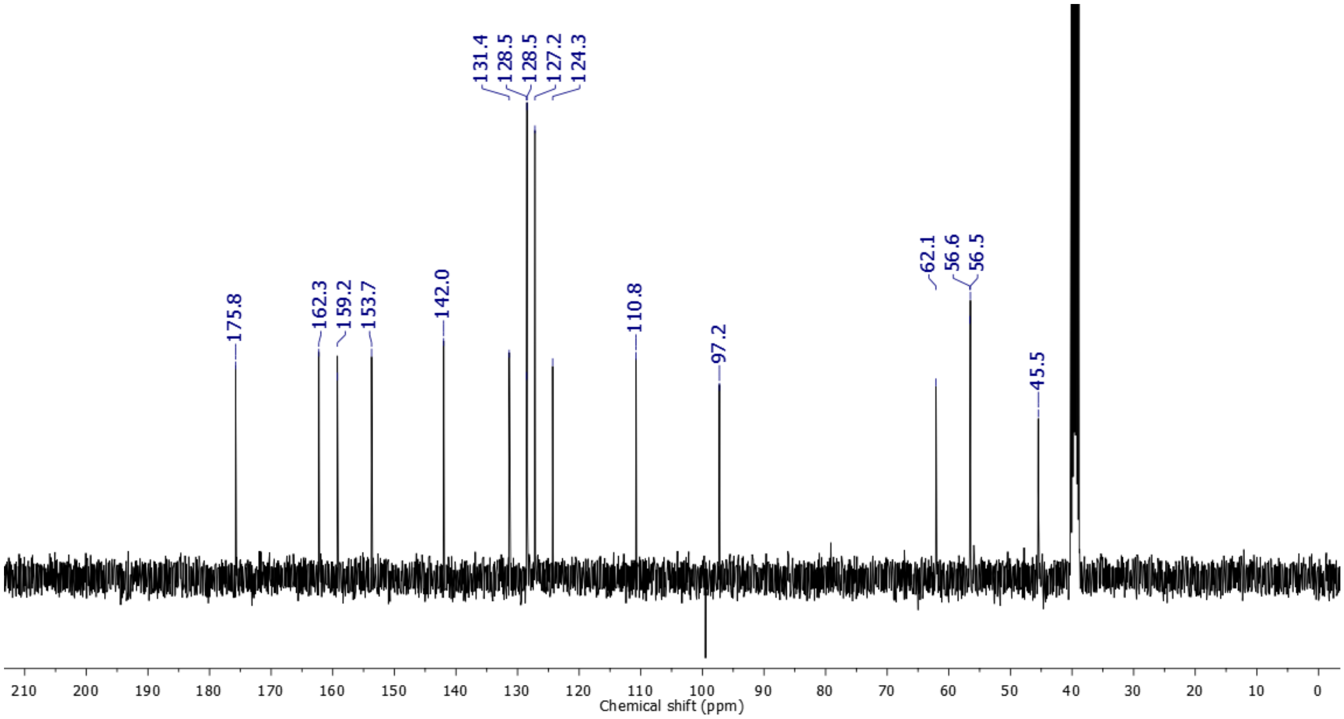


$^{13}$C NMR (101 MHz, DMSO-*d*6) spectrum of **27d**.

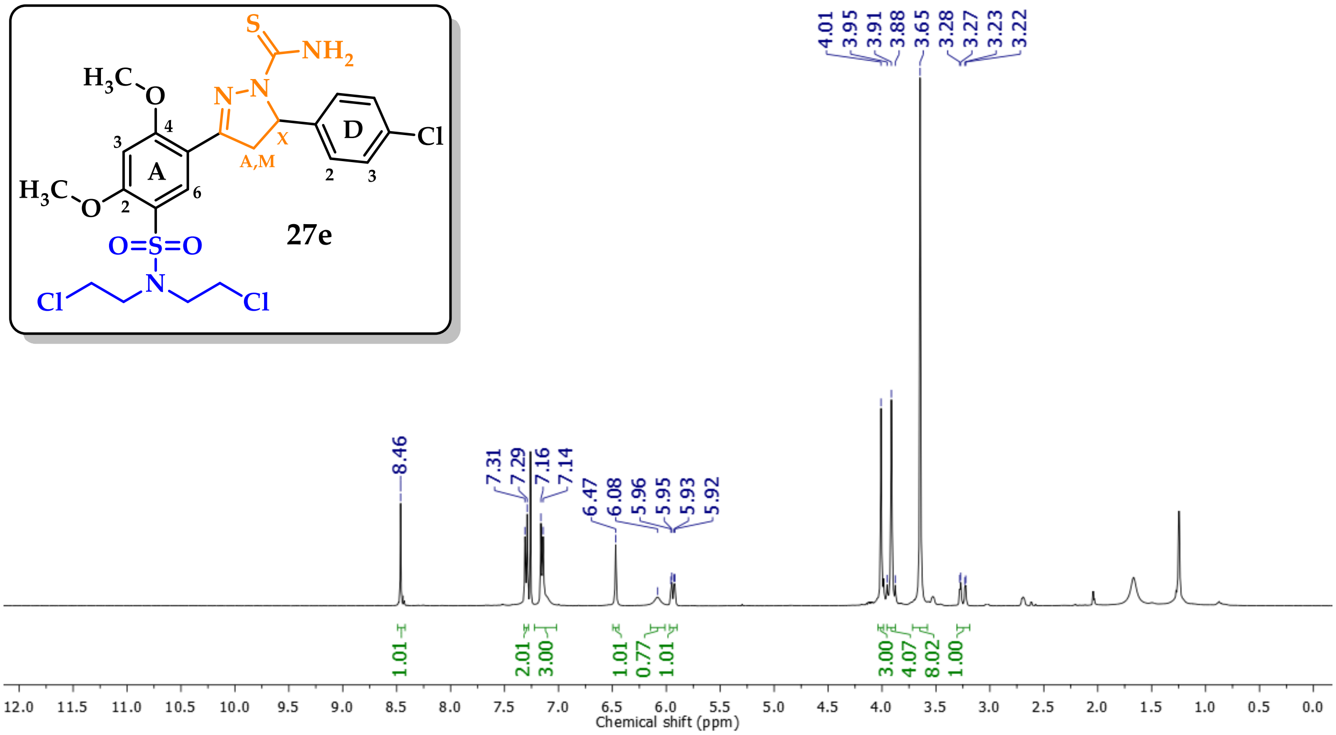


$^{1}H$ NMR (400 MHz, $CDCl_3$) spectrum of **27e**.

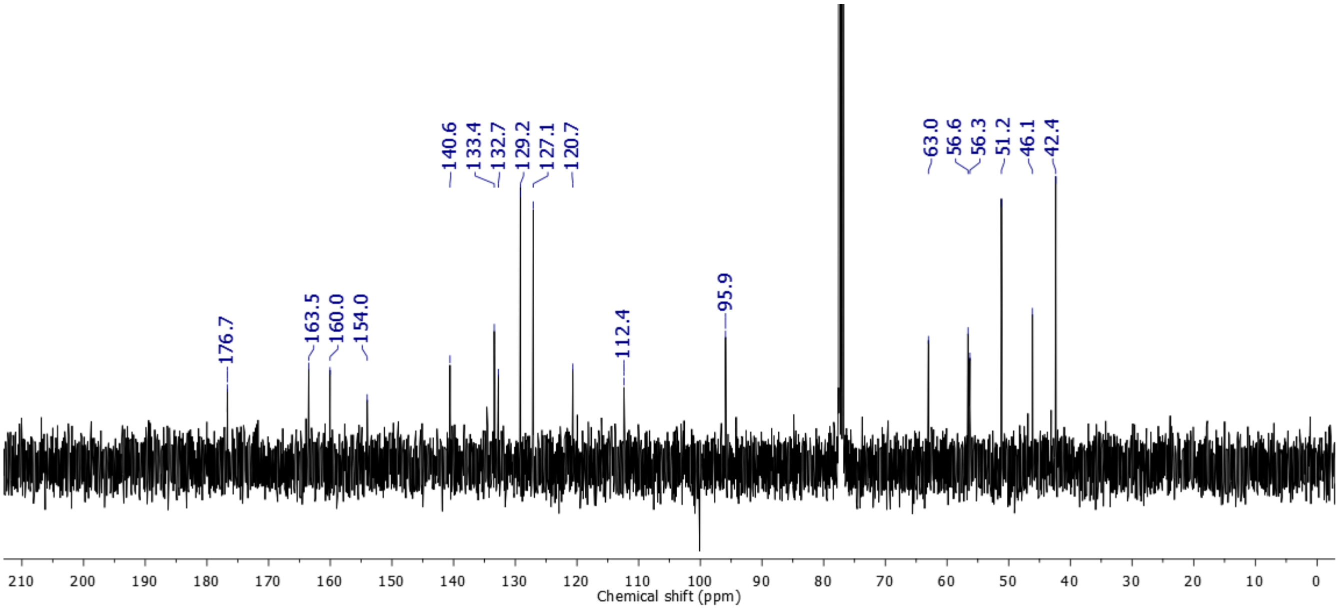


$^{13}C$ NMR (101 MHz, $CDCl_3$) spectrum of **27e**.

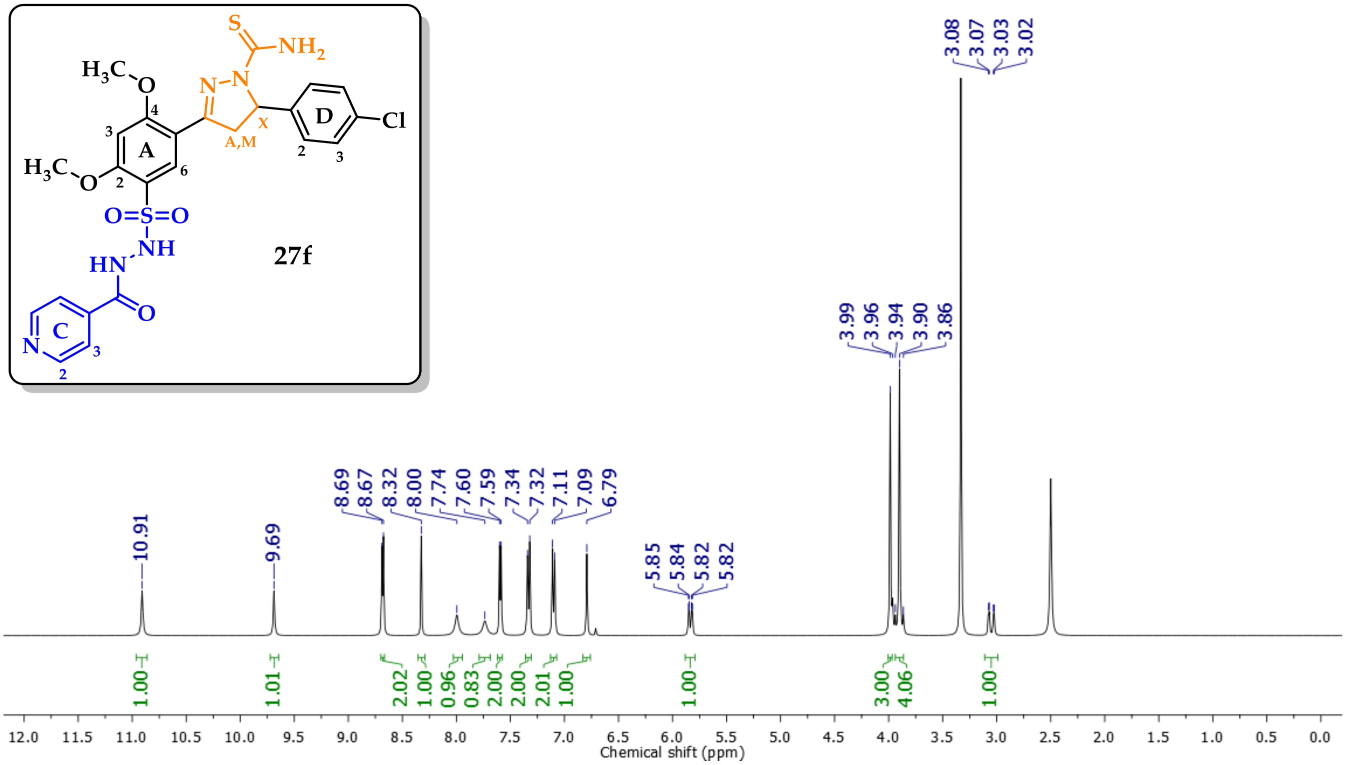


$^{1}H$ NMR (400 MHz, DMSO-$d_6$) spectrum of **27f**.

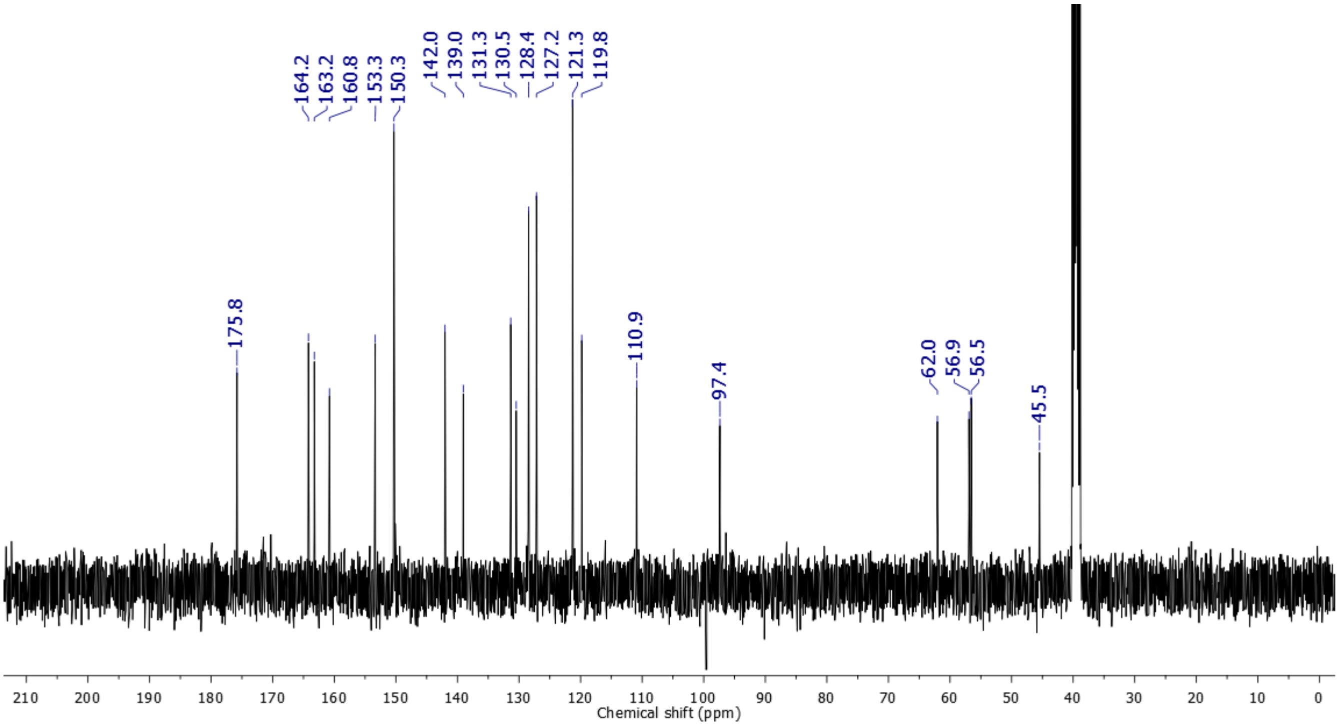


$^{13}C$ NMR (101 MHz, DMSO-$d_6$) spectrum of **27f**.

## Table S1. Single Crystal Diffraction Experimental Details

| Compound | **8b** | **14a** | **14b** |
|---|---|---|---|
| CCDC code | *2191609* | *2191607* | *2191610* |
| Crystal data | | | |
| Chemical formula | $C_{10}H_{13}NO_5S$ | $C_{15}H_{15}N_3O_5S$ | $C_{16}H_{17}N_3O_6S·H_2O$ |
| $M_r$ | 259.27 | 349.36 | 397.40 |
| Crystal system, space group | Monoclinic, $P2_1/c$ | Triclinic, $P\bar{1}$ | Triclinic, $P\bar{1}$ |
| Temperature (K) | 100 | 100 | 100 |
| $a$, $b$, $c$ (Å) | 10.1290 (3), 7.8633 (3), 15.0022 (6) | 8.1469 (4), 8.6691 (5), 11.2683 (6) | 10.4346 (4), 13.3686 (6), 19.7728 (8) |
| α, β, γ (°) | 90, 104.850 (1), 90 | 94.600 (2), 95.040 (2), 105.641 (2) | 84.686 (2), 87.900 (2), 73.780 (2) |
| $V$ (Å$^3$) | 1154.98 (7) | 758.92 (7) | 2636.89 (19) |
| $Z$ | 4 | 2 | 6 |
| Radiation type | Mo $K\alpha$ | Mo $K\alpha$ | Mo $K\alpha$ |
| μ (mm$^{-1}$) | 0.29 | 0.25 | 0.23 |
| Crystal size (mm) | 0.25 × 0.12 × 0.07 | 0.28 × 0.28 × 0.16 | 0.15 × 0.14 × 0.13 |
| | | | |
| Data collection | | | |
| Diffractometer | Bruker D8 Venture | | |
| Absorption correction | MULTISCAN; SADABS 2016/2 | | |
| $T_{min}$, $T_{max}$ | 0.619, 0.746 | 0.708, 0.746 | 0.708, 0.746 |
| No. of measured, independent and observed [$I > 2\sigma(I)$] reflections | 25007, 2651, 2356 | 38280, 3489, 2991 | 121286, 12120, 10461 |
| $R_{int}$ | 0.047 | 0.063 | 0.044 |
| (sin θ/λ)$_{max}$ (Å$^{-1}$) | 0.650 | 0.650 | 0.650 |
| | | | |
| Refinement | | | |
| $R[F^2 > 2\sigma(F^2)]$, $wR(F^2)$, $S$ | 0.029, 0.076, 1.05 | 0.035, 0.095, 1.10 | 0.032, 0.100, 1.05 |
| No. of reflections | 2651 | 3489 | 12120 |
| No. of parameters | 157 | 219 | 787 |
| H-atom treatment | H-atom parameters constrained | H-atom parameters constrained | H atoms treated by a mixture of independent and constrained refinement |
| $\Delta\rangle_{max}$, $\Delta\rangle_{min}$ (e Å$^{-3}$) | 0.31, -0.42 | 0.36, -0.44 | 0.37, -0.49 |

| Compound | **17d** | **20f** | **17c** |
|---|---|---|---|
| CCDC code | 2191609 | 2191607 | 2191610 |
| Crystal data | | | |
| Chemical formula | $C_{18}H_{19}NO_6S$ | $C_{24}H_{23}N_3O_9S$ | $C_{18}H_{19}NO_5S$ |
| $M_r$ | 377.40 | 529.51 | 361.40 |
| Crystal system, space group | Monoclinic, $P2_1/n$ | Triclinic, $P\bar{1}$ | Orthorhombic, *Pbcn* |
| Temperature (K) | 100 | 100 | 100 |
| *a*, *b*, *c* (Å) | 8.7129 (3), 15.0324 (5), 13.6114 (4) | 8.1544 (4), 12.3336 (7), 12.7882 (7) | 10.4991 (3), 10.1829 (3), 32.3481 (9) |
| α, β, γ (°) | 90, 107.554 (1), 90 | 86.224 (2), 71.888 (2), 89.788 (2) | 90, 90, 90 |
| $V$ (Å$^3$) | 1699.75 (10) | 1219.58 (11) | 3458.38 (17) |
| $Z$ | 4 | 2 | 8 |
| Radiation type | Mo $K\alpha$ | Mo $K\alpha$ | Mo $K\alpha$ |
| μ (mm$^{-1}$) | 0.23 | 0.19 | 0.22 |
| Crystal size (mm) | 0.24 × 0.20 × 0.12 | 0.34 × 0.21 × 0.18 | 0.20 × 0.15 × 0.14 |
| | | | |
| Data collection | | | |
| Diffractometer | Bruker D8 Venture | | |
| Absorption correction | MULTISCAN; SADABS 2016/2 | | |
| $T_{min}$, $T_{max}$ | 0.691, 0.746 | 0.705, 0.746 | 0.683, 0.746 |
| No. of measured, independent and observed [$I > 2\sigma(I)$] reflections | 28650, 3910, 3325 | 55766, 5603, 5018 | 29731, 3966, 3484 |
| $R_{int}$ | 0.051 | 0.039 | 0.045 |
| $(\sin \theta/\lambda)_{max}$ (Å$^{-1}$) | 0.650 | 0.650 | 0.650 |
| | | | |
| Refinement | | | |
| $R[F^2 > 2\sigma(F^2)]$, $wR(F^2)$, $S$ | 0.032, 0.087, 1.04 | 0.039, 0.109, 1.04 | 0.035, 0.093, 1.05 |
| No. of reflections | 3910 | 5603 | 3966 |
| No. of parameters | 238 | 336 | 229, 2 restraints |
| H-atom treatment | H-atom parameters constrained | H-atom parameters constrained | H-atom parameters constrained |
| $\Delta\rangle_{max}$, $\Delta\rangle_{min}$ (e Å$^{-3}$) | 0.34, -0.42 | 1.52, -0.34 | 0.42, -0.47 |

Computer programs: *APEX3* v2019.1-0 (Bruker Nano Inc., 2019), *SAINT* V8.38A (Bruker AXS Inc., 2017), *SHELXT*–2014/5 (Sheldrick, 2014), *SHELXL*–2018/2 (Sheldrick, 2018).
